# Green Drug Discovery: Novel Fragment Space from the Biomass-Derived Molecule Dihydrolevoglucosenone (Cyrene^TM^)

**DOI:** 10.3390/molecules28041777

**Published:** 2023-02-13

**Authors:** Tom Dekker, Jaap W. Harteveld, Gábor Wágner, Max C. M. de Vries, Hans Custers, Andrea C. van de Stolpe, Iwan J. P. de Esch, Maikel Wijtmans

**Affiliations:** Amsterdam Institute of Molecular and Life Sciences (AIMMS), Vrije Universiteit Amsterdam, 1081 HZ Amsterdam, The Netherlands

**Keywords:** drug discovery, fragment-based drug discovery, organic synthesis, Cyrene^TM^, sustainability, biomass

## Abstract

Biomass-derived molecules can provide a basis for sustainable drug discovery. However, their full exploration is hampered by the dominance of millions of old-fashioned screening compounds in classical high-throughput screening (HTS) libraries frequently utilized. We propose a fragment-based drug discovery (FBDD) approach as an efficient method to navigate biomass-derived drug space. Here, we perform a proof-of-concept study with dihydrolevoglucosenone (Cyrene^TM^), a pyrolysis product of cellulose. Diverse synthetic routes afforded a 100-membered fragment library with a diversity in functional groups appended. The library overall performs well in terms of novelty, physicochemical properties, aqueous solubility, stability, and three-dimensionality. Our study suggests that Cyrene-based fragments are a valuable green addition to the drug discovery toolbox. Our findings can help in paving the way for new hit drug candidates that are based on renewable resources.

## 1. Introduction

Renewable green resources from nature have the potential to serve as alternative input for both large-scale chemical production of consumer products [1,2], as well as for biomedical applications [3,4,5]. The underlying “biobased economy” has secured priority on the agendas of national and international governmental institutions [6]. The wish to use molecules derived from biomass as feedstock for the green production processes of pharmaceuticals is an integral part of these efforts [7,8]. Pyrolysis (heating) of biomass polymers has the potential to provide smaller and versatile offspring molecules [9,10] that may retain several fingerprints of the parents’ molecular properties. That is, depending on the biomass resource and pyrolysis conditions used, the offspring molecules can themselves be considerably biogenic, and biogenic molecules originating from natural products have been shown to have advantageous properties in drug research [11,12,13]. As a result of technological advances in pyrolysis processing, biomass-derived products are only now becoming readily available. Thus, these offspring molecules and the scaffolds that they represent have been underexplored in (drug) research. This means that the classical high-throughput screening (HTS) libraries that are being used in drug research have vastly lacked biomass-derived molecules. The time appears right for green drug discovery inspiration by biogenic biomass-derived molecules [14]. However, populating HTS libraries with a significant number of biomass-derived drug-like molecules is a highly inefficient approach. Instead, we consider fragment-based drug discovery (FBDD) as an ideal platform to significantly increase the chances that biomass-derived molecules are identified as suitable drug development starting points. In the last 20 years, FBDD has proven an attractive alternative to HTS for discovering new biologically active molecules [15,16,17]. FBDD uses comparatively small molecules (<300 Da), and therefore a reduced library size (~1000 compounds), to traverse the vast landscape of conceivable molecular structures (chemical space) and find starting points for drug discovery [18]. Fragment hits can be efficiently grown to drug-like molecules along synthetic growth vectors. The promise of FBDD is evident by the approval of six drugs originating from FBDD approaches as of January 2022 [19]. Biogenic fragments obtained from complex natural products have already been showcased [20,21,22,23], but were obtained from scarce, non-renewable resources through lengthy syntheses. In contrast, we are interested in exploring readily available biomass pyrolysis products as biogenic and green precursors for fragment libraries. The FBDD approach and its concise library sizes can uniquely ensure that green molecular entities are present at the very start of the drug discovery process, thus increasing the chances that these entities find their way to innovative medicines. Here, we disclose our efforts to this end using dihydrolevoglucosenone (Cyrene^TM^), a product now abundantly available from biomass cellulose, and show how Cyrene-based molecules can access novel fragment space.

## 2. Results

### 2.1. Selection of Precursor

**Figure 1:**
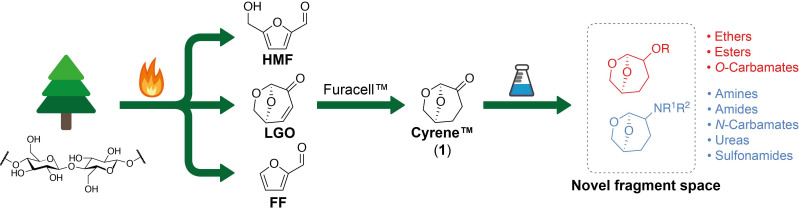
The approach pursued in this work. Pyrolysis of cellulose/wood gives, among others, LGO, which has become readily available as its reduced analog Cyrene. Functionalization of Cyrene with an array of functional groups provides a set of *O*- and *N*-substituted “green fragments”.

Pyrolysis of cellulose gives, amongst many other minor products, levoglucosenone (LGO), hydroxymethylfurfural (HMF), and furfural (FF) as major products (Figure 1) [24]. LGO can be further transformed into the biogenic biomass product dihydrolevoglucosenone (Cyrene, **1**) using the Furacell^TM^ process [25]. Cyrene was originally developed by York University in conjunction with the Circa Group [26]. It is propagated and intensively investigated as a “green alternative” to classical aprotic dipolar solvents because of its dipole moment (comparable polarity to DMF/NMP) [27], attractive hydrogen bond accepting capacity, relative stability of the acetal moiety [28], and low toxicity [29]. Cyrene won the Bio-based World News’ European Bio-based Innovation Award in 2017 and the Environmental Leader’s Top Product of 2019 award [30]. Cyrene is a clear offspring of its parent sugar molecule and key properties of the latter are inherited in its structure, e.g., well-defined stereochemistry and 3D structure, high oxygen-content, and geminal C–O bonds.

We recognized that Cyrene embodies a molecule with several interesting properties for our objectives: (1) It is now readily available at low cost, as its production became possible at multi-ton plant scale in 2019, and an EU-funded plant in France is also planned [31]; (2) Cyrene leaves room for growing to a library of fragments, owing to its very small size (molecular weight (MW) = 128 Da); (3) The Cyrene scaffold is virtually absent in “chemical drug space”. Only a handful of scattered articles have described its scaffold in a medicinal chemistry context [32,33,34,35,36], none of which in FBDD, and Cyrene has not been identified as a significant scaffold in natural products [37]. Interestingly, during the course of our work, a review on geminal diheteroatomic motifs drew attention to the potential of Cyrene in drug design [28]; (4) The structure of Cyrene has high 3D character, a feature typically underrepresented in current (commercial) fragment libraries [38,39]. How to attain a proper balance of 2D and 3D features in fragments is subject to ongoing investigations in the field [40,41], as the increased complexity of 3D fragments may give lower hit rates upon screening [42,43]. (5) Cyrene is highly water-soluble. This is a key property in drug discovery in general, and in particular for FBDD since high concentrations are required for biological testing of fragments [38]. Although ketone hydration to a *gem*-diol plays a key role in this high solubility [25], non-hydrated Cyrene has a high dipolarity comparable to dipolar aprotic solvents (Kamlet–Abboud–Taft analysis) [26]. Indeed, the glycol acetal of Cyrene is still water-miscible [44]. It was recently reported that *iso*-Cyrene, with the ketone shifted by one position, does not undergo hydration and as a result does not fully mix with water, but solutions of 10 mM of *iso*-Cyrene in water could nonetheless be obtained [45]. This indicates that Cyrene-derived fragments that lack the ketone, and thus also lack the ability to undergo hydration, could still have pharmacologically relevant solubility (i.e., high μM).

### 2.2. Design of Fragments

In principle, Cyrene offers two growth vectors: the electrophilic carbonyl moiety, and the nucleophilic enolizable α-methylene group. Hughes et al. have systematically exploited the latter strategy as a means toward intermediates of pharmaceutical relevance [33]. In the current proof-of-concept approach, we focused on the complementary growth vector, i.e., the electrophilic carbonyl moiety. Remarkably, growing from the carbonyl group with standard functional groups has seen extremely little precedent in the literature, as judged from a search with the Reaxys search engine (June 2022, see Supplementary Materials Table S1). This scarcity is most evident for growing with a nitrogen-based substituent, for which only two compounds are known (–NH_2_ and –N_3_) [46,47,48]. We employed a modular strategy that allowed the assembly of a Cyrene-derived fragment library by reductive replacement of the carbonyl moiety with oxygen- but mostly nitrogen-based functional groups, many of which have a prominent position in FBDD and medicinal chemistry at large (Figure 1). The Cyrene skeleton offers two hydrogen bond acceptor (HBA) atoms and is expected to compensate for an increase in LogP values that would result from the introduction of any apolar substituents. Except for esters and ethers, all groups explored by us present NH moieties (possessing dual H-bond accepting and donating properties), a feature recently highlighted in multiple FBDD survey studies as highly attractive [49,50]. Given the already high intrinsic 3D character of the Cyrene core and its inability to provide aromatic π–π protein–ligand interactions, we chose to pursue a more balanced character by appending an aromatic moiety to virtually all compounds (except **8b**, **9c–d**, **11i**, and **12f–g**). The chromophore thus incorporated would also facilitate product purification. We aimed at incorporating both aromatic and heteroaromatic groups with a high structural and positional diversity of aromatic substituents. In all efforts, MW ≤300, and heavy atom count (HAC) ≤20 was used as a limit for the product fragments [18].

### 2.3. Synthetic Routes

Acid- and base-compatibility factors need to be considered when performing synthetic chemistry with Cyrene. The acetal moiety is relatively stable and does not react in the presence of weak acids and, e.g., when heated with trifluoroacetic acid (TFA) at 60 °C for 18 h, but Cyrene does react with 2.0 M HCl [51]. Reactivity of the acetal core in the presence of strong acids was recently also disclosed for the regioisomeric *iso*-Cyrene [45]. Therefore, strong acids were avoided during the course of our work, and we found no evidence of significant decomposition of the acetal core. The base sensitivity of Cyrene has been thoroughly explored [52], but the underlying dimerization risk due to the enolizable α-position is eliminated once the sp^2^ center has been reduced to an sp^3^ center. In all, we selected a synthetic strategy mostly based on two key building blocks, i.e., **2** and **4**. Alcohol **2** was prepared on a large scale by NaBH_4_-based reduction of Cyrene, affording an inseparable ~9/1 mixture of diastereomers with the *endo* isomer reportedly being the major product, which we confirmed (vide infra) [53]. *Exo*-amine **4** has been reported through a 4-step synthesis route starting from LGO [46], and can also be accessed from LGO by an enzymatic transamination reaction as part of a process recently awarded the Peter J. Dunn Award for Green Chemistry 2022 [48,54]. For scaling purposes and to get access to the *endo* isomer as well, we resorted to an alternative route consisting of Ni-catalyzed hydrogenation of the known oxime of Cyrene (**3**) [55,56]. This sequence was routinely scaled to 20-g scale, affording an inseparable ~6:4 mixture of diastereomers with the tentatively assigned major diastereomer being the *endo* isomer. Exploratory efforts to separate the isomers through phtaloyl protection afforded some success, but this approach was not easily scalable within the timeframes of the project.

**Scheme 1:**
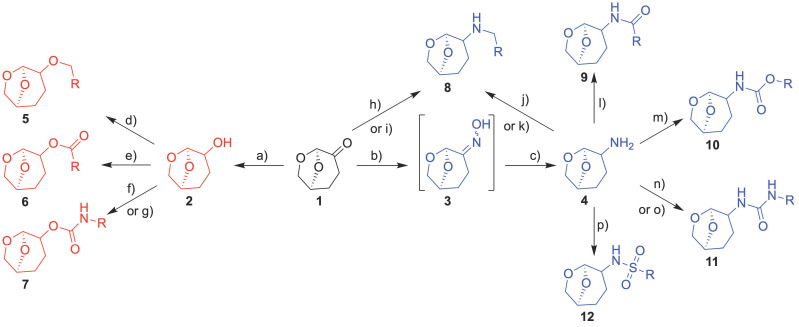
Synthetic routes. (a) NaBH_4_, MeOH, 0 °C → rt, 2 h, 99%; (b) NH_2_OH·HCl, pyridine, rt, 30 min, 96%; (c) RaNi, NH_3_, MeOH, H_2_, rt, 16 h, 99%; (d) [i] NaH, DMF, 0 °C; [ii] RCH_2_X, 0 °C→ rt, overnight, 6–71%; (e) RCOOH, EDCI, DMAP, DIPEA, DCM, rt, overnight, 4–47%; (f) For **7a,b**: RNCO, Et_3_N, THF, rt–50 °C, overnight–3 d, 32–33%; (g) For **7c,d**: [i] NaH or Et_3_N, THF, rt, 0.5–1 h; [ii] CDI, rt, 15 min–overnight; [iii] RNH_2_, rt–50 °C, overnight–2 d, 9–18%; (h) For **8a, 8c–e**, and **8g–k**: [i] RCH_2_NH_2_, AcOH, DCM, rt, 1–3 h; [ii] NaBH(OAc)_3_, rt, overnight–2 d, 5–86%; (i) For **8b** and **8f:** [i] RCH_2_NH_2_, MgSO_4_, DCM, rt, overnight; [ii] NaBH_4_, MeOH, rt, 2h, 11–47%; (j) For **8l**, **8m**, **8q**, and **8r**: [i] RCHO, DCE, rt, 1–5 h; [ii] NaBH(OAc)_3_, rt, overnight–2 d, 10–24%; (k) For **8n–p** and **8s:** [i] RCHO, MgSO_4_, DCM, rt, overnight; [ii] NaBH_4_, MeOH, rt, 2h, 11–33%; (l) RCOOH, EDCI, DMAP or HOBt, DIPEA or Et_3_N (if applicable), DCM or DMF, rt, overnight–3 d, 3–59%; (m) [i] CDI, THF, rt, overnight; [ii] ROH, NaH or Et_3_N, rt–150 °C, overnight–5d, 8–28%; (n) For **11a–f**: RNCO, DCM, 1–2.5 h, 36–70%; (o) For **11g–p**: [i] CDI, THF, rt, overnight; [ii] RNH_2_, Et_3_N (if applicable), rt–52 °C, overnight–3d, 12–63%; (p) RSO_2_Cl, Et_3_N, 0 °C → rt, 3.5 h–3 d, 5–50%. Yields refer to isolated compounds, as reported in Table 1.

In general, final fragments were synthesized from Cyrene or building blocks **2** and **4** using classical transformations (Scheme 1). Ethers **5** were prepared by benzylation using NaH to deprotonate the alcohol [57]. Treatment of **2** with 1-ethyl-3-(3-dimethylaminopropyl)carbodiimide (EDCI) and a carboxylic acid provided esters **6**. Owing to the low nucleophilicity of the OH moiety in **2**, preparation of *O*-carbamates **7** was relatively challenging but could be obtained either from the corresponding isocyanate (**7a–b**) or, with slightly improved results, through a 1,1′-carbonyldiimidazole (CDI)-based reaction with amines (**7c–d**). For amines **8**, we used two complementary strategies, either starting from Cyrene and an amine (**8a–k**) or from **4** and an aldehyde (**8l–s**), with both encompassing a direct as well as an indirect reductive amination approach. Amides **9** and *N*-carbamates **10** were prepared from **4** using standard conditions with EDCI and CDI, respectively. Ureas **11** were obtained from **4** after reaction with the corresponding isocyanate (**11a–f**) or through a reaction with CDI (**11g–p**). Last, treatment of **4** with sulfonyl chlorides furnished sulfonamides **12**. A total of 100 final fragments of eight different classes (**5–12**) were prepared, and structures are assembled in Table 1 and in Supplementary Materials Tables S2 and S3 (expanded version). For several compounds, it proved possible to get at least one (**7a**, **7b**, **8f**, **9n**, **9o**), but also two diastereomers (**8h,i**/**8q,r**/**9a,b**/**9f,g**/**9h,i**/**12a,b**/**12c,d**) separated. To unambiguously present the stereochemical results, the structures in Table 1 do not specify stereochemistry, but Supplementary Materials Table S3 includes proposed stereochemical assignments.

### 2.4. Stereochemistry

**Figure 2:**
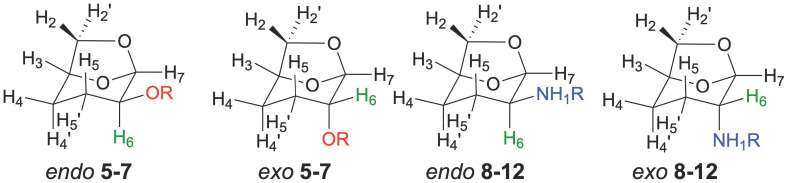
Depiction of the *endo* and *exo* configurations as well as of the numbering scheme used.

The employed reductive strategies yield an additional stereocenter. All O-analogs, i.e., ethers (**5**), esters (**6**), and *O*-carbamates (**7**), were prepared from highly diastereomerically enriched *endo*-**2** and virtually all were isolated as mixtures of two inseparable enantiopure diastereomers in an average diastereomeric ratio (d.r.) of ~12:1. Synthesis steps d–g (Scheme 1) do not provide an evident risk of stereochemical erosion and we therefore assume that the major diastereomer has the *endo* configuration in all isolated products **5**, **6**, and **7** (Figure 2, Supplementary Materials Table S3). For representative benzyl ether **5a**, 1D Nuclear Overhauser Effect (NOE) NMR analyses indeed indicate *endo* stereochemistry. As a further confirmation, the major ^1^H and ^13^C NMR signals for **5a** are identical to those reported for the *endo* isomer [53], while the minor ^13^C NMR signals in **5a** match those reported for the *exo* isomer (^1^H NMR data have not been reported for the *exo* isomer) [57].

Assignment of the stereochemistry in *N*-analogs proved to be more challenging, and success varied for several reasons: (1) As the d.r. value of **4** is small (~6:4), translation of the configuration of its major constituent to the isolated products (as done for *O*-analogs) in synthesis steps j–p (Scheme 1) is not possible; (2) Some products (**8a–k**) were prepared through a direct reductive amination on Cyrene without intermediacy of **4** (steps h and i, Scheme 1); (3) The extent to which diastereomers could be separated varied. Encouragingly, though, for several *N*-based classes (amines, amides, and sulfonamides) we were able to obtain at least one pair of two (reasonably) separated diastereomers (**8q**/**r**, **9f**/**g**, and **12c**/**d**, respectively). The proposed assignments of their relative stereochemistry were based on 1D NOE NMR experiments, supported by published X-ray structures of Cyrene derivatives and calculated low-energy conformations (MOE software). In brief, two crystal structures of *endo*-Cyrene derivatives clearly show that only one of the two protons at C^2^ (i.e., H^2^, Figure 2) is able to give significant NOE interactions with the protons of the aliphatic C^4^ and C^5^ groups in the shielded NMR region (ca. 1.5 ppm) [58], which allows distinction between H^2^ and H^2^′. The diastereomer that showed a significant NOE effect between H^2^ and H^1^ (**9f**/**g** and **12c**/**d**) or between H^2^ and the benzyl-CH_2_ moiety (**8q**/**r**), but a much lower (or absent) NOE interaction between H^2^ and H^6^, was assigned the *endo* configuration. In contrast, the diastereomer that showed a significant NOE effect between H^2^ and H^6^, but a much lower (or absent) NOE interaction between H^2^ and H^1^ (**9f**/**g** and **12c**/**d**) or between H^2^ and the benzyl-CH_2_ moiety (**8q**/**r**), was assigned the *exo* configuration. Having proposed the configurations for (reasonably) separated diastereomers in the amine (**8q,r**), amide (**9f,g**), and sulfonamide (**12c,d**) classes, we next identified resolved signals for each of those diastereomers pairs and used multiple appropriate ^1^H chemical shifts to propose the configuration of the two diastereomers in the remaining inseparable pairs within that same class. D.r. values were determined using signals that were sufficiently resolved between the major and minor diastereomer. The analyses suggest the stereochemical assignments of the isolated amine, amide, and sulfonamide products listed in Supplementary Materials Table S3, showing a bias toward enrichment with *endo* isomer. For other *N*-based classes (*N*-carbamates **10** and ureas **11**), the separation of diastereomers was not achieved and assignment of the relative stereochemistry for the major diastereomers in these classes was therefore not possible.

### 2.5. Physicochemical Properties

**Figure 3:**
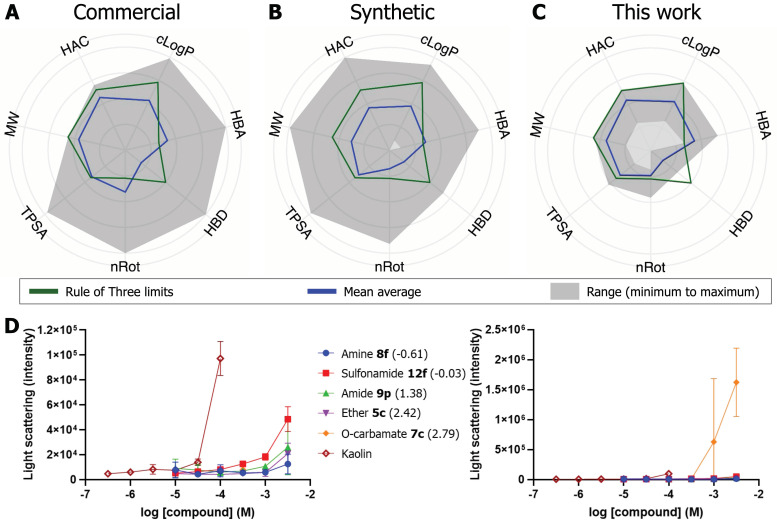
Physicochemical properties. (**A**–**C**) Distribution of calculated physicochemical properties of (**A**) Commercial “Fsp^3^” and “3D” fragment libraries extracted from a recent review [39], (**B**) 25 papers disclosing synthetic 3D fragment sets [40], and (**C**) the 100 compounds of the current paper. Axes scaling was based on the limits of all three libraries (**A**–**C**) combined and axis values are as follows: cLogP: [−2.4; 5.0], HBA: [0; 9], HBD: [0; 6], nRot: [0; 11], TPSA: [0; 140 Å^2^], MW: [95; 455 Da], HAC: [7; 27]). Values were calculated with KNIME software on the neutral chemical species. cLogP was calculated as SLogP. Mean average is depicted by the blue line. Ranges as defined by the minimum and maximum values are defined by the gray areas. The green line shows the “Rule of Three” limits (supplemented with a HAC limit of ≤20). (**D**) Nephelometry analysis of selected fragments and kaolin (control suspension) in HBSS buffer containing 1% DMSO. cLogD values are presented between brackets. Data points are the mean ± SD of values measured in triplo and normalized to kaolin control. Inflection points are considered to be a sign of aggregation and these are quantified using 3 times the SD of the average blank measurements. In the zoomed-in left panel, the 10^–3^ and 10^–2.5^ M points for **7c** are omitted for clarity, while the right panel contains the full set of data points.

In the presented library, a diversity of mostly aromatic groups was connected to the Cyrene scaffold through eight different functional groups, and other than MW < 300 and HAC ≤20 limits, no design factors were included. As a variation of Lipinski’s Ro5 for orally bioavailable clinical candidates, the Rule of Three (Ro3) has been proposed to estimate the suitability of compounds as fragments [59]. The Ro3 suggests the following limits: hydrophobicity (cLogP) ≤3, MW <300 Da, hydrogen bond donors (HBD) ≤3, HBA ≤3, rotatable bonds (nRot) ≤3 and topological polar surface area (TPSA) ≤60, with the latter two criteria not having been widely adopted [60]. More generally, these rules should be regarded as guidelines rather than as strict limits [60]. We calculated the 2D properties embodied in the Ro3, as well HAC values, of all 100 library members. The results are shown in Figure 3 and Table 2. For consistent comparison, we also applied these calculations to a database of commercial ”3D” and “Fsp^3^” fragment libraries extracted from a recent review (n = 29,866 molecules) [39], as well as to a set of 25 papers involving synthetic 3D fragment sets (n = 897 molecules) [40].

Despite the majority of the introduced substituents being aromatic, the average cLogP value is moderate (1.52), arguably as a result of compensation by the Cyrene moiety and the N/O heteroatom-based connections. The HBA count (3.92) is significantly higher than the Ro3 recommendation (≤3), which is a consequence of the two default acetal oxygen atoms in the Cyrene core supplemented by one or more HBA atoms in the used functional group. Violation of this Ro3 rule is in fact quite common, both in the commercial libraries and synthetic libraries, with an average HBA of 3.77 and 3.23, respectively. In terms of HBD count, ethers **5** and esters **6** present none, whereas all other classes by design offer at least one (**7–10**, **12**) or two (**11**) HBD atoms. Indeed, the average HBD count obtained (0.90) is lower than that of the commercial (1.18) and synthetic libraries (1.08). The nRot value on average (2.6) obeys the Ro3 guideline, but several members exceed the value. Given the rigidity of Cyrene, all rotations come from the connecting moiety (e.g., ethers **3** and amines **8** have three rotatable bonds) and are increased by any rotatable bonds in the substituent(s). Along similar reasonings as for the cLogP values, the polarity of the fragments as estimated from the TPSA value is still relatively high (53.0) but below the recommended limit (60). It is noted that the HAC value is high (17.9), which is undoubtedly a result of the default eight heavy atoms in the Cyrene moiety, but the average MW value (254 Da) still compares reasonably to that of the commercial (262 Da) and synthetic (232 Da) libraries. The Cyrene library has increased numbers of (saturated) rings and stereocenters (Table 2) compared to those of the commercial and synthetic libraries. Furthermore, while all three libraries have very similar percentages of carbon atoms and heteroatoms, in the Cyrene library, the distribution of heteroatoms is significantly skewed toward oxygen atoms at the expense of nitrogen atoms (Table 2).

### 2.6. Solubility and Stability

Given the expectation that the Cyrene skeleton can induce pharmacologically relevant solubilities in buffer by virtue of its polarity (*vide supra*), we were also interested in the solubility of the fragments in buffer. From the fragments with the lowest, median, and highest cLogD values, five different classes were selected (**8f**/**12f**, **9p**, and **5c**/**7c**, respectively). cLogD values were deemed more appropriate than cLogP owing to the ionizable groups in aqueous buffer at pH 7.4 for amines **8.** Solubility of these compounds was determined in HBSS buffer containing 1% DMSO using nephelometry, an accepted technique to estimate (fragment) solubility (Figure 3D) [38,61,62]. Compounds **8f, 12f**, **9p**, **5c**, and **7c** show no signs of precipitation or aggregation up to 3.2, 0.32, 1.0, 1.0, and 0.32 mM, respectively. Interestingly, the precipitate/aggregate formed by **7c** starting at 10^−3^ M induces an amount of scattering unusually high compared to the other compounds, the reason for which is unknown. In all, the exemplary nephelometry results bode well for the whole library in terms of the high concentrations typically required for fragment screening. We also note that virtually all final fragments are readily soluble at 10^–1^ M in DMSO.

The stability of the acetal moiety in Cyrene is known to be relatively high (*vide supra*) and it has been proposed that both a double anomeric effect as well as a destabilizing effect of an electron-withdrawing group (ketone) on any protonated acetal intermediate are key for this [28]. Both effects could still be operational in our final fragments. We incubated two *endo*/*exo* couples (**8h**/**8i** and **9h**/**9i**) as a 10^−2^ M solution in DMSO-d6 and as a 10^−4^ M solution in HBSS buffer/1% DMSO by NMR and LCMS analysis, respectively, at regular intervals at room temperature and after being frozen for three weeks. The results (Supplementary Materials Figure S1) show no or only minor reactivity. That is, only compounds **8h**/**8i** give ca. 2.5% of a byproduct after three weeks in buffer at room temperature, while no significant decomposition in DMSO was observed. No significant differences between the two diastereomers were observed in either couple.

### 2.7. Three-Dimensionality

Given the three-dimensional character of Cyrene, it is of interest to assess the three-dimensionality of the fragment library. Although Fsp^3^ is usually considered a non-ideal descriptor, it provides a qualitative measure, and the calculated Fsp^3^ for our library amounts to 0.55. In terms of Fsp^3^, commercial and synthetic 3D libraries have slightly higher Fsp^3^ values (0.67 and 0.60, respectively) than our library. Although the Cyrene part embodied in the final fragments boosts an Fsp^3^ sub-value of 1.0, virtually all members have one aromatic ring. The average overall Fsp^3^ value is nonetheless still higher than a proposed cut-off of 0.45 for three-dimensionality, and 96% of the library members meet this criterion [40].

**Figure 4:**
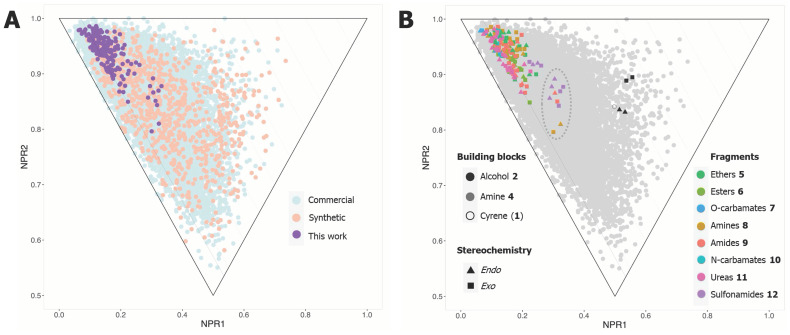
PMI plots of the Cyrene library. (**A**) Comparison of commercial “Fsp^3^” and “3D” fragment libraries extracted from a recent review [39], 25 papers disclosing synthetic 3D fragment sets [40], and the current work. (**B**) Comparison of Cyrene, building blocks **2** and **4**, the various fragment classes, and associated stereochemistry in the current work. The collected data points from the commercial and synthetic libraries in Figure 4B are light-gray, and the dashed circle represents all compounds that tend to be located more toward the disc–sphere axis (**8h,i**/**9l**/**12b,c,e**). Data points represent average PMI values per compound, obtained by averaging PMI values of individual conformations (ΔE_max_ ≤ 5 kcal·mol^−1^, RMSD > 0.1). The dashed straight line represents the cut-off for three-dimensionality (i.e., ΣNPR ≥ 1.07). For a given substituent both diastereomers are included as separate entries for the Cyrene library.

We also used principal moment of inertia (PMI) analysis, which is increasingly being embraced by the field as a three-dimensionality measure improved with respect to Fsp^3^ (Figure 4) [40,63,64]. For this purpose, per compound with a unique substituent, both diastereomers were incorporated as separate compounds (184 data points total). Of note, even though the appended groups were all aromatic groups, the Cyrene moiety was shown to still bestow a significant 3D character on the fragments. This is evidenced from the average ΣNPR value of 1.095 and from 77.2% of the fragments obeying the proposed PMI cut-off for 3D character (ΣNPR ≥ 1.07) [40]. Compared to the commercial and synthetic libraries (Figure 4A), the compounds are biased toward the “rod-like” region. When comparing the various classes (Figure 4B), few evident trends are visible. Carbamates (**7**, **10**) tend to occupy the top left of the graph. The encircled compounds, i.e., that tend to be located more toward the disc–sphere axis, contain a noteworthy high proportion of sulfonamides (**12b,c,e**), which could be a result of the non-flat nature of the sulfonamide bond. In addition, amine **8h,i**, the only *o*,*o*-disubstituted member in the amine series, and amide **9l**, the only compound with a “benzylic” substituent in the amide series, are in this region. In 86% of the cases, the *exo* isomer has a higher ΣNPR than the corresponding *endo* isomer, but the average absolute difference between *exo* and *endo* isomers (|ΔΣNPR|) is only 0.01 (0.00–0.06). Growing from only one exit vector results in an elongation of the molecule and this is evident by the shift toward the rod-vertex starting from the building blocks **1**, **2**, and **4** (Figure 4B).

## 3. Discussion

The use of biomass-derived compounds in making high-value products is an area of intense investigation, but the use in drug discovery seems only scarcely investigated. An approach based on FBDD allows fragments to be made based on biomass-derived precursors, and if hits are found, attractive molecular features and the use of renewable resources are incorporated from the start. The use of biomass is different from the existing inspirations used for ~20 years in FBDD. We describe here a fragment library prepared based on the biogenic precursor Cyrene, obtained on plant scale from the biomass-derived compound LGO. Cyrene has a unique 3D scaffold, appreciable polarity, and high enantiomeric purity. It has only recently become readily available, and arguably, as a result, very few drug discovery efforts with this compound have been undertaken. Convenient synthetic routes readily provided access to eight different classes of Cyrene-derived fragments. In general, reactions proceeded smoothly, albeit with variable isolated yields (3–86%). We contribute this to occasional incomplete conversions, appreciable aqueous solubility of some products leading to losses during extraction, and/or challenging chromatographic purifications. Where possible, the relative stereochemistry of pure diastereomers was assigned by NOE NMR, while for some diastereomer mixtures, the stereochemical composition was proposed by extrapolation. For the entire library, the combined analyses suggest that 55, 12, and 33 fragments have an *endo*, *exo* or unknown/ absent enrichment, respectively. Overall, the stereochemical control and purification of ensuing diastereomers leave room for improvement. Still, given the high enantiopurity of Cyrene, even a mixture of diastereomers only represents two stereoisomers. In the case where a library member consisting of a diastereomeric mixture gives a hit, additional efforts can separate the single diastereomers for further assays. The resulting 100-membered library comprises only novel compounds, except for **5a** and the very recently disclosed **8s [48,53]**, thus addressing unexplored parts of chemical space.

The library boasts acceptable physicochemical properties and all library members pass the PAINS filter [65]. The commonly calculated 2D properties of final fragments **5–12** (Figure 3) are comparable to those of commercial 3D and Fsp^3^ libraries and of synthetic 3D libraries. For cLogP, HAC, and HBA, our library matches the commercial libraries more closely than it matches the synthetic libraries. In contrast, our library more closely matches the values of the synthetic fragment libraries in terms of HBD, nRot, and TPSA. Overall, the calculated physicochemical properties partially echo the characteristics of the parent Cyrene skeleton. They indicate that the library of Cyrene-derived fragments mostly adheres to the Ro3 guidelines and that it displays properties similar to the commercial and synthetic 3D libraries. Compared to the Ro3, only the HBA count is violated, which is quite common in fragment libraries and does not necessarily present an obstacle as HBA atoms can facilitate meaningful interactions based on enthalpic factors. In contrast, the most noteworthy differences between the synthetic and commercial libraries are in ring count, number of stereocenters, and the relatively high percentage of oxygen atoms. Indeed, the biogenic offspring of the Cyrene library is distinctly visible in the increased numbers of (saturated) rings and stereocenters (Table 2), and in the relatively high percentage of oxygen atoms (Table 2), reflecting the glucose origin of Cyrene as well as the absence of nitrogen atoms in classes **5** and **6.** Not unexpectedly, for several of these parameters, our library bears considerable resemblance to natural product-derived fragments [20], even though Cyrene is not a known natural product. We note that only class **8** has nitrogen atoms that are predicted to be protonated at physiological pH.

All five compounds tested for solubility in buffer show no signs of precipitation/aggregation up to at least 0.32 mM, with three of these not aggregating up to at least 1.0 mM. In other words, meaningful pharmacological concentrations for fragments can be attained without the parent ketone of Cyrene present, in line with recent work on *iso*-Cyrene [45], and despite the presence of aromatic groups in the fragments. We speculate that this partially reflects the inherent properties of the Cyrene skeleton. Even screening at high concentrations in the mM range, e.g., using NMR spectroscopy, seems feasible. All fragments selected for incubation in DMSO and buffer showed no significant decomposition over a time span of three weeks under various conditions. This underscores the anticipated stability of the acetal functionality in the Cyrene skeleton, despite the absence of the ketone moiety, and strongly indicates the stability required for storage in plates and for biological assays.

The 3D shapes inferred from PMI analyses resemble mostly “rod-like” structures with, however, a significant three-dimensional character. The higher “rod-like” character compared to commercial and synthetic libraries can be rationalized by the rigidity of the Cyrene core and the notion that only one growth vector is addressed, in the current case by an aromatic group. The sulfonamide class, in particular, presents a strategy to get more diversity in 3D properties.

There are a few comments to be made about the potential performance of this library. By design, there is some diversity in the standard functional groups appended but the overall diversity of the library is limited as all members possess the Cyrene skeleton and only a single growth vector has been explored (Hughes et al. have probed the α-position, albeit not in an FBDD context [33]). This bias is a consequence of the conceptual goal of the current work and is illustrated clearly by the narrower distributions in Figure 4A and Figure 3C (gray), as well as by the shift toward the rod vertex in Figure 4B. We point out that the combined use of Cyrene and LGO, as well as biomass precursor levoglucosan [24], bodes well for exploring additional growth vectors at the central 7,8-dioxabicyclo[3.2.1]octane fragment scaffold. Moreover, basic nitrogen atoms, often key players in ionic interactions with a protein, are only modestly present in our series with classes **5–6** having no nitrogen atoms at all. Indeed, the oxygen count of the library is relatively high, partially reflecting the sugar origin of Cyrene. Additionally, the presence of three stereocenters leads to a relatively high complexity. We do not consider this a disadvantage per se, as it will be interesting to see how this can add to the ongoing explorations on the potential merits of 3D fragments. In all, a lower hit rate in screening this Cyrene library may be expected. However, a powerful feature of FBDD is that it is agnostic of the protein target, meaning that fragment libraries can in principle be screened against any of the hundreds of known protein drug targets, and those emerging in the future. At least equally importantly, any hit will by definition contain a scaffold based on “green” renewable resources. The described 100-membered library has been incorporated into our in-house fragment resources, either in primary screening plates or as part of a follow-up analog collection.

In conclusion, we have described a 100-membered fragment library that is based on a union of two crucial contemporary scientific topics, i.e., health and sustainability. Our work shows how biomass-derived precursors such as Cyrene^TM^ can provide sustainable entry into unexplored parts of chemical (fragment) space. Screening of the library on a variety of protein targets is foreseen. Parallel efforts in our laboratories will focus on the inclusion of less common functional groups (e.g., sulfoximine or groups linked through C–C bonds [66,67]), exploration of other vectors, incorporation of a custom-made automated workflow for optimal design, and exploration of biomass-derived precursors other than Cyrene (Figure 1).

## 4. Materials and Methods

### 4.1. Nephelometry

Nephelometry was performed using NEPHELOstar Plus equipment (BMG LABTECH, Ortenberg, Germany). Kaolin was used as the internal standard, and compounds were added to HBSS buffer as DMSO stock solution to a final concentration of 1% DMSO and a total volume of 200 μL. Precipitation or aggregation was considered significant when average values exceeded three times the standard deviation of the blanks. Blank values were omitted if they exceeded three times the standard deviation of the 24 blanks that were measured on each 96-well plate. All compounds were tested in triplo, and wells of suspected outliers were visually inspected before omitting any outliers. Data were processed in Prism version 8.0 (GraphPad, San Diego, CA, USA).

### 4.2. Incubation Experiments

LCMS studies: A 10 mM solution in DMSO was diluted with HBSS buffer to 0.10 mM. Fragment integrity was measured by LCMS at regular intervals (not shown) up to 20 d in the dark at rt, or after 20 d of freezing. NMR studies: A 10 mM solution in DMSO-d6 was prepared. Fragment integrity was measured with ^1^H NMR analysis at regular intervals (not shown) up to 20 d in the dark at rt, or after 20 d of freezing.

### 4.3. General Experimental

Unless mentioned otherwise, all reactions were performed under N_2_ atmosphere. Cyrene was a kind gift from Will&Co (Badhoevedorp, The Netherlands). Its specific optical rotation (CDCl_3_, 0.010 g/mL) was –236.2°, in accordance with literature values [68]. All other chemicals and solvents were obtained from commercial suppliers (primarily Sigma-Aldrich (St. Louis, MO, USA), Acros Organics (Geel, Belgium), Fluorochem (Hadfield, UK), and Combi-Blocks (San Diego, CA, USA)) and used without purification. DCM, DMF, and THF were dried by passing through a PureSolv solvent purification system (Innovative Technology, China). IUPAC names were adapted from ChemBioDraw Ultra 20.0 (PerkinElmer, Waltham, MA, USA), Collaborative Drug Discovery (CDD) software (Burlingame, CA, USA), or PubChem (Bethesda, MD, USA). Reactions were monitored using F_254_ aluminum-backed silica plates from Screening Devices (Amersfoort, The Netherlands) or Merck (Darmstadt, Germany) and visualized with 254 nm UV light or staining with KMnO_4_. Reverse-phase column chromatography was performed on CombiFlash Rf 200 equipment (Teledyne ISCO, Lincoln, NE, USA). Normal phase flash chromatography was performed on Biotage Isolera (Biotage, Uppsala, Sweden) or BUCHI Pure C-815 equipment (BUCHI, Flawil, Switzerland). Pre-packed columns were purchased from Screening Devices (Amersfoort, The Netherlands) (C_18_ and UltraPure irregular silica). Microwave reactions were carried out using a Biotage Initiator equipment (Biotage, Uppsala, Sweden). Nuclear magnetic resonance (NMR) spectra were determined with a Bruker Avance II 500 MHz or a Bruker Avance III HD 600 MHz spectrometer (Bruker, Billerica, MA, USA). Chemical shifts are reported in parts per million (ppm) against the reference compound using the signal of the residual non-deuterated solvent (CDCl_3_ δ = 7.26 ppm (1H), δ = 77.16 ppm (^13^C); DMSO-d6 δ = 2.50 ppm (^1^H), δ = 39.52 ppm (^13^C), CD_3_OD δ = 3.31 ppm (^1^H), δ = 49.00 ppm (^13^C)). NMR spectra were processed using MestReNova 14.1.1 software (Mestrelab, Santiago de Compostela, Spain). The peak multiplicities are defined as follows: s, singlet; d, doublet; t, triplet; q, quartet; dd, doublet of doublets; ddd, doublet of doublets of doublets; dt, doublet of triplets; dq, doublet of quartets; td, triplet of doublets; tt, triplet of triplets; qd, quartet of doublets; p, pentet; dp, doublet of pentets; br, broad signal; m, multiplet. For NMR listings, in addition to specific instructions that are given by the journal in the guidelines for authors the following additional procedures were used:

1. Multiplicity is not solely reported based on peak shapes, but also distinguishes the coupling to all non-equivalent protons that have similar *J* values;
2. If additional smaller couplings are observed or expected but are too small for accurate quantitation because the precision is smaller than the digital resolution, a symbol ^∆^ will be used;
3. The notation “m” is used in case of obscured accurate interpretation as a result of:
  - Overlapping signals for different protons, or;
  - A result of overlapping signal lines within the same proton signal;
4. For compounds that were isolated as mixtures of diastereomers with a d.r. < 9:1, signals were listed separately if possible. Signals were annotated with the corresponding diastereomer as follows: ^a^ signal(s) assigned to diastereomer 1; ^b^ signal(s) assigned to diastereomer 2; ^a/b^ signal(s) could not be assigned with certainty to either diastereomer 1 or 2; ^a,b^ signal(s) assigned to both diastereomer 1 and 2 (only applies to multiplets). Diastereomer 1 indicates the major diastereomer or an arbitrarily assigned diastereomer in the case of a d.r. of 1:1. The number of protons that cause a signal is corrected for the d.r. in the ^1^H NMR listings, i.e., in the example “3.77 (d, *J* = 6.1 Hz, 0.9H)^a^, 3.73 (d, *J* = 6.4 Hz, 0.1H)^b^”, the d.r. ≅ 9:1 and one proton of major diastereomer 1 gives a doublet at 3.77 ppm; the same proton in diastereomer 2 gives a doublet at 3.73 ppm. The corrected number of protons was summed in the case of overlapping signals. In the case of d.r. > 9:1 and/or in the case of extensive overlap of signals of both diastereomers, only the major signals were listed. For any fumaric acid salts, the CH protons of fumaric acid counterion (HOOC–C**H**=C**H**–COOH) were listed as “6.xx (m, 2H)^a,b^”, irrespective of the acid/base ratio;
5. NMR signals that could only be detected with HSQC analysis are denoted with a ^#^ symbol;
6. NMR signals that could only be detected with HMBC analysis are denoted with a * symbol;
7. If one or more signals remain undetected after extensive 1D and 2D NMR analyses, this will be mentioned;
8. Signals for exchangeable proton atoms (such as NH and OH groups) are only listed if clearly visible (excluding e.g., the use of D_2_O or CD_3_OD) and if confirmed by a D_2_O shake and/or HSQC.

Purity determination was performed with liquid chromatography using a Shimadzu LC-20AD liquid chromatography pump system with a Shimadzu SPDM20A photodiode array detector and MS detection with a Shimadzu LCMS-2010EV mass spectrometer operating in both positive and negative ionization mode (Shimadzu, Kyoto, Japan). A Waters XBridge C18 column (5 μm, 4.6 × 50 mm) was used at 40 °C (Waters, Milford, MA, USA). For acidic runs, 0.1% HCOOH in H2O and 0.1% HCOOH in MeCN were used as eluent A and B, respectively. For basic runs, 0.4% *w*/*v* NH_4_HCO_3_ in H2O and MeCN were used as eluent A and B, respectively. The gradient for acidic runs was 5:90:90:5:5% B at t = 0:4.5:6:6.5:8 min. Compound purities were calculated as the percentage peak area of the analyzed compound by UV detection at 254 nm or 230 nm (for compounds with low epsilon value at 254 nm). High-resolution mass spectra (HRMS) were recorded on a Bruker micrOTOF mass spectrometer (Bruker, Billerica, MA, USA) using ESI in positive ion mode. Compounds have an LC purity of ≥95% unless specified otherwise, calculated as the percentage peak area of the analyzed compound by UV detection at 254 or 230 nm (values are rounded). Purities refer to combined peak areas of diastereomers if both isomers are present. ^1^H NMR analysis showed that some compounds had traces of grease, but this was deemed non-interfering for early fragment screening. Details on the synthetic procedures are disclosed in the Supplementary Materials.

### 4.4. Computational Methods and Figures

Molecular conformations, physicochemical properties, and 3D metrics were calculated in KNIME v4.3.1 software (KNIME, Zurich, Switzerland) using RDKit (4.0.1.v202006261025), Erlwood (v4.0.0) and Vernalis (1.28.2.v202101281353) nodes. Data was processed into figures using RStudio v2022.02.2 software (Posit, Boston, MA, USA) with the ggplot package installed. Illustrations were made in Illustrator 2021 (Adobe, San Jose, CA, USA).

## Figures and Tables

**Table 1:** All 100 compounds prepared in this study. Three templates (I, II, III) are used for clarity. Unless mentioned otherwise, compounds are a mixture of *endo* and *exo* diastereomers. Supplementary Materials Tables S2 and S3 show all full structures, d.r. values and, where appropriate, proposed relative stereochemistry of the (major) diastereomer.

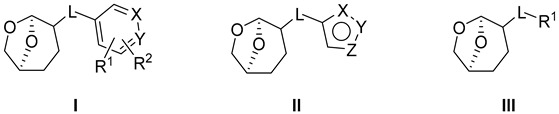								
	Compound	Template	L	X	Y	Z	R^1^	R^2^
Ethers	**5a**	I	–OCH_2_–	CH	CH	-	-	-
	**5b**	I	–OCH_2_–	CH	CH	-	2-Me	-
	**5c**	I	–OCH_2_–	CH	CH	-	4-Me	-
	**5d**	I	–OCH_2_–	CH	CH	-	3-CN	-
	**5e**	I	–OCH_2_–	CH	CH	-	4-CN	-
	**5f**	I	–OCH_2_–	CH	CH	-	3-OMe	-
	**5g**	I	–OCH_2_–	CH	CH	-	4-COOMe	-
	**5h**	I	–OCH_2_–	N	CH	-	-	-
	**5i**	I	–OCH_2_–	CH	N	-	-	-
Esters	**6a**	I	–O(CO)–	CH	CH	-	-	-
	**6b**	I	–O(CO)–	CH	CH	-	2-F	-
	**6c**	I	–O(CO)–	CH	CH	-	4-Cl	-
	**6d**	I	–O(CO)–	CH	CH	-	3-CN	-
	**6e**	I	–O(CO)–	CH	CH	-	2-OMe	-
	**6f**	I	–O(CO)–	CH	CH	-	4-NMe_2_	-
	**6g**	II	–O(CO)–	CH	O	CH	-	-
	**6h**	II	–O(CO)–	O	CH	CH	-	-
	**6i**	II	–O(CO)–	S	CH	CH	-	-
	**6j**	II	–O(CO)–	NMe	CH	CH	-	-
	**6k**	II	–O(CO)–	O	CH	N	-	-
	**6l**	II	–O(CO)–	N	O	CH	-	-
	**6m**	II	–O(CO)–	N	CH	S	-	-
	**6n**	II	–O(CO)–	NH	N	CH	-	-
	**6o**	II	–O(CO)–	NMe	N	CH	-	-
	**6p**	I	–O(CO)–	CH	N	-	-	-
	**6q**	I	–O(CO)–	N	CH	-	3-F	-
	**6r**	III	–O(CO)–	-	-	-	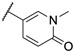	-
*O*-Carbamates	**7a** ^b^	I	–O(CO)NH–	CH	CH	-	2-Me	3-Me
	**7b** ^b^	I	–O(CO)NH–	CH	CH	-	4-F	-
	**7c**	I	–O(CO)NH–	CH	CH	-	4-Cl	-
	**7d**	I	–O(CO)NHCH_2_–	CH	CH	-	3-F	-
Amines	**8a**	I	–NHCH_2_–	CH	CH	-	4-Br	-
	**8b**	III	–NHCH_2_–	-	-	-	*c*Pr	-
	**8c**	I	–NHCH_2_–	CH	CH	-	3-Cl	4-Cl
	**8d**	I	–NHCH_2_–	CH	CH	-	2-OMe	4-OMe
	**8e**	I	–NHCH_2_–	CH	CH	-	3,4-methylenedioxy	
	**8f** ^a,b^	II	–NHCH_2_–	N	NMe	CH	-	-
	**8g**	I	–NHCH_2_–	CH	CH	-	4-OMe	-
	**8h** ^b^	I	–NHCH_2_–	CH	CH	-	2-Cl	6-Cl
	**8i** ^c^	I	–NHCH_2_–	CH	CH	-	2-Cl	6-Cl
	**8j**	I	–NHCH_2_–	N	CH	-	-	-
	**8k**	I	–NHCH_2_–	CH	N	-	-	-
	**8l**	I	–NHCH_2_–	CH	CH	-	4-Cl	-
	**8m**	I	–NHCH_2_–	CH	CH	-	3-F	-
	**8n** ^a^	I	–NHCH_2_–	CH	CH	-	2-Me	-
	**8o**	I	–NHCH_2_–	CH	CH	-	4-Me	-
	**8p** ^a^	I	–NHCH_2_–	CH	CH	-	4-SO_2_Me	-
	**8q** ^b^	I	–NHCH_2_–	CH	CH	-	4-CN	-
	**8r** ^c^	I	–NHCH_2_–	CH	CH	-	4-CN	-
	**8s** ^a^	I	–NHCH_2_–	CH	CH	-	-	-
Amides	**9a** ^b^	I	–NH(CO)–	CH	CH	-	-	-
	**9b** ^c^	I	–NH(CO)–	CH	CH	-	-	-
	**9c**	III	–NH(CO)–	-	-	-	Et	-
	**9d**	III	–NH(CO)–	-	-	-	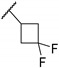	-
	**9e**	I	–NH(CO)–	CH	CH	-	2-OMe	-
	**9f** ^b^	I	–NH(CO)–	CH	CH	-	3-OMe	-
	**9g** ^c^	I	–NH(CO)–	CH	CH	-	3-OMe	-
	**9h** ^b^	I	–NH(CO)–	CH	CH	-	4-OMe	-
	**9i** ^c^	I	–NH(CO)–	CH	CH	-	4-OMe	-
	**9j**	II	–NH(CO)–	NH	CH	CH	-	-
	**9k**	III	–NH(CO)–	-	-	-	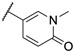	-
	**9l**	I	–NH(CO)CH_2_–	CH	N	-	-	-
	**9m**	I	–NH(CO)(CH_2_)_2_–	CH	CH	-	-	-
	**9n** ^b^	I	–NH(CO)–	CH	CH	-	3-NMe_2_	-
	**9o** ^b^	II	–NH(CO)–	S	CH	CH	-	-
	**9p**	II	–NH(CO)–	S	CH	CH	-	-
	**9q**	I	–NH(CO)–	CH	N	-	-	-
	**9r**	I	–NH(CO)–	CH	CH	-	3-F	-
	**9s**	I	–NH(CO)–	CH	CH	-	4-Cl	-
	**9t**	I	–NH(CO)–	CH	CH	-	3-CN	-
	**9u**	I	–NH(CO)–	CH	CH	-	4-CN	-
	**9v**	I	–NH(CO)–	CH	CH	-	2-F	-
	**9w**	I	–NH(CO)–	CH	CH	-	3-Cl	-
	**9x**	II	–NH(CO)–	O	CH	N	-	-
*N*-Carbamates	**10a**	I	–NH(CO)OCH_2_–	CH	CH	-	3-F	-
	**10b**	I	–NH(CO)O–	CH	CH	-	3-OMe	-
	**10c**	I	–NH(CO)O–	CH	CH	-	3-F	-
Ureas	**11a**	I	–NH(CO)NH–	CH	CH	-	4-F	-
	**11b**	I	–NH(CO)NH–	CH	CH	-	-	-
	**11c**	I	–NH(CO)NH–	CH	CH	-	2-F	4-F
	**11d**	I	–NH(CO)NH–	CH	CH	-	2-Me	3-Me
	**11e**	I	–NH(CO)NH–	CH	CH	-	4-OMe	-
	**11f**	I	–NH(CO)NH–	CH	CH	-	3-Me	4-Me
	**11g**	I	–NH(CO)NHCH_2_–	CH	CH	-	3-Me	-
	**11h**	I	–NH(CO)NH–	CH	CH	-	3-Me	-
	**11i**	III	–NH(CO)NEt_2_	-	-	-	-	-
	**11j**	I	–NH(CO)NHCH_2_–	CH	CH	-	4-Cl	-
	**11k**	I	–NH(CO)NH–	CH	CH	-	3-OMe	-
	**11l**	I	–NH(CO)NH–	CH	CH	-	2-OMe	-
	**11m**	I	–NH(CO)NHCH_2_–	CH	CH	-	3-F	-
	**11n**	I	–NH(CO)NH–	CH	CH	-	3-F	-
	**11o**	I	–NH(CO)NH–	CH	CH	-	2-F	-
	**11p**	I	–NH(CO)NHCH_2_–	CH	CH	-	2-F	-
Sulfonamides	**12a** ^b^	I	–NHSO_2_–	CH	CH	-	-	-
	**12b** ^c^	I	–NHSO_2_–	CH	CH	-	-	-
	**12c** ^b^	I	–NHSO_2_–	CH	CH	-	4-Me	-
	**12d** ^c^	I	–NHSO_2_–	CH	CH	-	4-Me	-
	**12e**	I	–NHSO_2_–	CH	CH	-	3-F	-
	**12f**	III	–NHSO_2_–	-	-	-	*c*Pr	-
	**12g**	III	–NHSO_2_–	-	-	-	*n*Pr	-

^a^ Isolated as the fumarate salt; ^b^ Diastereomer 1; ^c^ Diastereomer 2.

**Table 2:** Calculated properties. Average mean values for the properties of commercial “Fsp^3^” and “3D” fragment libraries extracted from a recent review [39], 25 papers disclosing synthetic 3D fragment sets [40], and the 100 compounds of the current paper. (Top) The “rule of three” physicochemical parameters supplemented with HAC; (Bottom) Additional structural parameters. Values were calculated with KNIME software on the neutral chemical species.

Property	Ro3 Limits	Fsp^3^/3D Commercial Libraries	3D Synthetic Libraries	Current Work
cLogP ^a^	3	1.56	1.13	1.52
HBA	3	3.77	3.23	3.92
HBD	3	1.18	1.08	0.90
nRot	3	4.5	2.0	2.6
TPSA	60	58.1	53.5	53.0
MW	300	262	232	254
HAC	20	18.3	16.2	17.9
#Aromatic rings	-	0.88	0.65	0.94
#Rings	-	2.01	2.28	2.98
#Saturated rings	-	1.07	1.32	2.03
#Stereocenters	-	1.07	1.67	3.00
% Carbon atoms ^b^	-	71.6	71.0	71.8
% heteroatoms ^b^	-	28.4	29.0	28.2
% Nitrogen atoms ^b^	-	13.8	10.2	6.7
% Oxygen atoms ^b^	-	11.4	14.7	19.0

^a^ cLogP was calculated as SLogP. ^b^ Percentage versus total number of heavy atoms.

## Data Availability

Not applicable.

## Supplementary Materials

The following supporting information can be downloaded at: https://www.mdpi.com/article/10.3390/molecules28041777/s1, Figure S1: Details for the incubation experiments. Section S1–2: Detailed synthetic procedures for the precursors and the 100 final fragments, respectively. Section S3: Selected analytical data (^1^H and ^13^C spectra). Table S1: Results of a literature search using the Reaxys search engine. Table S2: Expanded version of Table 1 containing all 100 fragments synthesized. Excel Table S3: Full structures, proposed stereochemistry, and details for the stereochemistry assignments.
