# Supplementary material for: Green Drug Discovery: Novel Fragment Space from the Biomass-Derived Molecule Dihydrolevoglucosenone (CyreneTM)
Source: Molecules. 2023 Feb 13;28(4):1777. doi: 10.3390/molecules28041777 (PMC9967789; doi:10.3390/molecules28041777)
Supplement: Supplementary file 1 [file molecules-28-01777-s001.zip › Supporting Information.pdf]

Green drug discovery: novel fragment space from the biomass-derived molecule dihydrolevoglucosenone (Cyrene^{TM})

*Tom Dekker, Jaap W. Harteveld, Gábor Wágner, Max C.M. de Vries, Hans Custers, Andrea C. van de Stolpe, Iwan J. P. de Esch, Maikel Wijtmans**

Author information
Amsterdam Institute of Molecular and Life Sciences (AIMMS), Vrije Universiteit Amsterdam, De Boelelaan 1108, 1081 HZ Amsterdam (The Netherlands)
*E-mail: m.wijtmans@vu.nl

Table of contents

Table S1: Results literature search	Page S2
Table S2: All 100 fragments synthesized	Page S3-S6
Figure S1: Incubation experiments	Page S7-S10
Section S1: Syntheses of the required precursors	Page S11-S12
Section S2: Syntheses of the final fragments	Page S13-S67
Section S3: Selected analytical data	Page S68-S81
References	Page S82

Table S1. Results of a literature search using the Reaxys search engine (June 6, 2022). Any R group was set to a carbon atom with maximal substituent count ("s6"). Any Z-bound heteroatom was set to maximal substituent count ("s6"). The number of hits refers to the number of discrete compounds reported in the literature (stereoisomers and any isotope labeled analogues were counted as one). Compounds only claimed in the Pubchem database but not in the primary literature were not included.

**Functional group**	**Class code**	**Search entry for Reaxys**	**Number of hits**
Ethers	**5**	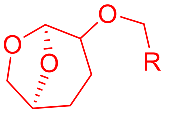	2<br>(vinyl, phenyl)
Esters	**6**	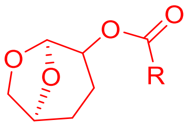	3<br>(methyl, dinitrophenyl, 2-allyl)
O-Carbamates	**7**	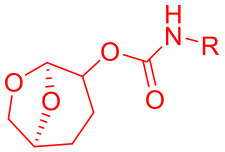	0
**Oxygen substitution total**		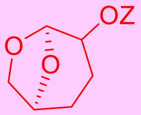	10<br>(alcohol, tosylate, mesylate, acetate, dinitrobenzoate, methacrylate, benzyl ether, allyl ether, CH_2SMe, CH_2OAc)
Amines	**8**	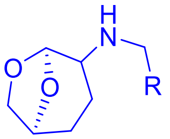	0
Amides	**9**	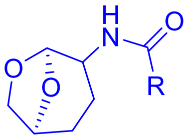	0
N-Carbamates	**10**	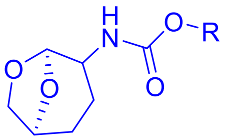	0
Ureas	**11**	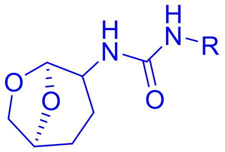	0
Sulfonamides	**12**	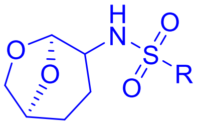	0
**Nitrogen substitution total**		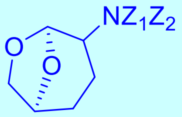	2<br>(amine, azide)

Table S2. All 100 fragments prepared in this study. This table is an expanded version of Table 1 in the main text. N-based compounds are drawn in blue, while O-based compounds are drawn in red. Unless mentioned otherwise, compounds are a mixture of *endo* and *exo* diastereomers. VUF codes represent our in-house coding. The Excel file Table S3 uploaded as Supporting Information contains more details on the stereochemistry.

	Ethers			Amines			Amides (continued)	
	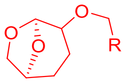			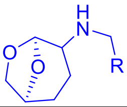			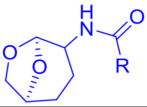	
**5a**	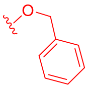	VUF-0025638	**8a**	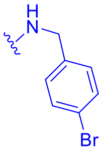	VUF-0025899	**9s**	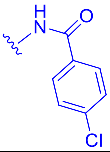	VUF-0025898
**5b**	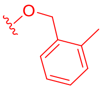	VUF-0025620	**8b**	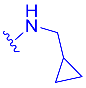	VUF-0025750	**9t**	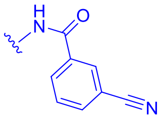	VUF-0025983
**5c**	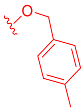	VUF-0025654	**8c**	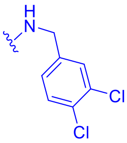	VUF-0025890	**9u**	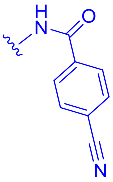	VUF-0025991
**5d**	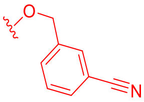	VUF-0025641	**8d**	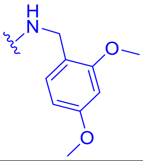	VUF-0025891	**9v**	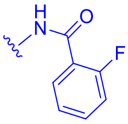	VUF-0025998
**5e**	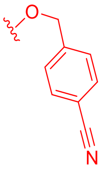	VUF-0025651	**8e**	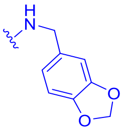	VUF-0025892	**9w**	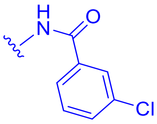	VUF-0026002
**5f**	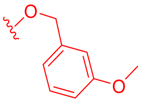	VUF-0025647	**8f** [a]	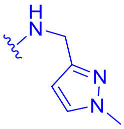 Diastereomer 1	VUF-0025759 fumarate	**9x**	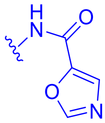	VUF-0025888
**5g**	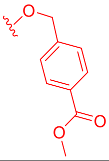	VUF-0025627	**8g**	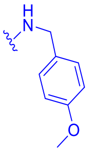	VUF-0025862		***N*-Carbamates**	
**5h**	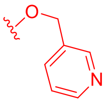	VUF-0025649	**8h**	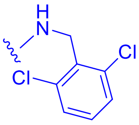 Diastereomer 1	VUF-0025863		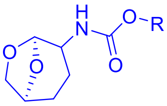	
**5i**	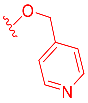	VUF-0025653	**8i**	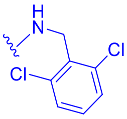 Diastereomer 2	VUF-0025864	**10a**	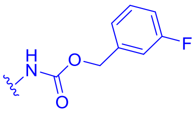	VUF-0025973

	Esters	
	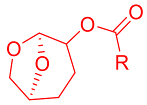	
**6a**	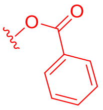	VUF-0025608
**6b**	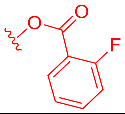	VUF-0025618
**6c**	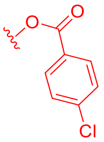	VUF-0025601
**6d**	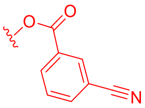	VUF-0025599
**6e**	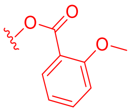	VUF-0025637
**6f**	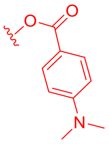	VUF-0025645
**6g**	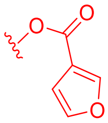	VUF-0025607
**6h**	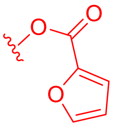	VUF-0025643
**6i**	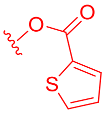	VUF-0025622
**6j**	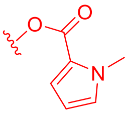	VUF-0025600

**8j**	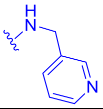	VUF-0025870
**8k**	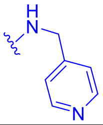	VUF-0025871
**8l**	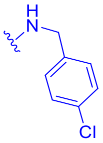	VUF-0025876
**8m**	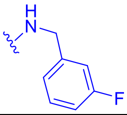	VUF-0025883
**8n** [a]	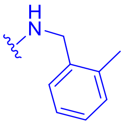	VUF-0025761 fumarate
**8o**	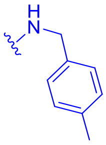	VUF-0025762
**8p** [a]	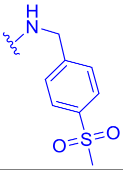	VUF-0025758 fumarate
**8q**	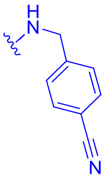 Diastereomer 1	VUF-0025896
**8r**	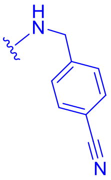 Diastereomer 2	VUF-0025897
**8s** [a]	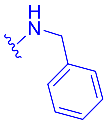	VUF-0025760 fumarate
	**Amides**	
	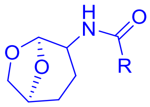	

**10b**	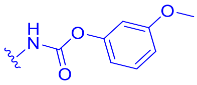	VUF-0026003
**10c**	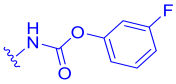	VUF-0026004
	**Ureas**	
	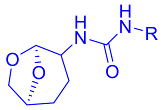	
**11a**	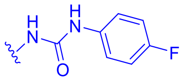	VUF-0025861
**11b**	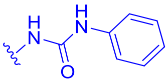	VUF-0025869
**11c**	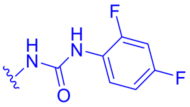	VUF-0025875
**11d**	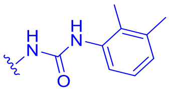	VUF-0025879
**11e**	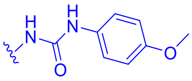	VUF-0025889
**11f**	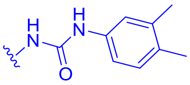	VUF-0025900
**11g**	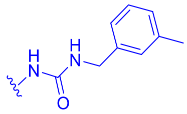	VUF-0025980
**11h**	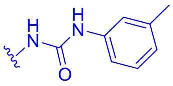	VUF-0025981

**6k**	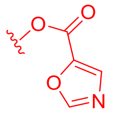	VUF-0025642	**9a**	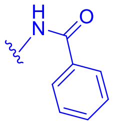 Diastereomer 1	VUF-0025752	**11i**	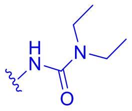	VUF-0025982
**6l**	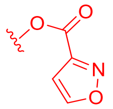	VUF-0025652	**9b**	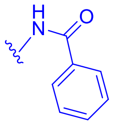 Diastereomer 2	VUF-0025753	**11j**	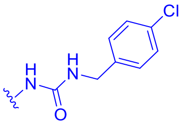	VUF-0025984
**6m**	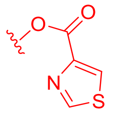	VUF-0025636	**9c**	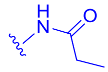	VUF-0025746	**11k**	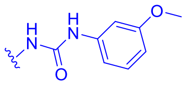	VUF-0025987
**6n**	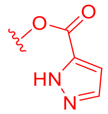	VUF-0025629	**9d**	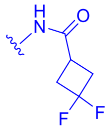	VUF-0025755	**11l**	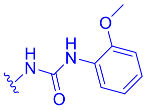	VUF-0025993
**6o**	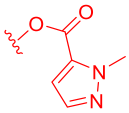	VUF-0025640	**9e**	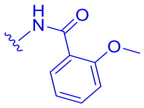	VUF-0025754	**11m**	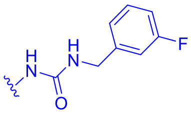	VUF-0025996
**6p**	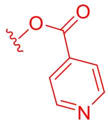	VUF-0025619	**9f**	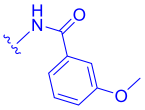 Diastereomer 1	VUF-0025742	**11n**	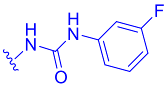	VUF-0025997
**6q**	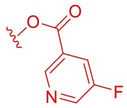	VUF-0025603	**9g**	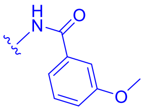 Diastereomer 2	VUF-0025743	**11o**	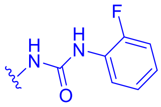	VUF-0025999
**6r**	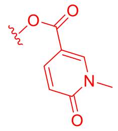	VUF-0025644	**9h**	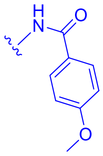 Diastereomer 1	VUF-0025744	**11p**	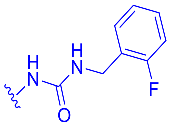	VUF-0026000
	***O*-Carbamates**		**9i**	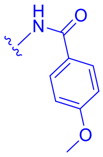 Diastereomer 2	VUF-0025745		**Sulfonamides**	
	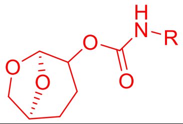		**9j**	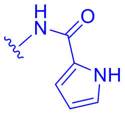	VUF-0025748		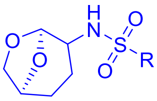	
**7a**	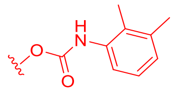 Diastereomer 1	VUF-0025866	**9k**	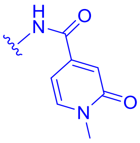	VUF-0025756	**12a**	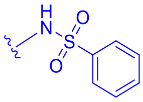 Diastereomer 1	VUF-0025859
**7b**	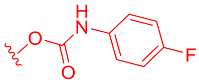 Diastereomer 1	VUF-0025895	**9l**	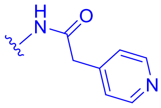	VUF-0025757	**12b**	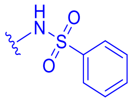 Diastereomer 2	VUF-0025860

**7c**	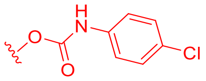	VUF-0025985	**9m**	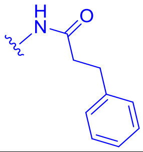	VUF-0025747	**12c**	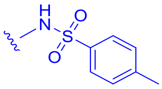 Diastereomer 1	VUF-0025867
**7d**	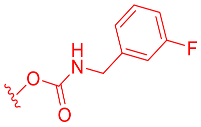	VUF-0026005	**9n**	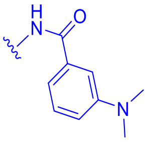 Diastereomer 1	VUF-0025865	**12d**	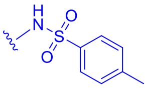 Diastereomer 2	VUF-0025868
			**9o**	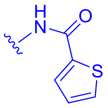 Diastereomer 1	VUF-0025872	**12e**	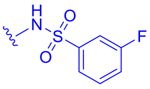	VUF-0025894
			**9p**	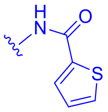	VUF-0025873	**12f**	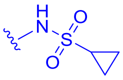	VUF-0025988
			**9q**	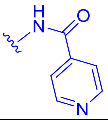	VUF-0025878	**12g**	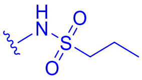	VUF-0025994
			**9r**	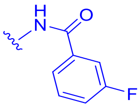	VUF-0025885			

[a] Fumarate salt.

Incubation experiments

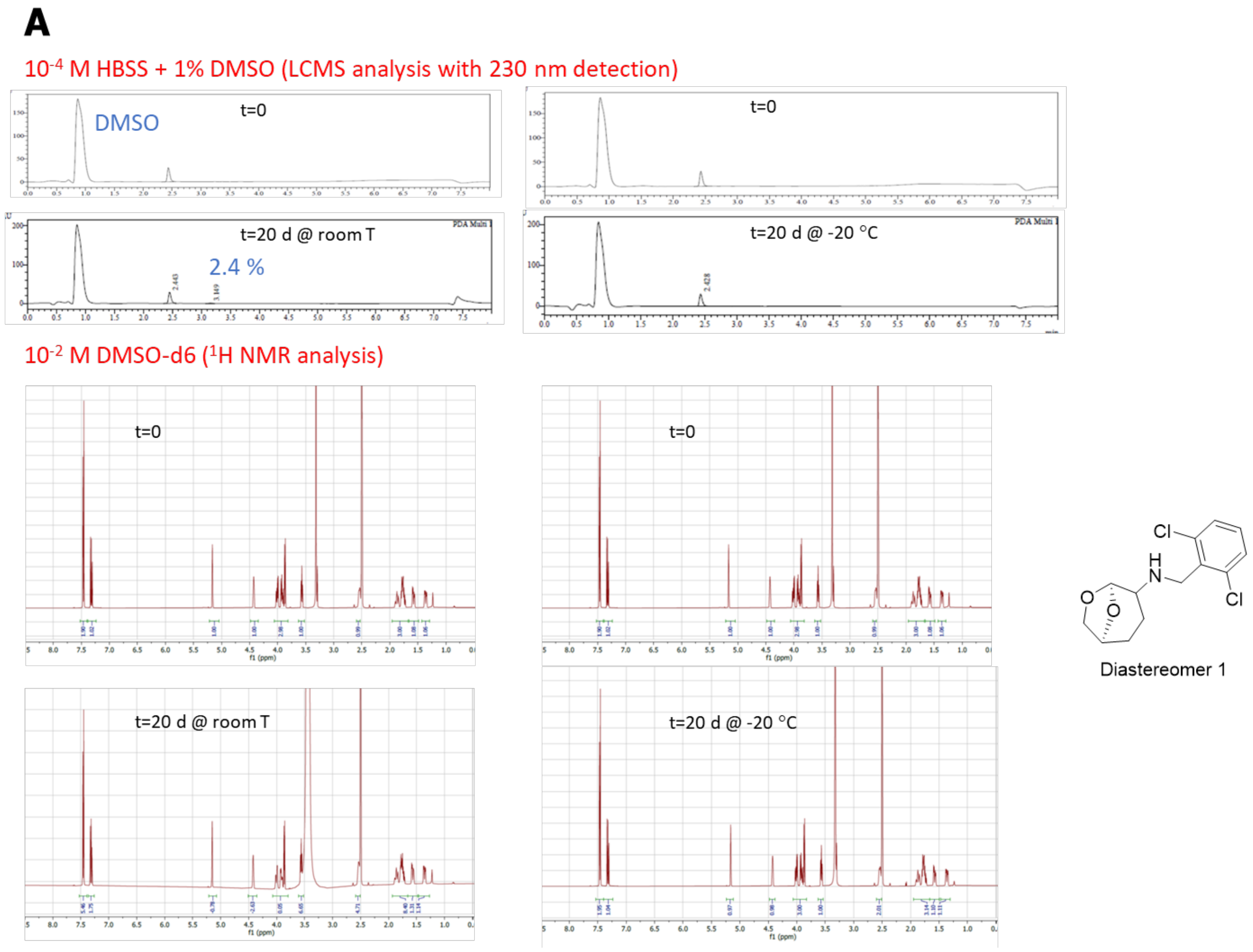

B

10^{-4} M HBSS + 1% DMSO (LCMS analysis with 230 nm detection)

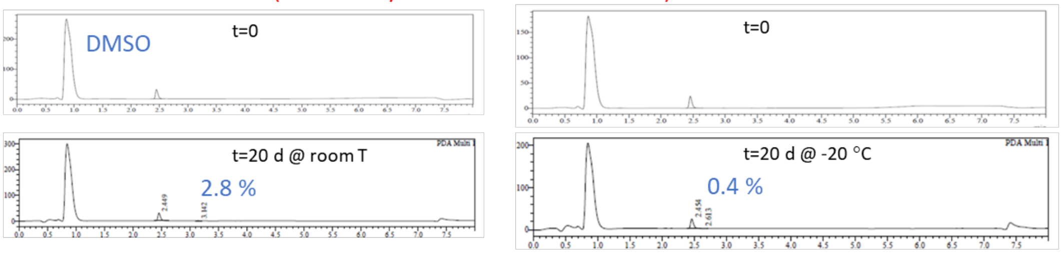

10^{-2} M DMSO-d6 (^{1}H NMR analysis)

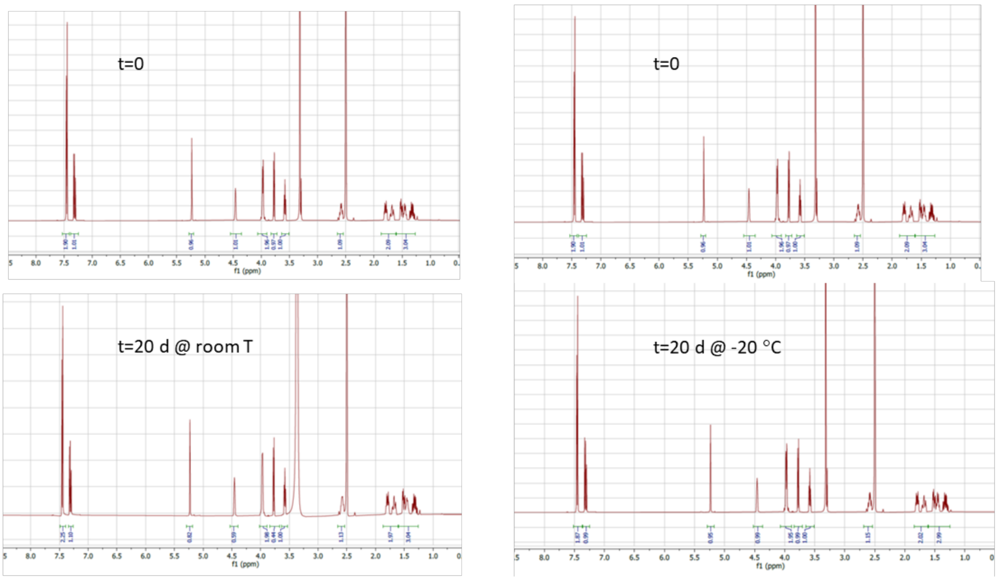

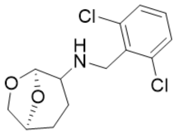

Diastereomer 2

c

10^{-4} M HBSS + 1% DMSO (LCMS analysis with 254 nm detection)

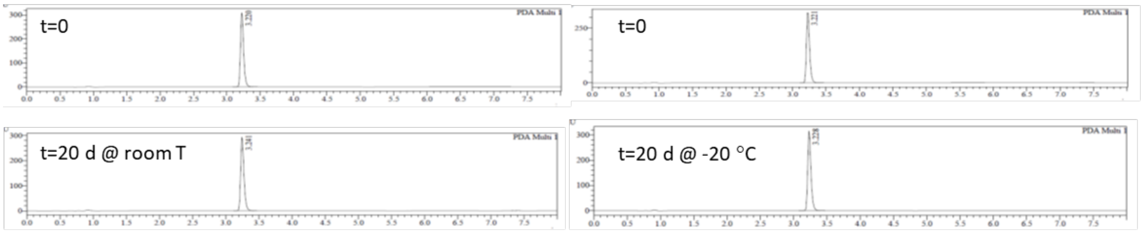

10^{-2} M DMSO-d6 (^{1}H NMR analysis)

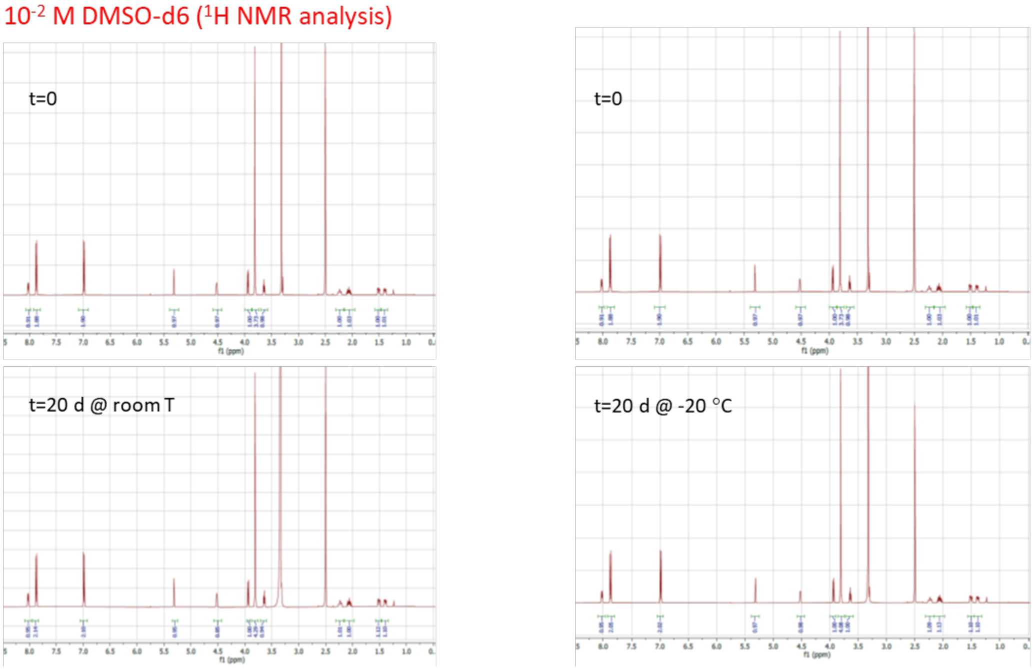

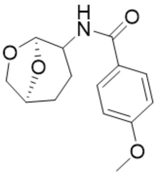

Diastereomer 1

D

10^{-4} M HBSS + 1% DMSO (LCMS analysis with 254 nm detection)

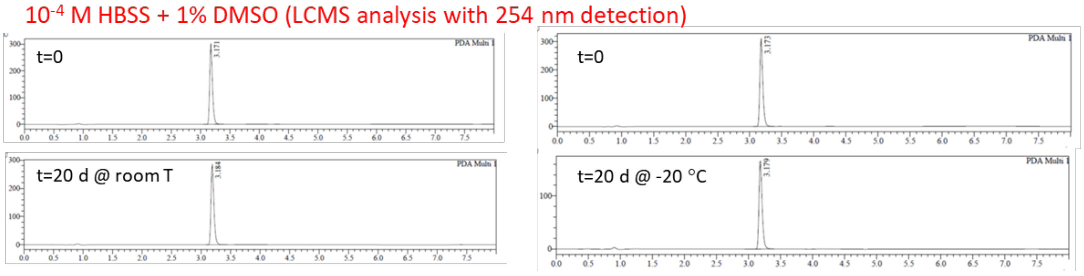

10^{-2} M DMSO-d6 (^{1}H NMR analysis)

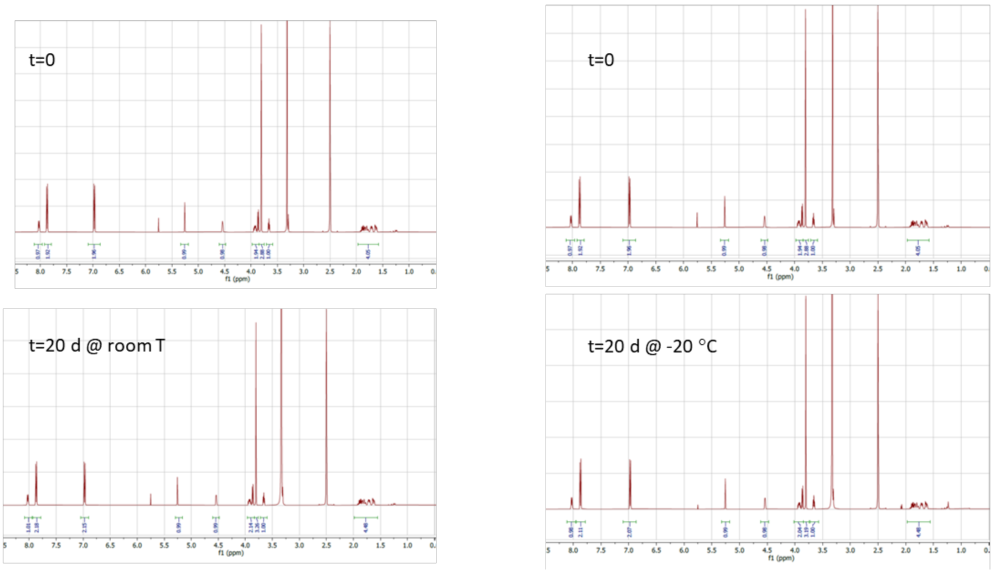

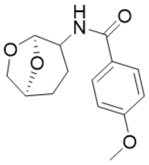

Diastereomer 2

Figure S1. Incubation experiments for (A) **8h**, (B) **8i**, (C) **9h** and (D) **9i**. (*Top panels*) A 10 mM solution in DMSO was diluted with HBSS buffer to 0.10 mM. Fragment integrity was measured by LC-MS at regular intervals (not shown) up to 20 d in the dark at rt (left), or after 20 d of freezing (right). (*Bottom panels*) A 10 mM solution in DMSO-d6 was prepared. Fragment integrity was measured with ^{1}H NMR analysis at regular intervals (not shown) up to 20 d in the dark at rt (left), or after 20 d of freezing (right).

Section S1: Syntheses of the required precursors

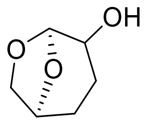

(1S,5R)-6,8-dioxabicyclo[3.2.1]octan-4-ol (2). This compound was prepared as described[1]. Cyrene (23.7 g, 0.185 mol) was dissolved in MeOH (450 mL) at 0 °C. Crushed $NaBH_4$ tablets (10.0 g, 0.264 mol, 1.42 eq) were added (caution: H_2 gas liberation). The mixture was stirred for 2 h while being allowed to warm to room temperature. The mixture was quenched with 8 M aq. HCl (33 mL, 0.264 mol, 1.42 eq) in MeOH (50 mL). The resulting white suspension was filtered and the solvent was removed *in vacuo*. The residue was suspended in acetone (150 mL). The solid was filtered and the solvents were removed from the filtrate *in vacuo*. This yielded the product as a yellow oil (23.9 g, 0.184 mol, 99%). Based on 1D NOE NMR analysis as well as on a comparison to reported data for the *endo* isomer[2], and for a ca. 1/1 mixture of *endo/exo* isomer[3], the product is obtained as a ~9/1 ratio of *endo/exo* isomers. **^{1}H NMR** (600 MHz, $CDCl_3$) δ 5.29 (s^{Δ}, 1H), 4.52 – 4.43 (m, 1H), 3.83 (d^{Δ}, *J* = 7.1 Hz, 1H), 3.81 – 3.75 (m, 1H), 3.61 – 3.52 (m, 1H), 2.21 (s, 1H), 2.04 – 1.97 (m, 1H), 1.91 – 1.82 (m, 1H), 1.61 – 1.53 (m, 1H), 1.53 – 1.45 (m, 1H). **^{13}C NMR** (151 MHz, $CDCl_3$) δ 103.0, 73.4, 72.9, 69.1, 68.3, 27.9, 26.1.

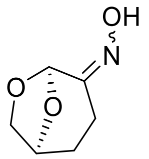

(1S,5R)-6,8-dioxabicyclo[3.2.1]octan-4-one oxime (3). This compound was prepared according to a reported procedure[4]. A solution of Cyrene (9.6 mL, 93.7 mmol) in pyridine (240 mL) was stirred and $H_2NOH.HCl$ (14.86 g, 214.9 mmol) was added. The reaction mixture was left to stir for 30 min at room temperature. EtOAc (250 mL) was added and the mixture was washed with water. The organic layer was dried, filtered and evaporated resulting in an oil. The remaining pyridine was co-evaporated with 50/50 *c*Hex/EtOAc. This afforded the product (12.83 g, 96 %) as opaque colored crystals. ^{1}H-NMR data was in accordance with the literature and suggests only one isomer to be present[4]. **^{1}H NMR** (500 MHz, $CDCl_3$) δ 5.53 (s^{Δ}, 1H), 4.70 – 4.62 (m, 1H), 3.93 (dd, *J* = 7.3, 0.9 Hz, 1H), 3.88 (ddd, *J* = 7.1, 5.3, 1.6 Hz, 1H), 3.07 (ddddd, *J* = 16.9, 7.3, 1.2, 1.2, 1.1 Hz, 1H), 2.25 (ddd^{Δ}, *J* = 16.9, 11.6, 8.2 Hz, 1H), 2.12 – 2.02 (m, 1H), 1.81 – 1.73 (m, 1H).

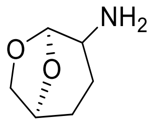

(1S,5R)-6,8-dioxabicyclo[3.2.1]octan-4-amine (4). This reaction was carried out based on reported general conditions[5]. Oxime **3** (12.83 g, 89.62 mmol) was dissolved in 250 mL of a solution of NH_3 in MeOH (1 N, 15 mL). A Raney-Nickel slurry (50 % in H_2O, 5.78 g) was filtered and the residue washed with MeOH 3 times. The residue was added to the solution of oxime. A H_2 balloon was applied to the flask. After 16 hours of stirring at room temperature, the reaction mixture was filtered over Celite and the solvent was evaporated. This provided the product as a light-colored oil (11.43 g, 99 %). Based on a comparison with reported data for the *exo* isomer, it is suggested that the product is obtained as a ~6/4 ratio of *endo/exo* isomers[6]. **^{1}H NMR** (500 MHz, $CDCl_3$) δ 5.26 (s^{Δ}, 0.4H)[b], 5.20 (s^{Δ}, 0.6H)[a], 4.54 – 4.44 (m, 1H)[a,b], 3.91 (d^{Δ}, *J* = 7.1 Hz, 0.4H)[b], 3.81 (d^{Δ}, *J* = 7.0 Hz, 0.6H)[a], 3.79 – 3.75 (m, 1H)[a,b], 2.83 – 2.79 (m, 0.4H)[b], 2.76 (dd^{Δ}, *J* = 11.1, 5.0 Hz, 0.6H)[a], 2.11 – 2.01 (m, 1H)[a,b], 1.93 – 1.82 (m, 1H)[a,b], 1.54 (m, 0.6H)[a], 1.47 – 1.42

(m, 0.4H)[b], 1.42 – 1.31 (m, 1H)[a,b]. **^{13}C NMR** (126 MHz, $CDCl_3$) δ 105.0[a], 104.1[b], 73.6[b], 72.8[a], 68.2[a], 67.0[b], 51.4[a], 49.5[b], 28.2[a], 26.4[b], 24.9[a], 24.2[b]. The compound has previously been reported as various *exo*-salts [7].

Section S2: Syntheses of the final fragments

General procedure A1 – Ethers

Alcohol **2** was dissolved in DMF and the mixture was cooled with an ice bath. NaH (60% in mineral oil) was added. Once liberation of H_2 gas (safety warning!) was no longer observed, TBAI and the indicated alkyl halide were added. The ice bath was removed and the reaction mixture was stirred overnight at rt. The mixture was diluted with H_2O and extracted with Et_2O as indicated. The combined organic layers were washed as indicated (if applicable), dried over Na_2SO_4, filtered and concentrated *in vacuo*. Subsequent purification by column chromatography provided the desired ether.

General procedure A2 – Ethers

Alcohol **2** was dissolved in DMF and the mixture was cooled with an ice bath. NaH (60% in mineral oil) was added. Once liberation of H_2 gas (safety warning!) was no longer observed, TBAI was added. In a separate vessel, the HCl or HBr salt of the indicated alkyl halide was dissolved in DMF and an equimolar amount of NaH (60% in mineral oil) was added. Once liberation of H_2 gas (safety warning!) was no longer observed, the resulting suspension was added dropwise to the reaction mixture. The ice bath was removed and the reaction mixture was stirred overnight at rt. The mixture was diluted with H_2O/satd. aq. $NaHCO_3$ (1:1) and extracted with Et_2O (10×). The combined organic layers were dried over Na_2SO_4, filtered and concentrated *in vacuo*. Subsequent purification by column chromatography (20% EtOAc in cHex + 1% Et_3N) provided the desired ether.

General procedure B – Esters

The indicated carboxylic acid, EDCI·HCl, DMAP and DIPEA were added to a solution of alcohol **2** in DCM. The reaction mixture was stirred overnight at rt. The mixture was diluted with the indicated aqueous solution and extracted with DCM. The combined organic layers were washed as indicated, dried over Na_2SO_4, filtered, and concentrated *in vacuo*. The residue was subjected to column chromatography to provide the desired ester.

General procedure C1a – Amines; direct reductive amination of Cyrene

The indicated amine and AcOH were added to a stirring solution of Cyrene (**1**) in DCM. The reaction mixture was stirred at rt for the indicated time and $NaBH(OAc)_3$ was added. The reaction mixture was stirred at rt for the indicated time. The reaction mixture was quenched with 1.0 M aq. NaOH and extracted as indicated. The combined organic layers were washed as indicated, dried over Na_2SO_4 or $MgSO_4$, filtered, concentrated *in vacuo*, and subjected to column chromatography to provide the desired amine.

General procedure C2a – Amines; indirect reductive amination of Cyrene

The indicated amine and $MgSO_4$ were added to a stirring solution of Cyrene (**1**) in DCM (2.5 mL). The reaction mixture was stirred at rt overnight, filtered and concentrated *in vacuo*. The residue was dissolved in MeOH (2.5 mL) and $NaBH_4$ was added. The reaction mixture was stirred at rt for 2 h, concentrated *in vacuo* and subjected to column chromatography. Where applicable, the fumarate salt was prepared by dissolving the purified amine and fumaric acid (1.0 eq) in MeOH, concentrating of the resulting solution, and recrystallization of the residue from EtOH or *i*-PrOH.

General procedure C1b – Amines; direct reductive amination by amine 4

The indicated aldehyde was added to a stirring solution of amine **4** in DCE that was being cooled with an ice bath. The reaction mixture was stirred for the indicated time and $NaBH(OAc)_3$ was added. The reaction mixture was stirred at rt for the indicated time. The reaction mixture was diluted with DCM and washed with 2.0 M aq. NaOH and brine as indicated. The combined organic layers were dried over Na_2SO_4 or $MgSO_4$, filtered, concentrated *in vacuo*, and subjected to column chromatography to provide the desired amine.

General procedure C2b – Amines; indirect reductive amination by amine 4

Amine **4** and $MgSO_4$ were added to a stirring solution of the indicated aldehyde in DCE (2.5 mL). The reaction mixture was stirred at rt overnight, filtered and concentrated *in vacuo*. The residue was dissolved in MeOH (2.5 mL) and $NaBH_4$ was added. The reaction mixture was stirred at rt for 2 h, concentrated *in vacuo* and subjected to column chromatography. Where applicable, the fumarate salt was prepared by dissolving the purified amine and fumaric acid (1.0 eq) in MeOH, concentrating of the resulting solution, and recrystallization of the residue from EtOH or *i*-PrOH.

General procedure D1 – Amides, variant 1

The indicated carboxylic acid, EDCI·HCl, DMAP and DIPEA were added to a solution of amine **4** in DCM. The reaction mixture was stirred for the indicated time at rt. The mixture was diluted with H_2O and extracted with DCM as indicated. The combined organic layers were washed as indicated (if applicable), dried over Na_2SO_4, filtered, and concentrated *in vacuo*. The residue was subjected to column chromatography to provide the desired amide.

General procedure D2 – Amides, variant 2

The indicated carboxylic acid, EDCI·HCl, DMAP and DIPEA (if applicable) were added to a solution of amine **4** in DMF. The reaction mixture was stirred for the indicated time at rt. The mixture was diluted with EtOAc and the organic layer was washed as indicated. Alternatively, the reaction mixture was diluted with H_2O and extracted with DCM as indicated, and the combined organic layers were washed as indicated. The combined organic layers were dried over Na_2SO_4, filtered, and concentrated *in vacuo*. The residue was subjected to column chromatography (if applicable) to provide the desired amide.

General procedure E1 – Ureas using an isocyanate

A solution of the indicated isocyanate in two-thirds of the indicated volume of DCM was added dropwise to a solution of amine **4** in one-third of the indicated volume of DCM. The resulting mixture was stirred for 1 h at rt. The precipitate was collected by vacuum filtration and washed with a minimal amount of DCM to provide the desired urea.

General procedure E2 – Ureas using CDI

Amine **4** was treated with CDI as described in detail for **10a**-**c** in different batches. The resulting intermediate was dissolved in THF and used as stock solution, with the indicated amount of amine **4** having been interpolated from the amount of amine **4** that was treated with CDI to prepare the stock solution.

The indicated amine and Et_3N (if applicable) were added to the indicated amount of stock solution, and the resulting mixture was stirred at the indicated time and temperature. The mixture was diluted with H_2O and extracted with EtOAc. The combined organic layers were washed as indicated (if applicable), dried over Na_2SO_4, filtered and concentrated *in vacuo*. Subsequent purification by column chromatography (if applicable) provided the desired urea.

General procedure F – Sulfonamides

Amine **4** was added to a stirring solution of the indicated sulphonyl chloride and Et_3N in DCM with the mixture being cooled with an ice bath. The reaction mixture was subsequently allowed to warm up to room temperature and stirred for the indicated time. If applicable, the reaction mixture was diluted with 1.0 M aq. HCl and extracted with DCM. The combined organic layers were washed with satd. aq. $NaHCO_3$, dried over Na_2SO_4, filtered, and concentrated *in vacuo*. Alternatively, the reaction mixture was concentrated *in vacuo*. The residue was subjected to column chromatography to provide the desired sulfonamide.

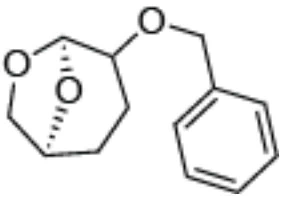

(1S,5R)-4-(benzyloxy)-6,8-dioxabicyclo[3.2.1]octane (5a)

General procedure A1 was performed, using alcohol **2** (250 mg, 1.92 mmol, 1.00 eq), bromomethylbenzene (431 mg, 2.52 mmol, 1.31 eq), DMF (8.0 mL), NaH (60% in mineral oil) (100 mg, 2.50 mmol, 1.30 eq) and TBAI (40 mg, 0.11 mmol, 0.056 eq). Extraction with H_2O (50 mL) and Et_2O (2×50 mL) and subsequent purification by column chromatography (EtOAc/cHex, 0–50%) provided the title compound (300 mg, 71%).

Diastereomeric ratio: 8:1. Proposed major diastereomer: *Endo*.

Its spectral data were in accordance with the literature[1].

^{1}H NMR (500 MHz, $CDCl_3$) δ 7.41 – 7.24 (m, 5H)^{a,b}, 5.42 (s$^{\Delta}$, 0.9H)^{a}, 5.41 – 5.36 (m, 0.1H)^{b}, 4.64 (d, *J* = 12.3 Hz, 0.1H)^{b}, 4.59 (ABq, 1.8H, $\Delta\delta_{AB}$= 0.02, J_{AB}= 12.3 Hz)^{a}, 4.54 (d, *J* = 12.3 Hz, 0.1H)^{b}, 4.52 (s, 0.1H)^{b}, 4.50 – 4.44 (m, 0.9H)^{a}, 3.92 – 3.85 (m, 1H)^{a,b}, 3.81 (ddd$^{\Delta}$, *J* = 6.9, 5.0, 1.5 Hz, 0.9H)^{a}, 3.77 (ddd$^{\Delta}$, *J* = 6.9, 5.1, 1.6 Hz, 0.1H)^{b}, 3.44 (ddd$^{\Delta}$, *J* = 10.3, 5.7, 1.6 Hz, 0.9H)^{a}, 3.32 (ddd$^{\Delta}$, *J* = 4.2, 1.9, 1.9 Hz, 0.1H)^{b}, 2.23 – 2.12 (m, 0.1H)^{b}, 2.01 – 1.92 (m, 0.9H)^{a}, 1.90 – 1.79 (m, 1H)^{a,b}, 1.79 – 1.67 (m, 1H)^{a,b}, 1.64 – 1.56 (m, 0.9H)^{a}, 1.44 – 1.36 (m, 0.1H)^{b}. **^{13}C NMR** (126 MHz, $CDCl_3$) δ 138.5^{b}, 138.4^{a}, 128.5^{a,b}, 127.8^{a,b}, 127.8^{a,b}, 101.1^{a}, 100.9^{b}, 75.8^{a}, 73.2^{a}, 73.2^{b}, 73.0^{b}, 71.4^{b}, 70.8^{a}, 68.5^{a}, 66.8^{b}, 28.0^{a}, 25.3^{b}, 23.0^{a}, 20.3^{b}. **LCMS** (acidic): t_R: 4.03 min, purity: 93.2% (254 nm). **HRMS**: $(M + Na)^+$ calcd. for $C_{13}H_{16}O_3$: 243.0992, found: 243.1001.

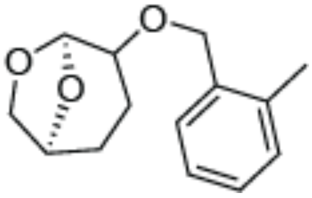

(1S,5R)-4-[(2-methylphenyl)methoxy]-6,8-dioxabicyclo[3.2.1]octane (5b)

General procedure A1 was performed, using alcohol **2** (130 mg, 0.999 mmol, 1.00 eq), 1-(bromomethyl)-2-methylbenzene (203 mg, 1.10 mmol, 1.10 eq), DMF (2.0 mL), NaH (60% in mineral oil) (42 mg, 1.0 mmol, 1.1 eq) and TBAI (18 mg, 0.050 mmol, 0.050 eq). Extraction with H_2O (10 mL) and Et_2O (3×15 mL), followed

by a wash of the combined organic layers with H_2O (4×10 mL) and brine (4×10 mL) and subsequent purification by column chromatography (EtOAc/cHex, 13%) provided the title compound (74 mg, 31%).

Diastereomeric ratio: >9:1. Proposed major diastereomer: *Endo*.

^{1}H NMR (600 MHz, $CDCl_3$) δ 7.33 – 7.29 (m, 1H), 7.22 – 7.13 (m, 3H), 5.43 (s$^{\Delta}$, 1H), 4.58 (ABq, 2H, $\Delta\delta_{AB}$= 0.2, J_{AB}= 12.0 Hz), 4.50 – 4.46 (m, 1H), 3.90 (d$^{\Delta}$, *J* = 7.1 Hz, 1H), 3.82 (ddd$^{\Delta}$, *J* = 6.9, 5.0, 1.5 Hz, 1H), 3.44 (ddd$^{\Delta}$, *J* = 10.3, 5.8, 1.5 Hz, 1H), 2.34 (s, 3H), 2.01 – 1.93 (m, 1H), 1.90 – 1.81 (m, 1H), 1.79 – 1.69 (m, 1H), 1.63 – 1.59 (m, 2H). **^{13}C NMR** (151 MHz, $CDCl_3$) δ 137.0, 136.1, 130.4, 128.9, 128.1, 125.9, 101.1, 75.9, 73.3, 69.4, 68.6, 28.1, 22.9, 19.0. **LCMS** (acidic): t_R: 4.46 min, purity: 90.3% (254 nm). **HRMS**: $(M + Na)^+$ calcd. for $C_{14}H_{18}O_3$: 257.1148, found: 257.1147.

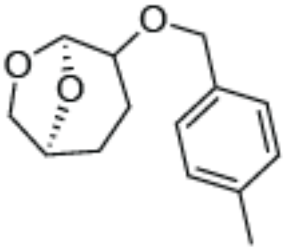

(1S,5R)-4-[(4-methylphenyl)methoxy]-6,8-dioxabicyclo[3.2.1]octane (5c)

General procedure A1 was performed, using alcohol **2** (187 mg, 1.44 mmol, 1.00 eq), 1-(bromomethyl)-4-methylbenzene (299 mg, 1.62 mmol, 1.13 eq), DMF (1.0 mL), NaH (60% in mineral oil) (90 mg, 2.3 mmol, 1.6 eq) and TBAI (27 mg, 0.073 mmol, 0.051 eq). Extraction with H_2O (20 mL) and Et_2O (3×20 mL), followed by a wash of the combined organic layers with H_2O (4×20 mL) and brine (20 mL) and subsequent purification by column chromatography (EtOAc/cHex, 20%) provided the title compound (145 mg, 43%).

Diastereomeric ratio: 9:1. Proposed major diastereomer: *Endo*.

^{1}H NMR (500 MHz, $CDCl_3$) δ 7.22 (m, 2H)^{a,b}, 7.13 (m, 2H)^{a,b}, 5.39 (s$^{\Delta}$, 0.9H)^{a}, 5.37 – 5.33 (m, 0.1H)^{b}, 4.61 – 4.46 (m, 2.1H)^{a,b}, 4.45 – 4.39 (m, 0.9H)^{a}, 3.88 – 3.82 (m, 1H)^{a,b}, 3.77 (dd$^{\Delta}$, *J* = 6.1, 6.1 Hz, 0.9H)^{a}, 3.73 (dd$^{\Delta}$, *J* = 6.4, 5.6 Hz, 0.1H)^{b}, 3.46 – 3.36 (m, 0.9H)^{a}, 3.32 – 3.27 (m, 0.1H)^{b}, 2.32 (m, 3H)^{a,b}, 2.19 – 2.10 (m, 0.1H)^{b}, 1.97 – 1.89 (m, 0.9H)^{a}, 1.86 – 1.76 (m, 1H)^{a,b}, 1.76 – 1.64 (m, 1H)^{a,b}, 1.60 – 1.52 (m, 0.9H)^{a}, 1.36 (dd$^{\Delta}$, *J* = 13.8, 5.9Hz, 0.1H)^{b}. **^{13}C NMR** (126 MHz, $CDCl_3$) δ 137.1^{a,b}, 135.2^{a,b}, 128.9^{a,b}, 127.6^{a,b}, 100.9^{a}, 100.7^{b}, 75.4^{a}, 72.9^{a}, 72.8^{b}, 72.6^{b}, 70.9^{b}, 70.3^{a}, 68.2^{a}, 66.5^{b}, 27.7^{a}, 25.1^{b}, 22.7^{a}, 21.0^{a,b}, 20.0^{b}. **LCMS** (acidic): t_R: 4.45 min, purity: 93.2% (254 nm). **HRMS**: $(M + Na)^+$ calcd. for $C_{14}H_{18}O_3$: 257.1148, found: 257.1161.

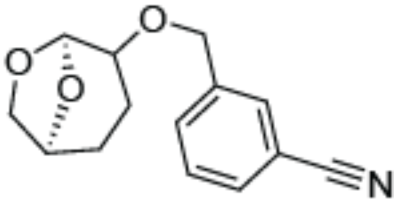

3-{[(1S,5R)-6,8-dioxabicyclo[3.2.1]octan-4-yloxy]methyl}benzonitrile (5d)

General procedure A1 was performed, using alcohol **2** (136 mg, 1.05 mmol, 1.00 eq), 3-(bromomethyl)benzonitrile (228 mg, 1.17 mmol, 1.12 eq), DMF (2.0 mL), NaH (60% in mineral oil) (54 mg, 1.4 mmol, 1.3 eq) and TBAI (21 mg, 0.057 mmol, 0.055 eq). Extraction with H_2O (20 mL) and Et_2O (3×15 mL), followed by a wash of the combined organic layers with H_2O (4×15 mL) and brine (20 mL) and subsequent purification by column chromatography (EtOAc/cHex, 20%) provided the title compound (131 mg, 51%).

Diastereomeric ratio: 9:1. Proposed major diastereomer: *Endo*.

^{1}H NMR (500 MHz, $CDCl_3$) δ 7.68 – 7.63 (m, 1H)^{a,b}, 7.61 – 7.54 (m, 2H)^{a,b}, 7.47 – 7.41 (m, 1H)^{a,b}, 5.45 (s$^{\Delta}$, 0.9H), 5.43 – 5.41 (m, 0.1H)^{b}, 4.66 – 4.55 (m, 2H)^{a,b}, 4.55 – 4.52 (m, 0.1H)^{b}, 4.52 – 4.47 (m, 0.9H)^{a}, 3.94 – 3.88 (m, 1H)^{a,b}, 3.83 (ddd$^{\Delta}$, *J* = 6.9, 5.0, 1.5 Hz, 0.9H)^{a}, 3.79 (ddd$^{\Delta}$, *J* = 6.8, 5.1, 1.6 Hz, 0.1H)^{b}, 3.46 (ddd$^{\Delta}$, *J* = 10.1, 5.7, 1.5 Hz, 0.9H)^{a}, 3.34 (ddd$^{\Delta}$, *J* = 4.1, 1.9, 1.9 Hz, 0.1H)^{b}, 2.21 – 2.11 (m, 0.1H)^{b}, 2.06 – 1.95 (m, 0.9H)^{a}, 1.95 – 1.82 (m, 1H)^{a,b}, 1.81 – 1.69 (m, 1H)^{a,b}, 1.68 – 1.53 (m, 0.9H)^{a}, 1.43 (dd$^{\Delta}$, *J* = 13.9, 6.2 Hz, 0.1H)^{b}. **^{13}C NMR** (126 MHz, $CDCl_3$) δ 140.1^{a,b}, 131.8^{a,b}, 131.4^{a,b}, 131.0^{a,b}, 129.3^{a,b}, 118.9^{a,b}, 112.6^{a,b}, 100.8^{a}, 100.5^{b}, 76.7^{a,}, 74.0^{b}, 73.2^{a}, 73.2^{b}, 70.2^{b}, 69.5^{a}, 68.6^{a}, 66.9^{b}, 28.0^{a}, 25.3^{b}, 22.9^{a}, 20.4^{b}. **LCMS** (acidic): t_R: 3.88 min, purity: 95.8% (254 nm). **HRMS**: $(M + Na)^+$ calcd. for $C_{14}H_{15}NO_3$: 268.0944, found: 268.0957.

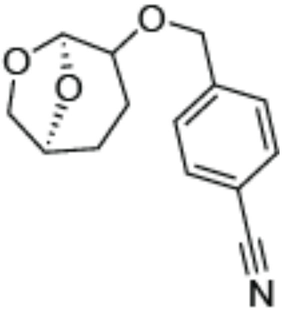

4-{[(1S,5R)-6,8-dioxabicyclo[3.2.1]octan-4-yloxy]methyl}benzonitrile (5e)

General procedure A1 was performed, using alcohol **2** (132 mg, 1.01 mmol, 1.00 eq), 4-(bromomethyl)benzonitrile (219 mg, 1.12 mmol, 1.10 eq), DMF (2.0 mL), NaH (60% in mineral oil) (45 mg, 1.1 mmol, 1.1 eq) and TBAI (19 mg, 0.053 mmol, 0.052 eq). Extraction with H_2O (20 mL) and Et_2O (3×15 mL), followed by a wash of the combined organic layers with H_2O (4×15 mL) and brine (20 mL) and subsequent purification by column chromatography (EtOAc/cHex, 20%) provided the title compound (28 mg, 11%).

Diastereomeric ratio: 9:1. Proposed major diastereomer: *Endo*.

^{1}H NMR (500 MHz, $CDCl_3$) δ 7.66 – 7.60 (m, 2H)^{a,b}, 7.49 – 7.42 (m, 2H)^{a,b}, 5.45 (s$^{\Delta}$, 0.9H)^{a}, 5.42 (s$^{\Delta}$, 0.1H)^{b}, 4.70 – 4.57 (m, 2H)^{a,b}, 4.56 – 4.52 (m, 0.1H)^{b}, 4.52 – 4.48 (m, 0.9H)^{a}, 3.93 – 3.88 (m, 1H)^{a,b}, 3.83 (ddd$^{\Delta}$, *J* = 6.8, 5.1, 1.3 Hz, 0.9H)^{a}, 3.79 (ddd$^{\Delta}$, *J* = 6.8, 5.1, 1.4 Hz, 0.1H)^{b}, 3.46 (ddd$^{\Delta}$, *J* = 10.2, 5.8, 1.3 Hz, 0.9H)^{a}, 3.34 (ddd$^{\Delta}$, *J* = 4.1, 1.8, 1.8 Hz, 0.1H)^{b}, 2.21 – 2.11 (m, 0.1H)^{b}, 2.04 – 1.96 (m, 0.9H)^{a}, 1.93 – 1.82 (m, 1H)^{a,b}, 1.81 – 1.70 (m, 1H)^{a,b}, 1.69 – 1.59 (m, 0.9H)^{a}, 1.43 (dd$^{\Delta}$, *J* = 13.7, 6.2 Hz, 0.1H)^{b}. **^{13}C NMR** (126 MHz, $CDCl_3$) δ 144.0^{a,b}, 132.4^{a,b}, 127.8^{a,b}, 119.0^{a,b}, 111.5^{a,b}, 100.8^{a}, 100.6^{b}, 76.8^{a}, 74.1^{b}, 73.2^{a}, 73.2^{b}, 70.5^{b}, 69.8^{a}, 68.6^{a}, 66.9^{b}, 28.0^{a}, 25.3^{b}, 22.9^{a}, 20.4^{b}. **LCMS** (acidic): t_R: 3.85 min, purity: 93.6% (254 nm). **HRMS**: $(M + Na)^+$ calcd. for $C_{14}H_{15}NO_3$: 268.0944, found: 268.0951.

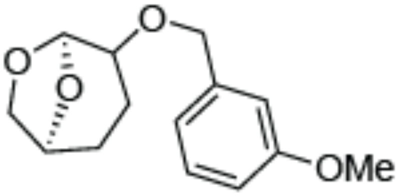

(1S,5R)-4-[(3-methoxyphenyl)methoxy]-6,8-dioxabicyclo[3.2.1]octane (5f)

General procedure A1 was performed, using alcohol **2** (136 mg, 1.05 mmol, 1.00 eq), 1-(chloromethyl)-3-methoxybenzene (180 mg, 1.15 mmol, 1.10 eq), DMF (2.0 mL), NaH (60% in mineral oil) (50 mg, 1.2 mmol, 1.2 eq) and TBAI (22 mg, 0.060 mmol, 0.057 eq). Extraction with H_2O (20 mL) and Et_2O (3×15 mL), followed by a wash of the combined organic layers with H_2O (4×15 mL) and brine (20 mL) and subsequent purification by column chromatography (EtOAc/cHex, 10%) provided the title compound (20 mg, 8%).

Diastereomeric ratio: 9:1. Proposed major diastereomer: *Endo*.

^{1}H NMR (500 MHz, $CDCl_3$) δ 7.29 – 7.20 (m, 1H)[a,b], 6.95 – 6.88 (m, 2H)[a,b], 6.85 – 6.79 (m, 1H)[a,b], 5.42 (s[Δ], 0.9H)[a], 5.38 (s[Δ], 0.1H)[b], 4.64 – 4.49 (m, 2.1H)[a,b], 4.50 – 4.44 (m, 0.9H)[a], 3.93 – 3.86 (m, 1H)[a,b], 3.84 – 3.81 (m, 0.9H)[a,b], 3.81 (m, 3H)[a,b], 3.77 (ddd[Δ], *J* = 6.9, 5.0, 1.3 Hz, 0.1H)[b], 3.43 (ddd[Δ], *J* = 10.2, 5.9, 1.3 Hz, 0.9H)[a], 3.34 – 3.29 (m, 0.1H)[b], 2.23 – 2.13 (m, 0.1H)[b], 2.02 – 1.94 (m, 0.9H)[a], 1.90 – 1.80 (m, 1H)[a,b], 1.80 – 1.68 (m, 1H)[a,b], 1.63 – 1.56 (m, 0.9H)[a], 1.44 – 1.37 (m, 0.1H)[b]. **^{13}C NMR** (126 MHz, $CDCl_3$) δ 159.9[a,b], 140.1[a,b], 129.6[a,b], 120.1[a,b], 113.4[a], 113.4[b], 113.2[b], 113.2[a], 101.2[a], 101.0[b], 75.8[a], 73.3[a,b], 73.2[b], 73.0[b], 71.3[b], 70.7[a], 68.5[a], 66.8[b], 55.4[a,b], 28.0[a], 25.4[b], 23.0[a], 20.3[b]. **LCMS** (acidic): t_R: 4.06 min, purity: 94.4% (254 nm). **HRMS**: $(M + Na)^+$ calcd. for $C_{14}H_{18}O_4$: 273.1097, found: 273.1108.

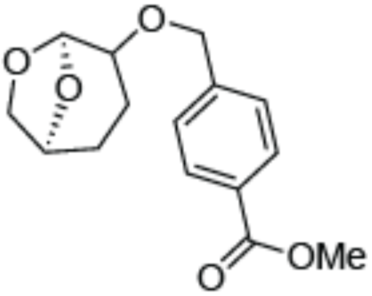

methyl 4-{[(1S,5R)-6,8-dioxabicyclo[3.2.1]octan-4-yloxy]methyl}benzoate (5g)

General procedure A1 was performed, using alcohol **2** (132 mg, 1.01 mmol, 1.00 eq), methyl 4-(bromomethyl)benzoate (232 mg, 1.01 mmol, 1.00 eq), DMF (2.8 mL), NaH (60% in mineral oil) (48 mg, 1.2 mmol, 1.2 eq) and TBAI (22 mg, 0.061 mmol, 0.060 eq). Extraction with H_2O (20 mL) and Et_2O (3×15 mL), followed by a wash of the combined organic layers with H_2O (4×15 mL) and brine (20 mL) and subsequent purification by column chromatography (EtOAc/cHex, 17%) provided the title compound (32 mg, 11%).

Diastereomeric ratio: 9:1. Proposed major diastereomer: *Endo*.

^{1}H NMR (600 MHz, $CDCl_3$) δ 8.01 – 7.95 (m, 2H)[a,b], 7.42 – 7.36 (m, 2H)[a,b], 5.42 (s[Δ], 0.9H)[a], 5.40 – 5.38 (m, 0.1H)[b], 4.65 (d, *J* = 13.0 Hz, 0.1H)[b], 4.61 (ABq, 1.8H, $\Delta\delta_{AB}$= 0.02, J_{AB}= 13.0 Hz)[a], 4.57 (d, *J* = 13.1 Hz, 0.1H)[b], 4.52 – 4.49 (m, 0.1H)[b], 4.48 – 4.44 (m, 0.9H)[b], 3.91 – 3.85 (m, 4H)[a,b], 3.80 (ddd[Δ], *J* = 6.9, 5.0, 1.5 Hz, 0.9H)[a],

3.75 (ddd$^{\Delta}$, *J* = 6.9, 5.1, 1.6 Hz, 0.1H)[b], 3.42 (ddd$^{\Delta}$, *J* = 10.2, 5.8, 1.6 Hz, 0.9H)[a], 3.31 (ddd$^{\Delta}$, *J* = 4.3, 1.9, 1.9 Hz, 0.1H)[b], 2.19 – 2.11 (m, 0.1H)[b], 2.00 – 1.90 (m, 0.9H)[a], 1.88 – 1.76 (m, 1H)[a,b], 1.76 – 1.66 (m, 1H)[a,b], 1.63 – 1.55 (m, 0.9H)[a], 1.42 – 1.35 (m, 0.1H)[b]. **^{13}C NMR** (151 MHz, $CDCl_3$) δ 167.0[a,b], 143.8[b], 143.7[a], 129.8[b], 129.8[a], 129.5[a,b], 127.3[a,b], 100.9[a], 100.7[b], 76.4[a], 73.6[b], 73.2[a], 73.1[b], 70.8[b], 70.1[a], 68.5[a], 66.8[b], 52.1[a,b], 27.9[a], 25.2[b], 22.9[a], 20.3[b]. **LCMS** (acidic): t_R: 4.05 min, purity: 97.2% (254 nm). **HRMS**: $(M + Na)^+$ calcd. for $C_{15}H_{18}O_5$: 301.1046, found: 301.1044.

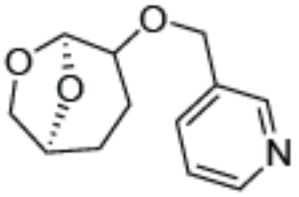

3-{[(1S,5R)-6,8-dioxabicyclo[3.2.1]octan-4-yloxy]methyl}pyridine (5h)

General procedure A2 was performed, using alcohol **2** (137 mg, 1.05 mmol, 1.00 eq), NaH (60% in mineral oil) (46 mg, 1.2 mmol, 1.1 eq) and TBAI (19 mg, 0.053 mmol, 0.050 eq) in DMF (1.0 mL), and 3-(chloromethyl)pyridine hydrochloride (190 mg, 1.16 mmol, 1.10 eq) and NaH (60% in mineral oil) (46 mg, 1.2 mmol, 1.1 eq) in DMF (2.0 mL). Purification by column chromatography (EtOAc/cHex + 1% Et_3N, 20%) provided the title compound (98 mg, 42%).

Diastereomeric ratio: 8:1. Proposed major diastereomer: *Endo*.

^{1}H NMR (500 MHz, $CDCl_3$) δ 8.59 – 8.54 (m, 1H)[a,b], 8.54 – 8.48 (m, 1H)[a,b], 7.76 – 7.70 (m, 1H)[a,b], 7.33 – 7.27 (m, 1H)[a,b], 5.42 (s$^{\Delta}$, 0.9H)[a], 5.38 (s$^{\Delta}$, 0.1H)[b], 4.65 – 4.52 (m, 2H)[a,b], 4.52 – 4.49 (m, 0.1H)[b], 4.49 – 4.45 (m, 0.9H)[a], 3.87 (m, 1H)[a,b], 3.80 (ddd$^{\Delta}$, *J* = 6.8, 5.1, 1.3 Hz, 0.9H)[a], 3.76 (ddd$^{\Delta}$, *J* = 6.8, 5.1, 1.3 Hz, 0.1H)[b], 3.44 (ddd$^{\Delta}$, *J* = 10.2, 5.8, 1.4 Hz, 0.9H)[a], 3.33 (ddd$^{\Delta}$, *J* = 4.1, 2.1, 2.1 Hz, 0.1H)[b], 2.18 – 2.08 (m, 0.1H)[b], 2.05 – 1.92 (m, 0.9H)[a], 1.92 – 1.79 (m, 1H)[a,b], 1.79 – 1.66 (m, 1H)[a,b], 1.66 – 1.57 (m, 0.9H)[a], 1.40 (dd$^{\Delta}$, *J* = 13.7, 6.1 Hz, 0.1H)[b]. **^{13}C NMR** (126 MHz, $CDCl_3$) δ 148.6[a,b], 148.5[b], 148.5[a], 136.1[a], 136.1[b], 134.2[b], 134.2[a], 123.7[b], 123.7[a], 100.8[a], 100.5[b], 76.5[a], 73.7[b], 73.2[a], 73.1[b], 68.8[b], 68.5[a], 68.1[a], 66.8[b], 27.9[a], 25.2[b], 22.8[a], 20.2[b]. **LCMS** (basic): t_R: 2.98 min, purity: 98.3% (254 nm). **HRMS**: $(M + H)^+$ calcd. for $C_{12}H_{15}NO_3$: 222.1125, found: 222.1131.

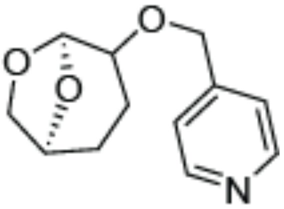

4-{[(1S,5R)-6,8-dioxabicyclo[3.2.1]octan-4-yloxy]methyl}pyridine (5i)

General procedure A2 was performed, using alcohol **2** (144 mg, 1.11 mmol, 1.00 eq), NaH (60% in mineral oil) (52 mg, 1.3 mmol, 1.2 eq) and TBAI (20 mg, 0.055 mmol, 0.050 eq) in DMF (2.0 mL), and 4-(bromomethyl)pyridine hydrobromide (308 mg, 1.22 mmol, 1.10 eq) and NaH (60% in mineral oil) (49 mg, 1.2 mmol, 1.1 eq) in DMF (2.0 mL). Purification by column chromatography (EtOAc/cHex + 1% Et_3N, 20%) provided the title compound (14 mg, 6%).

Diastereomeric ratio: 9:1. Proposed major diastereomer: *Endo*.

^{1}H NMR (500 MHz, $CDCl_3$) δ 8.63 – 8.48 (m, 2H)^{a,b}, 7.33 – 7.27 (m, 2H)^{a,b}, 5.45 (s$^{\Delta}$, 0.9H)^{a}, 5.43 (s$^{\Delta}$, 0.1H)^{b}, 4.64 (d, *J* = 10.6 Hz, 0.1H)^{b}, 4.60 (ABq, $\Delta\delta_{AB}$= 0.01, J_{AB}= 10.1 Hz, 1.8H)^{a}, 4.56 (d, *J* = 9.6 Hz, 0.1H)^{b}, 4.54 – 4.51 (m, 0.1H)^{b}, 4.51 – 4.47 (m, 0.9H)^{a}, 3.94 – 3.86 (m, 1H)^{a,b}, 3.82 (ddd$^{\Delta}$, *J* = 6.8, 5.1, 1.3 Hz, 0.9H)^{a}, 3.78 (ddd$^{\Delta}$, *J* = 6.8, 5.2, 1.4 Hz, 0.1H)^{b}, 3.45 (ddd$^{\Delta}$, *J* = 10.2, 5.8, 1.3 Hz, 0.9H)^{a}, 3.33 (ddd$^{\Delta}$, *J* = 4.2, 2.1, 2.1 Hz, 0.1H)^{b}, 2.21 – 2.11 (m, 0.1H)^{b}, 2.05 – 1.96 (m, 0.9H)^{b}, 1.93 – 1.82 (m, 1H)^{a,b}, 1.81 – 1.69 (m, 1H)^{a,b}, 1.68 – 1.58 (m, 0.9H)^{a}, 1.45 – 1.39 (m, 0.1H)^{b}. **^{13}C NMR** (126 MHz, $CDCl_3$) δ 149.5^{b}, 149.5^{a}, 148.2^{a,b}, 122.0^{a,b}, 100.8^{a}, 100.5^{b}, 76.9^{a}, 74.2^{b}, 73.2^{a}, 73.2^{b}, 69.7^{a}, 69.0^{b}, 68.6^{a}, 66.9^{a}, 27.9^{a}, 25.3^{b}, 22.8^{a}, 20.3^{b}. **LCMS** (acidic): t_R: 1.85 min, purity: 98.0% (254 nm). **HRMS**: $(M + H)^+$ calcd. for $C_{12}H_{15}NO_3$: 222.1125, found: 222.1129.

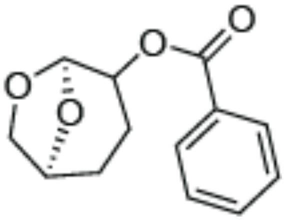

(1S,5R)-6,8-dioxabicyclo[3.2.1]octan-4-yl benzoate (6a)

General procedure B was performed, using alcohol **2** (150 mg, 1.15 mmol, 1.00 eq), benzoic acid (170 mg, 1.39 mmol, 1.21 eq), DCM (5.0 mL), EDCI·HCl (289 mg, 1.51 mmol, 1.31 eq), DMAP (33 mg, 0.27 mmol, 0.23 eq) and DIPEA (0.48 mL, 2.8 mmol, 2.4 eq). Extraction with satd. aq. $NaHCO_3$ (20 mL) and DCM (3×15 mL), followed by a wash of the combined organic layers with satd. aq. NH_4Cl (20 mL) and subsequent purification by column chromatography (EtOAc/cHex, 20%) provided the title compound (104 mg, 39%).

Diastereomeric ratio: >9:1. Proposed major diastereomer: *Endo*.

^{1}H NMR (500 MHz, $CDCl_3$) δ 8.06 (d$^{\Delta}$, *J* = 7.3 Hz, 2H), 7.56 (t$^{\Delta}$, *J* = 7.4, 7.4 Hz, 1H), 7.43 (dd$^{\Delta}$, *J* = 7.7, 7.7 Hz, 2H), 5.52 (s$^{\Delta}$, 1H), 4.99 (dd$^{\Delta}$, *J* = 10.3, 5.9 Hz, 1H), 4.62 – 4.54 (m, 1H), 3.98 (d$^{\Delta}$, *J* = 7.1 Hz, 1H), 3.88 (dd$^{\Delta}$, *J* = 6.0, 6.0 Hz, 1H), 2.18 – 2.09 (m, 1H), 2.09 – 1.98 (m, 1H), 1.98 – 1.87 (m, 1H), 1.70 (dd$^{\Delta}$, *J* = 13.7, 5.7 Hz, 1H). **^{13}C NMR** (126 MHz, $CDCl_3$) δ 166.0, 133.3, 129.9, 128.5, 128.5, 100.7, 73.3, 72.0, 68.7, 28.0, 22.1. **LCMS** (acidic): t_R: 4.21 min, purity: 97.5% (254 nm). **HRMS**: $(M + Na)^+$ calcd. for $C_{13}H_{14}O_4$: 257.0784, found: 257.0791.

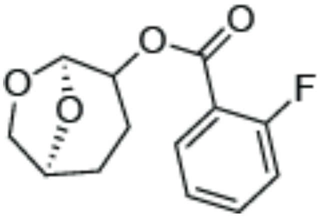

(1S,5R)-6,8-dioxabicyclo[3.2.1]octan-4-yl 2-fluorobenzoate (6b)

General procedure B was performed, using alcohol **2** (130 mg, 0.999 mmol, 1.00 eq), 2-fluorobenzoic acid (168 mg, 1.20 mmol, 1.20 eq), DCM (3.0 mL), EDCI·HCl (230 mg, 1.20 mmol, 1.20 eq), DMAP (24 mg, 0.20 mmol, 0.20 eq) and DIPEA (0.42 mL, 2.4 mmol, 2.4 eq). Extraction with H_2O (25 mL) and DCM (3×25 mL), followed by a wash of the combined organic layers with satd. aq. $NaHCO_3$ (15 mL), satd. aq. NH_4Cl (2×15

mL) and brine (15 mL) and subsequent purification by column chromatography (EtOAc/cHex, 25%) provided the title compound (16 mg, 6%).

Diastereomeric ratio: >9:1. Proposed major diastereomer: *Endo*.

^{1}H NMR (600 MHz, $CDCl_3$) δ 7.93 (ddd^Δ, *J* = 7.5, 7.5, 1.9 Hz, 1H), 7.54 – 7.48 (m, 1H), 7.19 (dd^Δ, *J* = 7.6, 7.6 Hz, 1H), 7.12 (dd^Δ, *J* = 10.9, 8.3 Hz, 1H), 5.53 (s^Δ, 1H), 4.99 (ddd, *J* = 10.4, 6.0, 1.6 Hz, 1H), 4.59 – 4.54 (m, 1H), 3.97 (d^Δ, *J* = 7.1 Hz, 1H), 3.87 (ddd, *J* = 6.9, 5.0, 1.5 Hz, 1H), 2.17 – 2.11 (m, 1H), 2.07 – 1.98 (m, 1H), 1.96 – 1.88 (m, 1H), 1.71 – 1.67 (m, 1H). **^{13}C NMR** (151 MHz, $CDCl_3$) δ 163.57 (d, *J* = 3.7 Hz), 162.10 (d, *J* = 260.7 Hz), 134.64 (d, *J* = 9.1 Hz), 132.1, 123.92 (d, *J* = 3.9 Hz), 118.55 (d, *J* = 9.4 Hz), 117.01 (d, *J* = 22.1 Hz), 100.4, 73.2, 72.2, 68.6, 27.9, 21.9. **LCMS** (acidic): t_R: 4.13 min, purity: 98.6% (254 nm). **HRMS**: $(M + Na)^+$ calcd. for $C_{13}H_{13}FO_4$: 275.0690, found: 275.0686.

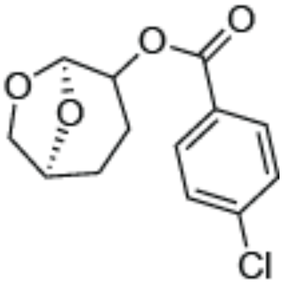

(1S,5R)-6,8-dioxabicyclo[3.2.1]octan-4-yl 4-chlorobenzoate (6c)

General procedure B was performed, using alcohol **2** (131 mg, 1.01 mmol, 1.00 eq), 4-chlorobenzoic acid (201 mg, 1.28 mmol, 1.28 eq), DCM (4.0 mL), EDCI·HCl (236 mg, 1.23 mmol, 1.22 eq), DMAP (26 mg, 0.22 mmol, 0.22 eq) and DIPEA (0.42 mL, 2.4 mmol, 2.4 eq). Extraction with H_2O (20 mL) and DCM (3×15 mL), followed by a wash of the combined organic layers with brine (20 mL) and subsequent purification by column chromatography (EtOAc/cHex, 20%) provided the title compound (93 mg, 34%).

Diastereomeric ratio: >9:1. Proposed major diastereomer: *Endo*.

^{1}H NMR (500 MHz, $CDCl_3$) δ 8.05 – 7.94 (m, 2H), 7.45 – 7.36 (m, 2H), 5.50 (s^Δ, 1H), 4.97 (ddd, *J* = 10.6, 6.0, 1.6 Hz, 1H), 4.60 – 4.56 (m, 1H), 3.98 (d^Δ, *J* = 7.1 Hz, 1H), 3.88 (ddd, *J* = 6.8, 5.0, 1.5 Hz, 1H), 2.16 – 2.08 (m, 1H), 2.08 – 1.98 (m, 1H), 1.97 – 1.85 (m, 1H), 1.75 – 1.66 (m, 1H). **^{13}C NMR** (126 MHz, $CDCl_3$) δ 165.2, 139.7, 131.3, 128.8, 128.5, 100.6, 73.3, 72.2, 68.7, 28.0, 22.1. **LCMS** (acidic): t_R: 4.71 min, purity: >99% (254 nm). **HRMS**: $(M + Na)^+$ calcd. for $C_{13}H_{13}ClO_4$: 291.0395, found: 291.0407.

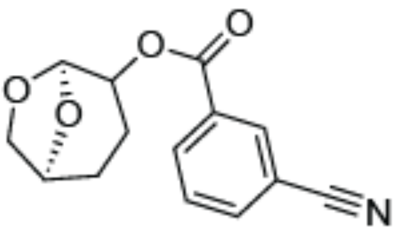

(1S,5R)-6,8-dioxabicyclo[3.2.1]octan-4-yl 3-cyanobenzoate (6d)

General procedure B was performed, using alcohol **2** (153 mg, 1.18 mmol, 1.00 eq), 3-cyanobenzoic acid (212 mg, 1.44 mmol, 1.22 eq), DCM (4.0 mL), EDCI·HCl (270 mg, 1.41 mmol, 1.20 eq), DMAP (40 mg, 0.33 mmol, 0.28 eq) and DIPEA (0.49 mL, 2.8 mmol, 2.4 eq). Extraction with H_2O (20 mL) and DCM (3×15 mL), followed by a wash of the combined organic layers with satd. aq. NH_4Cl (20 mL) and subsequent purification by column chromatography (EtOAc/cHex, 20%) provided the title compound (117 mg, 38%).

Diastereomeric ratio: >9:1. Proposed major diastereomer: *Endo*.

^{1}H NMR (500 MHz, $CDCl_3$) δ 8.33 (dd, *J* = 1.4, 1.4 Hz, 1H), 8.27 (ddd, *J* = 7.9, 1.4, 1.4 Hz, 1H), 7.83 (ddd, *J* = 7.8, 1.4, 1.4 Hz, 1H), 7.57 (dd^Δ, *J* = 7.9, 7.9 Hz, 1H), 5.50 (s^Δ, 1H), 4.99 (ddd, *J* = 10.5, 6.1, 1.4 Hz, 1H), 4.60 – 4.54 (m, 1H), 3.98 (d^Δ, *J* = 7.2 Hz, 1H), 3.88 (ddd, *J* = 6.8, 5.1, 1.2 Hz, 1H), 2.16 – 2.08 (m, 1H), 2.08 – 1.99 (m, 1H), 1.97 – 1.86 (m, 1H), 1.77 – 1.62 (m, 1H). **^{13}C NMR** (126 MHz, $CDCl_3$) δ 164.0, 136.3, 133.9, 133.5, 131.3, 129.5, 117.9, 113.1, 100.3, 73.2, 72.8, 68.7, 27.9, 22.0. **LCMS** (acidic): t_R: 4.04 min, purity: >99% (254 nm). **HRMS**: $(M + Na)^+$ calcd. for $C_{14}H_{13}NO_4$: 282.0737, found: 282.0747.

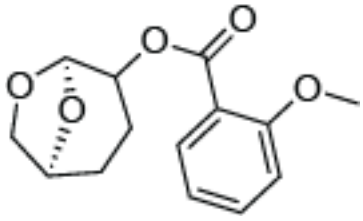

(1S,5R)-6,8-dioxabicyclo[3.2.1]octan-4-yl 2-methoxybenzoate (6e)

General procedure B was performed, using alcohol **2** (137 mg, 1.05 mmol, 1.00 eq), 2-methoxybenzoic acid (193 mg, 1.27 mmol, 1.21 eq), DCM (4.2 mL), EDCI·HCl (243 mg, 1.27 mmol, 1.21 eq), DMAP (26 mg, 0.21 mmol, 0.20 eq) and DIPEA (0.44 mL, 2.5 mmol, 2.4 eq). Extraction with H_2O (50 mL) and DCM (3×50 mL), followed by a wash of the combined organic layers with satd. aq. NH_4Cl (50 mL) and brine (50 mL) and subsequent purification by column chromatography (EtOAc/cHex, 50%) provided the title compound (61 mg, 22%).

Diastereomeric ratio: >9:1. Proposed major diastereomer: *Endo*.

^{1}H NMR (500 MHz, $CDCl_3$) δ 7.82 (dd, *J* = 7.9, 1.8 Hz, 1H), 7.46 (ddd, *J* = 8.4, 7.4, 1.8 Hz, 1H), 7.02 – 6.92 (m, 2H), 5.52 (s^Δ, 1H), 4.97 (ddd, *J* = 10.5, 6.0, 1.6 Hz, 1H), 4.57 – 4.54 (m, 1H), 3.96 (d^Δ, *J* = 7.1 Hz, 1H), 3.89 (s, 3H), 3.86 (ddd, *J* = 6.9, 5.1, 1.6 Hz, 1H), 2.17 – 2.09 (m, 1H), 2.06 – 1.97 (m, 1H), 1.95 – 1.83 (m, 1H), 1.72 – 1.64 (m, 1H). **^{13}C NMR** (126 MHz, $CDCl_3$) δ 165.3, 159.5, 133.8, 131.9, 120.2, 119.8, 112.1, 100.7, 73.3, 71.7, 68.6, 56.1, 28.0, 22.1. **LCMS** (acidic): t_R: 3.93 min, purity: >99% (254 nm). **HRMS**: $(M + Na)^+$ calcd. for $C_{14}H_{16}O_5$: 287.0890, found: 287.0891.

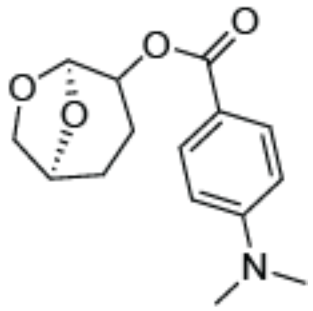

(1S,5R)-6,8-dioxabicyclo[3.2.1]octan-4-yl 4-(dimethylamino)benzoate (6f)

General procedure B was performed, using alcohol **2** (145 mg, 1.11 mmol, 1.00 eq), 4-(dimethylamino)benzoic acid (223 mg, 1.35 mmol, 1.21 eq), DCM (4.0 mL), EDCI·HCl (270 mg, 1.41 mmol, 1.26 eq), DMAP (31 mg, 0.26 mmol, 0.23 eq) and DIPEA (0.74 mL, 4.2 mmol, 3.8 eq). Extraction with H_2O (20 mL) and DCM (3×15 mL), followed by a wash of the combined organic layers with brine (20 mL) and subsequent purification by column chromatography (EtOAc/cHex, 20%) provided the title compound (58 mg, 19%).

Diastereomeric ratio: >9:1. Proposed major diastereomer: *Endo*.

^{1}H NMR (500 MHz, $CDCl_3$) δ 7.91 (m, 2H), 6.63 (m, 2H), 5.49 (s$^\Delta$, 1H), 4.93 (ddd, *J* = 10.5, 5.9, 1.6 Hz, 1H), 4.56 – 4.50 (m, 1H), 3.96 (d$^\Delta$, *J* = 7.1 Hz, 1H), 3.85 (ddd, *J* = 6.8, 5.1, 1.5 Hz, 1H), 3.02 (s, 6H), 2.13 – 2.04 (m, 1H), 2.06 – 1.95 (m, 1H), 1.94 – 1.82 (m, 1H), 1.66 (dd$^\Delta$, *J* = 13.7, 5.8 Hz, 1H). **^{13}C NMR** (126 MHz, $CDCl_3$) δ 166.2, 153.4, 131.6, 116.8, 110.9, 101.0, 73.2, 71.1, 68.6, 40.2, 28.0, 22.2. **LCMS** (acidic): t_R: 4.41 min, purity: 93.8% (254 nm). **HRMS**: $(M + H)^+$ calcd. for $C_{15}H_{19}NO_4$: 278.1387, found: 278.1400.

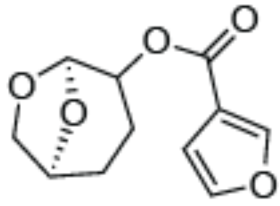

(1S,5R)-6,8-dioxabicyclo[3.2.1]octan-4-yl furan-3-carboxylate (6g)

General procedure B was performed, using alcohol **2** (150 mg, 1.15 mmol, 1.00 eq), furan-3-carboxylic acid (158 mg, 1.41 mmol, 1.22 eq), DCM (4.6 mL), EDCI·HCl (300 mg, 1.57 mmol, 1.36 eq), DMAP (30 mg, 0.25 mmol, 0.21 eq) and DIPEA (0.48 mL, 2.8 mmol, 2.4 eq). Extraction with H_2O (20 mL) and DCM (3×15 mL), followed by a wash of the combined organic layers with brine (20 mL) and subsequent purification by column chromatography (EtOAc/cHex, 20%) provided the title compound (13 mg, 5%).

Diastereomeric ratio: >9:1. Proposed major diastereomer: *Endo*.

^{1}H NMR (500 MHz, $CDCl_3$) δ 8.04 (dd, *J* = 1.5, 0.7 Hz, 1H), 7.41 (dd, *J* = 1.7, 1.7 Hz, 1H), 6.75 (dd, *J* = 2.0, 0.8 Hz, 1H), 5.47 (s$^\Delta$, 1H), 4.91 (ddd, *J* = 10.5, 5.9, 1.6 Hz, 1H), 4.57 – 4.53 (m, 1H), 3.95 (d$^\Delta$, *J* = 7.1 Hz, 1H), 3.86 (ddd, *J* = 6.9, 5.0, 1.5 Hz, 1H), 2.11 – 2.05 (m, 1H), 2.05 – 1.96 (m, 1H), 1.91 – 1.81 (m, 1H), 1.67 (dd$^\Delta$, *J* = 13.7, 5.9 Hz, 1H). **^{13}C NMR** (126 MHz, $CDCl_3$) δ 162.6, 148.2, 143.9, 119.2, 110.0, 100.6, 73.3, 71.6, 68.7, 28.0, 22.1. **LCMS** (acidic): t_R: 3.68 min, purity: 98.3% (254 nm). **HRMS**: $(M + Na)^+$ calcd. for $C_{11}H_{12}O_5$: 247.0577, found: 247.0586.

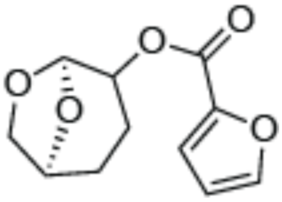

(1S,5R)-6,8-dioxabicyclo[3.2.1]octan-4-yl furan-2-carboxylate (6h)

General procedure B was performed, using alcohol **2** (162 mg, 1.24 mmol, 1.00 eq), furan-2-carboxylic acid (167 mg, 1.49 mmol, 1.20 eq), DCM (4.0 mL), EDCI·HCl (286 mg, 1.49 mmol, 1.20 eq), DMAP (30 mg, 0.25 mmol, 0.20 eq) and DIPEA (0.52 mL, 3.0 mmol, 2.4 eq). Extraction with H_2O (20 mL) and DCM (3×15 mL), followed by a wash of the combined organic layers with satd. aq. NH_4Cl (20 mL) and subsequent purification by column chromatography (EtOAc/cHex, 20%) provided the title compound (102 mg, 37%).

Diastereomeric ratio: >9:1. Proposed major diastereomer: *Endo*.

^{1}H NMR (500 MHz, $CDCl_3$) δ 7.61 – 7.55 (m, 1H), 7.22 (d$^\Delta$, *J* = 3.5 Hz, 1H), 6.50 (dd, *J* = 3.5, 1.7 Hz, 1H), 5.49 (s$^\Delta$, 1H), 4.96 (ddd$^\Delta$, *J* = 10.0, 5.8, 1.2 Hz, 1H), 4.60 – 4.53 (m, 1H), 3.97 ($^{d\Delta}$, *J* = 7.1 Hz, 1H), 3.90 – 3.83 (m, 1H), 2.16 – 2.06 (m, 1H), 2.07 – 1.96 (m, 1H), 1.96 – 1.85 (m, 1H), 1.69 (dd$^\Delta$, *J* = 13.6, 5.7 Hz, 1H). **^{13}C NMR** (126 MHz, $CDCl_3$) δ 158.1, 146.7, 144.4, 118.6, 112.0, 100.5, 73.3, 72.0, 68.7, 28.0, 22.1. **LCMS** (acidic): t_R: 3.56 min, purity: 98.9% (254 nm). **HRMS**: $(M + Na)^+$ calcd. for $C_{11}H_{12}O_5$: 247.0577, found: 247.0583.

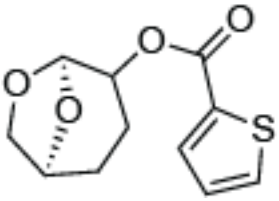

(1S,5R)-6,8-dioxabicyclo[3.2.1]octan-4-yl thiophene-2-carboxylate (6i)

General procedure B was performed, using alcohol **2** (300 mg, 2.31 mmol, 1.00 eq), thiophene-2-carboxylic acid (354 mg, 2.77 mmol, 1.20 eq), DCM (3.0 mL), EDCI·HCl (530 mg, 2.77 mmol, 1.20 eq), DMAP (56 mg, 0.46 mmol, 0.20 eq) and DIPEA (0.96 mL, 5.5 mmol, 2.4 eq). Extraction with H_2O (25 mL) and DCM (25 mL), followed by a wash of the combined organic layers with satd. aq. $NaHCO_3$ (15 mL), satd. aq. NH_4Cl (2×15 mL) and brine (15 mL) and subsequent purification by column chromatography (EtOAc/cHex, 17%) provided the title compound (117 mg, 21%).

Diastereomeric ratio: >9:1. Proposed major diastereomer: *Endo*.

^{1}H NMR (600 MHz, $CDCl_3$) δ 7.80 (dd, *J* = 3.7, 1.3 Hz, 1H), 7.54 (dd, *J* = 4.9, 1.3 Hz, 1H), 7.07 (dd, *J* = 5.0, 3.7 Hz, 1H), 5.48 (s$^\Delta$, 1H), 4.91 (ddd, *J* = 10.4, 5.9, 1.6 Hz, 1H), 4.55 – 4.51 (m, 1H), 3.94 (d$^\Delta$, *J* = 7.2 Hz, 1H), 3.84 (ddd, *J* = 6.9, 5.0, 1.5 Hz, 1H), 2.11 – 2.05 (m, 1H), 2.03 – 1.94 (m, 1H), 1.93 – 1.84 (m, 1H), 1.66 (dd$^\Delta$, *J* = 14.0, 5.6 Hz, 1H). **^{13}C NMR** (151 MHz, $CDCl_3$) δ 161.6, 133.8, 133.5, 132.8, 127.8, 100.5, 73.2, 72.2, 68.6, 27.9, 22.0. **LCMS** (acidic): t_R: 3.98 min, purity: >99% (254 nm). **HRMS**: $(M + H)^+$ calcd. for $C_{11}H_{12}O_4S$: 241.0529, found: 241.0536.

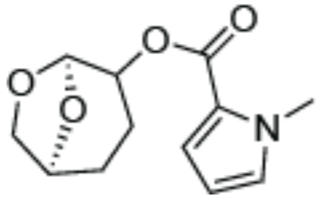

(1S,5R)-6,8-dioxabicyclo[3.2.1]octan-4-yl 1-methyl-1H-pyrrole-2-carboxylate (6j)

General procedure B was performed, using alcohol **2** (148 mg, 1.14 mmol, 1.00 eq), 1-methylpyrrole-2-carboxylic acid (171 mg, 1.36 mmol, 1.20 eq), DCM (4.0 mL), EDCI·HCl (261 mg, 1.36 mmol, 1.20 eq), DMAP (30 mg, 0.25 mmol, 0.22 eq) and DIPEA (0.48 mL, 2.7 mmol, 2.4 eq). Extraction with H_2O (20 mL) and DCM (3×15 mL), followed by a wash of the combined organic layers with brine (20 mL) and subsequent purification by column chromatography (EtOAc/cHex, 20%) provided the title compound (70 mg, 26%).

Diastereomeric ratio: >9:1. Proposed major diastereomer: *Endo*.

^{1}H NMR (500 MHz, $CDCl_3$) δ 7.01 (dd, *J* = 4.0, 1.8 Hz, 1H), 6.78 (dd, *J* = 2.1, 2.1 Hz, 1H), 6.10 (dd, *J* = 4.0, 2.5 Hz, 1H), 5.47 (s^{Δ}, 1H), 4.89 (ddd, *J* = 10.1, 5.8, 1.4 Hz, 1H), 4.58 – 4.51 (m, 1H), 3.95 (d^{Δ}, *J* = 7.1 Hz, 1H), 3.90 (s, 3H), 3.85 (ddd, *J* = 6.8, 5.1, 1.3 Hz, 1H), 2.11 – 2.03 (m, 1H), 2.03 – 1.95 (m, 1H), 1.93 – 1.82 (m, 1H), 1.70 – 1.63 (m, 1H). **^{13}C NMR** (126 MHz, $CDCl_3$) δ 160.6, 129.9, 122.2, 118.7, 108.0, 100.9, 73.3, 70.9, 68.6, 37.0, 28.0, 22.2. **LCMS** (acidic): t_R: 3.99 min, purity: >99% (254 nm). **HRMS**: $(M + Na)^+$ calcd. for $C_{12}H_{15}NO_4$: 260.0893, found: 260.0904.

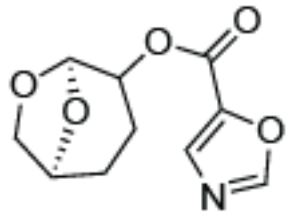

(1S,5R)-6,8-dioxabicyclo[3.2.1]octan-4-yl 1,3-oxazole-5-carboxylate (6k)

EDCI·HCl (232 mg, 1.21 mmol, 1.20 eq), DMAP (25 mg, 0.20 mmol, 0.20 eq), DIPEA (0.42 mL, 2.4 mmol, 2.4 eq) and 1,3-oxazole-5-carboxylic acid (137 mg, 1.21 mmol, 1.20 eq) were added to a stirring solution of alcohol **2** (131 mg, 1.01 mmol, 1.00 eq) in DCM (4.0 mL). The resulting solution was stirred for 6 d at rt. DMF (0.5 mL) was added and the vial was placed in the microwave for 30 min at 40 °C, 30 min at 80 °C, and 5 min at 140 °C. The reaction mixture was diluted with H_2O and extracted with DCM (3×). The combined organic layers were washed with satd. aq. NH_4Cl, dried over Na_2SO_4, filtered and concentrated *in vacuo*. The residue was subjected to column chromatography (EtOAc/cHex, 20–30%) to provide the title compound (40 mg, 18%).

Diastereomeric ratio: >9:1. Proposed major diastereomer: *Endo*.

^{1}H NMR (500 MHz, $CDCl_3$) δ 8.01 (s, 1H), 7.81 (s, 1H), 5.48 (s^{Δ}, 1H), 4.97 (ddd, *J* = 10.3, 6.0, 1.6 Hz, 1H), 4.59 – 4.54 (m, 1H), 3.96 (d^{Δ}, *J* = 7.1 Hz, 1H), 3.87 (ddd, *J* = 6.9, 5.0, 1.5 Hz, 1H), 2.14 – 2.07 (m, 1H), 2.07 – 1.97 (m, 1H), 1.96 – 1.85 (m, 1H), 1.75 – 1.65 (m, 1H). **^{13}C NMR** (126 MHz, $CDCl_3$) δ 157.0, 153.6, 142.5, 134.1, 100.2, 73.2, 72.7, 68.7, 28.0, 22.0. **LCMS** (acidic): t_R: 2.98 min, purity: >99% (254 nm). **HRMS**: $(M + H)^+$ calcd. for $C_{10}H_{11}NO_5$: 226.0710, found: 226.0716.

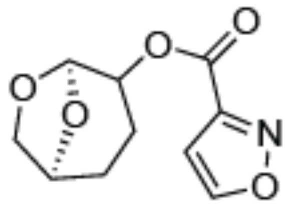

(1S,5R)-6,8-dioxabicyclo[3.2.1]octan-4-yl 1,2-oxazole-3-carboxylate (6l)

General procedure B was performed, using alcohol **2** (137 mg, 1.05 mmol, 1.00 eq), 1,2-oxazole-3-carboxylic acid (127 mg, 1.13 mmol, 1.07 eq), DCM (4.0 mL), EDCI·HCl (248 mg, 1.29 mmol, 1.23 eq), DMAP (26 mg, 0.21 mmol, 0.20 eq) and DIPEA (0.44 mL, 2.5 mmol, 2.4 eq). Extraction with H_2O (20 mL) and DCM (3×15 mL), followed by a wash of the combined organic layers with satd. aq. NH_4Cl (20 mL) and subsequent purification by column chromatography (EtOAc/cHex, 20%) provided the title compound (10 mg, 4%).

Diastereomeric ratio: >9:1. Proposed major diastereomer: *Endo*.

^{1}H NMR (500 MHz, $CDCl_3$) δ 8.53 (d, *J* = 1.7 Hz, 1H), 6.79 (d, *J* = 1.7 Hz, 1H), 5.53 (s$^\Delta$, 1H), 5.02 (ddd, *J* = 10.2, 5.9, 1.3 Hz, 1H), 4.59 – 4.55 (m, 1H), 3.97 (d$^\Delta$, *J* = 7.2 Hz, 1H), 3.87 (ddd, *J* = 6.8, 5.1, 1.1 Hz, 1H), 2.19 – 2.10 (m, 1H), 2.08 – 1.99 (m, 1H), 1.99 – 1.91 (m, 1H), 1.75 – 1.68 (m, 1H). **^{13}C NMR** (126 MHz, $CDCl_3$) δ 160.1, 159.3, 155.4, 105.4, 100.0, 73.3, 73.2, 68.7, 28.0, 21.8. **LCMS** (acidic): t_R: 3.19 min, purity: 90.4% (254 nm). **HRMS**: $(M + Na)^+$ calcd. for $C_{10}H_{11}NO_5$: 248.0529, found: 248.0539.

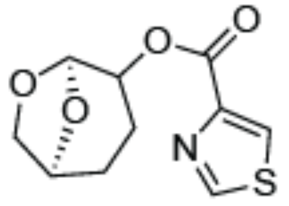

(1S,5R)-6,8-dioxabicyclo[3.2.1]octan-4-yl 1,3-thiazole-4-carboxylate (6m)

General procedure B was performed, using alcohol **2** (137 mg, 1.05 mmol, 1.00 eq), 1,3-thiazole-4-carboxylic acid (181 mg, 1.40 mmol, 1.33 eq), DCM (4.2 mL), EDCI·HCl (256 mg, 1.34 mmol, 1.27 eq), DMAP (27 mg, 0.22 mmol, 0.21 eq) and DIPEA (0.44 mL, 2.5 mmol, 2.4 eq). Extraction with H_2O (50 mL) and DCM (3×50 mL), followed by a wash of the combined organic layers with satd. aq. NH_4Cl (50 mL) and brine (50 mL) and subsequent purification by column chromatography (EtOAc/cHex, 33%) provided the title compound (57 mg, 22%).

Diastereomeric ratio: >9:1. Proposed major diastereomer: *Endo*.

^{1}H NMR (500 MHz, $CDCl_3$) δ 8.83 (d, *J* = 2.1 Hz, 1H), 8.25 (d, *J* = 2.1 Hz, 1H), 5.51 (s$^\Delta$, 1H), 5.00 (ddd, *J* = 10.2, 5.8, 1.6 Hz, 1H), 4.57 – 4.52 (m, 1H), 3.95 (d$^\Delta$, *J* = 7.2 Hz, 1H), 3.84 (ddd, *J* = 6.9, 5.0, 1.5 Hz, 1H), 2.16 – 2.08 (m, 1H), 2.05 – 1.97 (m, 1H), 1.97 – 1.88 (m, 1H), 1.72 – 1.64 (m, 1H). **^{13}C NMR** (126 MHz, $CDCl_3$) δ 160.5, 153.6, 147.7, 127.8, 100.3, 73.2, 72.4, 68.6, 28.0, 22.0. **LCMS** (acidic): t_R: 2.93 min, purity: >99% (254 nm). **HRMS**: $(M + Na)^+$ calcd. for $C_{10}H_{11}NO_4S$: 264.0301, found: 264.0300.

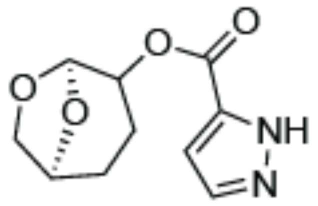

(1S,5R)-6,8-dioxabicyclo[3.2.1]octan-4-yl 1H-pyrazole-5-carboxylate (6n)

General procedure B was performed, using alcohol **2** (133 mg, 1.02 mmol, 1.00 eq), 1H-pyrazole-5-carboxylic acid (152 mg, 1.36 mmol, 1.33 eq), DCM (3.0 mL), EDCI·HCl (254 mg, 1.32 mmol, 1.29 eq), DMAP (34 mg, 0.28 mmol, 0.27 eq) and DIPEA (0.45 mL, 2.6 mmol, 2.5 eq). Extraction with H_2O (20 mL) and DCM (20 mL), followed by a wash of the combined organic layers with satd. aq. NH_4Cl (20 mL) and brine (20 mL) and subsequent purification by column chromatography (EtOAc/cHex, 33%) provided the title compound (34 mg, 15%).

Diastereomeric ratio: >9:1. Proposed major diastereomer: *Endo*.

^{1}H NMR (600 MHz, $CDCl_3$) δ 7.88 (s$^{\Delta}$, 1H), 7.52 (s, 1H), 6.94 (s$^{\Delta}$, 1H), 5.54 (s$^{\Delta}$, 1H), 5.02 (dd$^{\Delta}$, *J* = 10.1, 5.8 Hz, 1H), 4.60 – 4.55 (m, 1H), 3.99 (d, *J* = 7.0 Hz, 1H), 3.91 – 3.87 (m, 1H), 2.19 – 2.10 (m, 1H), 2.07 – 1.99 (m, 1H), 1.96 – 1.88 (m, 1H), 1.71 (dd$^{\Delta}$, *J* = 13.8, 5.5 Hz, 1H). **^{13}C NMR** (151 MHz, $CDCl_3$) δ 160.3, 140.0, 133.5, 108.9, 100.4, 73.3, 72.5, 68.7, 28.0, 22.1. **LCMS** (acidic): t_R: 2.81 min, purity: >99% (254 nm). **HRMS**: $(M + Na)^+$ calcd. for $C_{10}H_{12}N_2O_4$: 247.0689, found: 247.0700.

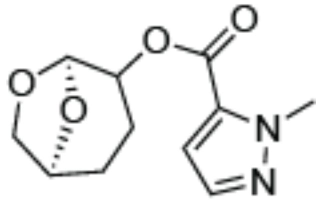

(1S,5R)-6,8-dioxabicyclo[3.2.1]octan-4-yl 1-methyl-1H-pyrazole-5-carboxylate (6o)

General procedure B was performed, using alcohol **2** (133 mg, 1.02 mmol, 1.00 eq), 2-methylpyrazole-3-carboxylic acid (155 mg, 1.23 mmol, 1.21 eq), DCM (4.0 mL), EDCI·HCl (232 mg, 1.21 mmol, 1.18 eq), DMAP (30 mg, 0.24 mmol, 0.24 eq) and DIPEA (0.43 mL, 2.5 mmol, 2.4 eq). Extraction with H_2O (20 mL) and DCM (3×15 mL), followed by a wash of the combined organic layers with satd. aq. NH_4Cl (20 mL) and subsequent purification by column chromatography (EtOAc/cHex, 20%) provided the title compound (114 mg, 47%).

Diastereomeric ratio: >9:1. Proposed major diastereomer: *Endo*.

^{1}H NMR (500 MHz, $CDCl_3$) δ 7.45 (d, *J* = 2.1 Hz, 1H), 6.89 (d, *J* = 2.1 Hz, 1H), 5.48 (s$^{\Delta}$, 1H), 4.93 (ddd, *J* = 10.5, 5.9, 1.6 Hz, 1H), 4.58 – 4.55 (m, 1H), 4.17 (s, 3H), 3.96 (d$^{\Delta}$, *J* = 7.2 Hz, 1H), 3.87 (ddd, *J* = 7.0, 5.0, 1.6 Hz, 1H), 2.13 – 2.06 (m, 1H), 2.06 – 1.97 (m, 1H), 1.94 – 1.84 (m, 1H), 1.72 – 1.64 (m, 1H). **^{13}C NMR** (126 MHz, $CDCl_3$) δ 159.2, 137.9, 132.2, 111.9, 100.4, 73.3, 72.1, 68.7, 39.8, 28.0, 22.0. **LCMS** (acidic): t_R: 3.43 min, purity: >99% (254 nm). **HRMS**: $(M + H)^+$ calcd. for $C_{11}H_{14}N_2O_4$: 239.1026, found: 239.1038.

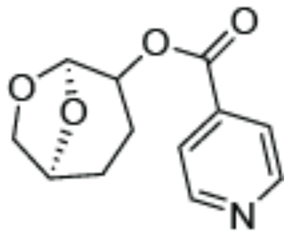

(1S,5R)-6,8-dioxabicyclo[3.2.1]octan-4-yl pyridine-4-carboxylate (6p)

General procedure B was performed, using alcohol **2** (130 mg, 0.999 mmol, 1.00 eq), pyridine-4-carboxylic acid (148 mg, 1.20 mmol, 1.20 eq), DCM (3.0 mL), EDCI·HCl (230 mg, 1.20 mmol, 1.20 eq), DMAP (24 mg, 0.20 mmol, 0.20 eq) and DIPEA (0.42 mL, 2.4 mmol, 2.4 eq). Extraction with H_2O (25 mL) and DCM (25 mL), followed by a wash of the combined organic layers with satd. aq. $NaHCO_3$ (15 mL), satd. aq. NH_4Cl (2×15 mL) and brine (15 mL) and subsequent purification by column chromatography (EtOAc+1%Et_3N) provided the title compound (51 mg, 22%).

Diastereomeric ratio: >9:1. Proposed major diastereomer: *Endo*.

^{1}H NMR (600 MHz, $CDCl_3$) δ 8.76 (m, 2H), 7.89 – 7.84 (m, 2H), 5.48 (s^Δ, 1H), 4.97 (ddd, *J* = 10.4, 6.0, 1.6 Hz, 1H), 4.58 – 4.53 (m, 1H), 3.95 (d^Δ, *J* = 7.2 Hz, 1H), 3.86 (ddd, *J* = 6.9, 5.1, 1.5 Hz, 1H), 2.14 – 2.07 (m, 1H), 2.06 – 1.97 (m, 1H), 1.95 – 1.85 (m, 1H), 1.72 – 1.65 (m, 1H). **^{13}C NMR** (151 MHz, $CDCl_3$) δ 164.4, 150.2, 137.6, 123.2, 100.2, 73.2, 72.9, 68.7, 27.9, 21.9. **LCMS** (acidic): t_R: 2.93 min, purity: >99% (254 nm). **HRMS**: $(M + H)^+$ calcd. for $C_{12}H_{13}NO_4$: 236.0917, found: 236.0925.

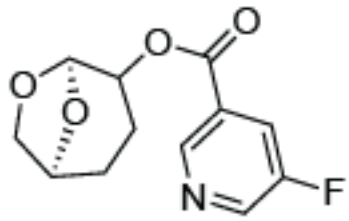

(1S,5R)-6,8-dioxabicyclo[3.2.1]octan-4-yl 5-fluoropyridine-3-carboxylate (6q)

General procedure B was performed, using alcohol **2** (135 mg, 1.04 mmol, 1.00 eq), 5-fluoropyridine-3-carboxylic acid (184 mg, 1.30 mmol, 1.26 eq), DCM (4.0 mL), EDCI·HCl (245 mg, 1.28 mmol, 1.23 eq), DMAP (35 mg, 0.29 mmol, 0.28 eq) and DIPEA (0.44 mL, 2.5 mmol, 2.4 eq). Extraction with H_2O (20 mL) and DCM (3×15 mL), followed by a wash of the combined organic layers with satd. aq. NH_4Cl (20 mL) and subsequent purification by column chromatography (EtOAc/cHex, 20%) provided the title compound (72 mg, 27%).

Diastereomeric ratio: >9:1. Proposed major diastereomer: *Endo*.

^{1}H NMR (500 MHz, $CDCl_3$) δ 9.06 (s^Δ, 1H), 8.65 (d^Δ, *J* = 2.8 Hz, 1H), 8.01 (ddd, *J* = 8.5, 2.8, 1.7 Hz, 1H), 5.51 (s^Δ, 1H), 5.01 (ddd, *J* = 10.5, 6.1, 1.4 Hz, 1H), 4.60 – 4.56 (m, 1H), 3.98 (d^Δ, *J* = 7.2 Hz, 1H), 3.88 (ddd, *J* = 6.8, 5.1, 1.3 Hz, 1H), 2.17 – 2.10 (m, 1H), 2.09 – 2.00 (m, 1H), 1.99 – 1.88 (m, 1H), 1.72 (dd^Δ, *J* = 13.6, 5.8 Hz, 1H). **^{13}C NMR** (126 MHz, $CDCl_3$) δ 163.65 (d, *J* = 2.1 Hz), 159.18 (d, *J* = 257.9 Hz), 146.91 (d, *J* = 4.1 Hz), 142.42 (d, *J* = 23.2 Hz), 127.35 (d, *J* = 3.5 Hz), 123.91 (d, *J* = 19.5 Hz), 100.3, 73.3, 72.9, 68.7, 28.0, 22.0. **LCMS** (acidic): t_R: 3.55 min, purity: >99% (254 nm). **HRMS**: $(M + H)^+$ calcd. for $C_{12}H_{12}FNO_4$: 254.0823, found: 254.0832.

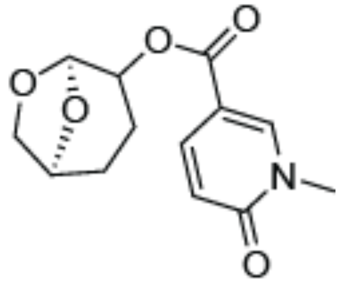

(1S,5R)-6,8-dioxabicyclo[3.2.1]octan-4-yl 1-methyl-6-oxo-1,6-dihydropyridine-3-carboxylate (6r)

General procedure B was performed, using alcohol **2** (131 mg, 1.01 mmol, 1.00 eq), 1-methyl-6-oxopyridine-3-carboxylic acid (186 mg, 1.22 mmol, 1.21 eq), DCM (4.0 mL), EDCI·HCl (234 mg, 1.22 mmol, 1.22 eq), DMAP (25 mg, 0.20 mmol, 0.20 eq) and DIPEA (0.42 mL, 2.4 mmol, 2.4 eq). Extraction with H_2O (30 mL) and DCM (3×20 mL), followed by a wash of the combined organic layers with satd. aq. NH_4Cl (20 mL) and subsequent purification by column chromatography (EtOAc/cHex, 20%) provided the title compound (10 mg, 4%).

Diastereomeric ratio: >9:1. Proposed major diastereomer: *Endo*.

^{1}H NMR (500 MHz, $CDCl_3$) δ 8.87 (s^Δ, 1H), 8.17 (dd, *J* = 8.7, 2.3 Hz, 1H), 6.76 (d^Δ, *J* = 8.7 Hz, 1H), 5.51 (s^Δ, 1H), 4.97 (ddd, *J* = 10.4, 6.0, 1.3 Hz, 1H), 4.62 – 4.51 (m, 1H), 4.00 (s, 3H), 3.97 (d^Δ, *J* = 7.1 Hz, 1H), 3.87 (ddd, *J* = 6.7, 4.8, 1.2 Hz, 1H), 2.15 – 2.08 (m, 1H), 2.08 – 1.98 (m, 1H), 1.97 – 1.86 (m, 1H), 1.70 (dd^Δ, *J* = 13.6, 5.8 Hz, 1H). **^{13}C NMR** (126 MHz, $CDCl_3$) δ 167.0, 164.8, 150.3, 139.9, 119.6, 110.8, 100.6, 73.3, 72.0, 68.7, 54.3, 28.0, 22.1. **LCMS** (acidic): t_R: 3.92 min, purity: >99% (254 nm). **HRMS**: $(M + H)^+$ calcd. for $C_{13}H_{15}NO_5$: 266.1023, found: 266.1032.

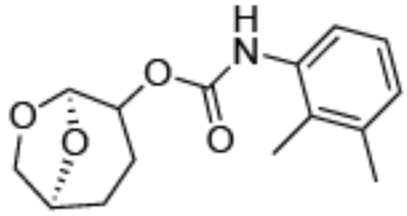

(1S,5R)-6,8-dioxabicyclo[3.2.1]octan-4-yl N-(2,3-dimethylphenyl)carbamate (7a)

Alcohol **2** (260 mg, 2.00 mmol, 1.00 eq) was added to a stirring solution of 1-isocyanato-2,3-dimethylbenzene (735 mg, 4.99 mmol, 2.50 eq) and Et_3N (0.28 mL, 2.0 mmol, 1.0 eq) in THF (10.0 mL). The mixture was stirred overnight at rt, diluted with EtOAc (10 mL) and washed with H_2O (2×15 mL). The organic layer was dried over $MgSO_4$, filtered, and concentrated *in vacuo*. The residue was subjected to column chromatography (EtOAc/cHex, 20%) to provide the title compound (185 mg, 33%).

Diastereomeric ratio: >19:1. Proposed major diastereomer: *Endo*.

^{1}H NMR (500 MHz, $CDCl_3$) δ 7.52 (d^Δ, *J* = 6.2 Hz, 1H), 7.09 (dd, *J* = 7.8, 7.8 Hz, 1H), 6.96 (d^Δ, *J* = 7.5 Hz, 1H), 6.50 (s, 1H), 5.50 (s^Δ, 1H), 4.77 (ddd, *J* = 10.5, 6.0, 1.6 Hz, 1H), 4.59 – 4.51 (m, 1H), 3.95 (d^Δ, *J* = 7.1 Hz, 1H), 3.86 (ddd, *J* = 6.9, 5.1, 1.5 Hz, 1H), 2.29 (s, 3H), 2.14 (s, 3H), 2.18 – 2.06 (m, 1H), 2.04 – 1.94 (m, 1H), 1.87 – 1.75 (m, 1H), 1.70 – 1.63 (m, 1H). **^{13}C NMR** (126 MHz, $CDCl_3$) δ 153.3, 137.4, 135.4, 126.7, 126.1, 120.3,

100.9, 73.2, 68.6, 27.9, 22.3, 20.8, 13.6; (2 missing signals). **LCMS** (acidic): t_R: 3.95 min, purity: 98.1% (230 nm). **HRMS**: $(M + H)^+$ calcd. for $C_{15}H_{19}NO_4$: 278.1387, found: 278.1383.

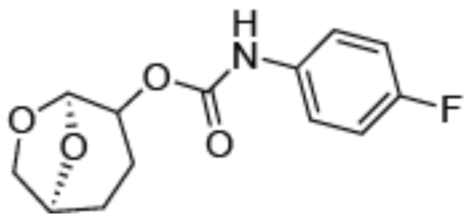

(1S,5R)-6,8-dioxabicyclo[3.2.1]octan-4-yl N-(4-fluorophenyl)carbamate (7b)

1-Fluoro-4-isocyanatobenzene (680 mg, 4.96 mmol, 2.89 eq) was added to a stirring solution of alcohol **2** (223 mg, 1.71 mmol, 1.00 eq) and Et_3N (0.25 mL, 1.8 mmol, 1.0 eq) in THF (5.5 mL). The mixture was stirred for 3 d at rt, diluted with EtOAc (10 mL) and washed with H_2O (2×15 mL). The organic layer was dried over $MgSO_4$, filtered, and concentrated *in vacuo*. The residue was subjected to column chromatography (EtOAc/cHex, 25%) and subsequently (MeOH/DCM, 25%) to provide the title compound (148 mg, 32%).

Diastereomeric ratio: >19:1. Proposed major diastereomer: *Endo*.

^{1}H NMR (500 MHz, $CDCl_3$) δ 7.36 – 7.28 (m, 2H), 7.04 – 6.96 (m, 2H), 6.70 (s, 1H), 5.47 (s^Δ, 1H), 4.76 (ddd, *J* = 10.4, 6.0, 1.6 Hz, 1H), 4.59 – 4.52 (m, 1H), 3.94 (d^Δ, *J* = 7.1 Hz, 1H), 3.86 (ddd, *J* = 6.9, 5.0, 1.5 Hz, 1H), 2.12 – 2.05 (m, 1H), 2.04 – 1.95 (m, 1H), 1.85 – 1.75 (m, 1H), 1.67 (dd^Δ, *J* = 13.9, 6.1 Hz, 1H). **^{13}C NMR** (126 MHz, $CDCl_3$) δ 159.2 (d, *J* = 242.5 Hz), 133.7, 120.5 (s^Δ), 115.9 (d, *J* = 22.6 Hz), 100.8, 73.2, 72.0, 68.6, 27.9, 22.3; (one missing signal). **LCMS** (acidic): t_R: 3.76 min, purity: 97.8% (230 nm). **HRMS**: $(M + H)^+$ calcd. for $C_{13}H_{14}FNO_4$: 268.0980, found: 268.0972.

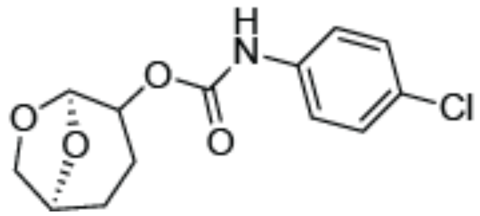

(1S,5R)-6,8-dioxabicyclo[3.2.1]octan-4-yl (4-chlorophenyl)carbamate (7c)

Et_3N (0.45 mL, 3.2 mmol, 2.4 eq) was added to a stirring solution of alcohol **2** (174 mg, 1.34 mmol, 1.00 eq) in THF (3.0 mL). The resulting mixture was stirred for 1 h at rt, and CDI (238 mg, 1.47 mmol, 1.10 eq) was added. The mixture was stirred for 15 min at rt. 4-Chloroaniline (189 mg, 1.48 mmol, 1.11 eq) was added. The mixture was stirred overnight at 40 °C and for an additional 24 h at 50 °C. The reaction mixture was diluted with H_2O (5 mL) and extracted with EtOAc (3x5 mL). The combined organic layers were dried over $MgSO_4$, filtered, and concentrated *in vacuo*. The residue was subjected to reverse phase column chromatography (0.1% HCOOH in MeCN and 0.1% HCOOH in H_2O, 5-95%) to provide the title compound (34 mg, 9%).

Diastereomeric ratio: >9:1. Proposed major diastereomer: *Endo*.

^{1}**H NMR** (300 MHz, $CDCl_3$) δ 7.36 – 7.20 (m, 4H), 6.74 (s, 1H), 5.46 (s^Δ, 1H), 4.75 (dd^Δ, *J* = 10.3, 5.9 Hz, 1H), 4.59 – 4.50 (m, 1H), 3.93 (d^Δ, *J* = 7.1 Hz, 1H), 3.85 (dd^Δ, *J* = 6.2, 6.2 Hz, 1H), 2.15 – 1.91 (m, 2H), 1.88 – 1.57 (m, 2H). ^{13}**C NMR** (76 MHz, $CDCl_3$) δ 152.5, 136.4, 129.2, 128.7, 119.9, 100.7, 73.2, 72.1, 68.6, 27.9, 22.2. **LCMS** (acidic): t_R: 4.26 min, purity: 96.6% (230 nm). **HRMS**: $(M + H)^+$ calcd. for $C_{13}H_{14}ClNO_4$: 284.0684, found: 284.0692.

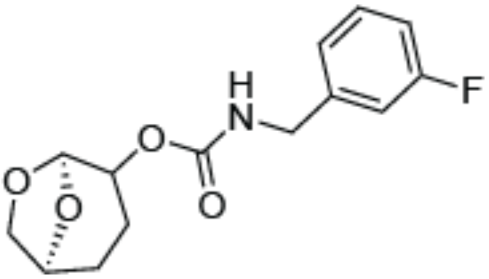

(1S,5R)-6,8-dioxabicyclo[3.2.1]octan-4-yl (3-fluorobenzyl)carbamate (7d)

NaH (60% in mineral oil) (107 mg, 2.67 mmol, 1.50 eq) was added to a stirring solution of alcohol **2** (232 mg, 1.78 mmol, 1.00 eq) in THF (7.5 mL) (H_2 gas liberation; safety warning). The mixture was stirred for 30 min at rt, and CDI (434 mg, 2.67 mmol, 1.50 eq) was added. The mixture was stirred at rt overnight. (3-Fluorophenyl)methanamine (335 mg, 2.67 mmol, 1.50 eq) was added, and the mixture was stirred overnight at rt. The reaction mixture was diluted with H_2O (8 mL) and extracted with EtOAc (2×8 mL). The combined organic layers were dried over Na_2SO_4, filtered, and concentrated *in vacuo*. The residue was subjected to column chromatography (EtOAc/cHex, 30%) to provide the title compound (92 mg, 18%).

Diastereomeric ratio: 8:1. Proposed major diastereomer: *Endo*.

^{1}**H NMR** (300 MHz, $CDCl_3$) δ 7.37 – 7.27 (m, 1H), 7.10 – 6.89 (m, 3H), 5.43 (s^Δ, 1H), 5.16 (s^Δ, 1H), 4.69 (ddd, *J* = 10.2, 6.0, 1.6 Hz, 1H), 4.57 – 4.48 (m, 1H), 4.42 – 4.25 (m, 2H), 3.90 (d^Δ, *J* = 7.0 Hz, 1H), 3.83 (ddd, *J* = 6.9, 5.0, 1.3 Hz, 1H), 2.18 – 1.85 (m, 2H), 1.83 – 1.58 (m, 3H). ^{13}**C NMR** (76 MHz, $CDCl_3$) δ 163.1 (d, *J* = 245.9 Hz)*, 155.7, 140.9*, 130.3 (d, *J* = 8.2 Hz), 123.0 (d, *J* = 3.0 Hz), 114.5 (d, *J* = 21.0 Hz), 114.4 (d, *J* = 21.7 Hz), 100.9, 73.2, 71.9, 68.6, 44.6, 27.9, 22.3. **LCMS** (acidic): t_R: 3.78 min, purity: 97.9% (230 nm). **HRMS**: $(M + H)^+$ calcd. for $C_{14}H_{16}FNO_4$: 281.1136, found: 282.1142

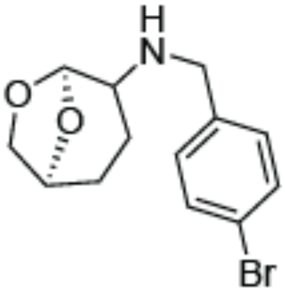

(1S,5R)-N-[(4-bromophenyl)methyl]-6,8-dioxabicyclo[3.2.1]octan-4-amine (8a)

General procedure C1a was performed, using Cyrene (150 mg, 1.17 mmol, 1.00 eq), (4-bromophenyl)methanamine (218 mg, 1.17 mmol, 1.00 eq), DCM (2.5 mL), $NaBH(OAc)_3$ (323 mg, 1.52 mmol, 1.30 eq) and AcOH (0.10 mL, 1.7 mmol, 1.5 eq), and a reaction time of 2 h and 3 d, respectively.

Extraction with 1.0 aq. M NaOH (40 mL) and DCM (15 mL), followed by a wash of the combined organic layers with H_2O (2×20 mL) and subsequent purification by column chromatography (EtOAc/cHex, 45%) provided the title compound (87 mg, 25%).

Diastereomeric ratio: 5:1. Proposed major diastereomer: *Endo*.

^{1}H NMR (500 MHz, $CDCl_3$) δ 7.47 – 7.40 (m, 2H)[a,b], 7.24 – 7.18 (m, 2H)[a,b], 5.41 (s[Δ], 0.8H)[a], 5.40 – 5.37 (m, 0.2H)[b], 4.52 – 4.48 (m, 0.8H)[a], 4.48 – 4.45 (m, 0.2H)[b], 3.91 (d[Δ], *J* = 7.1 Hz, 0.2H)[b], 3.85 – 3.71 (m, 3.8H)[a,b], 2.64 (ddd, *J* = 10.7, 5.6, 1.4 Hz, 0.8H)[a], 2.62 – 2.59 (m, 0.2H)[b], 2.05 – 1.98 (m, 0.2H)[b], 1.98 – 1.91 (m, 0.8H)[a], 1.91 – 1.80 (m, 1H)[a,b], 1.71 – 1.64 (m, 0.2H)[b], 1.61 – 1.49 (m, 0.8H)[a], 1.46 – 1.35 (m, 1H)[a,b]. **^{13}C NMR** (126 MHz, $CDCl_3$) δ 139.6[a,b], 131.6[a], 131.6[b], 129.9[a,b], 120.8[a,b], 102.7[b], 102.2[a], 73.4[b], 73.2[a], 68.3[a], 67.1[b], 56.5[a], 54.7[b], 50.6[b], 49.9[a], 28.3[a], 25.3[b], 24.5[a], 19.8[b]. **LCMS** (acidic): t_R: 2.41 min, purity: 97.5% (254 nm). **HRMS**: $(M + H)^+$ calcd. for $C_{13}H_{16}BrNO_2$: 298.0437, found: 298.0431.

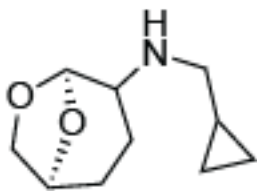

(1S,5R)-N-(cyclopropylmethyl)-6,8-dioxabicyclo[3.2.1]octan-4-amine (8b)

General procedure C2a was performed, using amine **4** (275 mg, 2.13 mmol, 1.00 eq), cyclopropylmethanamine (158 mg, 2.22 mmol, 1.04 eq), DCM (2.6 mL), $NaBH_4$ (81 mg, 2.1 mmol, 1.0 eq) and $MgSO_4$ (339 mg, 2.82 mmol, 1.32 eq). Purification by column chromatography (MeOH/DCM + 1% Et_3N, 0–10%) provided the title compound (184 mg, 47%).

Diastereomeric ratio: >9:1. Proposed major diastereomer: *Endo*.

^{1}H NMR (500 MHz, $CDCl_3$) δ 5.41 (s[Δ], 1H), 4.50 – 4.46 (m, 1H), 3.82 (d[Δ], *J* = 7.0 Hz, 1H), 3.77 (ddd, *J* = 6.8, 5.1, 1.4 Hz, 1H), 2.67 (ddd, *J* = 11.1, 5.6, 1.2 Hz, 1H), 2.57 – 2.42 (m, 2H), 1.97 – 1.89 (m, 1H), 1.89 – 1.81 (m, 1H), 1.71 – 1.58 (m, 1H), 1.59 – 1.51 (m, 1H), 1.43 – 1.31 (m, 1H), 0.99 – 0.85 (m, 1H), 0.52 – 0.41 (m, 2H), 0.17 – 0.03 (m, 2H). **^{13}C NMR** (126 MHz, $CDCl_3$) δ 102.1, 73.2, 68.3, 57.1, 51.9, 28.3, 24.4, 11.7, 3.6. **LCMS** (basic): t_R: 3.26 min, purity: >99% (254 nm). **HRMS**: $(M + H)^+$ calcd. for $C_{10}H_{17}NO_2$: 184.1332, found: 184.1332.

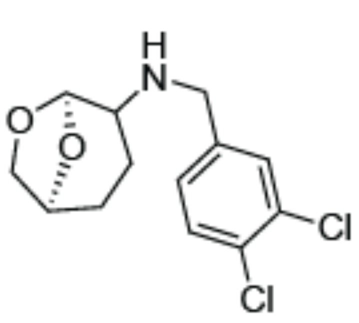

(1S,5R)-N-[(3,4-dichlorophenyl)methyl]-6,8-dioxabicyclo[3.2.1]octan-4-amine (8c)

General procedure C1a was performed, using Cyrene (192 mg, 1.50 mmol, 1.00 eq), (3,4-dichlorophenyl)methanamine (264 mg, 1.50 mmol, 1.00 eq), DCM (2.0 mL), NaBH(OAc)$_3$ (413 mg, 1.95 mmol, 1.30 eq) and AcOH (0.11 mL, 1.9 mmol, 1.3 eq), and a reaction time of 1 h and overnight, respectively. Extraction with 1.0 M aq. NaOH and EtOAc (2×) and subsequent purification by column chromatography (EtOAc/cHex, 0–65%) provided the title compound (61 mg, 14%).

Diastereomeric ratio: 3:1. Proposed major diastereomer: *Endo*.

^{1}H NMR (500 MHz, CDCl$_3$) δ 7.47 (d, *J* = 2.0 Hz, 0.3H)^b, 7.45 (d, *J* = 2.0 Hz, 0.7H)^a, 7.37 (d, *J* = 8.2 Hz, 0.3H)^b, 7.37 (d, *J* = 8.2 Hz, 0.7H)^a, 7.19 (dd, *J* = 8.3, 2.0 Hz, 0.3H)^b, 7.17 (dd, *J* = 8.3, 2.0 Hz, 0.7H)^a, 5.41 (s$^\Delta$, 0.7H)^a, 5.40 – 5.37 (m, 0.3H)^b, 4.55 – 4.49 (m, 0.7H)^a, 4.49 – 4.45 (m, 0.3H)^b, 3.92 (d, *J* = 7.0 Hz, 0.3H)^b, 3.86 – 3.81 (m, 1H)^{a,b}, 3.81 – 3.72 (m, 2.7H)^{a,b}, 2.62 (ddd, *J* = 10.8, 5.5, 1.4 Hz, 0.7H)^a, 2.60 – 2.58 (m, 0.3H)^b, 2.03 – 1.92 (m, 1H)^{a,b}, 1.92 – 1.81 (m, 1H)^{a,b}, 1.71 – 1.64 (m, 0.3H)^b, 1.57 (dd$^\Delta$, *J* = 14.0, 6.0 Hz, 0.7H)^b, 1.46 – 1.35 (m, 1H)^{a,b}. **^{13}C NMR** (126 MHz, CDCl$_3$) δ 141.1^b, 141.0^a, 130.4^a, 130.4^b, 130.0^b, 130.0^a, 127.4^{a,b}, 102.7^b, 102.2^a, 73.4^b, 73.3^a, 68.4^a, 67.1^b, 56.5^a, 54.8^b, 50.1^b, 49.4^a, 28.2^a, 25.3^b, 24.5^a, 19.7^b. **LCMS** (acidic): t_R: 2.63 min, purity: 97.6% (230 nm). **HRMS**: (M + H)$^+$ calcd. for $C_{13}H_{15}Cl_2NO_2$: 288.0553, found: 288.0547.

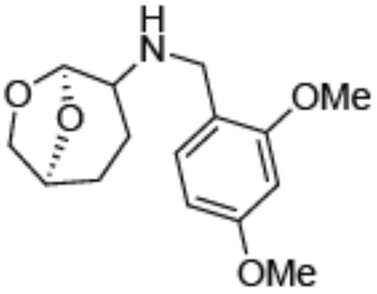

(1S,5R)-N-[(2,4-dimethoxyphenyl)methyl]-6,8-dioxabicyclo[3.2.1]octan-4-amine (8d)

General procedure C1a was performed, using Cyrene (128 mg, 0.999 mmol, 1.00 eq), (2,4-dimethoxyphenyl)methanamine (167 mg, 0.999 mmol, 1.00 eq), DCM (2.0 mL), NaBH(OAc)$_3$ (275 mg, 1.30 mmol, 1.30 eq) and AcOH (0.069 mL, 1.2 mmol, 1.2 eq), and a reaction time of 2.5 h and two nights, respectively. Extraction with 1.0 M aq. NaOH (10 mL) and EtOAc (2×10 mL), followed by a wash of the combined organic layers with H_2O (2×10 mL) and subsequent purification by column chromatography (EtOAc/cHex, 60–100%) provided the title compound (192 mg, 69%).

Diastereomeric ratio: 4:1. Proposed major diastereomer: *Endo*.

^{1}H NMR (500 MHz, CDCl$_3$) δ 7.16 (d$^\Delta$, *J* = 8.1 Hz, 0.2H)^b, 7.14 (d$^\Delta$, *J* = 7.9 Hz, 0.8H)^a, 6.47 – 6.40 (m, 2H)^{a,b}, 5.40 (s$^\Delta$, 0.8H)^a, 5.36 – 5.34 (m, 0.2H)^b, 4.50 – 4.43 (m, 1H)^{a,b}, 3.90 (d$^\Delta$, *J* = 7.0 Hz, 0.2H)^b, 3.86 – 3.59 (m, 9.8H)^{a,b}, 2.65 (ddd, *J* = 10.7, 5.4, 1.4 Hz, 0.8H)^a, 2.63 – 2.61 (m, 0.2H)^b, 2.06 – 1.96 (m, 0.2H)^b, 1.93 – 1.86 (m, 0.8H)^a, 1.86 – 1.79 (m, 1H)^{a,b}, 1.76 – 1.61 (m, 0.2H)^b, 1.57 – 1.49 (m, 0.8H)^a, 1.47 – 1.34 (m, 1H)^{a,b}. **^{13}C NMR** (126 MHz, CDCl$_3$) δ 160.2^b, 160.1^a, 158.6^a, 158.6^b, 130.3^b, 130.2^a, 121.3^a, 121.3^b, 104.0^b, 103.9^a, 103.0^b, 102.5^a, 98.6^{a,b}, 73.4^b, 73.2^a, 68.3^a, 67.0^b, 56.9^a, 55.5^b, 55.5^a, 55.4^{a,b}, 55.2^b, 46.7^b, 45.8^a, 28.3^a, 25.4^b, 24.4^a, 20.0^b. **LCMS** (acidic): t_R: 2.37 min, purity: 96.0% (254 nm). **HRMS**: (M + H)$^+$ calcd. for $C_{15}H_{21}NO_4$: 280.1543, found: 280.1542.

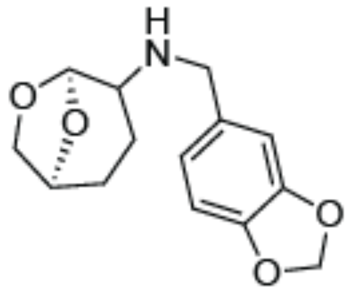

(1S,5R)-N-[(2H-1,3-benzodioxol-5-yl)methyl]-6,8-dioxabicyclo[3.2.1]octan-4-amine (8e)

General procedure C1a was performed, using Cyrene (256 mg, 2.00 mmol, 1.00 eq), 1,3-benzodioxol-5-ylmethanamine (302 mg, 2.00 mmol, 1.00 eq), DCM (2.7 mL), $NaBH(OAc)_3$ (550 mg, 2.60 mmol, 1.30 eq) and AcOH (0.14 mL, 2.4 mmol, 1.2 eq), and a reaction time of 1 h and overnight, respectively. Extraction with 1.0 M aq. NaOH (10 mL) and EtOAc (2×10 mL), followed by a wash of the combined organic layers with H_2O (2×10 mL) and subsequent purification by column chromatography (MeOH/DCM, 5%) provided the title compound (97 mg, 18%).

Diastereomeric ratio: 3:1. Proposed major diastereomer: *Endo*.

^{1}H NMR (500 MHz, $CDCl_3$) δ 6.87 (d, *J* = 1.6 Hz, 0.2H)^b, 6.85 (d, *J* = 1.1 Hz, 0.8H)^a, 6.79 – 6.72 (m, 2H)^{a,b}, 5.93 (s^Δ, 0.5H)^b, 5.93 (s^Δ, 1.5H)^a, 5.40 (s^Δ, 0.8H)^a, 5.39 – 5.36 (m, 0.2H)^b, 4.50 – 4.47 (m, 0.8H)^a, 4.47 – 4.44 (m, 0.2H)^b, 3.91 (d^Δ, *J* = 7.1 Hz, 0.2H)^b, 3.83 (d^Δ, *J* = 7.0 Hz, 0.8H)^a, 3.81 – 3.67 (m, 3H)^{a,b}, 2.65 (ddd, *J* = 10.7, 5.5, 1.4 Hz, 0.8H)^a, 2.63 – 2.60 (m, 0.2H)^b, 2.05 – 1.98 (m, 0.2H)^b, 1.98 – 1.91 (m, 0.8H)^a, 1.91 – 1.80 (m, 1H)^{a,b}, 1.71 – 1.64 (m, 0.2H)^b, 1.58 – 1.53 (m, 0.8H)^a, 1.44 – 1.35 (m, 1H)^{a,b}. **^{13}C NMR** (126 MHz, $CDCl_3$) δ 147.8^{a,b}, 146.6^{a,b}, 134.6^b, 134.5^a, 121.2^a, 121.2^b, 108.7^b, 108.7^a, 108.2^a, 108.2^b, 102.8^b, 102.3^a, 101.0^{a,b}, 73.4^b, 73.2^a, 68.3^a, 67.1^b, 56.3^a, 54.5^b, 51.1^b, 50.4^a, 28.3^a, 25.3^b, 24.5^a, 19.8^b. **LCMS** (acidic): t_R: 2.08 min, purity: 96.1% (230 nm). **HRMS**: $(M + H)^+$ calcd. for $C_{14}H_{17}NO_4$: 264.1230, found: 264.1224.

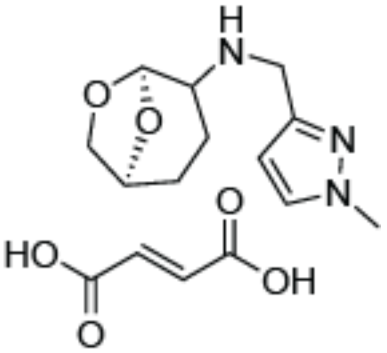

(1S,5R)-N-[(1-methyl-1H-pyrazol-3-yl)methyl]-6,8-dioxabicyclo[3.2.1]octan-4-amine (8f)

General procedure C2a was performed, using amine **4** (256 mg, 1.98 mmol, 1.00 eq), (1-methylpyrazol-3-yl)methanamine (220 mg, 1.98 mmol, 1.00 eq), DCM (2.5 mL), $NaBH_4$ (75 mg, 2.0 mmol, 1.0 eq) and $MgSO_4$ (239 mg, 1.98 mmol, 1.00 eq). Purification by column chromatography (MeOH/DCM + 1% Et_3N, 0–10%) and salt formation provided the title compound as a fumaric acid salt (75 mg, 11%; ratio base:fumaric acid 3:2).

Diastereomeric ratio: >19:1.

^{1}H NMR (500 MHz, CD_3OD) δ 7.71 – 7.66 (m, 1H), 6.67 – 6.61 (m, 2H), 6.52 – 6.47 (m, 1H), 5.61 (s$^\Delta$, 1H), 4.68 (s$^\Delta$, 1H), 4.30 – 4.14 (m, 2H), 4.11 – 4.05 (m, 1H), 3.95 (s, 3H), 3.90 – 3.83 (m, 1H), 3.29 – 3.18 (m, 1H), 2.24 – 2.13 (m, 1H), 2.03 – 1.92 (m, 1H), 1.90 – 1.77 (m, 2H). **^{13}C NMR** (126 MHz, CD_3OD) δ 173.8*, 144.2*, 136.57, 134.14, 107.47, 99.64, 74.59, 69.51, 56.94, 42.33, 39.19, 27.83, 20.55. **LCMS** (basic): t_R: 3.51 min, purity: 98.5% (230 nm). **HRMS**: $(M + H)^+$ calcd. for $C_{15}H_{23}N_3O_6$: 224.1394, found: 224.1398.

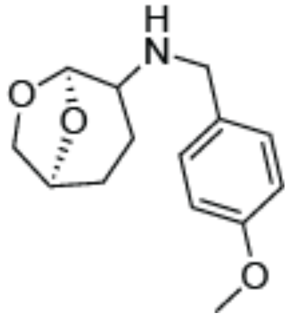

(1S,5R)-N-[(4-methoxyphenyl)methyl]-6,8-dioxabicyclo[3.2.1]octan-4-amine (8g)

General procedure C1a was performed, using Cyrene (212 mg, 1.65 mmol, 1.00 eq), (4-methoxyphenyl)methanamine (227 mg, 1.65 mmol, 1.00 eq), DCM (2.5 mL), $NaBH(OAc)_3$ (456 mg, 2.15 mmol, 1.30 eq) and AcOH (0.11 mL, 2.0 mmol, 1.2 eq), and a reaction time of 1 h and 36 h, respectively. Extraction with 1.0 M aq. NaOH (10 mL) and EtOAc (2×5 mL), followed by a wash of the combined organic layers with H_2O (2×10 mL) and subsequent purification by column chromatography (EtOAc/cHex, 30%) provided the title compound (74 mg, 18%).

Diastereomeric ratio: 2:1. Proposed major diastereomer: *Endo*.

^{1}H NMR (500 MHz, $CDCl_3$) δ 7.28 – 7.22 (m, 2H)^{a,b}, 6.89 – 6.83 (m, 2H)^{a,b}, 5.42 (s$^\Delta$, 0.7H)^a, 5.40 – 5.37 (m, 0.3H)^b, 4.52 – 4.47 (m, 0.7H)^a, 4.47 – 4.43 (m, 0.3H)^b, 3.91 (d$^\Delta$, *J* = 7.1 Hz, 0.3H)^b, 3.85 – 3.70 (m, 3.7H)^{a,b}, 3.79 (s, 1.0H)^b, 3.79 (s, 2.0H)^a, 2.67 (ddd, *J* = 10.8, 5.6, 1.4 Hz, 0.7H)^a, 2.65 – 2.62 (m, 0.3H)^b, 2.05 – 1.99 (m, 0.3H)^b, 1.99 – 1.92 (m, 0.7H)^a, 1.91 – 1.81 (m, 1H)^{a,b}, 1.72 – 1.66 (m, 0.3H)^b, 1.59 – 1.52 (m, 0.7H)^a, 1.46 – 1.35 (m, 1H)^{a,b}. **^{13}C NMR** (126 MHz, $CDCl_3$) δ 158.8^{a,b}, 132.7^b, 132.6^a, 129.3^{a,b}, 113.9^{a,b}, 102.8^b, 102.3^a, 73.4^b, 73.3^a, 68.3^a, 67.1^b, 56.5^a, 55.4^b, 55.4^a, 54.6^{b}50.8^b, 50.0^a, 28.3^a, 25.4^b, 24.5^a, 19.8^b. **LCMS** (acidic): t_R: 2.13 min, purity: 94.0% (230 nm). **HRMS**: $(M + H)^+$ calcd. for $C_{14}H_{19}NO_3$: 250.1438, found: 250.1435.

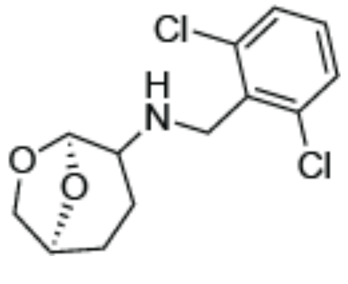

(1S,5R)-N-[(2,6-dichlorophenyl)methyl]-6,8-dioxabicyclo[3.2.1]octan-4-amine (8h and 8i)

General procedure C1a was performed, using Cyrene (256 mg, 2.00 mmol, 1.00 eq), (2,6-dichlorophenyl)methanamine (352 mg, 2.00 mmol, 1.00 eq), DCM (2.5 mL), $NaBH(OAc)_3$ (550 mg, 2.60 mmol, 1.30 eq) and AcOH (0.14 mL, 2.4 mmol, 1.2 eq), and a reaction time of 1 h and 36 h, respectively. Extraction with 1.0 M aq. NaOH (10 mL) and EtOAc (2×5 mL), followed by a wash of the combined organic layers with H_2O (2×10 mL) and subsequent purification by column chromatography (EtOAc/cHex, 25%) provided title compound **8h** (29 mg, 5%) and **8i** (174 mg, 30%).

8h: Diastereomeric ratio: >19:1. Proposed major diastereomer: *Exo*.

^{1}H NMR (500 MHz, $CDCl_3$) δ 7.29 (d, *J* = 8.4 Hz, 1H), 7.29 (d, *J* = 7.8 Hz, 1H), 7.13 (dd, *J* = 8.4, 7.7 Hz, 1H), 5.40 – 5.31 (m, 1H), 4.50 – 4.42 (m, 1H), 4.09 (ABq, 2H, $\Delta\delta_{AB}$= 0.08, J_{AB}= 12.5 Hz), 3.91 (d^{Δ}, *J* = 7.1 Hz, 1H), 3.76 (ddd, *J* = 6.8, 5.0, 1.5 Hz, 1H), 2.76 – 2.70 (m, 1H), 2.05 (ddddd, *J* = 13.6, 13.6, 5.1, 3.2, 1.5 Hz, 1H), 1.89 ($dddd^{\Delta}$, *J* = 13.6, 13.6, 5.4, 5.4 Hz, 1H), 1.68 (m, 1H), 1.39 (dd^{Δ}, *J* = 13.7, 5.7 Hz, 1H). **^{13}C NMR** (126 MHz, $CDCl_3$) δ 136.3, 136.0, 129.1, 128.6, 102.9, 73.4, 67.1, 55.6, 46.9, 25.4, 20.2. **LCMS** (acidic): t_R: 2.28 min, purity: >99% (230 nm). **HRMS**: $(M + H)^+$ calcd. for $C_{13}H_{15}Cl_2NO_2$: 288.0553, found: 288.0549.

8i: Diastereomeric ratio: >19:1. Proposed major diastereomer: *Endo*.

^{1}H NMR (500 MHz, $CDCl_3$) δ 7.28 (d, *J* = 8.4 Hz, 1H), 7.28 (d, *J* = 7.7 Hz, 1H), 7.12 (dd, *J* = 8.4, 7.7 Hz, 1H), 5.42 (s^{Δ}, 1H), 4.53 – 4.45 (m, 1H), 4.10 (ABq, 2H, $\Delta\delta_{AB}$= 0.01, J_{AB}= 12.8 Hz), 3.84 (d^{Δ}, *J* = 7.0 Hz, 1H), 3.78 (ddd, *J* = 6.9, 5.0, 1.4 Hz, 1H), 2.75 (ddd, *J* = 11.0, 5.3, 1.4 Hz, 1H), 1.92 – 1.83 (m, 2H), 1.58 – 1.51 (m, 1H), 1.49 – 1.39 (m, 1H). **^{13}C NMR** (126 MHz, $CDCl_3$) δ 136.3, 136.0, 129.0, 128.6, 102.3, 73.2, 68.4, 57.4, 46.2, 28.3, 24.5. **LCMS** (acidic): t_R: 2.24 min, purity: >99% (230 nm). **HRMS**: $(M + H)^+$ calcd. for $C_{13}H_{15}Cl_2NO_2$: 288.0553, found: 288.0546.

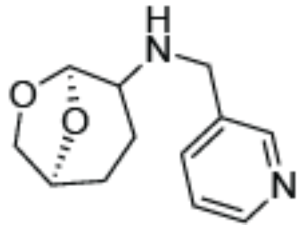

(1S,5R)-N-[(pyridin-3-yl)methyl]-6,8-dioxabicyclo[3.2.1]octan-4-amine (8j)

General procedure C1a was performed, using Cyrene (258 mg, 2.01 mmol, 1.00 eq), pyridin-3-ylmethanamine (218 mg, 2.01 mmol, 1.00 eq), DCM (4.0 mL), $NaBH(OAc)_3$ (555 mg, 2.62 mmol, 1.30 eq) and AcOH (0.14 mL, 2.5 mmol, 1.3 eq), and a reaction time of 3 h and overnight, respectively. Extraction with 1.0 M aq. NaOH (7 mL) and DCM (3×) and subsequent purification by column chromatography (0.35M ammonia in MeOH/EtOAc, 10%) provided the title compound (184 mg, 41%).

Diastereomeric ratio: 3:1. Proposed major diastereomer: *Endo*.

^{1}H NMR (500 MHz, $CDCl_3$) δ 8.68 – 8.46 (m, 2H)^{a,b}, 7.75 – 7.66 (m, 1H)^{a,b}, 7.38 – 7.22 (m, 1H)^{a,b}, 5.43 (s^{Δ}, 0.7H)^{a,b}, 5.41 – 5.37 (m, 0.3H)^{b}, 4.51 – 4.48 (m, 0.7H)^{a}, 4.48 – 4.45 (m, 0.3H)^{b}, 3.93 – 3.75 (m, 4H)^{a,b}, 2.65 (ddd, *J* = 10.7, 5.5, 1.4 Hz, 0.7H)^{a}, 2.62 – 2.59 (m, 0.3H)^{b}, 2.05 – 1.94 (m, 1H)^{a,b}, 1.92 – 1.80 (m, 1H)^{a,b}, 1.73 – 1.67 (m, 0.3H)^{b}, 1.60 – 1.54 (m, 0.7H)^{a}, 1.46 – 1.35 (m, 1H)^{a,b}. **^{13}C NMR** (126 MHz, $CDCl_3$) δ 149.7^{b}, 149.7^{a}, 148.6^{a,b}, 135.9^{b}135.9^{a}, 123.5^{a,b}, 102.7^{b}, 102.2^{a}, 73.4^{b}, 73.2^{a}, 68.3^{a}, 67.1^{b}, 56.5^{a}, 54.8^{b}, 48.6^{b}, 47.9^{a}, 28.2^{a},

25.3[b], 24.5[a], 19.6[b]. **LCMS** (acidic): t_R: 0.74 min, purity: 94.7% (254 nm). **HRMS**: $(M + H)^+$ calcd. for $C_{12}H_{16}N_2O_2$: 221.1285, found: 221.1283.

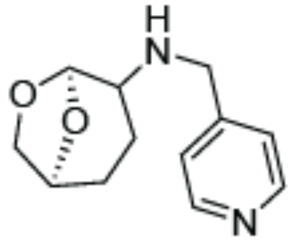

(1S,5R)-N-[(pyridin-4-yl)methyl]-6,8-dioxabicyclo[3.2.1]octan-4-amine (8k)

General procedure C1a was performed, using Cyrene (340 mg, 2.65 mmol, 1.00 eq), pyridin-4-ylmethanamine (287 mg, 2.65 mmol, 1.00 eq), DCM (5.0 mL), $NaBH(OAc)_3$ (731 mg, 3.45 mmol, 1.30 eq) and AcOH (0.20 mL, 3.4 mmol, 1.3 eq), and a reaction time of 1.5 h and overnight, respectively. Extraction with 1.0 M aq. NaOH and DCM (3×) and subsequent purification by column chromatography (0.35M ammonia in MeOH/EtOAc, 0–15%) provided the title compound (505 mg, 86%).

Diastereomeric ratio: 3:1. Proposed major diastereomer: *Endo*.

^{1}H NMR (500 MHz, $CDCl_3$) δ 8.63 – 8.51 (m, 2H)[a,b], 7.36 – 7.32 (m, 0.5H)[b], 7.32 – 7.30 (m, 1.5H)[a], 5.46 (s[Δ], 0.8H)[a], 5.45 – 5.43 (m, 0.2H)[b], 4.56 – 4.52 (m, 0.8H)[a], 4.52 – 4.50 (m, 0.2H)[b], 3.98 – 3.79 (m, 4H)[a,b], 2.67 (ddd, *J* = 10.7, 5.5, 1.4 Hz, 0.8H)[a], 2.64 – 2.62 (m, 0.2H)[b], 2.09 – 1.97 (m, 1H)[a,b], 1.97 – 1.85 (m, 1H)[a,b], 1.76 – 1.56 (m, 1H)[a,b], 1.51 – 1.41 (m, 1H)[a,b]. **^{13}C NMR** (126 MHz, $CDCl_3$) δ 150.0[a,b], 149.7[a,b], 123.0[b], 123.0[a], 102.7[b], 102.2[a], 73.4[b], 73.3[a], 68.4[a], 67.1[b], 56.7[a], 54.9[b], 50.0[b], 49.3[a], 28.2[a], 25.3[b], 24.5[a], 19.7[b]. **LCMS** (acidic): t_R: 0.74 min, purity: 95.8% (230 nm). **HRMS**: $(M + H)^+$ calcd. for $C_{12}H_{16}N_2O_2$: 221.1285, found: 221.1280.

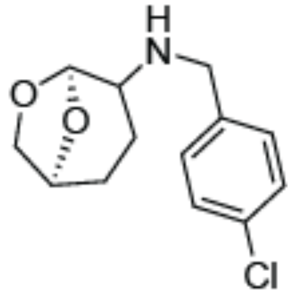

(1S,5R)-N-[(4-chlorophenyl)methyl]-6,8-dioxabicyclo[3.2.1]octan-4-amine (8l)

General procedure C1b was performed, using Cyrene (260 mg, 2.03 mmol, 1.00 eq), 4-chlorobenzaldehyde (341 mg, 2.42 mmol, 1.19 eq), DCE (20.0 mL), $NaBH(OAc)_3$ (1381 mg, 6.52 mmol, 3.21 eq), and a reaction time of 5 h and overnight, respectively. Diluting with DCM (20 mL) and washing with 2.0 M aq. NaOH (2×20 mL) and brine (2×20 mL), followed by purification by column chromatography (EtOAc/cHex, 35%) provided the title compound (123 mg, 24%).

Diastereomeric ratio: 1.6:1.0. Proposed major diastereomer: *Exo*.

^{1}H NMR (500 MHz, $CDCl_3$) δ 7.31 – 7.26 (m, 4H)^{a,b}, 5.41 (s$^{\Delta}$, 0.4H)^{b}, 5.40 – 5.36 (m, 0.6H)^{a}, 4.52 – 4.48 (m, 0.4H)^{b}, 4.48 – 4.44 (m, 0.6H)^{a}, 3.91 (d$^{\Delta}$, *J* = 7.0 Hz, 1H)^{a}, 3.88 – 3.72 (m, 3.4H)^{a,b}, 2.64 (ddd, *J* = 10.9, 5.5, 1.4 Hz, 0.4H)^{b}, 2.62 – 2.59 (m, 0.6H)^{a}, 2.04 – 1.92 (m, 1H)^{a,b}, 1.92 – 1.81 (m, 1H)^{a,b}, 1.72 – 1.64 (m, 0.6H)^{a}, 1.57 – 1.53 (m, 0.4H)^{b}, 1.46 – 1.36 (m, 1H)^{a,b}. **^{13}C NMR** (126 MHz, $CDCl_3$) δ 139.1^{a}, 139.0^{b}, 132.7^{b}, 132.7^{a}, 129.5^{a,b}, 128.6^{b}, 128.6^{a}, 102.8^{a}, 102.2^{b}, 73.4^{a}, 73.2^{b}, 68.3^{b}, 67.1^{a}, 56.5^{b}, 54.7^{a}, 50.6^{a}, 49.9^{b}, 28.3^{b}, 25.3^{a}, 24.5^{b}, 19.8^{a}. **LCMS** (acidic): t_R: 2.25 min, purity: 96.5% (254 nm). **HRMS**: $(M + H)^+$ calcd. for $C_{13}H_{16}ClNO_2$: 254.0942, found: 254.0936.

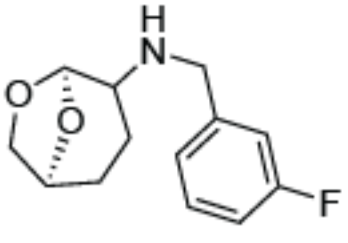

(1S,5R)-N-[(3-fluorophenyl)methyl]-6,8-dioxabicyclo[3.2.1]octan-4-amine (8m)

General procedure C1b was performed, using Cyrene (135 mg, 1.05 mmol, 1.00 eq), 3-fluorobenzaldehyde (157 mg, 1.26 mmol, 1.20 eq), DCE (10.0 mL), $NaBH(OAc)_3$ (804 mg, 3.79 mmol, 3.60 eq), and a reaction time of 1 h and 2 d, respectively. Diluting with DCM (12 mL) and washing with 2.0 M aq. NaOH (12 mL) and brine (12 mL), followed by purification by column chromatography (EtOAc/cHex, 17%) provided the title compound (24 mg, 10%).

Diastereomeric ratio: 1.2:1.0.

^{1}H NMR (500 MHz, CD_3OD) δ 7.36 – 7.28 (m, 1H)^{a,b}, 7.18 – 7.09 (m, 2H)^{a,b}, 7.01 – 6.93 (m, 1H)^{a,b}, 5.35 (s$^{\Delta}$, 0.5H)^{b}, 5.34 – 5.29 (m, 0.5H)^{a}, 4.49 – 4.44 (m, 1H)^{a,b}, 3.93 (d$^{\Delta}$, *J* = 7.1 Hz, 0.5H)^{a}, 3.90 – 3.74 (m, 2.5H)^{a,b}, 3.74 – 3.67 (m, 1H)^{a,b}, 2.60 (ddd, *J* = 10.8, 5.6, 1.5 Hz, 0.5H)^{b}, 2.56 – 2.50 (m, 0.5H)^{a}, 2.05 (ddddd, *J* = 13.7, 13.7, 5.3, 3.3, 1.5 Hz, 0.5H)^{a}, 1.98 – 1.89 (m, 0.5H)^{b}, 1.89 – 1.83 (m, 0.5H)^{a}, 1.83 – 1.75 (m, 0.5H)^{b}, 1.73 – 1.64 (m, 0.5H)^{a}, 1.59 (dd$^{\Delta}$, *J* = 13.8, 5.9 Hz, 0.5H)^{b}, 1.55 – 1.44 (m, 0.5H)^{b}, 1.42 (dd$^{\Delta}$, *J* = 14.0, 5.9 Hz, 0.5H)^{a}. **^{13}C NMR** (126 MHz, CD_3OD) δ 164.4 (d, *J* = 244.6 Hz)$^{a/b}$, 164.4 (d, *J* = 244.3 Hz)$^{a/b}$, 145.0 – 143.5 (m)^{a,b}, 131.3 – 130.8 (m)^{a,b}, 125.2 – 125.1 (m)^{ab}, 116.0 (d, *J* = 21.7 Hz)$^{a/b}$, 116.0 (d, *J* = 21.5 Hz)$^{a/b}$, 114.9 – 114.6 (m)^{a,b}, 103.4^{a}, 103.1^{b}, 74.7^{b}, 74.5^{a}, 69.1^{b}, 68.0^{a}, 57.7^{b}, 55.8^{a}, 51.3 (d, *J* = 1.8 Hz)^{a}, 50.5 (d, *J* = 1.8 Hz)^{b}, 29.0^{b}, 25.8^{a}, 24.5^{b}, 20.2^{a}. **LCMS** (acidic): t_R: 1.83 min, purity: 93.8% (254 nm). **HRMS**: $(M + H)^+$ calcd. for $C_{13}H_{16}FNO_2$: 238.1238, found: 238.1232.

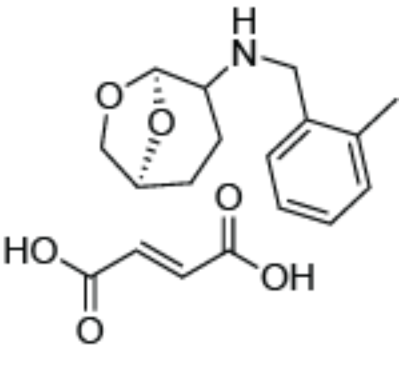

(1S,5R)-N-[(2-methylphenyl)methyl]-6,8-dioxabicyclo[3.2.1]octan-4-amine (8n)

General procedure C2b was performed, using amine **4** (268 mg, 2.07 mmol, 1.00 eq), 2-methylbenzaldehyde (249 mg, 2.07 mmol, 1.00 eq), DCM (2.5 mL), $NaBH_4$ (78 mg, 2.1 mmol, 1.0 eq) and $MgSO_4$ (250 mg, 2.07 mmol, 1.00 eq). Purification by column chromatography (MeOH/DCM + 1% Et_3N, 0–10%) and salt formation provided the title compound as a fumaric acid salt (108 mg, 15%; ratio base:fumaric acid 1:1).

Diastereomeric ratio: 1.0:1.0.

^{1}H NMR (500 MHz, CD_3OD) δ 7.43 – 7.36 (m, 1H)^{a,b}, 7.32 – 7.20 (m, 3H)^{a,b}, 6.65 (m, 2H)^{a,b}, 5.63 (s$^{\Delta}$, 0.5H)$^{a/b}$, 5.63 – 5.61 (m, 0.5H)$^{a/b}$, 4.62 – 4.55 (m, 1H)^{a,b}, 4.24 (ABq, 1.0H, $\Delta\delta_{AB}$= 0.02, J_{AB}= 13.5 Hz)$^{a/b}$, 4.18 (ABq, 1.0H, $\Delta\delta_{AB}$= 0.03, J_{AB}= 13.3 Hz)$^{a/b}$, 4.02 (d$^{\Delta}$, *J* = 7.4 Hz, 0.5H)$^{a/b}$, 3.98 (d$^{\Delta}$, *J* = 7.4 Hz, 0.5H)$^{a/b}$, 3.82 – 3.75 (m, 1H)^{a,b}, 3.31 (ddd, *J* = 10.5, 5.8, 1.4 Hz, 0.5H)$^{a/b}$, 3.18 (d$^{\Delta}$, *J* = 5.0 Hz, 0.5H)$^{a/b}$, 2.40 (s, 1.5H)$^{a/b}$, 2.39 (s, 1.5H)$^{a/b}$, 2.28 – 2.07 (m, 1.5H)^{a,b}, 1.99 – 1.85 (m, 1.0H)^{a,b}, 1.84 – 1.76 (m, 0.5H)$^{a/b}$, 1.76 – 1.69 (m, 0.5H)$^{a/b}$, 1.52 (dd$^{\Delta}$, *J* = 14.0, 5.1 Hz, 0.5H)$^{a/b}$. **^{13}C NMR** (126 MHz, CD_3OD) δ 171.1^{a,b}, 139.0$^{a/b}$, 138.8$^{a/b}$, 136.1^{a,b}, 132.1$^{a/b}$, 132.1$^{a/b}$, 131.7^{a,b}, 131.6$^{a/b}$, 131.4$^{a/b}$, 130.7$^{a/b}$, 130.7$^{a/b}$, 127.7$^{a/b}$, 99.8$^{a/b}$, 99.7$^{a/b}$, 75.3$^{a/b}$, 74.6$^{a/b}$, 69.6$^{a/b}$, 68.9$^{a/b}$, 58.6$^{a/b}$, 56.7$^{a/b}$, 48.5$^{a/b}$, 47.2$^{a/b}$, 28.3$^{a/b}$, 25.0$^{a/b}$, 21.2$^{a/b}$, 19.3$^{a/b}$, 19.2$^{a/b}$, 19.0$^{a/b}$. **LCMS** (basic): t_R: 5.16 min, purity: 97.2% (254 nm). **HRMS**: $(M + H)^+$ calcd. for $C_{18}H_{25}NO_6$: 234.1489, found: 234.1480.

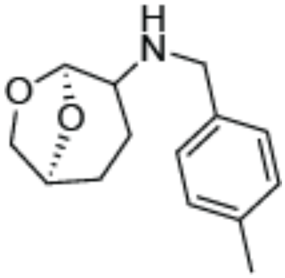

(1S,5R)-N-[(4-methylphenyl)methyl]-6,8-dioxabicyclo[3.2.1]octan-4-amine (8o)

General procedure C2b was performed, using amine **4** (268 mg, 2.07 mmol, 1.00 eq), 4-methylbenzaldehyde (249 mg, 2.07 mmol, 1.00 eq), DCM (2.5 mL), $NaBH_4$ (78 mg, 2.1 mmol, 1.0 eq) and $MgSO_4$ (250 mg, 2.07 mmol, 1.00 eq). Purification by column chromatography (MeOH/DCM + 1% Et_3N, 0–10%) provided the title compound (160 mg, 33%).

Diastereomeric ratio: 2:1. Proposed major diastereomer: *Exo*.

^{1}H NMR (500 MHz, $CDCl_3$) δ 7.25 – 7.20 (m, 2H)^{a,b}, 7.15 – 7.09 (m, 2H)^{a,b}, 5.43 (s$^{\Delta}$, 0.7H)^{a}, 5.42 – 5.38 (m, 0.3H)^{b}, 4.53 – 4.44 (m, 1H)^{a,b}, 3.91 (d$^{\Delta}$, *J* = 7.1 Hz, 0.3H)^{b}, 3.88 – 3.74 (m, 3.7H)^{a,b}, 2.68 (ddd, *J* = 10.8, 5.5, 1.3 Hz, 0.7H)^{a}, 2.65 (d$^{\Delta}$, *J* = 5.1 Hz, 0.3H)^{a,b}, 2.40 – 2.27 (m, 3H)^{a,b}, 2.08 – 1.99 (m, 0.3H)^{b}, 1.99 – 1.92 (m, 0.7H)^{a}, 1.91 – 1.81 (m, 1H)^{a,b}, 1.73 – 1.66 (m, 0.3H)^{b}, 1.55 (dd$^{\Delta}$, *J* = 13.9, 6.0 Hz, 0.7H)^{a}, 1.48 – 1.36 (m, 1H)^{a,b}. **^{13}C NMR** (126 MHz, $CDCl_3$) δ 137.2^{a,b}, 136.8^{b}, 136.7^{a}, 129.2^{a,b}, 128.2^{b}, 128.2^{a}, 102.7^{b}, 102.2^{a}, 73.4^{a}, 73.2^{b}, 68.3^{a}, 67.1^{b}, 56.4^{a}, 54.6^{b}, 51.0^{b}, 50.3^{a}, 28.3^{a}, 25.3^{b}, 24.3^{b}, 21.2^{a,b}, 19.7^{b}. **LCMS** (basic): t_R: 5.08 min, purity: 95.8% (254 nm). **HRMS**: $(M + H)^+$ calcd. for $C_{14}H_{19}NO_2$: 234.1489, found: 234.1482.

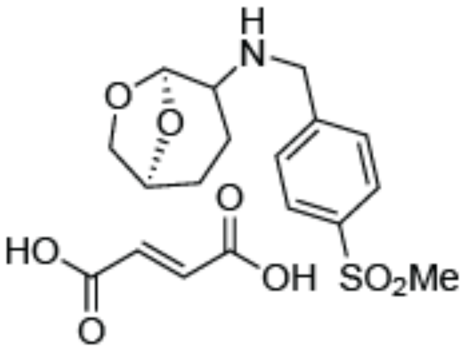

(1S,5R)-N-[(4-methanesulfonylphenyl)methyl]-6,8-dioxabicyclo[3.2.1]octan-4-amine (8p)

General procedure C2b was performed, using amine **4** (268 mg, 2.07 mmol, 1.00 eq), 4-methylsulfonylbenzaldehyde (382 mg, 2.07 mmol, 1.00 eq), DCM (2.5 mL), $NaBH_4$ (78 mg, 2.1 mmol, 1.0 eq) and $MgSO_4$ (250 mg, 2.07 mmol, 1.00 eq). Purification by column chromatography (MeOH/DCM + 1% Et_3N, 0–10%) and salt formation provided the title compound as a fumaric acid salt (210 mg, 28%; ratio base:fumaric acid 2:1).

Diastereomeric ratio: 1.1:1.0.

^{1}H NMR (500 MHz, CD_3OD) δ 8.01 – 7.95 (m, 2H)[a,b], 7.78 – 7.70 (m, 2H)[a,b], 6.54 – 6.49 (m, 2H)[a,b], 5.62 – 5.56 (m, 1H)[a,b], 4.64 (m, 1H)[a,b], 4.39 – 4.19 (m, 2H)[a,b], 4.04 (d[Δ], *J* = 7.5 Hz, 0.5H)[a/b], 4.01 (d[Δ], *J* = 7.5 Hz, 1H)[a,b], 3.79 (m, 1H)[a,b], 3.25 – 3.15 (m, 3.5H)[a,b], 3.15 – 3.06 (m, 0.5H)[a/b], 2.20 – 2.05 (m, 1.5H)[a,b], 1.97 – 1.83 (m, 1H)[a,b], 1.83 – 1.70 (m, 1H)[a,b], 1.56 (d[Δ], *J* = 8.5 Hz, 0.5H)[a/b]. **^{13}C NMR** (126 MHz, CD_3OD) δ 174.01*[,a,b], 141.4[a/b], 141.3[a/b], 139.9*[,a,b]136.4[a,b], 131.9[b], 131.8[a], 128.9[a,b], 99.6[a,b], 75.2[b], 74.6[a], 69.5[a], 68.7[b], 57.9[a], 55.9[b], 50.0[a/b], 48.9[a/b], 44.2[a,b], 27.8[a/b], 24.7[a/b], 20.9[a/b], 18.4[a/b]. **LCMS** (basic): t_R: 4.08 min, purity: >99% (254 nm). **HRMS**: $(M + H)^+$ calcd. for $C_{18}H_{25}NO_8S$: 298.1108, found: 298.1122.

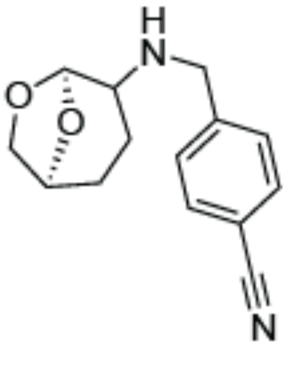

4-({[(1S,5R)-6,8-dioxabicyclo[3.2.1]octan-4-yl]amino}methyl)benzonitrile (8q and 8r)

General procedure C1b was performed, using Cyrene (144 mg, 1.12 mmol, 1.00 eq), 4-formylbenzonitrile (177 mg, 1.35 mmol, 1.20 eq), DCE (10.0 mL), $NaBH(OAc)_3$ (858 mg, 4.05 mmol, 3.60 eq), and a reaction time of 1 hr and 2 d, respectively. Diluting with DCM (20 mL) and washing with 2.0 M aq. NaOH (40 mL) and brine (60 mL), followed by purification by column chromatography (EtOAc/cHex, 25%) provided title compound **8q** (41 mg, 15%) and **8r** (42 mg, 15%).

8q: Diastereomeric ratio: 9:1. Proposed major diastereomer: *Exo*.

^{1}H NMR (500 MHz, $CDCl_3$) δ 7.64 – 7.58 (m, 2H), 7.53 – 7.46 (m, 2H), 5.41 (s^{Δ}, 1H), 4.50 – 4.45 (m, 1H), 4.01 – 3.85 (m, 3H), 3.78 (ddd, *J* = 6.9, 5.1, 1.5 Hz, 1H), 2.62 – 2.58 (m, 1H), 2.09 – 2.00 (m, 1H), 1.89 ($dddd^{\Delta}$, *J* = 13.7, 13.7, 5.5, 5.5 Hz, 1H), 1.73 – 1.66 (m, 1H), 1.43 (dd^{Δ}, *J* = 14.1, 5.6 Hz, 1H). **^{13}C NMR** (126 MHz, $CDCl_3$) δ 145.6*, 132.4, 128.8, 119.1, 111.0, 102.4, 73.4, 67.2, 54.8, 50.6, 25.2, 19.6. **LCMS** (acidic): t_R: 1.87 min, purity: 98.7% (230 nm). **HRMS**: $(M + H)^+$ calcd. for $C_{14}H_{16}N_2O_2$: 245.1285, found: 245.1279.

8r: Diastereomeric ratio: 10:1. Proposed major diastereomer: *Endo*.

^{1}H NMR (500 MHz, $CDCl_3$) δ 7.63 – 7.58 (m, 2H), 7.48 – 7.44 (m, 2H), 5.42 (s^{Δ}, 1H), 4.53 – 4.48 (m, 1H), 3.90 (m, 2H), 3.84 (d^{Δ}, *J* = 7.0 Hz, 1H), 3.79 (ddd, *J* = 6.9, 5.0, 1.5 Hz, 1H), 2.63 (ddd, *J* = 10.8, 5.6, 1.4 Hz, 1H), 2.02 – 1.93 (m, 1H), 1.86 (ddddd, *J* = 14.1, 12.9, 5.4, 3.2, 1.4 Hz, 1H), 1.58 (dd, *J* = 14.1, 6.1 Hz, 1H), 1.42 ($dddd^{\Delta}$, *J* = 13.0, 13.0, 10.7, 5.9 Hz, 1H). **^{13}C NMR** (126 MHz, $CDCl_3$) δ 146.3, 132.4, 128.7, 119.1, 110.9, 102.1, 73.2, 68.4, 56.7, 50.1, 28.2, 24.5. **LCMS** (acidic): t_R: 1.86 min, purity: 98.9% (254 nm). **HRMS**: $(M + H)^+$ calcd. for $C_{14}H_{16}N_2O_2$: 245.1285, found: 245.1276.

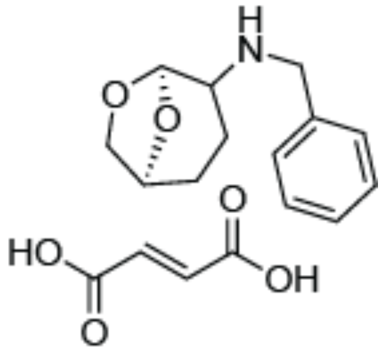

(1S,5R)-N-benzyl-6,8-dioxabicyclo[3.2.1]octan-4-amine (8s)

General procedure C2b was performed, using amine **4** (261 mg, 2.02 mmol, 1.00 eq), benzaldehyde (214 mg, 2.02 mmol, 1.00 eq), DCM (2.5 mL), $NaBH_4$ (76 mg, 2.0 mmol, 1.0 eq) and $MgSO_4$ (243 mg, 2.02 mmol, 1.00 eq). Purification by column chromatography (MeOH/DCM + 1% Et_3N, 0–10%) and salt formation provided the title compound as a fumaric acid salt (75 mg, 11%; ratio base:fumaric acid 1:1).

Diastereomeric ratio: 1.2:1.0.

The compound has previously been reported as the separated *exo* (free base, salt) and *endo* isomer (salt)[7].

^{1}H NMR (500 MHz, CD_3OD) δ 7.50 – 7.38 (m, 5H)^{a,b}, 6.67 – 6.63 (m, 2H)^{a,b}, 5.55 (s^{Δ}, 0.6H)^{a}, 5.54 – 5.52 (m, 0.4H)^{b}, 4.61 – 4.57 (m, 0.4H)^{b}, 4.57 – 4.53 (m, 0.6H)^{a}, 4.23 (ABq, 0.9H, $\Delta\delta_{AB}$= 0.03, J_{AB}= 13.2 Hz)^{b}, 4.16 (ABq, 1.1H, $\Delta\delta_{AB}$= 0.04, J_{AB}= 13.1 Hz)^{a}, 4.00 (dd^{Δ}, *J* = 7.3, 0.7 Hz, 0.4H)^{b}, 3.97 (dd^{Δ}, *J* = 7.4, 0.7 Hz, 0.6H)^{a}, 3.81 – 3.73 (m, 1H)^{a,b}, 3.20 (ddd, *J* = 10.8, 5.8, 1.5 Hz, 0.6H)^{a}, 3.10 – 3.05 (m, 0.4H)^{b}, 2.20 – 2.05 (m, 1.5H)^{a,b}, 1.96 – 1.86 (m, 0.6H)^{a}, 1.86 – 1.82 (m, 0.4H)^{b}, 1.82 – 1.75 (m, 0.6H)^{a}, 1.75 – 1.68 (m, 0.6H)^{a}, 1.59 – 1.46 (m, 0.4H)^{b}. **^{13}C NMR** (126 MHz, CD_3OD) δ 171.2^{a,b}, 136.2^{a,b}, 133.2$^{a/b}$, 133.1$^{a/b}$, 131.1^{b}, 130.9^{a}, 130.6^{b}, 130.5^{a}, 130.3^{b}, 130.3^{a}, 99.9^{a}, 99.7^{b}, 75.2^{b}, 74.6^{a}, 69.6^{a}, 68.9^{b}, 58.0^{a}, 56.0^{b}, 50.9^{b}, 49.8^{a}, 28.2^{a}, 25.0^{b}, 21.0^{a}, 18.6^{b}. **LCMS** (basic): t_R: 4.70 min, purity: >99% (254 nm). **HRMS**: $(M + H)^+$ calcd. for $C_{17}H_{23}NO_6$: 220.1332, found: 220.1342.

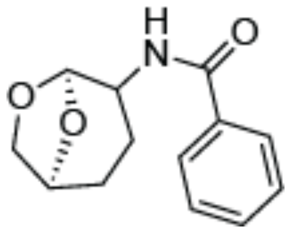

N-[(1S,5R)-6,8-dioxabicyclo[3.2.1]octan-4-yl]benzamide (9a and 9b)

General procedure D1 was performed, using amine **4** (263 mg, 2.04 mmol, 1.00 eq), benzoic acid (302 mg, 2.47 mmol, 1.21 eq), DCM (10.0 mL), EDCI·HCl (471 mg, 2.46 mmol, 1.21 eq), DMAP (154 mg, 1.26 mmol, 0.619 eq) and DIPEA (0.22 mL, 1.3 mmol, 0.62 eq), and a reaction time of overnight. Extraction with H_2O (40 mL) and DCM (3×30 mL) and subsequent purification by column chromatography (EtOAc/cHex + 1% Et_3N, 0–50%) provided title compound **9a** (92 mg, 19%) and **9b** (62 mg, 13%).

9a: Diastereomeric ratio: >19:1. Proposed major diastereomer: *Exo*.

^{1}H NMR (500 MHz, $CDCl_3$) δ 7.80 (m, 2H), 7.55 – 7.48 (m, 1H), 7.48 – 7.41 (m, 2H), 6.59 (d, *J* = 8.2 Hz, 1H), 5.42 – 5.37 (m, 1H), 4.59 – 4.53 (m, 1H), 4.25 – 4.18 (m, 1H), 4.01 (d^{Δ} , *J* = 7.2 Hz, 1H), 3.86 (ddd, *J* = 6.8, 5.1, 1.4 Hz, 1H), 2.14 ($dddd^{\Delta}$, *J* = 13.9, 13.9, 5.4, 5.4 Hz, 1H), 2.02 (ddddd, *J* = 15.1, 8.5, 3.6, 1.8, 1.6, 1.6 Hz, 1H), 1.75 – 1.67 (m, 1H), 1.53 (dd^{Δ} , *J* = 14.3, 5.1 Hz, 1H). **^{13}C NMR** (126 MHz, $CDCl_3$) δ 166.9, 134.6, 131.8, 128.7, 127.1, 101.6, 73.5, 67.6, 47.6, 25.6, 21.5. **LCMS** (acidic): t_R: 3.06 min, purity: >99% (254 nm). **HRMS**: $(M + H)^+$ calcd. for $C_{13}H_{15}NO_3$: 234.1125, found: 234.1128.

9b: Diastereomeric ratio: >19:1. Proposed major diastereomer: *Endo*.

^{1}H NMR (500 MHz, $CDCl_3$) δ 7.79 – 7.74 (m, 2H), 7.53 – 7.47 (m, 1H), 7.46 – 7.40 (m, 2H), 6.07 (d, *J* = 8.3 Hz, 1H), 5.41 (s^{Δ}, 1H), 4.62 – 4.53 (m, 1H), 4.29 (dddd, *J* = 10.7, 9.1, 5.9, 1.4 Hz, 1H), 3.91 (d^{Δ}, *J* = 7.1 Hz, 1H), 3.88 – 3.80 (m, 1H), 2.12 – 2.05 (m, 1H), 2.05 – 1.97 (m, 1H), 1.67 – 1.62 (m, 1H), 1.61 – 1.55 (m, 1H). **^{13}C NMR** (126 MHz, $CDCl_3$) δ 167.0, 134.4, 131.8, 128.7, 127.1, 102.2, 73.2, 68.3, 49.4, 27.9, 23.6. **LCMS** (acidic): t_R: 3.00 min, purity: >99% (254 nm). **HRMS**: $(M + H)^+$ calcd. for $C_{13}H_{15}NO_3$: 234.1125, found: 234.1136.

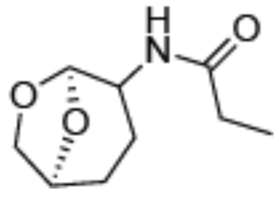

N-[(1S,5R)-6,8-dioxabicyclo[3.2.1]octan-4-yl]propanamide (9c)

General procedure D1 was performed, using amine **4** (262 mg, 2.03 mmol, 1.00 eq), propanoic acid (180 mg, 2.43 mmol, 1.20 eq), DCM (10.0 mL), EDCI·HCl (502 mg, 2.62 mmol, 1.29 eq), DMAP (181 mg, 1.48 mmol, 0.730 eq) and DIPEA (1.1 mL, 6.3 mmol, 3.1 eq), and a reaction time of overnight. Extraction with H_2O (30 mL) and DCM (3×30 mL), followed by a wash of the combined organic layers with satd. aq. Na_2CO_3 (3×20 mL) and subsequent purification by column chromatography (EtOAc/cHex + 1% Et_3N, 30–100%) provided the title compound (183 mg, 49%).

Diastereomeric ratio: 1.3:1.0. Proposed major diastereomer: *Endo*.

^{1}**H NMR** (500 MHz, $CDCl_3$) δ 5.96 (d, *J* = 6.0 Hz, 0.4H)^{b}, 5.49 (d, *J* = 6.7 Hz, 0.6H)^{a}, 5.27 (s$^{\Delta}$, 0.6H)^{a}, 5.26 – 5.24 (m, 0.4H)^{b}, 4.55 – 4.50 (m, 0.6H)^{a}, 4.50 – 4.46 (m, 0.4H)^{b}, 4.09 – 4.01 (m, 0.6H)^{a}, 4.01 – 3.96 (m, 0.4H)^{b}, 3.94 (d$^{\Delta}$, *J* = 7.2 Hz, 0.4H)^{b}, 3.84 (d$^{\Delta}$, *J* = 7.0 Hz, 0.6H)^{a}, 3.82 – 3.76 (m, 1H)^{a,b}, 2.26 – 2.14 (m, 2H)^{a,b}, 2.09 – 1.99 (m, 0.4H)^{b}, 1.98 – 1.88 (m, 1.6H)^{a,b}, 1.61 – 1.51 (m, 1H)^{a,b}, 1.51 – 1.40 (m, 1H)^{a,b}, 1.15 (dd, *J* = 7.6, 7.6 Hz, 1.3H)^{b}, 1.12 (dd, *J* = 7.6, 7.6 Hz, 1.7H)^{a}. ^{13}**C NMR** (126 MHz, $CDCl_3$) δ 173.4^{a}, 173.3^{b}, 102.1^{a}, 101.5^{b}, 73.3^{b}, 73.0^{a}, 68.2^{a}, 67.4^{b}, 48.8^{a}, 47.0^{b}, 29.9^{b}, 29.8^{a}, 27.8^{a}, 25.4^{b}, 23.4^{a}, 21.4^{b}, 9.9^{b}, 9.9^{a}. **LCMS**: No UV absorption at 230/254 nm. **HRMS**: $(M + H)^+$ calcd. for $C_9H_{15}NO_3$: 186.1125, found: 186.1122.

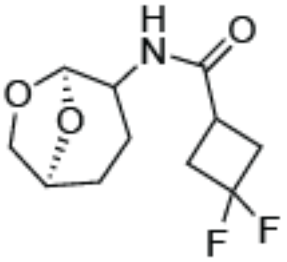

N-[(1S,5R)-6,8-dioxabicyclo[3.2.1]octan-4-yl]-3,3-difluorocyclobutane-1-carboxamide (9d)

General procedure D1 was performed, using amine **4** (261 mg, 2.02 mmol, 1.00 eq), 3,3-difluorocyclobutane-1-carboxylic acid (333 mg, 2.45 mmol, 1.21 eq), DCM (10.0 mL), EDCI·HCl (495 mg, 2.58 mmol, 1.28 eq), DMAP (207 mg, 1.69 mmol, 0.838 eq) and DIPEA (1.1 mL, 6.5 mmol, 3.2 eq), and a reaction time of overnight. Extraction with H_2O (40 mL) and DCM (3×30 mL), followed by a wash of the combined organic layers with satd. aq. NH_4Cl (30 mL) and subsequent purification by column chromatography (EtOAc/cHex + 1% Et_3N, 30–100%) provided the title compound (297 mg, 59%).

Diastereomeric ratio: 1.2:1.0. Proposed major diastereomer: *Endo*.

^{1}**H NMR** (600 MHz, $CDCl_3$) δ 5.96 (d, *J* = 7.6 Hz, 0.5H)^{b}, 5.49 (d, *J* = 7.8 Hz, 0.5H)^{a}, 5.27 (s$^{\Delta}$, 0.5H)^{a}, 5.26 – 5.24 (m, 0.5H)^{b}, 4.56 – 4.52 (m, 0.5H)^{a}, 4.52 – 4.48 (m, 0.5H)^{b}, 4.09 – 4.02 (m, 0.5H)^{a}, 4.00 (dddd, *J* = 9.2, 4.6, 2.3, 2.3 Hz, 0.5H)^{b}, 3.96 (d$^{\Delta}$, *J* = 7.2 Hz, 0.5H)^{b}, 3.85 (d$^{\Delta}$, *J* = 7.1 Hz, 0.5H)^{a}, 3.83 – 3.76 (m, 1H)^{a,b}, 2.93 – 2.81 (m, 2H)^{a,b}, 2.81 – 2.63 (m, 3H)^{a,b}, 2.07 (dddd$^{\Delta}$, *J* = 14.0, 14.0, 5.5, 5.5 Hz, 0.5H)^{b}, 2.00 – 1.88 (m, 1.5H)^{a,b}, 1.59 (dd$^{\Delta}$, *J* = 12.8, 5.6 Hz, 0.5H)^{a}, 1.57 – 1.52 (m, 0.5H)^{b}, 1.52 – 1.41 (m, 1H)^{a,b}. ^{13}**C NMR** (151 MHz, $CDCl_3$) δ 171.7 (dd, *J* = 2.5, 2.5 Hz)^{a}, 171.6 (dd, *J* = 2.2, 2.2 Hz)^{b}, 118.9 (dd, *J* = 286.1, 268.4 Hz)^{b}, 118.8 (dd, *J* = 286.4, 268.1 Hz)^{a}, 101.9^{a}, 101.3^{b}, 73.4^{b}, 73.1^{a}, 68.3^{a}, 67.5^{b}, 49.2^{a}, 47.4^{b}, 39.1 – 38.6 (m)^{a,b}, 28.5 – 28.2 (m)^{a,b}, 27.8^{a}, 25.4^{b}, 23.4^{a}, 21.4^{b}. **LCMS**: No UV absorption at 230/254 nm. **HRMS** calcd. for $C_{11}H_{15}F_2NO_3$: 248.1093, found: 248.1101.

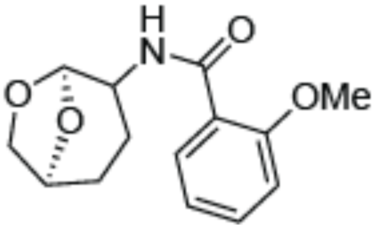

N-[(1S,5R)-6,8-dioxabicyclo[3.2.1]octan-4-yl]-2-methoxybenzamide (9e)

General procedure D1 was performed, using amine **4** (267 mg, 2.07 mmol, 1.00 eq), 2-methoxybenzoic acid (391 mg, 2.57 mmol, 1.24 eq), DCM (10.0 mL), EDCI·HCl (486 mg, 2.53 mmol, 1.23 eq), DMAP (153 mg, 1.25 mmol, 0.606 eq) and DIPEA (1.2 mL, 6.6 mmol, 3.2 eq), and a reaction time of 2 d. Extraction with H_2O (40 mL) and DCM (3×30 mL), followed by a wash of the combined organic layers with satd. aq. NH_4Cl (30 mL) and subsequent purification by column chromatography (EtOAc/cHex + 1% Et_3N, 30–100%) provided the title compound (195 mg, 36%).

Diastereomeric ratio: 1.1:1.0.

^{1}H NMR (500 MHz, $CDCl_3$) δ 8.43 (d, *J* = 7.9 Hz, 0.5H)[a], 8.22 – 8.14 (m, 1H)[a,b], 7.87 (d, *J* = 8.1 Hz, 0.5H)[b], 7.49 – 7.40 (m, 1H)[a,b], 7.12 – 7.03 (m, 1H)[a,b], 7.03 – 6.93 (m, 1H)[a,b], 5.42 (s^Δ, 0.5H)[b], 5.41 – 5.37 (m, 0.5H)[a], 4.60 – 4.53 (m, 1H)[a,b], 4.34 – 4.26 (m, 0.5H)[b], 4.26 – 4.20 (m, 0.5H)[a], 4.01 (d^Δ, *J* = 6.9 Hz, 0.5H)[a/b], 4.01 (s, 1.5H)[a/b], 3.97 (s, 1.5H)[a/b], 3.93 (d^Δ, *J* = 7.1 Hz, 0.5H)[a/b], 3.88 – 3.81 (m, 1H)[a,b], 2.18 – 2.06 (m, 1H)[a,b], 2.06 – 1.96 (m, 1H)[a,b], 1.74 – 1.67 (m, 0.5H)[a/b], 1.67 – 1.56 (m, 1H)[a,b], 1.53 (dd^Δ , *J* = 13.8, 5.2 Hz, 0.5H)[a/b]. **^{13}C NMR** (126 MHz, $CDCl_3$) δ 164.8, 164.6, 157.6, 157.6, 132.9, 132.8, 132.3, 132.3, 121.6, 121.4, 121.4, 121.3, 111.4, 111.4, 102.2, 101.7, 73.3, 73.1, 68.3, 67.4, 56.1, 56.0, 49.3, 47.5, 27.9, 25.7, 23.5, 21.6. **LCMS** (acidic): t_R: 3.37 min, purity: >99% (254 nm). **HRMS**: $(M + H)^+$ calcd. for $C_{14}H_{17}NO_4$: 264.1230, found: 264.1241.

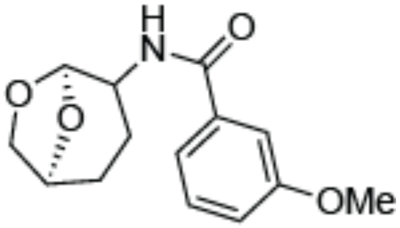

N-[(1S,5R)-6,8-dioxabicyclo[3.2.1]octan-4-yl]-3-methoxybenzamide (9f and 9g)

General procedure D1 was performed, using amine **4** (261 mg, 2.02 mmol, 1.00 eq), 3-methoxybenzoic acid (384 mg, 2.52 mmol, 1.25 eq), DCM (10.0 mL), EDCI·HCl (483 mg, 2.52 mmol, 1.25 eq), DMAP (126 mg, 1.03 mmol, 0.510 eq) and DIPEA (1.1 mL, 6.5 mmol, 3.2 eq), and a reaction time of overnight. Extraction with H_2O (30 mL) and DCM (3×30 mL), followed by a wash of the combined organic layers with brine (30 mL) and subsequent purification by column chromatography (EtOAc/cHex + 1% Et_3N, 0–60%) provided title compound **9f** (89 mg, 17%) and **9g** (120 mg, 23%).

9f: Diastereomeric ratio: >19:1. Proposed major diastereomer: *Exo*.

^{1}H NMR (500 MHz, $CDCl_3$) δ 7.38 (dd, *J* = 2.4, 1.6 Hz, 1H), 7.35 (dd, *J* = 7.8, 7.8 Hz, 1H), 7.31 (ddd, *J* = 7.6, 1.3, 1.3 Hz, 1H), 7.05 (ddd, *J* = 8.0, 2.6, 1.2 Hz, 1H), 6.58 (d, *J* = 8.6 Hz, 1H), 5.41 – 5.36 (m, 1H), 4.59 – 4.52 (m, 1H), 4.25 – 4.17 (m, 1H), 4.01 (d^Δ, *J* = 7.2 Hz, 1H), 3.86 (s, 3H), 3.88 – 3.82 (m, 1H), 2.14 (dddd^Δ, *J* = 13.9, 13.9, 5.4, 5.4 Hz, 1H), 2.07 – 1.95 (m, 1H), 1.75 – 1.67 (m, 1H), 1.53 (dd^Δ, *J* = 14.1, 5.3 Hz, 1H). **^{13}C NMR** (126 MHz, $CDCl_3$) δ 166.7, 160.0, 136.0, 129.7, 118.8, 117.9, 112.6, 101.6, 73.5, 67.6, 55.6, 47.7, 25.6, 21.5. **LCMS** (acidic): t_R: 3.23 min, purity: >99% (254 nm). **HRMS**: $(M + H)^+$ calcd. for $C_{14}H_{17}NO_4$: 264.1230, found: 264.1232.

9g: Diastereomeric ratio: >19:1. Proposed major diastereomer: *Endo*.

^{1}H NMR (500 MHz, $CDCl_3$) δ 7.35 (dd, *J* = 2.4, 1.6 Hz, 1H), 7.32 (dd, *J* = 7.8, 7.8 Hz, 1H), 7.27 (ddd, *J* = 7.7, 1.4, 1.4 Hz, 1H), 7.03 (ddd, *J* = 8.1, 2.6, 1.1 Hz, 1H), 6.09 (d, *J* = 8.8 Hz, 1H), 5.40 (s^Δ, 1H), 4.60 – 4.52 (m,

1H), 4.31 – 4.23 (m, 1H), 3.90 (d$^{\Delta}$, *J* = 7.1 Hz, 1H), 3.84 (s, 3H), 3.86 – 3.81 (m, 1H), 2.10 – 1.97 (m, 2H), 1.67 – 1.54 (m, 2H). **^{13}C NMR** (126 MHz, $CDCl_3$) δ 166.9, 160.0, 135.9, 129.7, 118.8, 118.0, 112.5, 102.1, 73.2, 68.3, 55.6, 49.5, 27.9, 23.6. **LCMS** (acidic): t_R: 3.17 min, purity: 98.8% (254 nm). **HRMS**: $(M + H)^+$ calcd. for $C_{14}H_{17}NO_4$: 264.1230, found: 264.1228.

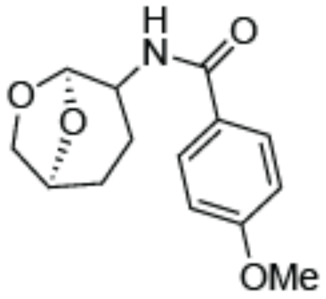

N-[(1S,5R)-6,8-dioxabicyclo[3.2.1]octan-4-yl]-4-methoxybenzamide (9h and 9i)

General procedure D1 was performed, using amine **4** (267 mg, 2.07 mmol, 1.00 eq), 4-methoxybenzoic acid (382 mg, 2.51 mmol, 1.22 eq), DCM (10.0 mL), EDCI·HCl (483 mg, 2.52 mmol, 1.22 eq), DMAP (176 mg, 1.44 mmol, 0.697 eq) and DIPEA (1.1 mL, 6.3 mmol, 3.1 eq), and a reaction time of overnight. Extraction with H_2O (30 mL) and DCM (3×30 mL), followed by a wash of the combined organic layers with brine (30 mL) and subsequent purification by column chromatography (EtOAc/cHex + 1% Et_3N, 20–50%) provided the (partially) separated amides. For **9h**: The residue was dissolved in DCM (20 mL) and washed with satd. aq. NH_4Cl (40 mL). This provided title compound **9h** (147 mg, 27%). For **9i**: The residue was dissolved in DCM (20 mL) and washed with satd. aq. NH_4Cl (40 mL). The organic layer was concentrated *in vacuo*, and the residue subjected to column chromatography (EtOAc/cHex + 1% Et_3N, 20–70%). This provided title compound **9i** (14 mg, 3%).

9h: Diastereomeric ratio: >19:1. Proposed major diastereomer: *Exo*.

^{1}H NMR (500 MHz, $CDCl_3$) δ 7.81 – 7.72 (m, 2H), 6.97 – 6.90 (m, 2H), 6.51 (d, *J* = 8.8 Hz, 1H), 5.38 (s$^{\Delta}$, 1H), 4.59 – 4.50 (m, 1H), 4.24 – 4.16 (m, 1H), 4.00 (d$^{\Delta}$, *J* = 7.2 Hz, 1H), 3.85 (s, 3H), 3.88 – 3.81 (m, 1H), 2.18 – 2.07 (m, 1H), 2.06 – 1.96 (m, 1H), 1.72 – 1.66 (m, 1H), 1.52 (dd$^{\Delta}$, *J* = 13.9, 5.4 Hz, 1H). **^{13}C NMR** (126 MHz, $CDCl_3$) δ 166.4, 162.4, 128.9, 126.8, 113.9, 101.7, 73.5, 67.6, 55.6, 47.5, 25.6, 21.5. **LCMS** (acidic): t_R: 3.17 min, purity: >99% (254 nm). **HRMS**: $(M + H)^+$ calcd. for $C_{14}H_{17}NO_4$: 264.1230, found: 264.1228.

9i: Diastereomeric ratio: >19:1. Proposed major diastereomer: *Endo*.

^{1}H NMR (500 MHz, $CDCl_3$) δ 7.78 – 7.69 (m, 2H), 6.95 – 6.85 (m, 2H), 6.00 (d, *J* = 8.9 Hz, 1H), 5.39 (s$^{\Delta}$, 1H), 4.60 – 4.53 (m, 1H), 4.31 – 4.21 (m, 1H), 3.90 (d$^{\Delta}$, *J* = 7.1 Hz, 1H), 3.84 (s, 3H), 3.87 – 3.78 (m, 1H), 2.10 – 1.94 (m, 2H), 1.66 – 1.52 (m, 2H). **^{13}C NMR** (126 MHz, $CDCl_3$) δ 166.5, 162.4, 128.9, 126.6, 113.8, 102.2, 73.1, 68.3, 55.5, 49.3, 27.9, 23.6. **LCMS** (acidic): t_R: 3.11 min, purity: >99% (254 nm). **HRMS**: $(M + H)^+$ calcd. for $C_{14}H_{17}NO_4$: 264.1230, found: 264.1234.

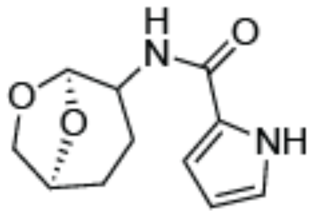

N-[(1S,5R)-6,8-dioxabicyclo[3.2.1]octan-4-yl]-1H-pyrrole-2-carboxamide (9j)

General procedure D1 was performed, using amine **4** (264 mg, 2.04 mmol, 1.00 eq), 1H-pyrrole-2-carboxylic acid (278 mg, 2.50 mmol, 1.23 eq), DCM (10.0 mL), EDCI·HCl (476 mg, 2.48 mmol, 1.22 eq), DMAP (125 mg, 1.02 mmol, 0.501 eq) and DIPEA (1.1 mL, 6.5 mmol, 3.2 eq), and a reaction time of 2 d. Extraction with H_2O (30 mL) and DCM (3×30 mL), followed by a wash of the combined organic layers with satd. aq. NH_4Cl (30 mL) and subsequent purification by column chromatography (EtOAc/cHex + 1% Et_3N, 20–90%) were performed. After following the general procedure, the residue was dissolved in DCM (40 mL) and washed with H_2O (40 mL). The aqueous layer was extracted with DCM (2×30 mL). The combined organic layers were concentrated *in vacuo* and the residue subjected to column chromatography (EtOAc/cHex + 1% Et_3N, 30–70%). This provided the title compound (121 mg, 27%).

Diastereomeric ratio: 1.0:1.0.

^{1}H NMR (500 MHz, CD_3OD) δ 6.93 – 6.87 (m, 1.5H)^{a,b}, 6.85 (dd, *J* = 3.7, 1.4 Hz, 0.5H)$^{a/b}$, 6.16 (dd, *J* = 3.7, 2.6 Hz, 0.5H)$^{a/b}$, 6.14 (dd, *J* = 3.7, 2.6 Hz, 0.5H)$^{a/b}$, 5.36 – 5.32 (m, 0.5H)$^{a/b}$, 5.31 (s$^{\Delta}$, 0.5H)$^{a/b}$, 4.56 – 4.50 (m, 1H)^{a,b}, 4.06 – 3.98 (m, 1H)^{a,b}, 3.97 – 3.92 (m, 1H)^{a,b}, 3.78 – 3.72 (m, 1H)^{a,b}, 2.21 – 2.10 (m, 1H)^{a,b}, 1.98 – 1.89 (m, 0.5H)$^{a/b}$, 1.88 – 1.77 (m, 1H)^{a,b}, 1.70 – 1.63 (m, 0.5H)$^{a/b}$, 1.61 – 1.56 (m, 0.5H)$^{a/b}$, 1.49 (d$^{\Delta}$, *J* = 8.9 Hz, 0.5H)$^{a/b}$. **^{13}C NMR** (126 MHz, CD_3OD) δ 163.3, 163.2, 126.6, 126.5, 123.1, 123.1, 112.7, 112.5, 110.3, 110.2, 103.2, 102.4, 74.7, 74.4, 69.2, 68.3, 51.0, 48.7, 29.0, 26.0, 23.1, 22.0. **LCMS** (acidic): t_R: 2.57 min, purity: >99% (254 nm). **HRMS**: $(M + H)^+$ calcd. for $C_{11}H_{14}N_2O_3$: 223.1077, found: 223.1070.

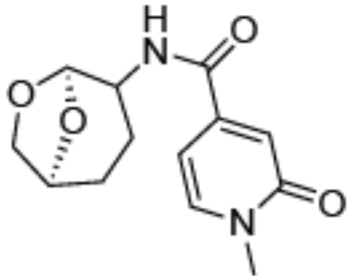

N-((1S,5R)-6,8-dioxabicyclo[3.2.1]octan-4-yl)-1-methyl-2-oxo-1,2-dihydropyridine-4-carboxamide (9k)

General procedure D1 was performed, using amine **4** (268 mg, 2.07 mmol, 1.00 eq), 1-methyl-2-oxopyridine-4-carboxylic acid (390 mg, 2.55 mmol, 1.23 eq), DCM (10.0 mL), EDCI·HCl (502 mg, 2.62 mmol, 1.26 eq), DMAP (131 mg, 1.07 mmol, 0.517 eq) and DIPEA (1.2 mL, 6.6 mmol, 3.2 eq), and a reaction time of overnight. Extraction with H_2O (40 mL) and DCM (3×30 mL), followed by a wash of the combined organic layers with satd. aq. NH_4Cl (40 mL) and subsequent purification by column chromatography (MeOH/DCM + 1% Et_3N, 0–10%) provided the title compound (124 mg, 23%).

Diastereomeric ratio: 1.1:1.0. Proposed major diastereomer: *Exo*.

^{1}H NMR (500 MHz, $CDCl_3$) δ 7.39 (d^{Δ}, *J* = 7.0 Hz, 0.5H)^{a,b}, 7.38 (d^{Δ}, *J* = 7.0 Hz, 0.5H)^{a,b}, 6.95 – 6.89 (m, 1H)^{a,b}, 6.65 (d, *J* = 9.0 Hz, 0.5H)^{b}, 6.62 – 6.56 (m, 1H)^{a}, 6.28 (d, *J* = 9.1 Hz, 0.5H)^{a}, 5.37 (s^{Δ}, 0.5H)^{a}, 5.36 – 5.34 (m, 0.5H)^{b}, 4.60 – 4.56 (m, 0.5H)^{a}, 4.56 – 4.52 (m, 0.5H)^{b}, 4.19 (dddd, *J* = 10.7, 9.2, 5.7, 1.6 Hz, 0.5H)^{a}, 4.16 – 4.09 (m, 0.5H)^{b}, 3.99 (d^{Δ}, *J* = 7.2 Hz, 0.5H)^{b}, 3.90 (d^{Δ}, *J* = 7.1 Hz, 0.5H)^{a}, 3.87 – 3.80 (m, 1H)^{a,b}, 3.59 (s, 1.5H)$^{a/b}$, 3.58 (s, 1.5H)$^{a/b}$, 2.19 – 1.95 (m, 2H)^{a,b}, 1.71 – 1.56 (m, 1.5H)^{a,b}, 1.53 (dd, *J* = 14.2, 5.8 Hz, 0.5H)^{b}. **^{13}C NMR** (126 MHz, $CDCl_3$) δ 164.5$^{a/b}$, 164.4$^{a/b}$, 162.8$^{a/b}$, 162.8$^{a/b}$, 145.7$^{a/b}$, 145.6$^{a/b}$, 139.2$^{a/b}$, 139.1$^{a/b}$, 118.1$^{a/b}$, 118.1$^{a/b}$, 104.8$^{a/b}$, 104.6$^{a/b}$, 101.7$^{a/b}$, 101.1$^{a/b}$, 73.5$^{a/b}$, 73.1$^{a/b}$, 68.3$^{a/b}$, 67.7$^{a/b}$, 49.7^{a}, 47.8^{b}, 38.0$^{a/b}$, 38.0$^{a/b}$, 27.8^{a}, 25.5^{b}, 23.3^{a}, 21.4^{b}. **LCMS** (acidic): t_R: 2.13 min, purity: >99% (254 nm). **HRMS**: $(M + H)^+$ calcd. for $C_{13}H_{16}N_2O_4$: 265.1183, found: 265.1193.

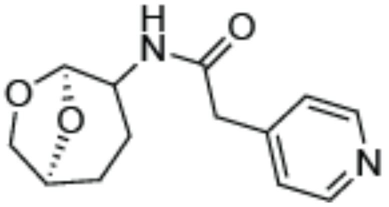

N-[(1S,5R)-6,8-dioxabicyclo[3.2.1]octan-4-yl]-2-(pyridin-4-yl)acetamide (9l)

General procedure D1 was performed, using amine **4** (275 mg, 2.13 mmol, 1.00 eq), 2-pyridin-4-ylacetic acid (367 mg, 2.68 mmol, 1.26 eq), DCM (10.0 mL), EDCI·HCl (534 mg, 2.78 mmol, 1.31 eq), DMAP (138 mg, 1.13 mmol, 0.531 eq) and DIPEA (1.2 mL, 6.8 mmol, 3.2 eq), and a reaction time of overnight. Extraction with H_2O (40 mL) and DCM (5×30 mL), followed by a wash of the combined organic layers with satd. aq. NH_4Cl (40 mL) and subsequent purification by column chromatography (MeOH/DCM + 1% Et_3N, 0–10%) provided the title compound (181 mg, 34%).

Diastereomeric ratio: 1.2:1.0. Proposed major diastereomer: *Endo*.

^{1}H NMR (500 MHz, $CDCl_3$) δ 8.67 – 8.53 (m, 2H)^{a,b}, 7.43 – 7.31 (m, 2H)^{a,b}, 6.11 (d, *J* = 7.5 Hz, 0.5H)^{b}, 5.67 (d, *J* = 8.0 Hz, 0.5H)^{a}, 5.28 (s^{Δ}, 0.5H)^{a}, 5.26 – 5.23 (m, 0H.5)^{b}, 4.55 – 4.51 (m, 0.5H)^{a}, 4.50 – 4.45 (m, 0.5H)^{b}, 4.09 – 4.02 (m, 0.5H)^{a}, 4.03 – 3.96 (m, 0.5H)^{b}, 3.95 (d^{Δ}, *J* = 7.2 Hz, 0.5H)^{b}, 3.84 (d^{Δ}, *J* = 7.1 Hz, 0.5H)^{a}, 3.82 – 3.76 (m, 1H)^{a,b}, 3.66 – 3.52 (m, 2H)^{a,b}, 2.12 – 2.01 (m, 0.5H)^{b}, 2.00 – 1.90 (m, 1H)^{a}, 1.90 – 1.82 (m, 0.5H)^{b}, 1.63 – 1.51 (m, 1H)^{a,b}, 1.51 – 1.40 (m, 1H)^{a,b}. **^{13}C NMR** (126 MHz, $CDCl_3$) δ 168.1$^{a/b}$, 168.0$^{a/b}$, 148.5$^{a/b}$, 148.4$^{a/b}$, 146.1^{a,b}, 125.2$^{a/b}$, 125.1$^{a/b}$, 101.8^{a}, 101.2^{b}, 73.4^{b}, 73.1^{a}, 68.3^{a}, 67.6^{b}, 49.4^{a}, 47.6^{b}, 43.1$^{a/b}$, 43.0$^{a/b}$, 27.8^{a}, 25.4^{b}, 23.3^{a}, 21.3^{b}. **LCMS** (acidic): t_R: 3.22 min, purity: >99% (254 nm). **HRMS**: $(M + H)^+$ calcd. for $C_{13}H_{16}N_2O_3$: 249.1234, found: 249.1245.

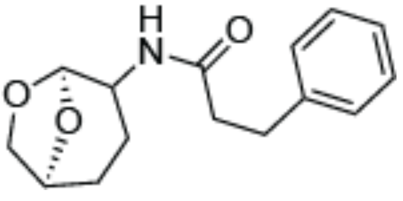

N-[(1S,5R)-6,8-dioxabicyclo[3.2.1]octan-4-yl]-3-phenylpropanamide (9m)

General procedure D1 was performed, using amine **4** (289 mg, 2.24 mmol, 1.00 eq), 3-phenylpropanoic acid (395 mg, 2.63 mmol, 1.18 eq), DCM (10.0 mL), EDCI·HCl (600 mg, 3.13 mmol, 1.40 eq), DMAP (144

mg, 1.18 mmol, 0.527 eq) and DIPEA (1.2 mL, 7.2 mmol, 3.2 eq), and a reaction time of overnight. Extraction with H_2O (30 mL) and DCM (3×30 mL), followed by a wash of the combined organic layers with satd. aq. NH_4Cl (30 mL) and subsequent purification by column chromatography (EtOAc/cHex + 1% Et_3N, 20–80%) provided the title compound (275 mg, 47%).

Diastereomeric ratio: 1.2:1.0. Proposed major diastereomer: *Endo*.

^{1}H NMR (500 MHz, $CDCl_3$) δ 7.32 – 7.25 (m, 2H)^{a,b}, 7.23 – 7.16 (m, 3H)^{a,b}, 5.77 (d, *J* = 8.4 Hz, 0.5H)^b, 5.32 (d, *J* = 8.7 Hz, 0.5H)^a, 5.19 (s$^\Delta$, 0.5H)^a, 5.17 – 5.12 (m, 0.5H)^b, 4.54 – 4.47 (m, 0.5H)^a, 4.46 – 4.39 (m, 0.5H)^b, 4.08 – 3.99 (m, 0.5H)^a, 3.98 – 3.93 (m, 0.5H)^b, 3.91 (d$^\Delta$, *J* = 7.2 Hz, 0.5H)^b, 3.81 (d$^\Delta$, *J* = 7.0 Hz, 0.5H)^a, 3.80 – 3.73 (m, 1H)^{a,b}, 3.03 – 2.90 (m, 2H)^{a,b}, 2.58 – 2.38 (m, 2H)^{a,b}, 2.04 – 1.82 (m, 1.5H)^{a,b}, 1.72 – 1.65 (m, 0.5H)^b, 1.59 – 1.51 (m, 0.5H)^a, 1.46 – 1.29 (m, 1.5)^{a,b}. **^{13}C NMR** (126 MHz, $CDCl_3$) δ 171.6$^{a/b}$, 171.5$^{a/b}$, 140.8$^{a/b}$, 140.8$^{a/b}$, 128.7$^{a/b}$, 128.7$^{a/b}$, 128.5$^{a/b}$, 128.5$^{a/b}$, 126.4$^{a/b}$, 126.4$^{a/b}$, 102.1^a, 101.5^b, 73.3^b, 73.1^a, 68.2^a, 67.5^b, 48.8^a, 47.1^b, 38.8^b, 38.7^a, 32.0^b, 31.8^a, 27.8^a, 25.3^b, 23.4^a, 21.4^b. **LCMS** (acidic): t_R: 3.36 min, purity: 97.2% (254 nm). **HRMS**: $(M + H)^+$ calcd. for $C_{15}H_{19}NO_3$: 262.1438, found: 262.1440.

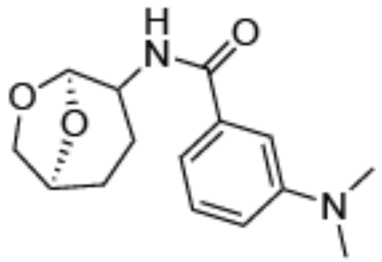

3-(dimethylamino)-N-[(1S,5R)-6,8-dioxabicyclo[3.2.1]octan-4-yl]benzamide (9n)

General procedure D2 was performed, using amine **4** (465 mg, 3.60 mmol, 1.80 eq), 3-(dimethylamino)benzoic acid (330 mg, 2.00 mmol, 1.00 eq), DMF (12.5 mL), EDCI·HCl (498 mg, 2.60 mmol, 1.30 eq) and DMAP (61 mg, 0.50 mmol, 0.25 eq), and a reaction time of overnight. Diluting with EtOAc (10 mL) and washing with brine (2×15 mL), followed by purification by column chromatography (EtOAc/cHex, 30%) provided the title compound (72 mg, 13%).

Diastereomeric ratio: >19:1. Proposed major diastereomer: *Endo*.

^{1}H NMR (500 MHz, $CDCl_3$) δ 7.29 – 7.22 (m, 1H), 7.18 (dd$^\Delta$, *J* = 2.4, 1.7 Hz, 1H), 6.96 (d$^\Delta$, *J* = 7.6 Hz, 1H), 6.83 (dd$^\Delta$, *J* = 8.2, 2.5 Hz, 1H), 6.06 (d, *J* = 8.8 Hz, 1H), 5.41 (s$^\Delta$, 1H), 4.61 – 4.54 (m, 1H), 4.35 – 4.23 (m, 1H), 3.91 (d$^\Delta$, *J* = 7.1 Hz, 1H), 3.86 – 3.80 (m, 1H), 2.99 (s, 6H), 2.13 – 1.96 (m, 2H), 1.67 – 1.53 (m, 2H). **^{13}C NMR** (126 MHz, $CDCl_3$) δ 167.9, 150.9, 135.3, 129.2, 115.5, 114.0, 111.5, 102.2, 73.2, 68.3, 49.4, 40.7, 28.0, 23.6. **LCMS** (acidic): t_R: 2.52 min, purity: >99% (230 nm). **HRMS**: $(M + H)^+$ calcd. for $C_{15}H_{20}N_2O_3$: 277.1547, found: 277.1546.

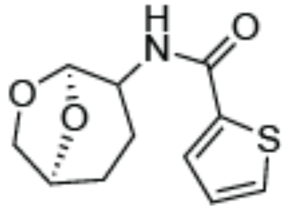

N-[(1S,5R)-6,8-dioxabicyclo[3.2.1]octan-4-yl]thiophene-2-carboxamide (9o and 9p)

General procedure D2 was performed, using amine **4** (370 mg, 2.86 mmol, 1.43 eq), thiophene-2-carboxylic acid (293 mg, 2.00 mmol, 1.00 eq), DMF (12.5 mL), EDCI·HCl (526 mg, 2.74 mmol, 1.37 eq) and DMAP (63 mg, 0.52 mmol, 0.26 eq), and a reaction time of overnight. Diluting with EtOAc and washing with brine (2×), followed by purification by column chromatography (EtOAc/cHex, 50%) provided title compound **9o** (69 mg, 14%) and **9p** (116 mg, 24%).

9o: Diastereomeric ratio: >19:1. Proposed major diastereomer: *Endo*.

^{1}H NMR (500 MHz, $CDCl_3$) δ 7.50 (dd, *J* = 3.7, 1.0 Hz, 1H), 7.48 (dd, *J* = 5.0, 1.0 Hz, 1H), 7.07 (dd, *J* = 4.9, 3.8 Hz, 1H), 5.92 (d, *J* = 8.5 Hz, 1H), 5.40 ($s^Δ$, 1H), 4.61 – 4.52 (m, 1H), 4.29 – 4.19 (m, 1H), 3.91 ($d^Δ$, *J* = 7.1 Hz, 1H), 3.87 – 3.80 (m, 1H), 2.13 – 1.95 (m, 2H), 1.68 – 1.54 (m, 2H). **^{13}C NMR** (126 MHz, $CDCl_3$) δ 161.4, 138.8, 130.3, 128.3, 127.7, 102.1, 73.2, 68.3, 49.4, 27.9, 23.6. **LCMS** (acidic): t_R: 2.82 min, purity: >99% (254 nm). **HRMS**: $(M + H)^+$ calcd. for $C_{11}H_{13}NO_3S$: 240.0689, found: 240.0686.

9p: Diastereomeric ratio: 2:1. Proposed major diastereomer: *Exo*.

^{1}H NMR (500 MHz, $CDCl_3$) δ 7.53 (dd, *J* = 3.7, 1.1 Hz, 0.7H)^a, 7.52 – 7.46 (m, 1.3H)^{a,b}, 7.12 – 7.05 (m, 1H)^{a,b}, 6.42 (d, *J* = 8.5 Hz, 0.7H)^a, 5.92 (d, *J* = 8.5 Hz, 0.3H)^b, 5.39 ($s^Δ$, 0.3H)^b, 5.39 – 5.37 (m, 0.7H)^a, 4.63 – 4.52 (m, 1H)^{a,b}, 4.24 (dddd, *J* = 10.7, 9.2, 5.9, 1.4 Hz, 0.3H)^b, 4.21 – 4.14 (m, 0.7H)^a, 4.00 ($d^Δ$, *J* = 7.2 Hz, 0.7H)^a, 3.91 (^{dΔ}, *J* = 7.1 Hz, 0.3H)^b, 3.88 – 3.80 (m, 1H)^{a,b}, 2.18 – 1.94 (m, 2H)^{a,b}, 1.76 – 1.68 (m, 0.7H)^a, 1.63 – 1.49 (m, 1.3H)^{a,b}. **^{13}C NMR** (126 MHz, $CDCl_3$) δ 161.4^b, 161.3^a, 139.0^a, 138.8^b, 130.3^b, 130.3^a, 128.3^b, 128.3^a, 127.7^a, 127.7^b, 102.1^b, 101.5^a, 73.5^a, 73.1^b, 68.3^b, 67.6^a, 49.4^b, 47.6^a, 27.9^b, 25.6^a, 23.6^b, 21.5^a. **LCMS** (acidic): t_R: 2.88 min, purity: >99% (254 nm). **HRMS**: $(M + H)^+$ calcd. for $C_{11}H_{13}NO_3S$: 240.0689, found: 240.0686.

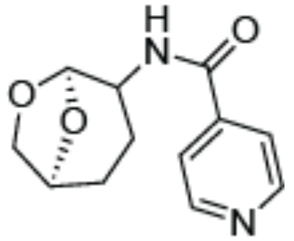

N-[(1S,5R)-6,8-dioxabicyclo[3.2.1]octan-4-yl]pyridine-4-carboxamide (9q)

General procedure D2 was performed, using amine **4** (373 mg, 2.89 mmol, 1.78 eq), pyridine-4-carboxylic acid (200 mg, 1.63 mmol, 1.00 eq), DMF (10.0 mL), EDCI·HCl (404 mg, 2.11 mmol, 1.30 eq) and DMAP (49 mg, 0.40 mmol, 0.25 eq), and a reaction time of 1 d. Diluting with EtOAc (20 mL) and washing with brine (3×20 mL), followed by purification by column chromatography (MeOH/EtOAc, 0–30%) provided the title compound (16 mg, 4%).

Diastereomeric ratio: 1.5:1:0. Proposed major diastereomer: *Endo*.

^{1}H NMR (500 MHz, $CDCl_3$) δ 8.84 – 8.67 (m, 2H)^{a,b}, 7.72 – 7.57 (m, 2H)^{a,b}, 6.67 (d, *J* = 7.7 Hz, 0.4H)^b, 6.22 (d, *J* = 7.2 Hz, 0.6H)^a, 5.40 ($s^Δ$, 0.6H)^a, 5.39 ($s^Δ$, 0.4H)^b, 4.59 ($s^Δ$, 0.6H)^a, 4.57 (s, 0.4H)^b, 4.32 – 4.23 (m, 0.6H)^a, 4.23 – 4.17 (m, 0.4H)^b, 4.02 ($d^Δ$, *J* = 7.2 Hz, 0.4H)^b, 3.91 ($d^Δ$, *J* = 7.1 Hz, 0.6H)^a, 3.89 – 3.81 (m, 1H)^a, 2.24 – 1.98 (m, 2H)^{a,b}, 1.75 – 1.51 (m, 2H)^{a,b}. **^{13}C NMR** (126 MHz, $CDCl_3$) δ 165.0^a, 164.9^b, 150.6^b, 150.5^a, 141.8^b, 141.7^a, 121.2^{a,b}, 101.8^a, 101.3^b, 73.5^b, 73.1^a, 68.4^a, 67.7^b, 49.7^a, 47.9^b, 27.8^a, 25.5^b, 23.5^a, 21.4^b. **LCMS**

(acidic): t_R: 1.82 min, purity: >99% (254 nm). **HRMS**: $(M + H)^+$ calcd. for $C_{12}H_{14}N_2O_3$: 235.1077, found: 235.1073.

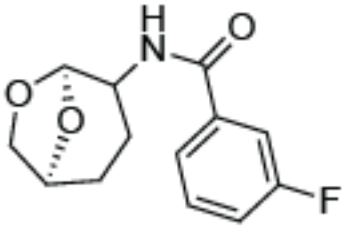

N-[(1S,5R)-6,8-dioxabicyclo[3.2.1]octan-4-yl]-3-fluorobenzamide (9r)

General procedure D2 was performed, using amine **4** (235 mg, 1.82 mmol, 1.75 eq), 3-fluorobenzoic acid (146 mg, 1.04 mmol, 1.00 eq), DMF (6.5 mL), EDCI·HCl (280 mg, 1.46 mmol, 1.40 eq) and DMAP (32 mg, 0.26 mmol, 0.25 eq), and a reaction time of overnight. Diluting with EtOAc (12 mL) and washing with brine (12 mL) provided the title compound (75 mg, 29%).

Diastereomeric ratio: 1.3:1.0. Proposed major diastereomer: *Endo*.

^{1}H NMR (500 MHz, $CDCl_3$) δ 7.60 – 7.45 (m, 2H)^{a,b}, 7.46 – 7.37 (m, 1H)^{a,b}, 7.24 – 7.15 (m, 1H)^{a,b}, 6.56 (d, *J* = 8.4 Hz, 0.4H)^b, 6.06 (d, *J* = 8.1 Hz, 0.6H)^a, 5.40 (s$^\Delta$, 0.6H)^a, 5.39 – 5.36 (m, 0.4H)^b, 4.60 – 4.54 (m, 1H)^{a,b}, 4.27 (dddd, *J* = 10.7, 9.2, 5.9, 1.5 Hz, 0.6H)^a, 4.20 (dddd, *J* = 9.4, 4.8, 2.3, 2.3 Hz, 0.4H)^b, 4.01 (d$^\Delta$, *J* = 7.2 Hz, 0.4H)^b, 3.91 (d$^\Delta$, *J* = 7.0 Hz, 0.6H)^a, 3.89 – 3.81 (m, 1H)^{a,b}, 2.21 – 1.96 (m, 2H)^{a,b}, 1.74 – 1.50 (m, 2H)^{a,b}. **^{13}C NMR** (126 MHz, $CDCl_3$) δ 165.8 – 165.6 (m)^{a,b}, 162.92 (d, *J* = 247.8 Hz)$^{a/b}$, 162.9 (d, *J* = 247.9 Hz)$^{a/b}$, 136.9 – 136.6 (m)^{a,b}, 130.5 – 130.3 (m)^{a,b}, 122.6 – 122.5 (m)^{a,b}, 119.1 – 118.5 (m)^{a,b}, 114.6 (d, *J* = 23.2 Hz)$^{a/b}$, 114.6 (d, *J* = 22.9 Hz)$^{a/b}$, 102.0^a, 101.5^b, 73.5^b, 73.2^a, 68.4^a, 67.6^b, 49.5^a, 47.8^b, 27.9^a, 25.6^b, 23.6^a, 21.5^b. **LCMS** (acidic): t_R: 3.15 min, purity: >99% (230 nm). **HRMS**: $(M + H)^+$ calcd. for $C_{13}H_{14}FNO_3$: 252.1031, found: 252.1021.

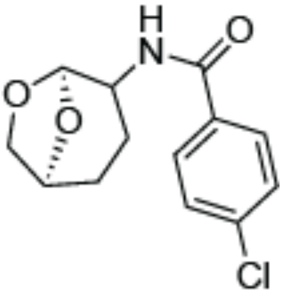

4-chloro-N-[(1S,5R)-6,8-dioxabicyclo[3.2.1]octan-4-yl]benzamide (9s)

General procedure D2 was performed, using amine **4** (221 mg, 1.71 mmol, 1.80 eq), 4-chlorobenzoic acid (149 mg, 0.95 mmol, 1.00 eq), DMF (5.9 mL), EDCI·HCl (237 mg, 1.24 mmol, 1.30 eq) and DMAP (29 mg, 0.24 mmol, 0.25 eq), and a reaction time of 2 d. Diluting with EtOAc (10 mL) and washing with brine (2×15 mL), followed by purification by column chromatography (EtOAc/cHex, 40%) provided the title compound (26 mg, 10%).

Diastereomeric ratio: 1.5:1.0. Proposed major diastereomer: *Endo*.

^{1}H NMR (500 MHz, $CDCl_3$) δ 7.77 – 7.67 (m, 2H)^{a,b}, 7.46 – 7.36 (m, 2H)^{a,b}, 6.54 (d, *J* = 8.3 Hz, 0.4H)^b, 6.03 (d, *J* = 8.8 Hz, 0.6H)^a, 5.39 (s^Δ, 0.6H)^a, 5.39 – 5.36 (m, 0.4H)^b, 4.61 – 4.56 (m, 0.6H)^a, 4.56 – 4.53 (m, 0.4H)^b, 4.26 (dddd, *J* = 10.6, 9.0, 5.8, 1.4 Hz, 0.6H)^a, 4.19 (dddd, *J* = 8.8, 4.4, 2.1, 2.1 Hz, 0.4H)^b, 4.01 (d^Δ, *J* = 7.2 Hz, 0.4H)^b, 3.91 (d^Δ, *J* = 7.1 Hz, 0.6H)^a, 3.88 – 3.82 (m, 1H)^{a,b}, 2.20 – 1.94 (m, 2H)^{a,b}, 1.75 – 1.49 (m, 2H)^{a,b}. **^{13}C NMR** (126 MHz, $CDCl_3$) δ 165.9$^{a/b}$, 165.8$^{a/b}$, 138.0^{a,b}, 132.9^b, 132.7^a, 129.0^b, 129.0^a, 128.6^b, 128.6^a, 102.0^a, 101.5^b, 73.5^b, 73.2^a, 68.3^a, 67.6^b, 49.5^a, 47.8^b, 27.9^a, 25.6^b, 23.6^a, 21.5^b. **LCMS** (acidic): t_R: 3.47 min, purity: >99% (254 nm). **HRMS**: $(M + H)^+$ calcd. for $C_{13}H_{14}ClNO_3$: 268.0735, found: 268.0726.

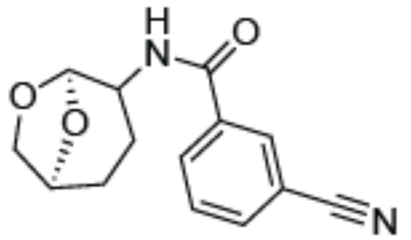

N-((1S,5R)-6,8-dioxabicyclo[3.2.1]octan-4-yl)-3-cyanobenzamide (9t)

General procedure D2 was performed, using amine **4** (165 mg, 1.28 mmol, 1.10 eq), 3-cyanobenzoic acid (171 mg, 1.16 mmol, 1.00 eq), DMF (7.5 mL), EDCI·HCl (290 mg, 1.51 mmol, 1.30 eq), DMAP (36 mg, 0.29 mmol, 0.25 eq) and DIPEA (0.26 mL, 1.5 mmol, 1.3 eq), and a reaction time of overnight. Diluting with EtOAc (14 mL) and washing with H_2O (7 mL) provided the title compound (26 mg, 9%).

Diastereomeric ratio: 1.1:1.0. Proposed major diastereomer: *Exo*.

^{1}H NMR (300 MHz, $CDCl_3$) δ 8.14 – 7.96 (m, 2H)^{a,b}, 7.85 – 7.74 (m, 1H)^{a,b}, 7.64 – 7.53 (m, 1H)^{a,b}, 6.59 (d, *J* = 8.4 Hz, 0.5H)^a, 6.11 (d, *J* = 8.8 Hz, 0.5H)^b, 5.45 – 5.36 (m, 1H)^{a,b}, 4.62 – 4.54 (m, 1H)^{a,b}, 4.34 – 4.23 (m, 0.5H)^b, 4.23 – 4.16 (m, 0.5H)^a, 4.02 (d^Δ, *J* = 7.2 Hz, 0.5H)^a, 3.92 (d^Δ, *J* = 7.1 Hz, 0.5H)^b, 3.90 – 3.81 (m, 1H)^{a,b}, 2.26 – 1.94 (m, 2H)^{a,b}, 1.82 – 1.47 (m, 2H)^{a,b}. **^{13}C NMR** (76 MHz, $CDCl_3$) δ 164.8$^{a/b}$, 164.7$^{a/b}$, 135.7$^{a/b}$, 135.6$^{a/b}$, 134.9^{a,b}, 131.4$^{a/b}$, 131.4$^{a/b}$, 131.0^{a,b}, 129.8$^{a/b}$, 129.7$^{a/b}$, 118.1$^{a/b}$, 118.1$^{a/b}$, 113.2$^{a/b}$, 113.1$^{a/b}$, 101.8^b, 101.3^a, 73.5^a, 73.1^b, 68.4^b, 67.7^a, 49.7^b, 47.9^a, 27.8^b, 25.5^a, 23.5^b, 21.4^a. **LCMS** (acidic): t_R: 3.08 min, purity: >99% (254 nm). **HRMS**: $(M + H)^+$ calcd. for $C_{14}H_{14}N_2O_3$: 259.1077, found: 259.1084.

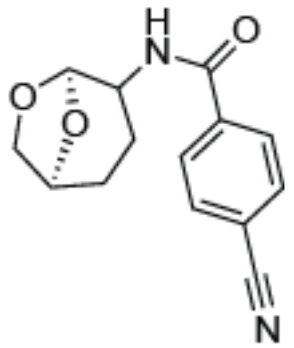

N-((1S,5R)-6,8-dioxabicyclo[3.2.1]octan-4-yl)-4-cyanobenzamide (9u)

General procedure D2 was performed, using amine **4** (122 mg, 0.945 mmol, 1.10 eq), 4-cyanobenzoic acid (127 mg, 0.860 mmol, 1.00 eq), DMF (5.4 mL), EDCI·HCl (214 mg, 1.12 mmol, 1.30 eq), DMAP (26 mg, 0.22

mmol, 0.25 eq) and DIPEA (0.20 mL, 1.1 mmol, 1.3 eq), and a reaction time of overnight. Extraction with H_2O (20 mL) and EtOAc (2×20 mL), followed by a wash of the combined organic layers with brine (10 mL) and subsequent purification by column chromatography (EtOAc/cHex, 0–70%) provided the title compound (84 mg, 38%).

Diastereomeric ratio: 1.2:1.0. Proposed major diastereomer: *Endo*.

^{1}H NMR (300 MHz, $CDCl_3$) δ 7.98 – 7.82 (m, 2H)^{a,b}, 7.82 – 7.67 (m, 2H)^{a,b}, 6.62 (d, *J* = 8.7 Hz, 0.5H)^{b}, 6.14 (d, *J* = 8.4 Hz, 0.5H)^{a}, 5.47 – 5.32 (m, 1H)^{a,b}, 4.65 – 4.52 (m, 1H)^{a,b}, 4.36 – 4.14 (m, 1H)^{a,b}, 4.02 (d$^{\Delta}$, *J* = 7.2 Hz, 0.5H)^{b}, 3.90 (d$^{\Delta}$, *J* = 7.2 Hz, 0.5H)^{a}, 3.89 – 3.77 (m, 1H)^{a,b}, 2.25 – 1.92 (m, 2H)^{a,b}, 1.73 – 1.48 (m, 2H)^{a,b}. **^{13}C NMR** (76 MHz, $CDCl_3$) δ 165.2$^{a/b}$, 165.1$^{a/b}$, 138.4$^{a/b}$, 138.2$^{a/b}$, 132.6$^{a/b}$, 132.6$^{a/b}$, 127.9$^{a/b}$, 127.9$^{a/b}$, 118.1$^{a/b}$, 115.3$^{a/b}$, 101.8^{a}, 101.3^{b}, 73.5^{b}, 73.1^{a}, 68.3^{a}, 67.7^{b}, 49.7^{a}, 47.9^{b}, 27.8^{a}, 25.5^{b}, 23.5^{a}, 21.4^{b}. **LCMS** (acidic): t_R: 3.09 min, purity: >99% (254 nm). **HRMS**: $(M + H)^+$ calcd. for $C_{14}H_{14}N_2O_3$: 259.1077, found: 259.1081.

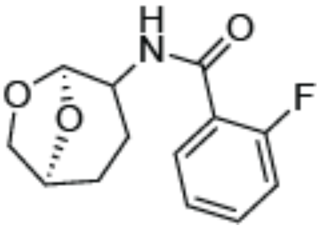

N-((1S,5R)-6,8-dioxabicyclo[3.2.1]octan-4-yl)-2-fluorobenzamide (9v)

General procedure D2 was performed, using amine **4** (113 mg, 0.875 mmol, 1.10 eq), 2-fluorobenzoic acid (111 mg, 0.796 mmol, 1.00 eq), DMF (5.0 mL), EDCI·HCl (198 mg, 1.04 mmol, 1.30 eq), DMAP (97 mg, 0.80 mmol, 1.0 eq) and DIPEA (0.18 mL, 1.0 mmol, 1.3 eq), and a reaction time of 3 d. Extraction with H_2O (10 mL) and EtOAc (2×10 mL), followed by a wash of the combined organic layers with brine (10 mL) and subsequent purification by column chromatography (EtOAc/cHex, 40%) provided the title compound (16 mg, 8%).

Diastereomeric ratio: 1.2:1.0. Proposed major diastereomer: *Endo*.

^{1}H NMR (600 MHz, $CDCl_3$) δ 8.10 – 8.03 (m, 1H)^{a,b}, 7.51 – 7.42 (m, 1H)^{a,b}, 7.30 – 7.23 (m, 1H)^{a,b}, 7.20 (dd, *J* = 9.6, 9.6 Hz, 0.5H)^{b}, 7.16 – 7.08 (m, 1H)^{a,b}, 6.66 (dd, *J* = 9.4, 9.4 Hz, 0.5H)^{a}, 5.42 (s$^{\Delta}$, 0.5H)^{a}, 5.41 – 5.37 (m, 0.5H)^{b}, 4.62 – 4.51 (m, 1H)^{a,b}, 4.37 – 4.27 (m, 0.5H)^{a}, 4.27 – 4.20 (m, 0.5H)^{b}, 4.01 (d$^{\Delta}$, *J* = 7.1 Hz, 0.5H)^{b}, 3.92 (d$^{\Delta}$, *J* = 7.1 Hz, 0.5H)^{a}, 3.87 – 3.82 (m, 1H)^{a,b}, 2.19 – 2.11 (m, 0.5H)^{b}, 2.11 – 1.97 (m, 1.5H)^{a,b}, 1.75 – 1.68 (m, 0.5H)^{b}, 1.67 – 1.57 (m, 1H)^{a}, 1.53 (dd$^{\Delta}$, *J* = 14.5, 5.7 Hz, 0.5H)^{b}. **^{13}C NMR** (151 MHz, $CDCl_3$) δ 163.2 – 162.6 (m)^{a,b}, 160.8 (d, *J* = 247.9 Hz)$^{a/b}$, 160.7 (d, *J* = 247.1 Hz)$^{a/b}$, 133.5 (m)^{a,b}, 132.2 (d, *J* = 2.2 Hz)$^{a/b}$, 132.1 (d, *J* = 2.2 Hz)$^{a/b}$, 125.2 – 124.7 (m)^{a,b}, 121.1 (m)^{*,a,b}116.2 (d, *J* = 24.8 Hz)$^{a/b}$, 116.2 (d, *J* = 24.7 Hz)$^{a/b}$, 102.0^{a}, 101.4^{b}, 73.5^{b}, 73.2^{a}, 68.4^{a}, 67.6^{b}, 49.6^{a}, 47.8^{b}, 27.9^{a}, 25.6^{b}, 23.5^{a}, 21.6^{b}. **LCMS** (acidic): t_R: 3.04 min, purity: >99% (254 nm). **HRMS**: $(M + H)^+$ calcd. for $C_{13}H_{14}FNO_3$: 252.1031, found: 252.1033.

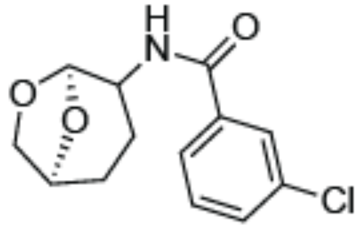

N-((1S,5R)-6,8-dioxabicyclo[3.2.1]octan-4-yl)-3-chlorobenzamide (9w)

General procedure D2 was performed, using amine **4** (128 mg, 0.991 mmol, 1.33 eq), 3-chlorobenzoic acid (117 mg, 0.747 mmol, 1.00 eq), DMF (4.7 mL), EDCI·HCl (169 mg, 0.882 mmol, 1.18 eq), DMAP (28 mg, 0.23 mmol, 0.31 eq) and DIPEA (0.20 mL, 1.1 mmol, 1.5 eq), and a reaction time of 2 d. Extraction with H_2O (10 mL) and EtOAc (3×10 mL), followed by a wash of the combined organic layers with brine (10 mL) and subsequent purification by column chromatography (EtOAc/cHex, 35%) provided the title compound (45 mg, 23%).

Diastereomeric ratio: 1.1:1.0. Proposed major diastereomer: *Endo*.

^{1}H NMR (500 MHz, $CDCl_3$) δ 7.77 (dd, *J* = 1.8, 1.8 Hz, 0.5H)$^{a/b}$, 7.75 (dd, *J* = 1.8, 1.8 Hz, 0.5H)$^{a/b}$, 7.66 (ddd, *J* = 7.8, 1.7, 1.1 Hz, 0.5H)$^{a/b}$, 7.63 (ddd, *J* = 7.8, 1.7, 1.1 Hz,0.5H)$^{a/b}$, 7.49 (ddd, *J* = 7.9, 2.1, 1.0 Hz, 0.5H)$^{a/b}$, 7.47 (ddd, *J* = 7.9, 2.0, 1.0 Hz, 0.5H)$^{a/b}$, 7.39 (dd, *J* = 7.9, 7.9 Hz, 0.5H)$^{a/b}$, 7.37 (dd, *J* = 7.9, 7.9 Hz, 0.5H)$^{a/b}$, 6.54 (d, *J* = 8.2 Hz, 0.5H)^b, 6.05 (d, *J* = 8.5 Hz, 0.5H)^a, 5.39 (s$^\Delta$, 0.5H)^a, 5.39 – 5.37 (m, 0.5H)^b, 4.57 (dd, *J* = 5.8, 3.4 Hz, 1H)$^{a/b}$, 4.31 – 4.22 (m, 0.5H)^a, 4.19 (dddd, *J* = 9.3, 4.9, 2.4, 2.4 Hz, 0.5H)^b, 4.01 (d$^\Delta$, *J* = 7.2 Hz, 0.5H)^b, 3.91 (d$^\Delta$, *J* = 7.1 Hz, 0.5H)^a, 3.89 – 3.80 (m, 1H)$^{a/b}$, 2.21 – 1.97 (m, 2H)$^{a/b}$, 1.74 – 1.51 (m, 2H)$^{a/b}$. **^{13}C NMR** (126 MHz, $CDCl_3$) δ 165.7$^{a/b}$, 165.6$^{a/b}$, 136.3$^{a/b}$, 136.2$^{a/b}$, 134.9$^{a/b}$, 134.9$^{a/b}$, 131.8$^{a/b}$, 130.1$^{a/b}$, 130.0$^{a/b}$, 127.5$^{a/b}$, 127.5$^{a/b}$, 125.2$^{a/b}$, 125.2$^{a/b}$, 102.0^a, 101.4^b, 73.5^b, 73.2^a, 68.4^a, 67.6^b, 49.6^a, 47.8^b, 27.9^a, 25.6^b, 23.6^a, 21.5^b. **LCMS** (acidic): t_R: 2.64 min, purity: >99% (254 nm). **HRMS**: $(M + H)^+$ calcd. for $C_{13}H_{14}ClNO_3$: 268.0735, found: 268.0740.

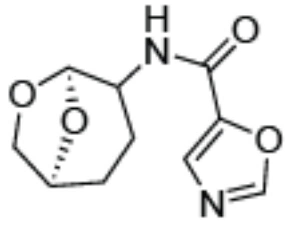

N-((1S,5R)-6,8-dioxabicyclo[3.2.1]octan-4-yl)oxazole-5-carboxamide (9x)

EDCI·HCl (463 mg, 2.42 mmol, 1.21 eq), HOBt (354 mg, 2.62 mmol, 1.31 eq), Et_3N (0.70 mL, 5.0 mmol, 2.5 eq) and 1,3-oxazole-5-carboxylic acid (251 mg, 2.22 mmol, 1.11 eq) were added to a stirring solution of amine **4** (258 mg, 2.00 mmol, 1.00 eq) in DCM (15.0 mL). The reaction mixture was stirred for 3 d at rt, concentrated *in vacuo*. The residue was subjected to column chromatography (EtOAc/cHex, 0–60%) to provide the title compound (174 mg, 39%).

Diastereomeric ratio: 1.0:1.0.

^{1}H NMR (500 MHz, $CDCl_3$) δ 8.25 – 8.20 (m, 1H)^{a,b}, 7.89 – 7.83 (m, 1H)^{a,b}, 7.36 (d, *J* = 7.5 Hz, 0.5H)^b, 6.89 (d, *J* = 7.8 Hz, 0.5H)^a, 5.43 – 5.35 (m, 1H)^{a,b}, 4.61 – 4.50 (m, 1H)^{a,b}, 4.26 – 4.18 (m, 0.5H)^a, 4.18 – 4.13 (m, 0.5H)^b, 4.00 (d$^\Delta$, *J* = 7.2 Hz, 0.5H)^b, 3.91 (d$^\Delta$, *J* = 7.1 Hz, 0.5H)^a, 3.87 – 3.81 (m, 1H)^{a,b}, 2.21 – 2.10 (m, 0.5H)^b,

2.10 – 1.94 (m, 1.5H)[a,b], 1.73 – 1.58 (m, 1.5H)[a,b], 1.55 – 1.48 (m, 0.5H)[b]. **^{13}C NMR** (126 MHz, $CDCl_3$) δ 159.9[a/b], 159.8[a/b], 150.6[a/b], 150.6[a/b], 141.6[a/b], 141.6[a/b], 136.0[a/b], 135.9[a/b], 101.9[a/b], 101.4[a/b], 73.4[a/b], 73.1[a/b], 68.4[a/b], 67.6[a/b], 48.7[a/b], 46.8[a/b], 27.9[a], 25.5[b], 23.4[a], 21.6[b]. **LCMS** (acidic): t_R: 2.28 min, purity: >99% (230 nm). **HRMS**: $(M + H)^+$ calcd. for $C_{10}H_{12}N_2O_4$: 225.0870, found: 225.0864.

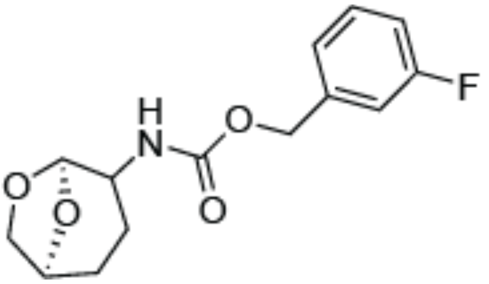

3-fluorobenzyl ((1S,5R)-6,8-dioxabicyclo[3.2.1]octan-4-yl)carbamate (10a)

Amine **4** (463 mg, 3.58 mmol, 1.00 eq) was dissolved in THF (15.0 mL) and CDI (871 mg, 5.38 mmol, 1.50 eq) was added. The reaction mixture was stirred at rt overnight, diluted with H_2O (15 mL) and extracted with EtOAc (2×30 mL). The combined organic phases were dried over Na_2SO_4, filtered, and concentrated *in vacuo*. The residue was dissolved in THF (15 mL) to give a stock solution which was used as such for the next reaction.

Half of this stock solution (theoretical amount of intermediate: 1.79 mmol, 1.00 eq) was added to a stirring solution of (3-fluorophenyl)methanol (678 mg, 5.38 mmol, 3.00 eq) in THF (3.0 mL) that was previously treated with NaH (60% in mineral oil) (215 mg, 5.38 mmol, 3.00 eq) (H_2 gas liberation; safety warning). The resulting mixture was stirred overnight at rt, diluted with H_2O (10 mL), and extracted with EtOAc (2×10 mL). The combined organic phases were dried over Na_2SO_4, filtered, and concentrated *in vacuo*. The residue was subjected to column chromatography (EtOAc/cHex, 30%) to give the title compound (143 mg, extrapolated yield starting from **4**: 28%).

Diastereomeric ratio: 1.0:1.0.

^{1}H NMR (500 MHz, $CDCl_3$) δ 7.39 – 7.27 (m, 1H)[a,b], 7.15 – 6.95 (m, 3H)[a,b], 5.41 – 5.24 (m, 1.5H)[a,b], 5.17 – 5.03 (m, 2H)[a,b], 4.81 (d, *J* = 8.6 Hz, 0.5H)[a], 4.54 – 4.50 (m, 0.5H)[a], 4.50 – 4.47 (m, 0.5H)[b], 3.95 (d^{Δ}, *J* = 7.2 Hz, 0.5H)[b], 3.84 (d^{Δ}, *J* = 6.9 Hz, 0.5H)[a], 3.83 – 3.70 (m, 2H)[a,b], 2.12 – 1.85 (m, 2H)[a,b], 1.61 – 1.38 (m, 2H)[a,b]. **^{13}C NMR** (126 MHz, $CDCl_3$) δ 163.0 (d, *J* = 246.3 Hz)[a/b], 163.0 (d, *J* = 246.2 Hz)[a/b], 155.5, 155.5, 139.2 – 139.0 (m)[a,b], 130.4 – 130.1 (m)[a,b], 123.7 – 123.4 (m)[a,b], 115.6 – 114.7 (m)[a,b], 102.2[a], 101.5[b], 73.4[b], 73.1[a], 68.3[a], 67.5[b], 66.0[a,b], 50.9[a], 49.0[b], 27.9[a], 25.4[b], 23.7[a], 21.7[b]. **LCMS** (acidic): t_R: 3.91 min, purity: >99% (254 nm). **HRMS**: $(M + H)^+$ calcd. for $C_{14}H_{16}FNO_4$: 282.1136, found: 282.1143.

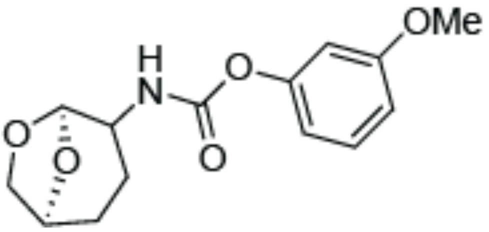

3-methoxyphenyl ((1S,5R)-6,8-dioxabicyclo[3.2.1]octan-4-yl)carbamate (10b)

Amine **4** (347 mg, 2.69 mmol, 1.00 eq) was dissolved in THF (8.0 mL) and CDI (654 mg, 4.03 mmol, 1.50 eq) was added. The reaction mixture was stirred at rt overnight, diluted with H_2O (5 mL) and extracted with EtOAc (3×10 mL). The combined organic phases were dried over Na_2SO_4, filtered, and concentrated *in vacuo*. The residue was dissolved in THF (2.0 mL) to give a stock solution which was used as such for the next reaction.

Half of this stock solution (theoretical amount of intermediate: 1.34 mmol, 1.00 eq) was added to a vial charged with 3-methoxyphenol (500 mg, 4.03 mmol, 3.00 eq) and Et_3N (0.19 mL, 1.34 mmol, 1.00 eq). The resulting mixture was stirred for 5 d at 50 °C, and subsequently for 4 h at 130 °C under microwave irradiation. The mixture was diluted with H_2O (5 mL) and extracted with EtOAc (2×5 mL). The combined organic phases were dried over Na_2SO_4, filtered, and concentrated *in vacuo*. The residue was subjected to column chromatography (EtOAc/cHex, 20%) to give the title compound (30 mg, extrapolated yield starting from **4**: 8%).

Diastereomeric ratio: 2:1.

^{1}H NMR (300 MHz, CD_3OD) δ 7.32 – 7.19 (m, 1H)^{a,b}, 6.84 – 6.73 (m, 1H)^{a,b}, 6.73 – 6.64 (m, 2H)^{a,b}, 5.36 – 5.27 (m, 1H)^{a,b}, 4.58 – 4.48 (m, 1H)^{a,b}, 4.00 (d$^{\Delta}$, *J* = 7.2 Hz, 0.7H)^{a}, 3.94 (d$^{\Delta}$, *J* = 7.1 Hz, 0.3H)^{b}, 3.85 – 3.70 (m, 4H)^{a,b}, 3.67 – 3.49 (m, 1H)^{a,b}, 2.22 – 1.81 (m, 2H)^{a,b}, 1.83 – 1.42 (m, 2H)^{a,b}. **^{13}C NMR** (76 MHz, CD_3OD) δ 162.0*^{,a,b},153.4*^{,a,b}, 130.7^{a,b}, 114.9^{a,b}, 112.1^{a,b}, 108.8^{a,b}, 102.9^{b}, 102.3^{a}, 74.7^{a}, 74.4^{b}, 69.2^{b}, 68.2^{a}, 55.9^{a,b}, 52.8^{b}, 50.5^{a}, 28.9^{b}, 26.0^{a}, 23.3^{b}, 22.2^{a}; (1 missing signal). **LCMS** (acidic): t_R: 3.63 min, purity: 97.6% (254 nm). **HRMS**: $(M + H)^+$ calcd. for $C_{14}H_{17}NO_5$: 280.1180, found: 280.1180.

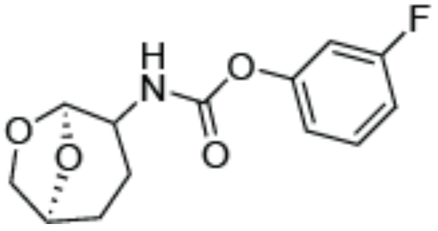

3-fluorophenyl ((1S,5R)-6,8-dioxabicyclo[3.2.1]octan-4-yl)carbamate (10c)

Amine **4** (347 mg, 2.69 mmol, 1.00 eq) was dissolved in THF (12.0 mL) and CDI (654 mg, 4.03 mmol, 1.50 eq) was added. The reaction mixture was stirred at rt overnight, diluted with H_2O (15 mL) and extracted with EtOAc (2×30 mL). The combined organic phases were dried over Na_2SO_4, filtered, and concentrated *in vacuo*. The residue was dissolved in THF (12.0 mL) to give a stock solution which was used as such for the next reaction.

Half of this stock solution (theoretical amount of intermediate: 1.34 mmol, 1.00 eq) was added to a a vial charged with 3-fluorophenol (452 mg, 4.03 mmol, 3.00 eq) and Et_3N (0.19 mL, 1.34 mmol, 1.00 eq). The resulting mixture was stirred overnight at 50 °C, diluted with H_2O (10 mL), and extracted with EtOAc (2×10 mL). The combined organic phases were dried over Na_2SO_4, filtered, and concentrated *in vacuo*. The residue was subjected to column chromatography (EtOAc/cHex, 30%) to give the title compound (71 mg, extrapolated yield starting from **4**: 20%).

Diastereomeric ratio: 1.1:1.0.

^{1}**H NMR** (300 MHz, $CDCl_3$) δ 7.32 – 7.20 (m, 1H)^{a,b}, 6.93 – 6.78 (m, 3H)^{a,b}, 5.50 (d, *J* = 8.7 Hz, 0.5H)^{b}, 5.34 (s$^{\Delta}$, 0.5H)^{a}, 5.33 – 5.29 (m, 0.5H)^{b}, 5.08 – 4.96 (m, 0.5H)^{a}, 4.54 – 4.41 (m, 1H)^{a,b}, 3.92 (d$^{\Delta}$, *J* = 7.2 Hz, 0.5H)^{b}, 3.84 (d$^{\Delta}$, 1H)^{a}, 3.80 – 3.69 (m, 2H)^{a,b}, 2.14 – 1.83 (m, 2H)^{a,b}, 1.66 – 1.36 (m, 2H)^{a,b}. ^{13}**C NMR** (76 MHz, $CDCl_3$) δ 130.3 – 129.7 (m)^{a,b}, 117.6 – 117.2 (m)^{a,b}, 112.8 – 112.1 (m)^{a,b}, 110.1 – 109.3 (m)^{a,b}, 101.8^{a}, 101.2^{b}, 73.3^{b}, 72.9^{a}, 68.3^{a}, 67.5^{b}, 50.9^{a}, 49.0^{b}, 27.7^{a}, 25.2^{b}, 23.5^{a}, 21.5^{b}; (quaternary carbons and carbonyl carbon are missing). **LCMS** (acidic): t_R: 3.72 min, purity: 96.7% (254 nm). **HRMS**: $(M + H)^+$ calcd. for $C_{13}H_{14}FNO_4$: 268.0980, found: 268.0984.

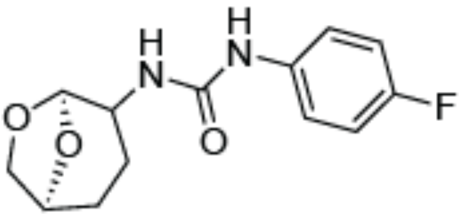

3-[(1S,5R)-6,8-dioxabicyclo[3.2.1]octan-4-yl]-1-(4-fluorophenyl)urea (11a)

General procedure E1 was performed, using amine **4** (258 mg, 2.00 mmol, 1.00 eq), 1-fluoro-4-isocyanatobenzene (374 mg, 2.00 mmol, 1.00 eq), DCM (2.5 mL), a reaction time of 1 h, and a reaction temperature of rt. This provided the title compound (323 mg, 61%).

Diastereomeric ratio: 1.2:1.0.

^{1}**H NMR** (500 MHz, $CDCl_3$) δ 7.32 – 7.20 (m, 2H)^{a,b}, 7.08 – 6.96 (m, 2H)^{a,b}, 6.48 (s, 0.5H)^{b}, 6.47 (s, 0.5H)^{a}, 5.36 (s$^{\Delta}$, 0.5H)^{a}, 5.34 – 5.31 (m, 0.5H)^{b}, 5.28 (d, *J* = 9.0 Hz, 0.5H)^{b}, 4.79 (d, *J* = 9.2 Hz, 0.5H)^{a}, 4.56 – 4.51 (m, 0.5H)^{a}, 4.50 – 4.44 (m, 0.5H)^{b}, 3.99 – 3.90 (m, 1.5H)^{a,b}, 3.86 – 3.76 (m, 1.5H)^{a,b}, 2.15 – 1.86 (m, 2H)^{a,b}, 1.68 – 1.39 (m, 2H)^{a,b}. ^{13}**C NMR** (126 MHz, $CDCl_3$) δ 157.9-161.1 (m)*, 155.1^{a,b}, 134.3 (m)^{*,a,b}, 123.3 (d, *J* = 8.0 Hz)$^{a/b}$, 123.1 (d, *J* = 8.0 Hz)$^{a/b}$, 116.1 (m)^{a,b}, 102.5^{a}, 101.8^{b}, 73.4^{b}, 73.1^{a}, 68.3^{a}, 67.5^{b}, 49.8^{a}, 48.0^{b}, 27.9^{a}, 25.5^{b}, 23.9^{a}, 21.8^{b}. **LCMS** (acidic): t_R: 3.20 min, purity: >99% (254 nm). **HRMS**: $(M + H)^+$ calcd. for $C_{13}H_{15}FN_2O_3$: 267.1140, found: 267.1142.

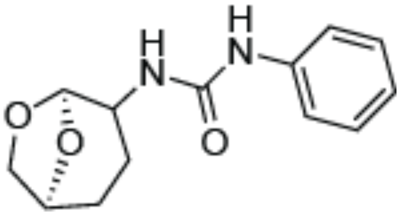

3-[(1S,5R)-6,8-dioxabicyclo[3.2.1]octan-4-yl]-1-phenylurea (11b)

General procedure E1 was performed, using amine **4** (318 mg, 2.46 mmol, 1.00 eq), isocyanatobenzene (294 mg, 2.46 mmol, 1.00 eq), DCM (2.4 mL), a reaction time of 2.5 h, and a reaction temperature of rt. This provided the title compound (289 mg, 47%).

Diastereomeric ratio: 1.2:1.0.

^{1}**H NMR** (500 MHz, DMSO-d6) δ 8.55 (s, 0.5H)^{b}, 8.43 (s, 0.5H)^{a}, 7.40 – 7.31 (m, 2H)^{a,b}7.25 – 7.18 (m, 2H)^{a,b}, 6.95 – 6.84 (m, 1H)^{a,b}, 6.45 (d, *J* = 8.9 Hz, 0.5H)^{b}, 6.06 (d, *J* = 8.6 Hz, 0.5H)^{a}, 5.26 – 5.21 (m, 0.5H)^{b}, 5.21 (s$^{\Delta}$

, 0.5H)[a], 4.58 – 4.47 (m, 1H)[a,], 3.95 (d[Δ], J = 7.2 Hz, 0.5H)[b], 3.87 (d[Δ], J = 7.1 Hz, 0.5H)[a], 3.68 – 3.52 (m, 2H)[a,b], 2.03 – 1.90 (m, 0.5H)[b], 1.90 – 1.72 (m, 1.5H)[a,b], 1.64 – 1.55 (m, 0.5H)[b], 1.52 – 1.41 (m, 1H)[a], 1.39 (dd[Δ], J = 14.0, 5.2 Hz, 0.5H)[b]. **^{13}C NMR** (126 MHz, DMSO) δ 154.4[a/b], 154.4[a/b], 140.3[b], 140.2[a], 128.7[a,b], 121.2[a], 121.1[b], 117.5[a], 117.4[b], 101.6[a], 101.0[b], 72.5[b], 72.3[a], 67.5[a], 66.5[b], 49.1[a], 46.8[b], 27.5[a], 24.8[b], 23.1[a], 21.5[b]. **LCMS** (acidic): t_R: 3.07 min, purity: >99% (254 nm). **HRMS**: $(M + H)^+$ calcd. for $C_{13}H_{16}N_2O_3$: 249.1234, found: 249.1233.

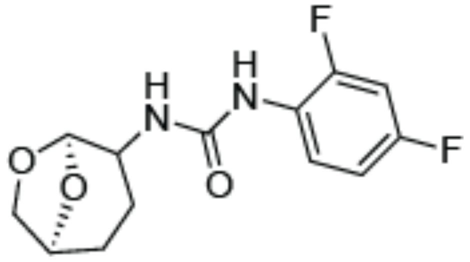

1-(2,4-difluorophenyl)-3-[(1S,5R)-6,8-dioxabicyclo[3.2.1]octan-4-yl]urea (11c)

General procedure E1 was performed, using amine **4** (332 mg, 2.57 mmol, 1.00 eq), 2,4-difluoro-1-isocyanatobenzene (399 mg, 2.57 mmol, 1.00 eq), DCM (4.5 mL), a reaction time of 1 h, and a reaction temperature of rt. This provided the title compound (508 mg, 70%).

Diastereomeric ratio: 1.5:1.0.

^{1}H NMR (500 MHz, DMSO- d_6) δ 7.93 (s, 0.4H)[b], 7.82 (s, 0.6H)[a,b], 7.29 – 7.17 (m, 1H)[a,b], 7.14 – 7.03 (m, 2H)[a,b], 6.66 (d, J = 8.9 Hz, 0.4H)[b], 6.30 (d, J = 8.6 Hz, 0.6H)[a], 5.29 – 5.21 (m, 0.4H)[b], 5.19 (s[Δ], 0.6H)[a], 4.58 – 4.48 (m, 1H)[a,b], 3.94 (d[Δ], J = 7.2 Hz, 0.4H)[b], 3.87 (d[Δ], J = 7.1 Hz, 0.6H)[a], 3.69 – 3.61 (m, 1H)[a,b], 3.61 – 3.51 (m, 1H)[a,b], 2.03 – 1.92 (m, 0.4H)[b], 1.92 – 1.72 (m, 1.6H)[a,b], 1.67 – 1.42 (m, 1.6H)[a,b], 1.42 – 1.34 (m, 0.4H)[b]. **^{13}C NMR** (126 MHz, DMSO- d_6) δ 159.0 – 156.2 (m)[a,b], 154.2[a], 154.2[b], 126.7 – 126.1 (m)[a,b], 116.2 – 115.2 (m)[a,b], 112.0 – 111.3 (m)[a,b], 101.5[a], 100.9[b], 72.5[b], 72.3[a], 67.6[a], 66.5[b], 49.6[a], 47.3[b], 27.5[a], 24.8[b], 22.9[a], 21.5[b]. **LCMS** (acidic): t_R: 2.86 min, purity: 97.0% (254 nm). **HRMS**: $(M + H)^+$ calcd. for $C_{13}H_{14}F_2N_2O_3$: 285.1045, found: 285.1043.

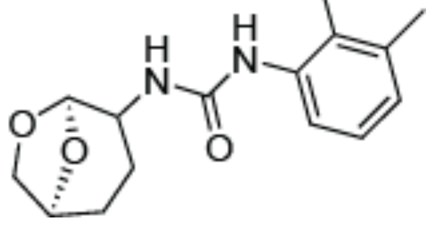

1-(2,3-dimethylphenyl)-3-[(1S,5R)-6,8-dioxabicyclo[3.2.1]octan-4-yl]urea (11d)

A solution of 1-isocyanato-2,3-dimethylbenzene (251 mg, 1.70 mmol, 1.00 eq) in DCM (1.6 mL) was added dropwise to a solution of amine **4** (220 mg, 1.70 mmol, 1.00 eq) in DCM (0.6 mL). The resulting mixture was stirred for 1 h at rt, diluted with DCM (15 mL) and washed with brine (2×10 mL). The organic layer was dried over Na_2SO_4, filtered and concentrated *in vacuo*. The residue was subjected to column chromatography (EtOAc/cHex, 30–70%) to provide the title compound (190 mg, 40%).

Diastereomeric ratio: 1.2:1.0.

^{1}H NMR (500 MHz, $CDCl_3$) δ 7.21 (d$^\Delta$, *J* = 7.8 Hz, 0.5H)[b], 7.18 (d$^\Delta$, *J* = 7.7 Hz, 0.5H)[a], 7.15 – 7.08 (m, 1H)[a,b], 7.08 – 7.03 (m, 1H)[a,b], 6.25 (s, 0.5H)[b], 6.23 (s, 0.5H)[a], 5.33 (s$^\Delta$, 0.5H)[a], 5.30 – 5.26 (m, 0.5H)[b], 5.24 (d, *J* = 9.2 Hz, 0.5H)[b], 4.74 (d, *J* = 9.2 Hz, 0.5H)[a], 4.55 – 4.46 (m, 0.5H)[a], 4.43 – 4.36 (m, 0.5H)[b], 4.03 – 3.89 (m, 1.4H)[a,b], 3.81 – 3.70 (m, 1.6H)[a,b], 2.31 (s, 1.4H)[b], 2.30 (s, 1.6H)[a], 2.20 (s, 1.4H)[b], 2.18 (s, 1.6H)[a], 2.09 – 1.99 (m, 0.5H)[b], 1.99 – 1.88 (m, 1H)[a], 1.86 – 1.76 (m, 0.5H)[b], 1.63 – 1.57 (m, 0.5H)[b], 1.57 – 1.51 (m, 0.5H)[a], 1.47 – 1.40 (m, 0.5H)[b], 1.40 – 1.33 (m, 0.5H)[a]. **^{13}C NMR** (126 MHz, $CDCl_3$) δ 156.1[a/b], 156.0[a/b], 138.5[a,b], 135.7[a/b], 135.6[a/b], 132.5[a/b], 132.3[a/b], 128.3[a/b], 128.3[a/b], 126.5[a,b], 124.1[a/b], 123.9[a/b], 102.6[a], 101.9[b], 73.3[b], 73.0[a], 68.2[a], 67.4[b], 49.9[a], 48.0[b], 28.0[a], 25.5[b], 23.9[a], 21.8[b], 20.7[a/b], 20.7[a/b], 14.1[a/b], 14.1[a/b]. **LCMS** (acidic): t_R: 3.41 min, purity: >99% (254 nm). **HRMS**: $(M + H)^+$ calcd. for $C_{15}H_{20}N_2O_3$: 277.1547, found: 277.1537.

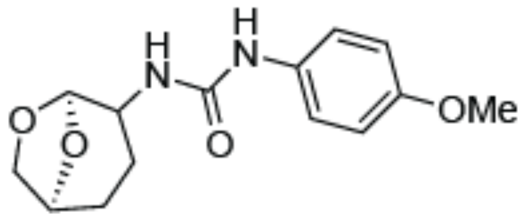

3-[(1S,5R)-6,8-dioxabicyclo[3.2.1]octan-4-yl]-1-(4-methoxyphenyl)urea (11e)

A solution of the 1-isocyanato-4-methoxybenzene (224 mg, 1.50 mmol, 1.00 eq) in DCM (1.8 mL) was added dropwise to a solution of amine **4** (194 mg, 1.50 mmol, 1.00 eq) in DCM (0.6 mL). The resulting mixture was stirred for 1 h at rt, diluted with DCM and washed with H_2O (2×). The organic layer was dried (Na_2SO_4), filtered and concentrated *in vacuo* to provide the title compound (220 mg, 53%).

Diastereomeric ratio: 1.4:1.0.

^{1}H NMR (500 MHz, $CDCl_3$) δ 7.23 – 7.14 (m, 2H)[a,b], 6.92 – 6.84 (m, 2H)[a,b], 6.42 (s, 0.4H)[b], 6.38 (s, 0.6H)[a], 5.35 (s$^\Delta$, 0.6H)[a], 5.34 – 5.27 (m, 0.8H)[b], 4.80 (d, *J* = 9.2 Hz, 0.6H)[a], 4.55 – 4.49 (m, 0.6H)[a], 4.47 – 4.41 (m, 0.4H)[a], 4.01 – 3.88 (m, 1.4H)[a,b], 3.85 – 3.72 (m, 4.6H)[a,b], 2.11 – 1.82 (m, 2H)[a,b], 1.67 – 1.51 (m, 1H)[a,b], 1.49 – 1.34 (m, 1H)[a,b]. **^{13}C NMR** (126 MHz, $CDCl_3$) δ 157.2[a], 157.1[b], 155.8[a], 155.7[b], 131.0[b], 130.8[a], 124.8[a], 124.5[b], 114.8[a,b], 102.6[a], 101.9[b], 73.3[b], 73.1[a], 68.2[a], 67.4[b], 55.7[a,b], 49.8[a], 48.0[b], 28.0[a], 25.5[b], 23.9[a], 21.8[b]. **LCMS** (acidic): t_R: 3.00 min, purity: 96.1% (254 nm). **HRMS**: $(M + H)^+$ calcd. for $C_{14}H_{18}N_2O_4$: 279.1339, found: 279.1331.

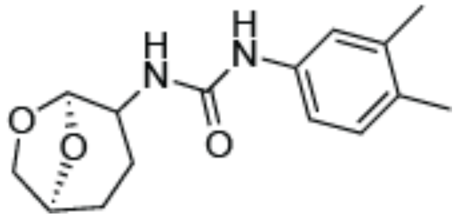

1-(3,4-dimethylphenyl)-3-[(1S,5R)-6,8-dioxabicyclo[3.2.1]octan-4-yl]urea (11f)

General procedure E1 was performed, using amine **4** (140 mg, 1.09 mmol, 1.00 eq), 4-isocyanato-1,2-dimethylbenzene (160 mg, 1.09 mmol, 1.10 eq), DCM (2.0 mL), a reaction time of 2 h, and a reaction temperature of rt. This provided the title compound (107 mg, 36%).

Diastereomeric ratio: 1.3:1.0.

^{1}H NMR (500 MHz, DMSO- d_6) δ 8.35 (s, 0.4H)[a,b], 8.22 (s, 0.6H)[a], 7.13 (d, *J* = 1.9 Hz, 0.4H)[b], 7.11 (d, *J* = 1.9 Hz, 0.6H)[a], 7.08 (dd, *J* = 2.1, 2.1 Hz, 0.4H)[b], 7.06 (dd, *J* = 2.1, 2.1 Hz, 0.6H)[a], 6.96 (s[Δ], 0.6H)[a], 6.94 (s[Δ], 0.4H)[b], 6.37 (d, *J* = 9.0 Hz, 0.4H)[b], 5.99 (d, *J* = 8.7 Hz, 0.6H)[a], 5.24 – 5.20 (m, 0.4H)[b], 5.19 (s[Δ], 0.6H)[a], 4.56 – 4.49 (m, 1H)[a,b], 3.94 (d, *J* = 7.3 Hz, 0.4H)[b], 3.86 (d, *J* = 7.3 Hz, 0.6H)[a], 3.69 – 3.53 (m, 2H)[a,b], 2.21 – 2.04 (m, 6H)[a,b], 2.00 – 1.90 (m, 0.4H)[b], 1.89 – 1.72 (m, 1.6H)[a,b], 1.63 – 1.54 (m, 0.6H)[a], 1.50 – 1.33 (m, 1.4H)[a,b]. **^{13}C NMR** (126 MHz, DMSO- d_6) δ 154.4[a], 154.4[b], 138.0[b], 137.9[a], 136.2[a,b], 129.6[a,b], 128.7[a], 128.6[b], 118.9[a], 118.8[b], 115.1[a], 115.0[b], 101.7[a], 101.1[b], 72.5[b], 72.3[a], 67.5[a], 66.5[b], 49.1[a], 46.8[b], 27.5[a], 24.9[b], 23.1[a], 21.5[b], 19.7[b], 19.6[a], 18.6[a,b]. **LCMS** (acidic): t_R: 3.62 min, purity: >99% (254 nm). **HRMS**: $(M + H)^+$ calcd. for $C_{15}H_{20}N_2O_3$: 277.1547, found: 277.1541.

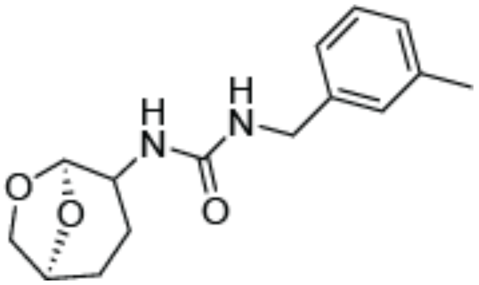

1-((1S,5R)-6,8-dioxabicyclo[3.2.1]octan-4-yl)-3-(3-methylbenzyl)urea (11g)

General procedure E2 was performed, using amine **4** (159 mg, 0.896 mmol, 1.00 eq), *m*-tolylmethanamine (109 mg, 0.896 mmol, 1.00 eq), THF (4.0 mL), a reaction time of overnight, and a reaction temperature of rt. Extraction with H_2O (5 mL) and EtOAc (2×9 mL) provided the title compound (30 mg, 12%).

Diastereomeric ratio: 1.5:1.0.

^{1}H NMR (300 MHz, $CDCl_3$) δ 7.24 – 7.15 (m, 1H)[a,b], 7.12 – 7.02 (m, 3H)[a,b], 5.32 (s[Δ], 0.6H)[a], 5.29 – 5.23 (m, 0.4H)[b], 5.03 (d, *J* = 9.3 Hz, 0.4H)[b], 4.89 (dd, *J* = 5.5, 5.5 Hz, 0.4H)[b], 4.84 (dd, *J* = 5.2, 5.2 Hz, 0.6H)[a], 4.58 (d, *J* = 9.3 Hz, 0.4H)[b], 4.53 – 4.46 (m, 0.6H)[a], 4.46 – 4.38 (m, 0.4H)[b], 4.35 – 4.24 (m, 2H)[a,b], 3.98 – 3.68 (m, 3H)[a,b], 2.33 (s, 1.2H)[b], 2.32 (s, 1.8H)[a], 2.07 – 1.75 (m, 2H)[a,b], 1.62 – 1.31 (m, 2H)[a,b]. **^{13}C NMR** (76 MHz, $CDCl_3$) δ 157.2[a/b], 157.2[a/b], 138.9[a/b], 138.9[a/b], 138.4[a/b], 138.3[a/b], 128.6[b], 128.6[a], 128.3[a,b], 128.2[b], 128.1[a], 124.6[b], 124.6[a], 102.6[a], 101.9[b], 73.2[b], 73.0[a], 68.1[a], 67.3[b], 49.7[a], 47.9[b], 44.7[b], 44.5[a], 27.9[a], 25.3[b], 23.9[a], 21.7[b], 21.4[a,b]. **LCMS** (acidic): t_R: 3.47 min, purity: 97.7% (254 nm). **HRMS**: $(M + H)^+$ calcd. for $C_{15}H_{20}N_2O_3$: 277.1547, found: 277.1552.

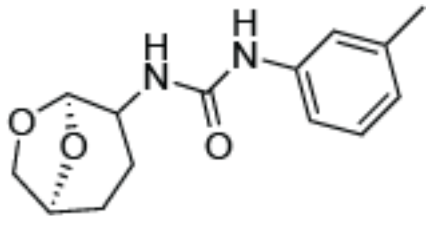

1-((1S,5R)-6,8-dioxabicyclo[3.2.1]octan-4-yl)-3-(m-tolyl)urea (11h)

General procedure E2 was performed, using amine **4** (265 mg, 1.49 mmol, 1.00 eq), *m*-toluidine (160 mg, 1.49 mmol, 1.00 eq), THF (6.6 mL), a reaction time of overnight, and a reaction temperature of rt. Extraction with H_2O (6 mL) and EtOAc (12 mL) provided the title compound (50 mg, 13%).

Diastereomeric ratio: 1.1:1.0.

^{1}H NMR (300 MHz, $CDCl_3$) δ 7.23 – 7.02 (m, 3H)^{a,b}, 6.96 – 6.78 (m, 2H)^{a,b}, 5.66 (d, *J* = 9.3 Hz, 0.5H)^{b}, 5.39 (s$^{\Delta}$, 0.5H)^{a}, 5.36 – 5.30 (m, 0.5H)^{b}, 5.20 (d, *J* = 9.2 Hz, 0.5H)^{a}, 4.57 – 4.50 (m, 0.5H)^{a}, 4.50 – 4.42 (m, 0.5H)^{b}, 4.05 – 3.89 (m, 1.5H)^{a,b}, 3.88 – 3.73 (m, 1.5H)^{a,b}, 2.31 (s, 1.5H)^{b}, 2.31 (s, 1.5H)^{a}, 2.18 – 1.83 (m, 2H)^{a,b}, 1.68 – 1.35 (m, 2H)^{a,b}. **^{13}C NMR** (76 MHz, $CDCl_3$) δ 155.3$^{a/b}$, 155.2$^{a/b}$, 139.4^{a,b}, 138.6$^{a/b}$, 138.4$^{a/b}$, 129.3^{a,b}, 124.8$^{a/b}$, 124.7$^{a/b}$, 121.8$^{a/b}$, 121.6$^{a/b}$, 118.1$^{a/b}$, 117.9$^{a/b}$, 102.6^{a}, 101.9^{b}, 73.4$^{a/b}$, 73.1$^{a/b}$, 68.2^{a}, 67.4^{b}, 49.8^{a}, 47.9^{b}, 27.9^{a}, 25.5^{b}, 23.9^{a}, 21.8^{b}, 21.6^{a,b}. **LCMS** (acidic): t_R: 3.53 min, purity: 95.4% (254 nm). **HRMS**: $(M + H)^+$ calcd. for $C_{14}H_{18}N_2O_3$: 263.1390, found: 263.1398.

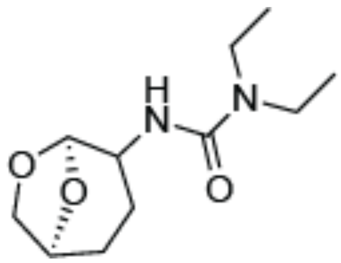

3-((1S,5R)-6,8-dioxabicyclo[3.2.1]octan-4-yl)-1,1-diethylurea (11i)

General procedure E2 was performed, using amine **4** (265 mg, 1.50 mmol, 1.00 eq), diethylamine (218 mg, 2.98 mmol, 2.00 eq), THF (6.6 mL), a reaction time of 2 d, and a reaction temperature of rt. Extraction with H_2O (6 mL) and EtOAc (12 mL) provided the title compound (104 mg, 31%).

Diastereomeric ratio: 1.1:1.0.

^{1}H NMR (300 MHz, $CDCl_3$) δ 5.32 (s$^{\Delta}$, 0.5H)^{a}, 5.31 – 5.24 (m, 0.5H)^{b}, 4.82 (d, *J* = 8.9 Hz, 0.5H)^{b}, 4.56 – 4.44 (m, 1H)^{a,b}, 4.25 (d, *J* = 9.2 Hz, 0.5H)^{a}, 4.03 – 3.74 (m, 3H)^{a,b}, 3.36 – 3.14 (m, 4H)^{a,b}, 2.11 – 1.84 (m, 2H)^{a,b}, 1.67 – 1.37 (m, 2H)^{a,b}, 1.19 – 1.06 (m, 6H)^{a,b}. **^{13}C NMR** (76 MHz, $CDCl_3$) δ 156.5$^{a/b}$, 156.4$^{a/b}$, 102.9^{a}, 102.3^{b}, 73.3^{b}, 73.1^{a}, 68.2^{a}, 67.3^{b}, 50.1^{a}, 48.2^{b}, 41.4$^{a/b}$, 41.3$^{a/b}$, 28.1^{a}, 25.7^{b}, 24.2^{a}, 21.8^{a}, 14.0$^{a/b}$, 13.9$^{a/b}$. **LCMS** (acidic): t_R: 3.47 min, purity: >99% (254 nm). **HRMS**: $(M + H)^+$ calcd. for $C_{11}H_{20}N_2O_3$: 229.1547, found: 229.1553.

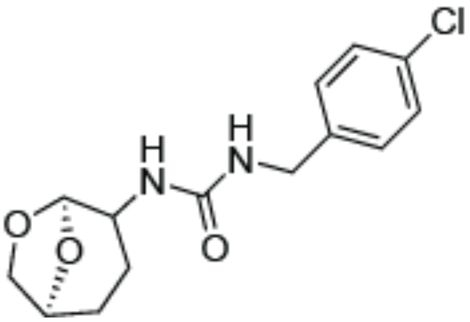

1-((1S,5R)-6,8-dioxabicyclo[3.2.1]octan-4-yl)-3-(4-chlorobenzyl)urea (11j)

General procedure E2 was performed, using amine **4** (191 mg, 1.08 mmol, 1.00 eq), (4-chlorophenyl)methanamine (152 mg, 1.08 mmol, 1.00 eq), THF (5.0 mL), Et_3N (0.40 mL, 2.9 mmol, 2.7 eq), a reaction time of 3 d, and a reaction temperature of rt. Extraction with H_2O (5 mL) and EtOAc (3×5 mL) and subsequent purification by column chromatography (EtOAc/cHex, 0–80%) provided the title compound (200 mg, 63%).

Diastereomeric ratio: 1.0:1.0.

^{1}H NMR (300 MHz, $CDCl_3$) δ 7.33 – 7.22 (m, 2H)^{a,b}, 7.22 – 7.12 (m, 2H)^{a,b}, 5.30 (s$^{\Delta}$, 0.5H)^{a}, 5.28 – 5.24 (m, 0.5H)^{b}, 5.19 (d, *J* = 9.3 Hz, 0.5H)^{b}, 5.15 – 5.04 (m, 1H)^{a,b}, 4.76 (d, *J* = 9.3 Hz, 0.5H)^{a}, 4.54 – 4.45 (m, 0.5H)^{a}, 4.45 – 4.39 (m, 0.5H)^{b}, 4.35 – 4.19 (m, 2H)^{a,b}, 3.92 (d$^{\Delta}$, *J* = 7.2 Hz, 0.5H)^{b}, 3.90 – 3.68 (m, 2.5H)^{a,b}, 2.06 – 1.77 (m, 2H)^{a,b}, 1.62 – 1.32 (m, 2H)^{a,b}. **^{13}C NMR** (76 MHz, $CDCl_3$) δ 157.4$^{a/b}$, 157.3$^{a/b}$, 137.9$^{a/b}$, 137.8$^{a/b}$, 133.2$^{a/b}$, 133.1$^{a/b}$, 128.9$^{a/b}$, 128.9$^{a/b}$, 128.9$^{a/b}$, 128.8$^{a/b}$, 102.7^{a}, 102.0^{b}, 73.3$^{a/b}$, 73.0$^{a/b}$, 68.2^{b}, 67.4^{a}, 49.9^{a}, 48.0^{b}, 43.9$^{a/b}$, 43.8$^{a/b}$, 27.9^{a}, 25.4^{b}, 24.0^{a}, 21.8^{b}. **LCMS** (acidic): t_R: 3.57 min, purity: >99% (230 nm). **HRMS**: $(M + H)^+$ calcd. for $C_{14}H_{17}ClN_2O_3$: 297.1001, found: 297.1008.

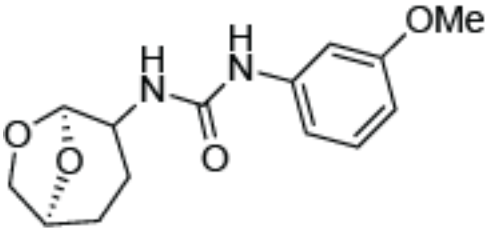

1-((1S,5R)-6,8-dioxabicyclo[3.2.1]octan-4-yl)-3-(3-methoxyphenyl)urea (11k)

General procedure E2 was performed, using amine **4** (319 mg, 1.79 mmol, 1.25 eq), 3-methoxyaniline (176 mg, 1.43 mmol, 1.00 eq), THF (8.0 mL), Et_3N (0.25 mL, 1.8 mmol, 1.3 eq), a reaction time of 2 d, and a reaction temperature of 50 °C. Extraction with H_2O (8 mL) and EtOAc (2×10 mL) and subsequent purification by column chromatography (EtOAc/cHec, 60%) provided the title compound (158 mg, 32%).

Diastereomeric ratio: 1.1:1.0.

^{1}H NMR (300 MHz, $CDCl_3$) δ 7.24 – 7.10 (m, 1H)^{a,b}, 7.06 – 6.92 (m, 2H)^{a,b}, 6.83 – 6.71 (m, 1H)^{a,b}, 6.67 – 6.55 (m, 1H)^{a,b}, 5.74 (d, *J* = 9.2 Hz, 0H)^{b}, 5.39 (s$^{\Delta}$, 0.5H)^{a}, 5.37 – 5.32 (m, 0.5H)^{b}, 5.29 (d, *J* = 9.2 Hz, 0.5H)^{a}, 4.60 – 4.50 (m, 0.5H)^{a}, 4.50 – 4.43 (m, 0.5H)^{b}, 4.05 – 3.89 (m, 1.5H)^{a,b}, 3.89 – 3.70 (m, 4.5H)^{a,b}, 2.16 – 1.82 (m, 2H)^{a,b}, 1.81 – 1.34 (m, 2H)^{a,b}. **^{13}C NMR** (76 MHz, $CDCl_3$) δ 160.6$^{a/b}$, 160.5$^{a/b}$, 155.2$^{a/b}$, 155.1$^{a/b}$, 140.0$^{a/b}$, 139.9$^{a/b}$, 130.1$^{a/b}$, 130.1$^{a/b}$, 112.8$^{a/b}$, 112.7$^{a/b}$, 109.7^{a,b}, 106.3$^{a/b}$, 106.1$^{a/b}$, 102.6^{a}, 101.9^{b}, 73.4^{b}, 73.1^{a}, 68.2^{a},

67.5[b], 55.4[a,b], 49.7[a], 47.9[b], 27.9[a], 25.5[b], 23.9[a], 21.8[b]. **LCMS** (acidic): t_R: 3.30 min, purity: >99% (254 nm). **HRMS**: $(M + H)^+$ calcd. for $C_{14}H_{18}N_2O_4$: 279.1339, found: 279.1352.

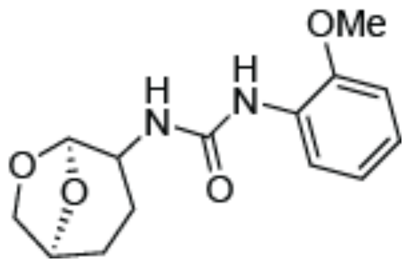

1-((1S,5R)-6,8-dioxabicyclo[3.2.1]octan-4-yl)-3-(2-methoxyphenyl)urea (11l)

General procedure E2 was performed, using amine **4** (159 mg, 0.896 mmol, 2.00 eq), 2-methoxyaniline (55 mg, 0.45 mmol, 1.00 eq), THF (4.5 mL), a reaction time of overnight, and a reaction temperature of 50 °C. Extraction with H_2O (20 mL) and EtOAc (2×20 mL), followed by a wash of the combined organic layers with H_2O (10 mL) and brine (10 mL) and subsequent purification by column chromatography (EtOAc/cHex, 0–60%) provided the title compound (113 mg, 37%).

Diastereomeric ratio: 1.1:1.0.

^{1}H NMR (300 MHz, $CDCl_3$) δ 8.07 – 7.98 (m, 1H)[a,b], 7.10 – 6.88 (m, 3H)[a,b], 6.88 – 6.80 (m, 1H)[a,b], 5.41 (s[Δ], 0.5H)[a], 5.36 (s[Δ], 0.5H)[b], 4.59 – 4.53 (m, 0.5H)[a], 4.53 – 4.48 (m, 0.5H)[b], 4.05 – 3.90 (m, 1.5H)[a,b], 3.89 – 3.77 (m, 4.5H)[a,b], 2.17 – 1.88 (m, 2H)[a,b], 1.73 – 1.40 (m, 2H)[a,b]. **^{13}C NMR** (76 MHz, $CDCl_3$) δ 154.6[a/b], 154.5[a/b], 148.1[a,b], 128.4[a,b], 122.8[a/b], 122.7[a/b], 121.4[a/b], 121.3[a/b], 119.6[a/b], 119.5[a/b], 110.2[a/b], 110.2[a/b], 102.8[a], 101.9[b], 73.4[b], 73.1[a], 68.2[a], 67.5[b], 55.7[a/b], 55.7[a/b], 49.6[a], 47.9[b], 28.0[a], 25.5[b], 24.0[a], 21.8[b]. **LCMS** (acidic): t_R: 3.41 min, purity: >99% (254 nm). **HRMS**: $(M + H)^+$ calcd. for $C_{14}H_{18}N_2O_4$: 279.1339, found: 279.1341.

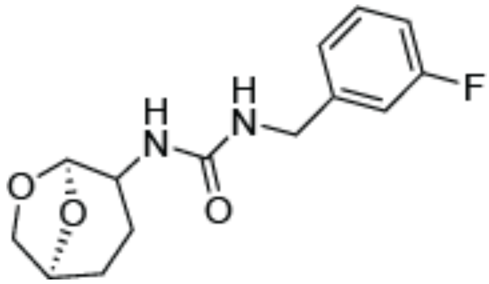

1-((1S,5R)-6,8-dioxabicyclo[3.2.1]octan-4-yl)-3-(3-fluorobenzyl)urea (11m)

General procedure E2 was performed, using amine **4** (159 mg, 0.896 mmol, 1.00 eq), (3-fluorophenyl)methanamine (112 mg, 0.896 mmol, 1.00 eq), THF (4.0 mL), a reaction time of overnight, and a reaction temperature of rt. Extraction with H_2O (5 mL) and EtOAc (9 mL) and subsequent purification by column chromatography (EtOAc/cHex, 80%) provided the title compound (35 mg, 14%).

Diastereomeric ratio: 1.5:1.0.

^{1}H NMR (500 MHz, $CDCl_3$) δ 7.33 – 7.17 (m, 1H)[a,b], 7.11 – 6.85 (m, 3H)[a,b], 5.31 (s[Δ], 0.6H)[a], 5.27 (s[Δ], 0.4H)[b], 4.57 – 4.47 (m, 0.6H)[a], 4.47 – 4.39 (m, 04H)[b], 4.40 – 4.24 (m, 2H)[a,b], 3.93 (d, J = 7.1 Hz, 0.4H)[b], 3.90 – 3.66 (m, 2.6H)[a,b], 2.11 – 1.76 (m, 2H)[a,b], 1.64 – 1.35 (m, 2H)[a,b]. **^{13}C NMR** (126 MHz, $CDCl_3$) δ 163.2 (d, J = 246.4

Hz)^{b}, 163.1 (d, J = 246.1 Hz)^{a,b}, 157.4^{a,b}, 142.4 – 141.7 (m)^{a,b}, 130.7 – 130.0 (m)^{a,b}, 123.4 – 122.8 (m)^{a,b}, 114.7 – 114.0 (m)^{a,b}, 102.7^{a}, 101.9^{b}, 73.3^{b}, 73.1^{a}, 68.2^{a}, 67.4^{b}, 50.0^{a}, 48.1^{b}, 44.2^{b}, 44.1^{a}, 28.0^{a}, 25.5^{b}, 24.0^{a}, 21.9^{b}. **LCMS** (acidic): t_R: 3.28 min, purity: >99% (254 nm). **HRMS**: (M + H)$^+$ calcd. for $C_{14}H_{17}FN_2O_3$: 281.1296, found: 281.1301.

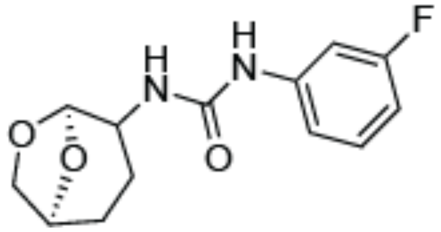

1-((1S,5R)-6,8-dioxabicyclo[3.2.1]octan-4-yl)-3-(3-fluorophenyl)urea (11n)

General procedure E2 was performed, using amine **4** (199 mg, 1.12 mmol, 1.00 eq), 3-fluoroaniline (124 mg, 1.12 mmol, 1.00 eq), THF (5.0 mL), a reaction time of 1 d, and a reaction temperature of 52 °C. Extraction with H_2O (5 mL) and EtOAc (9 mL) and subsequent purification by column chromatography (EtOAc/cHex, 60%) provided the title compound (96 mg, 32%).

Diastereomeric ratio: 2:1.

^{1}H NMR (300 MHz, DMSO) δ 8.78 (s, 0.3H)^{b}, 8.67 (s, 0.7H)^{a}, 7.51 – 7.38 (m, 1H)^{a,b}, 7.30 – 7.18 (m, 1H)^{a,b}, 7.02 – 6.92 (m, 1H)^{a,b}, 6.76 – 6.64 (m, 1H)^{a,b}, 6.52 (d, J = 9.0 Hz, 0.3H)^{b}, 6.15 (d, J = 8.6 Hz, 0.7H)^{a}, 5.25 – 5.22 (m, 0.3H)^{b}, 5.21 (s$^{\Delta}$, 0.7H)^{a}, 4.59 – 4.49 (m, 1H)^{a,b}, 3.95 (d$^{\Delta}$, J = 7.2 Hz, 0.3H)^{b}, 3.87 (d$^{\Delta}$, J = 7.1 Hz, 0.7H)^{a}, 3.70 – 3.54 (m, 2H)^{a,b}, 2.06 – 1.69 (m, 2H)^{a,b}, 1.65 – 1.32 (m, 2H)^{a,b}. **^{13}C NMR** (76 MHz, DMSO) δ 162.5 (d, J = 240.1 Hz)^{b}, 162.5 (d, J = 240.1 Hz)^{a}, 154.2^{a}, 154.2^{b}, 142.3 – 141.9 (m)^{a,b}, 130.4 – 130.1 (m)^{a,b}, 113.3 (d, J = 2.7 Hz)^{a}, 113.2 (d, J = 2.8 Hz)^{b}, 107.5 (d, J = 21.2 Hz)^{a}, 107.4 (d, J = 21.5 Hz)^{b}, 104.2 (d, J = 26.5 Hz)^{a}, 104.1 (d, J = 26.5 Hz)^{b}, 101.5^{a}, 100.9^{b}, 72.5^{b}, 72.3^{a}, 67.6^{a}, 66.6^{b}, 49.1^{a}, 46.9^{b}, 27.5^{a}, 24.8^{a,b}, 23.0^{a}, 21.5^{b}. **LCMS** (acidic): t_R: 2.49 min, purity: >99% (254 nm). **HRMS**: (M + H)$^+$ calcd. for $C_{13}H_{15}FN_2O_3$: 267.1140, found: 267.1142.

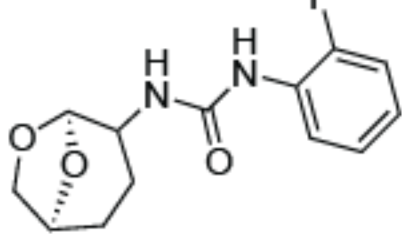

1-((1S,5R)-6,8-dioxabicyclo[3.2.1]octan-4-yl)-3-(2-fluorophenyl)urea (11o)

General procedure E2 was performed, using amine **4** (159 mg, 0.896 mmol, 1.00 eq), 2-fluoroaniline (100 mg, 0.896 mmol, 1.00 eq), THF (4.0 mL), a reaction time of 1 h, and a reaction temperature of rt. Extraction with H_2O (10 mL) and EtOAc (2×10 mL) and subsequent purification by column chromatography (EtOAc/cHex, 35%) provided the title compound (72 mg, 30%).

Diastereomeric ratio: 1.2:1.0.

^{1}H NMR (300 MHz, $CDCl_3$) δ 8.10 – 7.91 (m, 1H)^{a,b}, 7.25 – 7.14 (m, 1H)^{a,b}, 7.13 – 6.89 (m, 3H)^{a,b}, 5.94 (d, *J* = 9.2 Hz, 0.5H)^{b}, 5.54 (d, *J* = 9.3 Hz, 0.5H)^{a}, 5.41 (s$^{\Delta}$, 0.5H)^{a}, 5.36 (s$^{\Delta}$, 0.5H)^{b}, 4.59 – 4.52 (m, 0.5H)^{a}, 4.52 – 4.43 (m, 0.5H)^{b}, 4.12 – 3.90 (m, 1.5H)^{a,b}, 3.90 – 3.73 (m, 1.5H)^{a,b}, 2.19 – 1.88 (m, 2H)^{a,b}, 1.72 – 1.36 (m, 2H)^{a,b}. **^{13}C NMR** (76 MHz, $CDCl_3$) δ 154.8$^{a/b}$, 154.7$^{a/b}$, 153.0 (d, *J* = 242.5 Hz)$^{a/b}$, 153.0 (d, *J* = 242.5 Hz)$^{a/b}$, 127.5 – 127.0 (m)^{a,b}, 125.0 – 124.5 (m)^{a,b}, 123.6 – 123.1 (m)^{a,b}, 122.1 – 121.8 (m)^{a,b}, 115.1 (d, *J* = 19.4 Hz)$^{a/b}$, 115.1 (d, *J* = 19.4 Hz)$^{a/b}$, 102.7^{a}, 101.9^{b}, 73.4^{b}, 73.1^{a}, 68.2^{a}, 67.4^{b}, 49.6^{a}, 47.9^{b}, 27.9^{a}, 25.5^{a}, 23.8^{a}, 21.8^{b}. **LCMS** (acidic): t_R: 3.39 min, purity: >99% (254 nm). **HRMS**: $(M + H)^+$ calcd. for $C_{13}H_{15}FN_2O_3$: 267.1140, found: 267.1143.

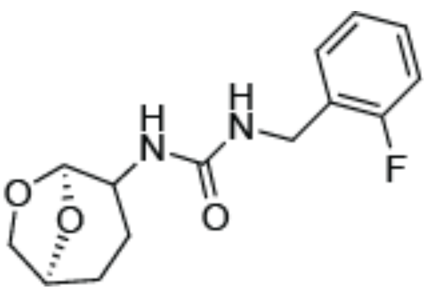

1-((1S,5R)-6,8-dioxabicyclo[3.2.1]octan-4-yl)-3-(2-fluorobenzyl)urea (11p)

General procedure E2 was performed, using amine **4** (159 mg, 0.896 mmol, 1.00 eq), (2-fluorophenyl)methanamine (123 mg, 0.986 mmol, 1.10 eq), THF (4.0 mL), a reaction time of 1 h, and a reaction temperature of rt. Extraction with H_2O (10 mL) and EtOAc (3×10 mL) and subsequent purification by reverse phase column chromatography (0.1% HCOOH in MeCN and 0.1% HCOOH in H_2O, 5-95%) provided the title compound (64 mg, 25%).

Diastereomeric ratio: 1.0:1.0.

^{1}H NMR (600 MHz, $CDCl_3$) δ 7.34 (ddd, *J* = 7.6, 7.6, 1.7 Hz, 0.5H)$^{a/b}$, 7.32 (ddd, *J* = 7.6, 7.6, 1.7 Hz, 0.5H)$^{a/b}$, 7.25 – 7.19 (m, 1H)^{a,b}, 7.12 – 7.05 (m, 1H)^{a,b}, 7.05 – 6.97 (m, 1H)^{a,b}, 5.30 (s$^{\Delta}$, 0.5H)^{a}, 5.28 – 5.23 (m, 0.5H)^{b}, 5.23 – 4.58 (m, 2H)^{a,b}, 4.53 – 4.46 (m, 0.5H)^{a}, 4.46 – 4.41 (m, 0.5H)^{b}, 4.41 – 4.33 (m, 2H), 3.92 (d$^{\Delta}$, *J* = 7.2 Hz, 0.5H)^{b}, 3.89 – 3.81 (m, 1H)^{a,b}, 3.80 (d$^{\Delta}$, *J* = 7.1 Hz, 0.5H)^{a}, 3.79 – 3.72 (m, 1H)^{a,b}, 2.06 – 1.84 (m, 2H)^{a,b}, 1.57 – 1.51 (m, 1H)^{a,b}, 1.46 – 1.36 (m, 1H)^{a,b}. **^{13}C NMR** (151 MHz, $CDCl_3$) δ 161.09 (d, *J* = 244.8 Hz)$^{a/b}$, 161.06 (d, *J* = 245.9 Hz)$^{a/b}$, 157.3$^{a/b}$, 157.2$^{a/b,}$130.4 – 130.0 (m)^{a,b}, 129.20 (d, *J* = 8.2 Hz)$^{a/b}$, 129.15 (d, *J* = 8.2 Hz)$^{a/b}$, 126.4 – 126.2 (m)^{a,b}, 124.42 (d, *J* = 3.5 Hz)$^{a/b}$, 124.39 (d, *J* = 3.5 Hz)$^{a/b}$, 115.41 (d, *J* = 21.3 Hz)$^{a/b}$, 115.38 (d, *J* = 21.4 Hz)$^{a/b}$, 102.7^{a}, 102.0^{b}, 73.3^{b}, 73.1^{a}, 68.2^{a}, 67.4^{b}, 49.9^{a}, 48.0^{b}, 38.6 (d, *J* = 3.8 Hz)$^{a/b}$, 38.5 (d, *J* = 3.9 Hz)$^{a/b}$, 28.0^{a}, 25.5^{b}, 24.0^{a}, 21.8^{b}. **LCMS** (acidic): t_R: 3.23 min, purity: >99% (254 nm). **HRMS**: $(M + H)^+$ calcd. for $C_{14}H_{17}FN_2O_3$: 281.1296, found: 281.1297.

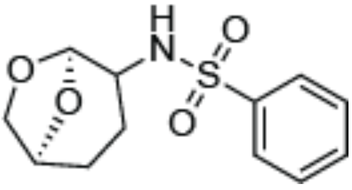

N-[(1S,5R)-6,8-dioxabicyclo[3.2.1]octan-4-yl]benzenesulfonamide (12a and 12b)

General procedure F was performed, using amine **4** (194 mg, 1.50 mmol, 1.20 eq), benzenesulfonyl chloride (265 mg, 1.50 mmol, 1.00 eq), DCM (1.6 mL), Et_3N (0.42 mL, 3.0 mmol, 2.0 eq), and a reaction time of 3.5 h. Extraction with 1.0 M aq. HCl (15 mL) and DCM (15 mL), followed by a wash of the combined organic layers with satd. aq. $NaHCO_3$ (15 mL) and subsequent purification by column chromatography (EtOAc/cHex, 33%) provided title compound **12a** (25 mg, 7%) and **12b** (17 mg, 5%).

12a: Diastereomeric ratio: >19:1. Proposed major diastereomer: *Exo*.

^{1}H NMR (500 MHz, $CDCl_3$) δ 7.98 – 7.82 (m, 2H), 7.63 – 7.54 (m, 1H), 7.54 – 7.48 (m, 2H), 5.10 (d, *J* = 9.9 Hz, 1H), 4.98 (s$^{\Delta}$, 1H), 4.49 – 4.41 (m, 1H), 3.88 (d$^{\Delta}$, *J* = 7.2 Hz, 1H), 3.78 – 3.72 (m, 1H), 3.35 – 3.29 (m, 1H), 2.01 – 1.89 (m, 2H), 1.55 – 1.51 (m, 1H), 1.44 – 1.39 (m, 1H). **^{13}C NMR** (126 MHz, $CDCl_3$) δ 141.2, 132.9, 129.4, 127.0, 101.2, 73.2, 67.6, 51.1, 25.0, 22.0. **LCMS** (acidic): t_R: 3.23 min, purity: >99% (230 nm). **HRMS**: $(M + H)^+$ calcd. for $C_{12}H_{15}NO_4S$: 270.0795, found: 270.0797.

12b: Diastereomeric ratio: 7:1. Proposed major diastereomer: *Endo*.

^{1}H NMR (500 MHz, $CDCl_3$) δ 7.92 – 7.85 (m, 2H), 7.62 – 7.56 (m, 1H), 7.55 – 7.48 (m, 2H), 5.05 (s$^{\Delta}$, 1H), 4.64 (d, *J* = 10.0 Hz, 1H), 4.49 – 4.41 (m, 1H), 3.81 (d$^{\Delta}$, *J* = 7.2 Hz, 1H), 3.78 – 3.72 (m, 1H), 3.42 – 3.30 (m, 1H), 1.89 – 1.78 (m, 2H), 1.56 – 1.40 (m, 2H). **^{13}C NMR** (126 MHz, $CDCl_3$) δ 141.5, 132.9, 129.4, 127.0, 101.9, 101.2, 73.0, 68.3, 53.1, 28.0, 24.7. **LCMS** (acidic): t_R: 3.22 min, purity: >99% (230 nm). **HRMS**: $(M + H)^+$ calcd. for $C_{12}H_{15}NO_4S$: 270.0795, found: 270.0795.

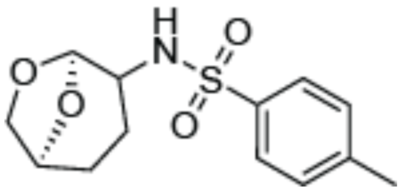

N-[(1S,5R)-6,8-dioxabicyclo[3.2.1]octan-4-yl]-4-methylbenzene-1-sulfonamide (12c and 12d)

General procedure F was performed, using amine **4** (193 mg, 1.49 mmol, 1.20 eq), 4-methylbenzenesulfonyl chloride (285 mg, 1.49 mmol, 1.00 eq), DCM (1.2 mL), Et_3N (0.42 mL, 3.0 mmol, 2.0 eq), and a reaction time of overnight. Extraction with 1.0 M aq. HCl (30 mL) and DCM (30 mL), followed by a wash of the combined organic layers with satd. aq. $NaHCO_3$ (20 mL) and subsequent purification by column chromatography (EtOAc/cHex, 30%) provided title compound **12c** (23 mg, 6%) and **12d** (24 mg, 7%).

12c: Diastereomeric ratio: >19:1. Proposed major diastereomer: *Exo*.

^{1}H NMR (600 MHz, $CDCl_3$) δ 7.78 – 7.73 (m, 2H), 7.32 – 7.27 (m, 2H), 5.09 (d, *J* = 8.5 Hz, 1H), 4.97 (s$^{\Delta}$, 1H), 4.50 – 4.40 (m, 1H), 3.87 (d$^{\Delta}$, *J* = 7.3 Hz, 1H), 3.78 – 3.70 (m, 1H), 3.34 – 3.25 (m, 1H), 2.42 (s, 3H), 2.02 – 1.87 (m, 2H), 1.57 – 1.37 (m, 2H). **^{13}C NMR** (151 MHz, $CDCl_3$) δ 143.7, 138.2, 130.0, 127.0, 101.2, 73.2, 67.6, 51.0, 25.1, 22.0, 21.7. **LCMS** (acidic): t_R: 3.55 min, purity: >99% (230 nm). **HRMS**: $(M + H)^+$ calcd. for $C_{13}H_{17}NO_4S$: 284.0951, found: 284.0952.

12d: Diastereomeric ratio: >19:1. Proposed major diastereomer: *Endo*.

^{1}H NMR (600 MHz, $CDCl_3$) δ 7.80 – 7.73 (m, 2H), 7.33 – 7.27 (m, 2H), 5.05 (s$^{\Delta}$, 1H), 4.68 (d, *J* = 10.0 Hz, 1H), 4.48 – 4.42 (m, 1H), 3.80 (d$^{\Delta}$, *J* = 7.2 Hz, 1H), 3.76 – 3.72 (m, 1H), 3.37 – 3.31 (m, 1H), 2.42 (s, 3H), 1.89 – 1.78 (m, 2H), 1.57 – 1.40 (m, 2H). **^{13}C NMR** (151 MHz, $CDCl_3$) δ 143.7, 138.5, 130.0, 127.0, 101.9, 73.0,

68.3, 53.0, 28.0, 24.7, 21.7. **LCMS** (acidic): t_R: 3.55 min, purity: >99% (230 nm). **HRMS**: $(M + H)^+$ calcd. for $C_{13}H_{17}NO_4S$: 284.0951, found: 284.0947.

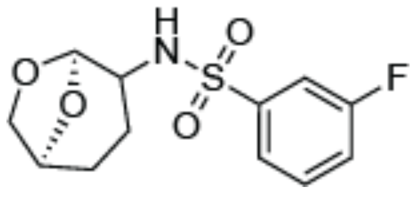

N-[(1S,5R)-6,8-dioxabicyclo[3.2.1]octan-4-yl]-3-fluorobenzene-1-sulfonamide (12e)

General procedure F was performed, using amine **4** (310 mg, 2.40 mmol, 1.20 eq), 3-fluorobenzenesulfonyl chloride (467 mg, 2.40 mmol, 1.00 eq), DCM (2.0 mL), Et_3N (0.67 mL, 4.8 mmol, 2.0 eq), and a reaction time of overnight. Extraction with 1.0 M aq. HCl (20 mL) and DCM (20 mL), followed by a wash of the combined organic layers with satd. aq. $NaHCO_3$ (20 mL) and subsequent purification by column chromatography (EtOAc/cHex, 20%) provided the title compound (200 mg, 35%).

Diastereomeric ratio: >19:1. Proposed major diastereomer: *Endo*.

^{1}H NMR (500 MHz, $CDCl_3$) δ 7.71 (ddd, *J* = 7.8, 1.7, 1.1 Hz, 1H), 7.62 (ddd, *J* = 8.2, 2.1, 2.1 Hz, 1H), 7.54 (ddd, *J* = 8.1, 8.0, 5.3 Hz, 1H), 7.32 (dddd, *J* = 8.3, 8.3, 2.5, 0.9 Hz, 1H), 5.13 (s^Δ, 1H), 4.71 (d, *J* = 10.1 Hz, 1H), 4.56 – 4.46 (m, 1H), 3.85 (d^Δ, *J* = 7.2 Hz, 1H), 3.83 – 3.77 (m, 1H), 3.48 – 3.37 (m, 1H), 1.96 – 1.83 (m, 2H), 1.59 – 1.45 (m, 2H). **^{13}C NMR** (126 MHz, $CDCl_3$) δ 131.2 (d, *J* = 7.8 Hz), 122.7 (d, *J* = 3.3 Hz), 120.1 (d, *J* = 21.2 Hz), 114.4 (d, *J* = 24.3 Hz), 101.8, 73.0, 68.4, 53.2, 28.0, 24.8. **LCMS** (basic, negative ion mode): t_R: 3.61 min, purity: 98.7% (230 nm), $(M - H)^-$ calcd. for $C_{12}H_{14}FNO_4S$: 286, found: 286. **HRMS**: corresponding ion not detected in positive ion mode.

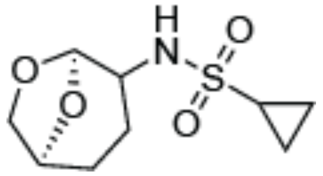

N-((1S,5R)-6,8-dioxabicyclo[3.2.1]octan-4-yl)cyclopropanesulfonamide (12f)

General procedure F was performed, using amine **4** (199 mg, 1.54 mmol, 1.20 eq), cyclopropanesulfonyl chloride (217 mg, 1.54 mmol, 1.00 eq), DCM (10.0 mL), and a reaction time of 3 d. Purification by column chromatography (MeOH/DCM, 0–10%) provided the title compound (149 mg, 50%).

Diastereomeric ratio: 1.7:1.0. Proposed major diastereomer: *Exo*.

^{1}H NMR (300 MHz, $CDCl_3$) δ 5.41 (s^Δ, 0.4H)^b, 5.40 – 5.35 (m, 0.6H)^a, 4.91 (d, *J* = 10.2 Hz, 0.6H)^a, 4.57 – 4.47 (m, 1H)^{a,b}, 4.41 (d, *J* = 9.7 Hz, 0.4H)^b, 3.95 (d^Δ, *J* = 7.2 Hz, 0.6H)^a, 3.89 – 3.77 (m, 1.4H)^{a,b}, 3.53 – 3.39 (m, 1H)^{a,b}, 2.50 – 2.36 (m, 1H)^{a,b}, 2.19 – 1.85 (m, 2H)^{a,b}, 1.82 – 1.44 (m, 2H)^{a,b}, 1.30 – 1.07 (m, 2H)^{a,b}, 1.07 – 0.92 (m, 2H)^{a,b}. **^{13}C NMR** (76 MHz, $CDCl_3$) δ 102.6^b, 101.8^a, 73.3^a, 73.0^b, 68.4^b, 67.6^a, 53.4^b, 51.4^a, 31.8^b, 31.5^a, 28.1^b, 25.4^a, 25.0^b, 22.7^a, 5.9^a, 5.6^b. **LCMS**: No UV absorption at 230/254 nm. **HRMS**: $(M + H)^+$ calcd. for $C_9H_{15}NO_4S$: 234.0795, found: 234.0799.

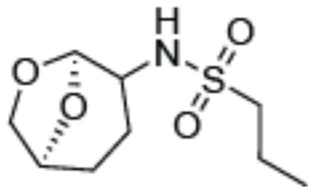

N-((1S,5R)-6,8-dioxabicyclo[3.2.1]octan-4-yl)propane-1-sulfonamide (12g)

General procedure F was performed, using amine **4** (232 mg, 1.80 mmol, 1.00 eq), propane-1-sulfonyl chloride (427 mg, 2.99 mmol, 1.67 eq), DCM (7.5 mL), Et_3N (0.42 mL, 3.0 mmol, 1.7 eq), and a reaction time of overnight. Purification by column chromatography (EtOAc/cHex, 0–100%) provided the title compound (31 mg, 7%).

Diastereomeric ratio: 1.8:1.0. Proposed major diastereomer: *Exo*.

^{1}H NMR (300 MHz, $CDCl_3$) δ 5.29 (s^{Δ}, 0.4H)^{b}, 5.27 (s^{Δ}, 0.6H)^{b}, 4.71 (d, *J* = 10.1 Hz, 0.6H)^{a}, 4.51 – 4.37 (m, 1H)^{a,b}, 4.20 (d, *J* = 9.8 Hz, 0.4H)^{b}, 3.88 (d^{Δ} , *J* = 7.2 Hz, 0.6H)^{a}, 3.84 – 3.69 (m, 1.4H)^{a,b}, 3.44 – 3.28 (m, 1H)^{a,b}, 3.00 – 2.85 (m, 2H)^{a,b}, 1.90 – 1.34 (m, 4H)^{a,b}, 1.08 – 0.94 (m, 3H)^{a,b}. **^{13}C NMR** (76 MHz, $CDCl_3$) δ 102.6^{b}, 101.8^{a}, 73.3^{a}, 73.0^{b}, 68.4^{b}, 67.6^{a}, 56.3^{b}, 56.0^{a}, 53.3^{b}, 51.4^{a}, 28.1^{b}, 25.4^{a}, 25.1^{b}, 22.8^{a}, 17.7^{a,b}, 13.1^{a,b}. **LCMS**: No UV absorption at 230/254 nm. **HRMS**: $(M + H)^{+}$ calcd. for $C_9H_{17}NO_4S$: 236.0951, found: 236.0954.

Section S3: Selected analytical data

^{1}H-NMR spectrum of **2**

^{1}H NMR (600 MHz, $CDCl_3$) δ 5.29 (s$^{\Delta}$, 1H), 4.52 – 4.43 (m, 1H), 3.83 (d$^{\Delta}$, J = 7.1 Hz, 1H), 3.81 – 3.75 (m, 1H), 3.61 – 3.52 (m, 1H), 2.21 (s, 1H), 2.04 – 1.97 (m, 1H), 1.91 – 1.82 (m, 1H), 1.61 – 1.53 (m, 1H), 1.53 – 1.45 (m, 1H).

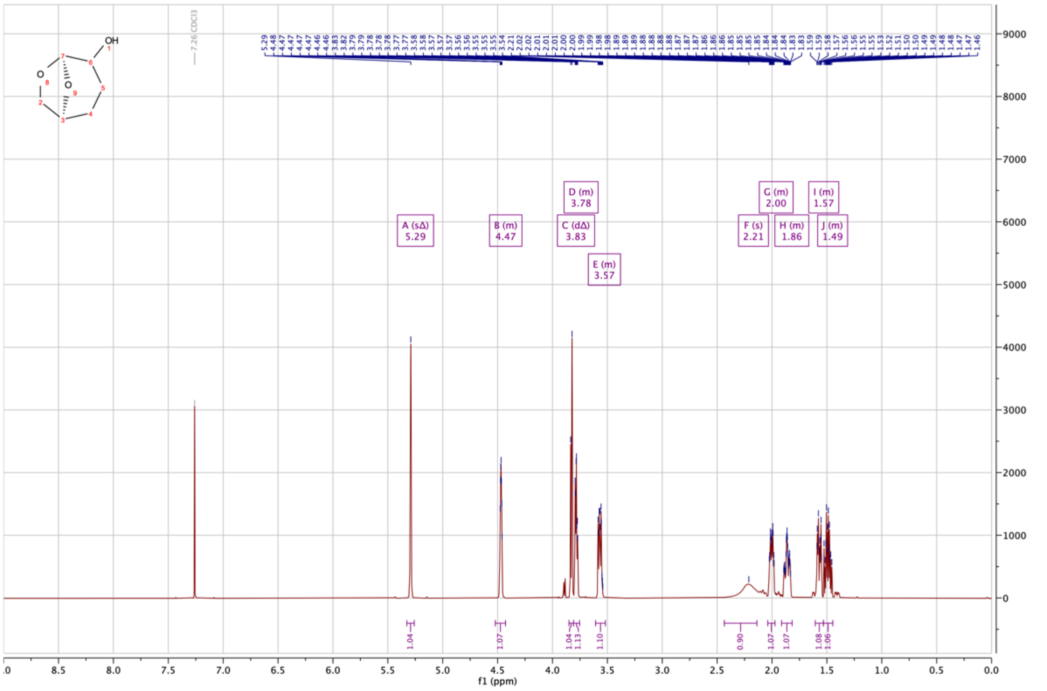

^{13}C-NMR spectrum of **2**

^{13}C NMR (151 MHz, $CDCl_3$) δ 103.0, 73.4, 72.9, 69.1, 68.3, 27.9, 26.1.

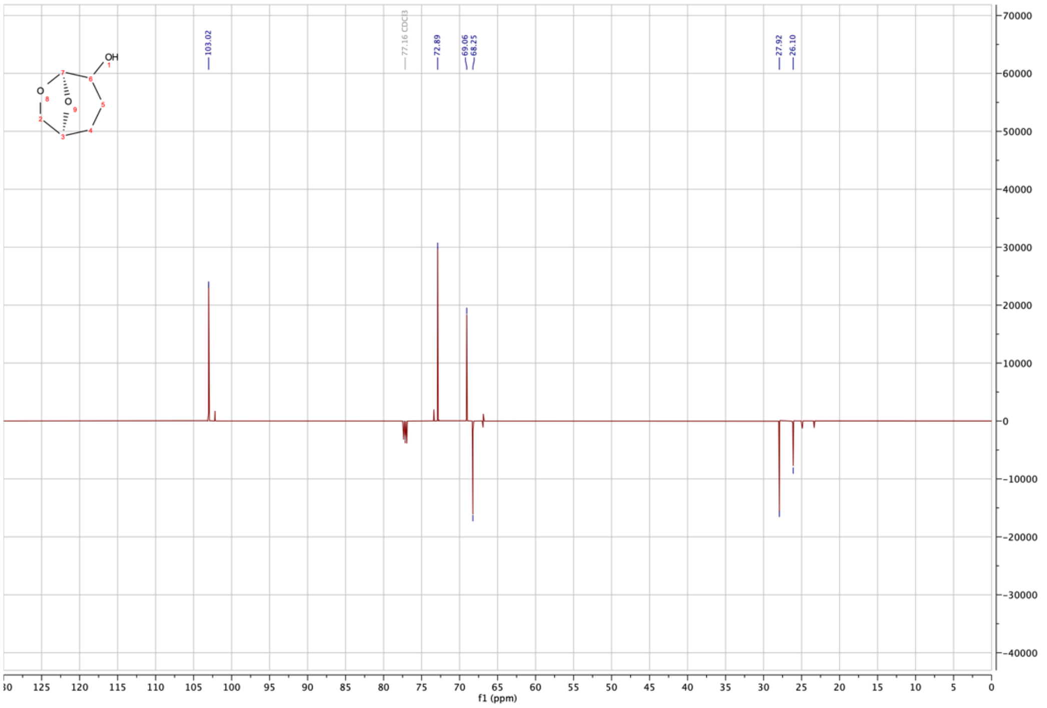

^{1}H-NMR spectrum of **3**

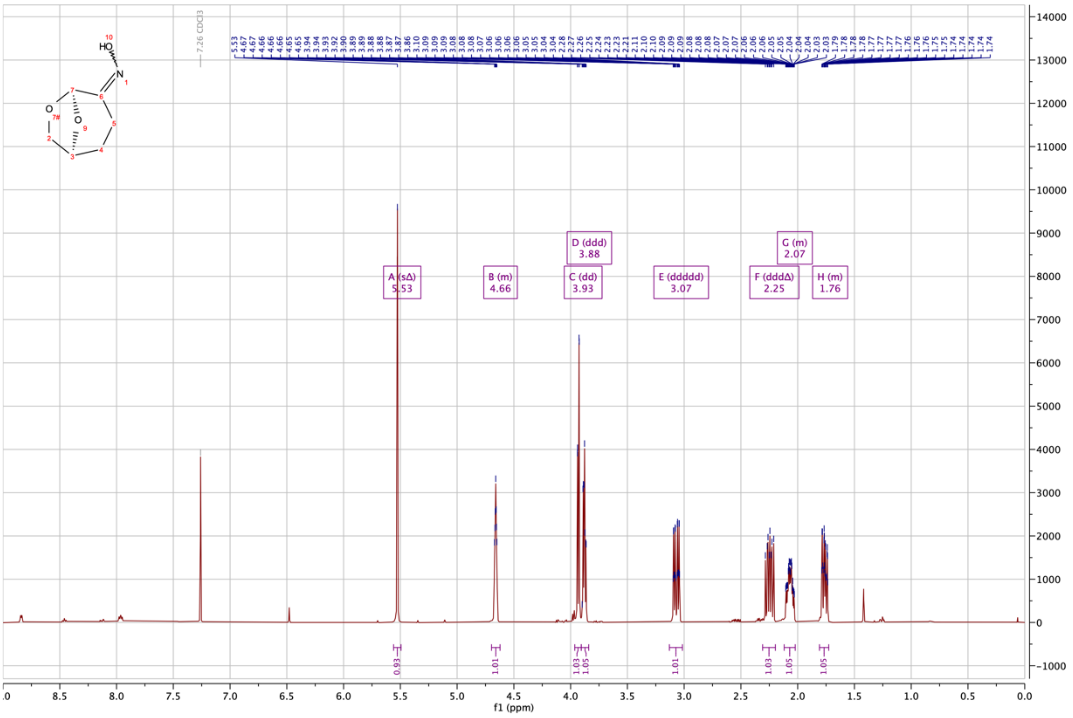

^{1}H-NMR spectrum of **4**

^{1}H NMR (500 MHz, $CDCl_3$) δ 5.26 ($s^Δ$, 0.4H)^b, 5.20 ($s^Δ$, 0.6H)^a, 4.54 – 4.44 (m, 1H)^{a,b}, 3.91 ($d^Δ$, J = 7.1 Hz, 0.4H)^b, 3.81 ($d^Δ$, J = 7.0 Hz, 0.6H)^a, 3.79 – 3.75 (m, 1H)^{a,b}, 2.83 – 2.79 (m, 0.4H)^b, 2.76 ($dd^Δ$, J = 11.1, 5.0 Hz, 0.6H)^a, 2.11 – 2.01 (m, 1H)^{a,b}, 1.93 – 1.82 (m, 1H)^{a,b}, 1.54 (m, 0.6H)^a, 1.47 – 1.42 (m, 0.4H)^b, 1.42 – 1.31 (m, 1H)^{a,b}.

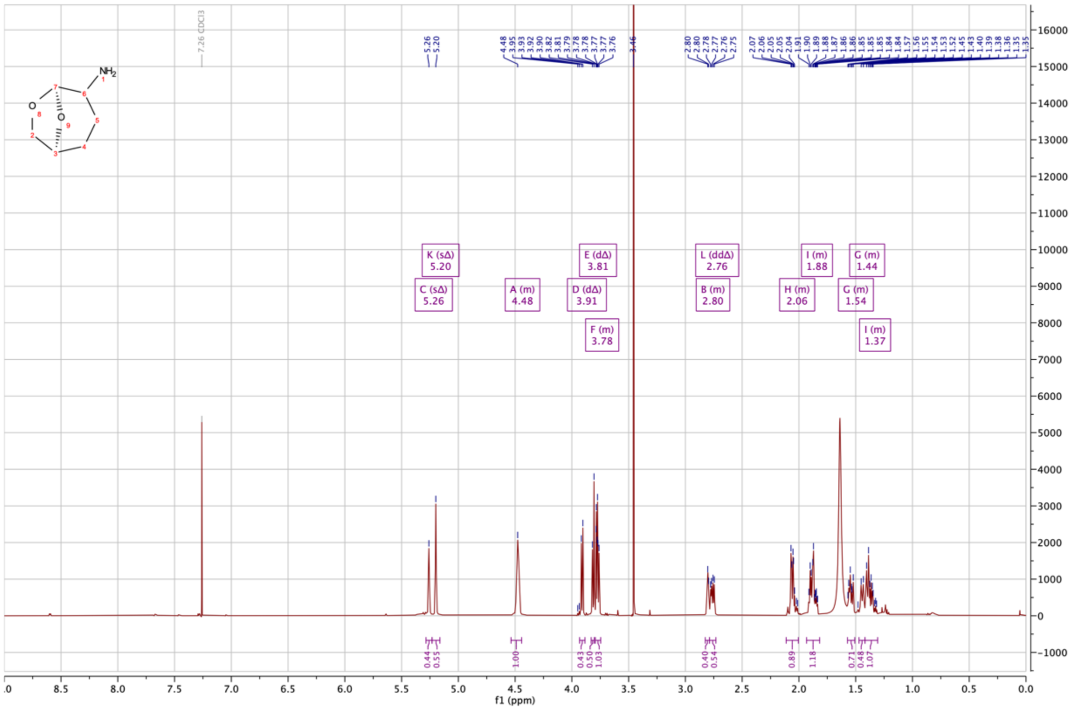

^{13}C-NMR spectrum of **4**

^{13}C NMR (126 MHz, $CDCl_3$) δ 105.0^a, 104.1^b, 73.6^b, 72.8^a, 68.2^a, 67.0^b, 51.4^a, 49.5^b, 28.2^a, 26.4^b, 24.9^a, 24.2^b.

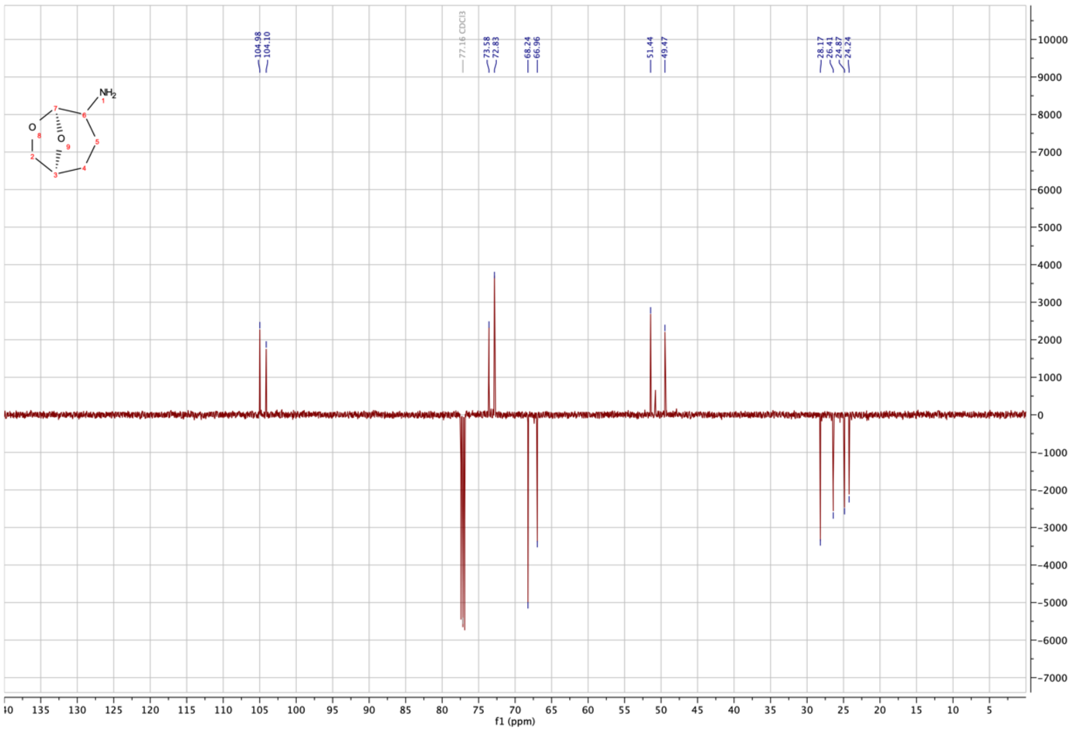

^{1}H-NMR spectrum of **5a**

^{1}H NMR (500 MHz, $CDCl_3$) δ 7.41 – 7.24 (m, 5H)^{a,b}, 5.42 (s$^{\Delta}$, 0.9H)^{a}, 5.41 – 5.36 (m, 0.1H)^{b}, 4.64 (d, J = 12.3 Hz, 0.1H)^{b}, 4.59 (ABq, 1.8H, $\Delta\delta_{AB}$ = 0.02, J_{AB} = 12.3 Hz)^{a}, 4.54 (d, J = 12.3 Hz, 0.1H)^{b}, 4.52 (s, 0.1H)^{b}, 4.50 – 4.44 (m, 0.9H)^{a}, 3.92 – 3.85 (m, 1H)^{a,b}, 3.81 (ddd$^{\Delta}$, J = 6.9, 5.0, 1.5 Hz, 0.9H)^{a}, 3.77 (ddd$^{\Delta}$, J = 6.9, 5.1, 1.6 Hz, 0.1H)^{b}, 3.44 (ddd$^{\Delta}$, J = 10.3, 5.7, 1.6 Hz, 0.9H)^{a}, 3.32 (ddd$^{\Delta}$, J = 4.2, 1.9, 1.9 Hz, 0.1H)^{b}, 2.23 – 2.12 (m, 0.1H)^{b}, 2.01 – 1.92 (m, 0.9H)^{a}, 1.90 – 1.79 (m, 1H)^{a,b}, 1.79 – 1.67 (m, 1H)^{a,b}, 1.64 – 1.56 (m, 0.9H)^{a}, 1.44 – 1.36 (m, 0.1H)^{b}.

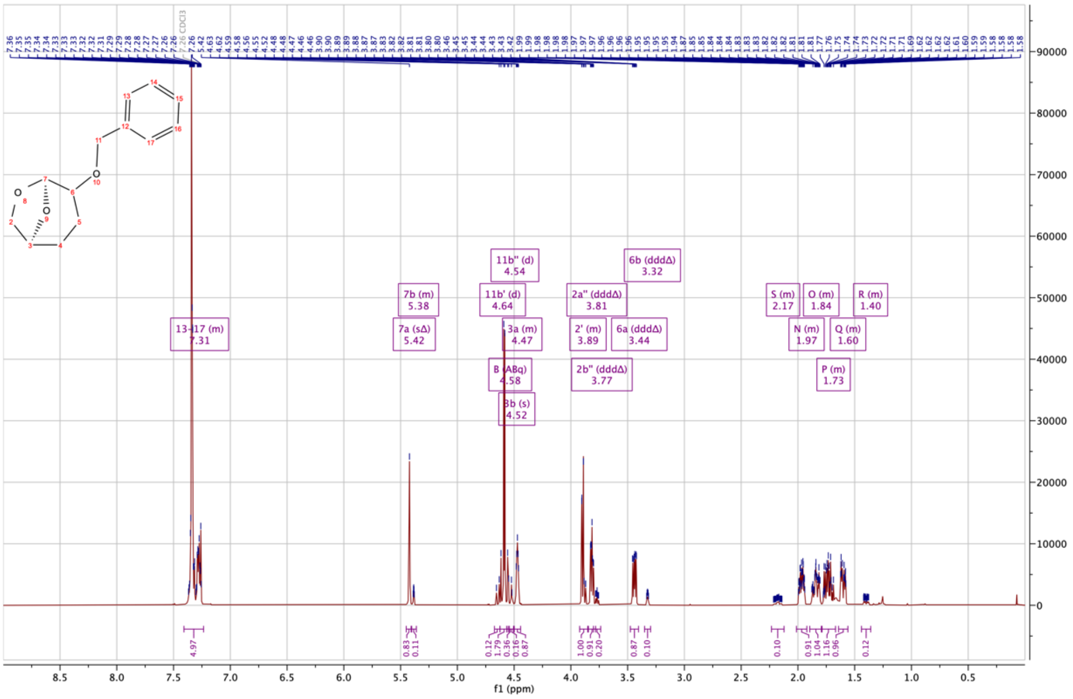

^{13}C-NMR spectrum of **5a**

^{13}C NMR (126 MHz, $CDCl_3$) δ 138.5^{b}, 138.4^{a}, 128.5^{a,b}, 127.8^{a,b}, 127.8^{a,b}, 101.1^{a}, 100.9^{b}, 75.8^{a}, 73.2^{a}, 73.2^{b}, 73.0^{b}, 71.4^{b}, 70.8^{a}, 68.5^{a}, 66.8^{b}, 28.0^{a}, 25.3^{b}, 23.0^{a}, 20.3^{b}.

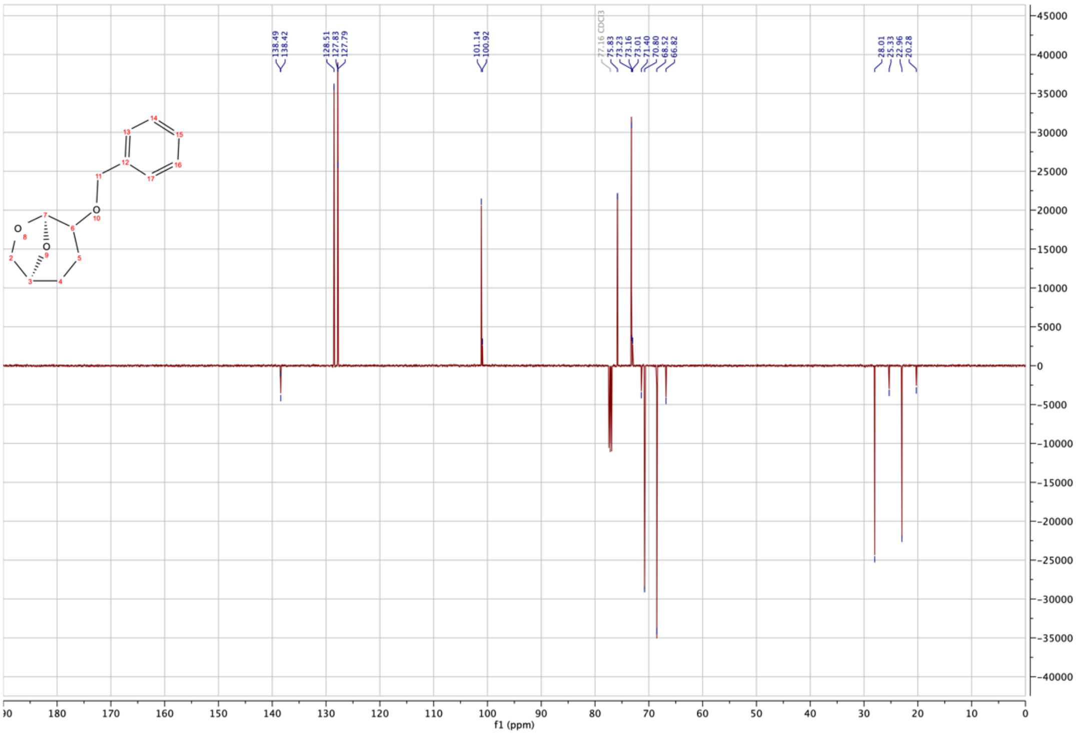

^{1}H-NMR spectrum of **6a**

^{1}H NMR (500 MHz, $CDCl_3$) δ 8.06 (d^{Δ}, J = 7.3 Hz, 2H), 7.56 (t^{Δ}, J = 7.4, 7.4 Hz, 1H), 7.43 (dd^{Δ}, J = 7.7, 7.7 Hz, 2H), 5.52 (s^{Δ}, 1H), 4.99 (dd^{Δ}, J = 10.3, 5.9 Hz, 1H), 4.62 – 4.54 (m, 1H), 3.98 (d^{Δ}, J = 7.1 Hz, 1H), 3.88 (dd^{Δ}, J = 6.0, 6.0 Hz, 1H), 2.18 – 2.09 (m, 1H), 2.09 – 1.98 (m, 1H), 1.98 – 1.87 (m, 1H), 1.70 (dd^{Δ}, J = 13.7, 5.7 Hz, 1H).

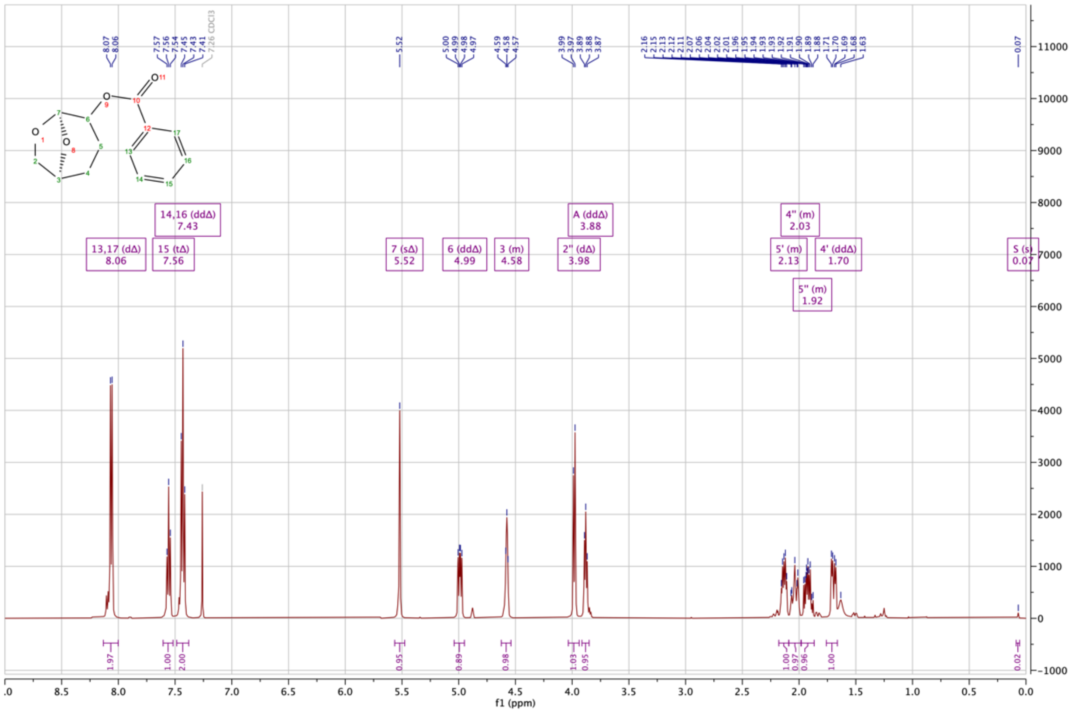

^{13}C-NMR spectrum of **6a**

^{13}C NMR (126 MHz, $CDCl_3$) δ 166.0, 133.3, 129.9, 128.5, 128.5, 100.7, 73.3, 72.0, 68.7, 28.0, 22.1.

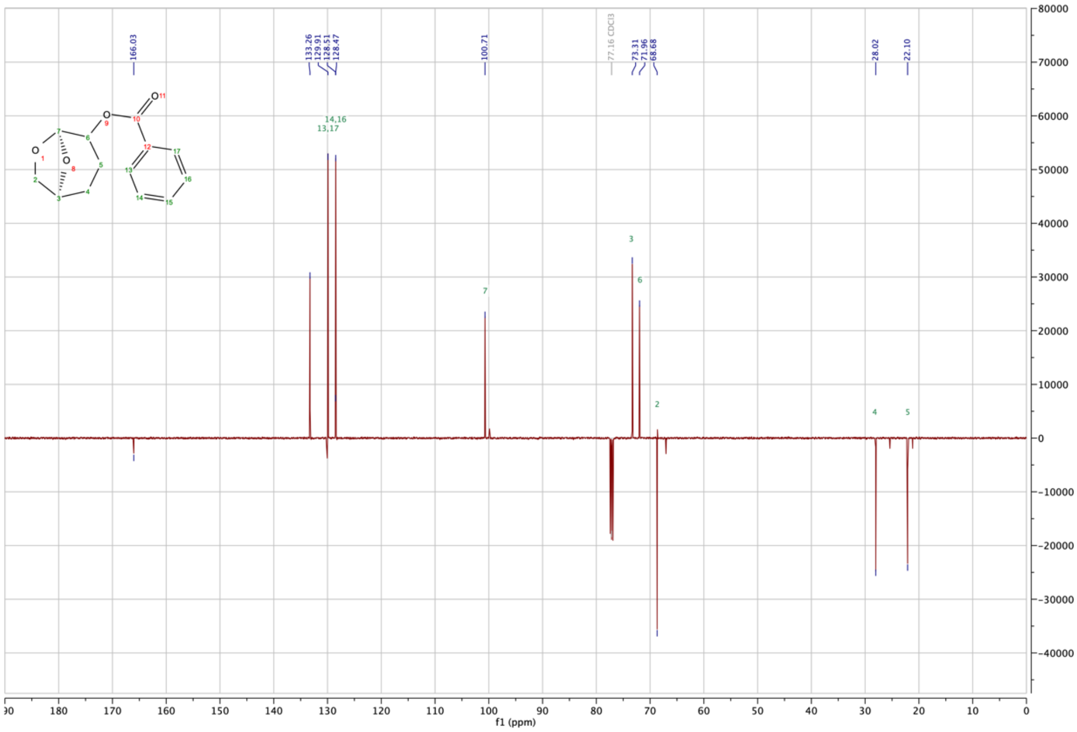

^{1}H-NMR spectrum of **7a**

^{1}H NMR (500 MHz, $CDCl_3$) δ 7.52 (d$^{\Delta}$, J = 6.2 Hz, 1H), 7.09 (dd, J = 7.8, 7.8 Hz, 1H), 6.96 (d$^{\Delta}$, J = 7.5 Hz, 1H), 6.50 (s, 1H), 5.50 (s$^{\Delta}$, 1H), 4.77 (ddd, J = 10.5, 6.0, 1.6 Hz, 1H), 4.59 – 4.51 (m, 1H), 3.95 (d$^{\Delta}$, J = 7.1 Hz, 1H), 3.86 (ddd, J = 6.9, 5.1, 1.5 Hz, 1H), 2.29 (s, 3H), 2.14 (s, 3H), 2.18 – 2.06 (m, 1H), 2.04 – 1.94 (m, 1H), 1.87 – 1.75 (m, 1H), 1.70 – 1.63 (m, 1H).

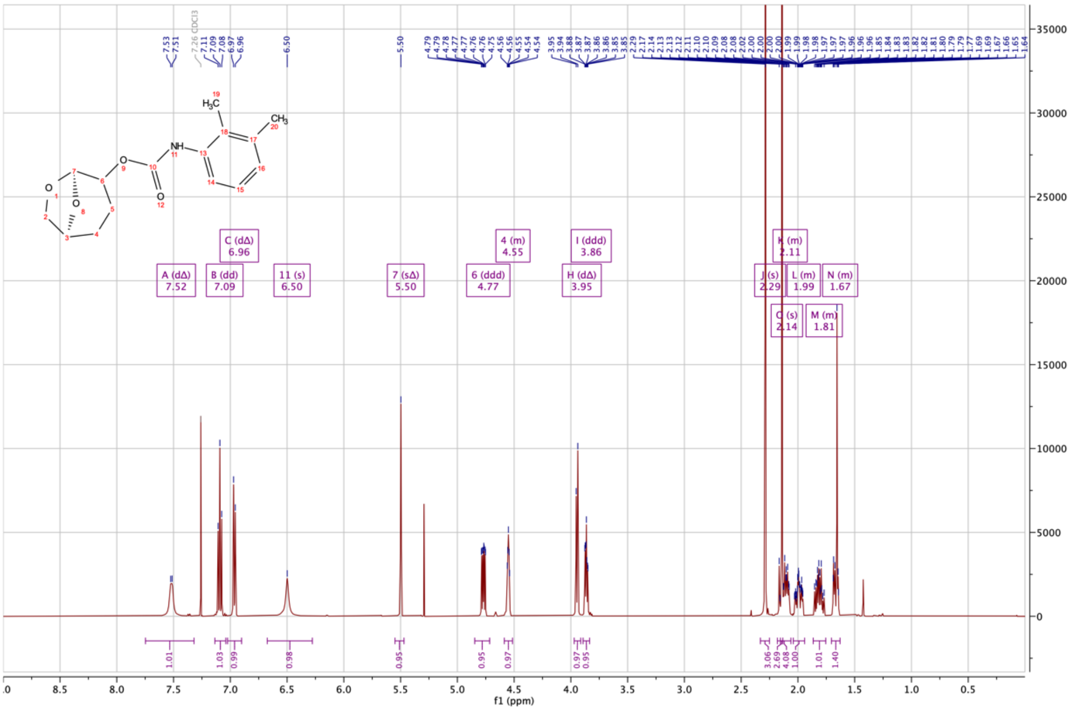

^{13}C-NMR spectrum of **7a**

^{13}C NMR (126 MHz, $CDCl_3$) δ 153.3, 137.4, 135.4, 126.7, 126.1, 120.3, 100.9, 73.2, 68.6, 27.9, 22.3, 20.8, 13.6; (2 missing signals).

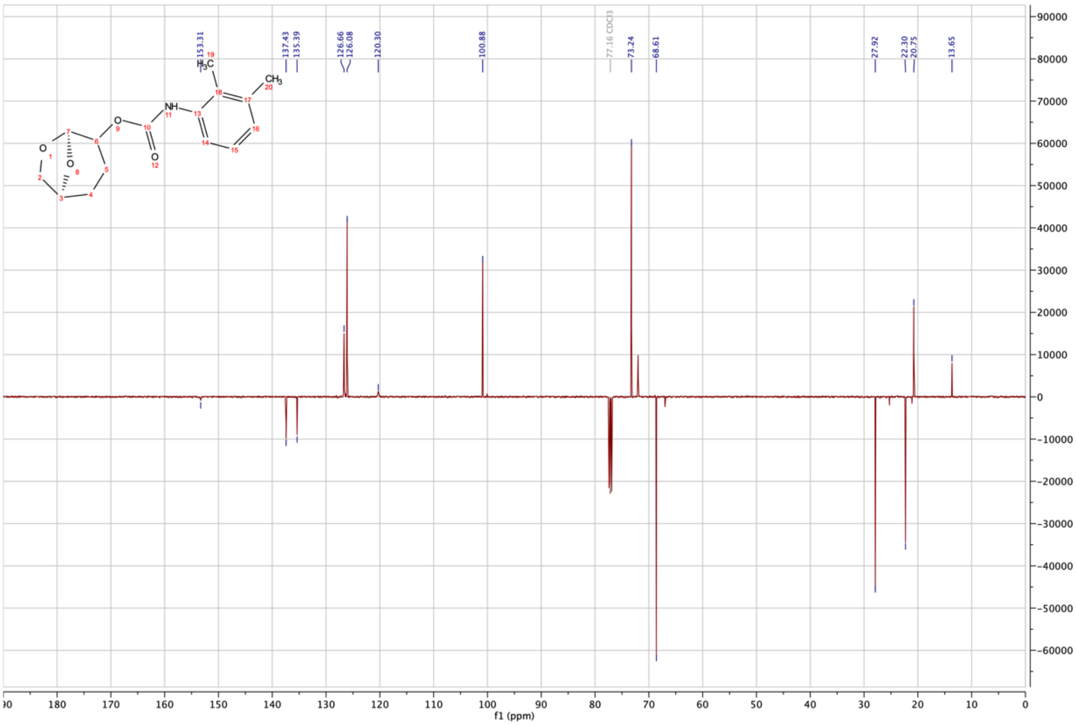

^{1}H-NMR spectrum of **8h**

^{1}H NMR (500 MHz, $CDCl_3$) δ 7.29 (d, J = 8.4 Hz, 1H), 7.29 (d, J = 7.8 Hz, 1H), 7.13 (dd, J = 8.4, 7.7 Hz, 1H), 5.40 – 5.31 (m, 1H), 4.50 – 4.42 (m, 1H), 4.09 (ABq, 2H, $\Delta\delta_{AB}$ = 0.08, J_{AB} = 12.5 Hz), 3.91 (d$^\Delta$, J = 7.1 Hz, 1H), 3.76 (ddd, J = 6.8, 5.0, 1.5 Hz, 1H), 2.76 – 2.70 (m, 1H), 2.05 (ddddd, J = 13.6, 13.6, 5.1, 3.2, 1.5 Hz, 1H), 1.89 (dddd$^\Delta$, J = 13.6, 13.6, 5.4, 5.4 Hz, 1H), 1.68 (m, 1H), 1.39 (dd$^\Delta$, J = 13.7, 5.7 Hz, 1H).

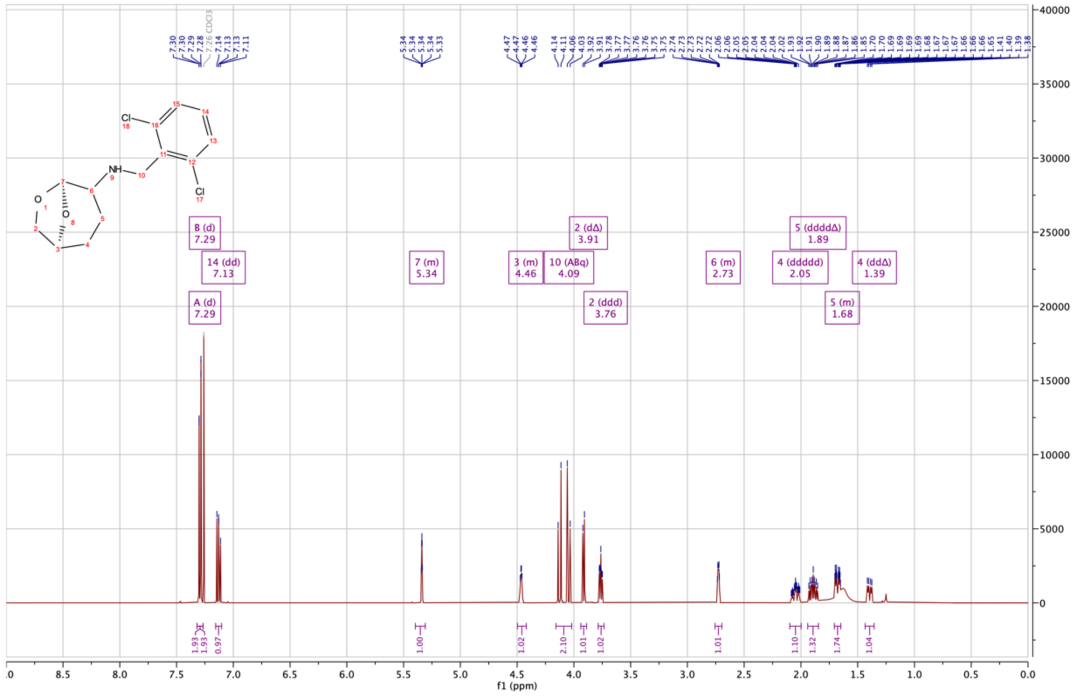

^{13}C-NMR spectrum of **8h**

^{13}C NMR (126 MHz, $CDCl_3$) δ 136.3, 136.0, 129.1, 128.6, 102.9, 73.4, 67.1, 55.6, 46.9, 25.4, 20.2.

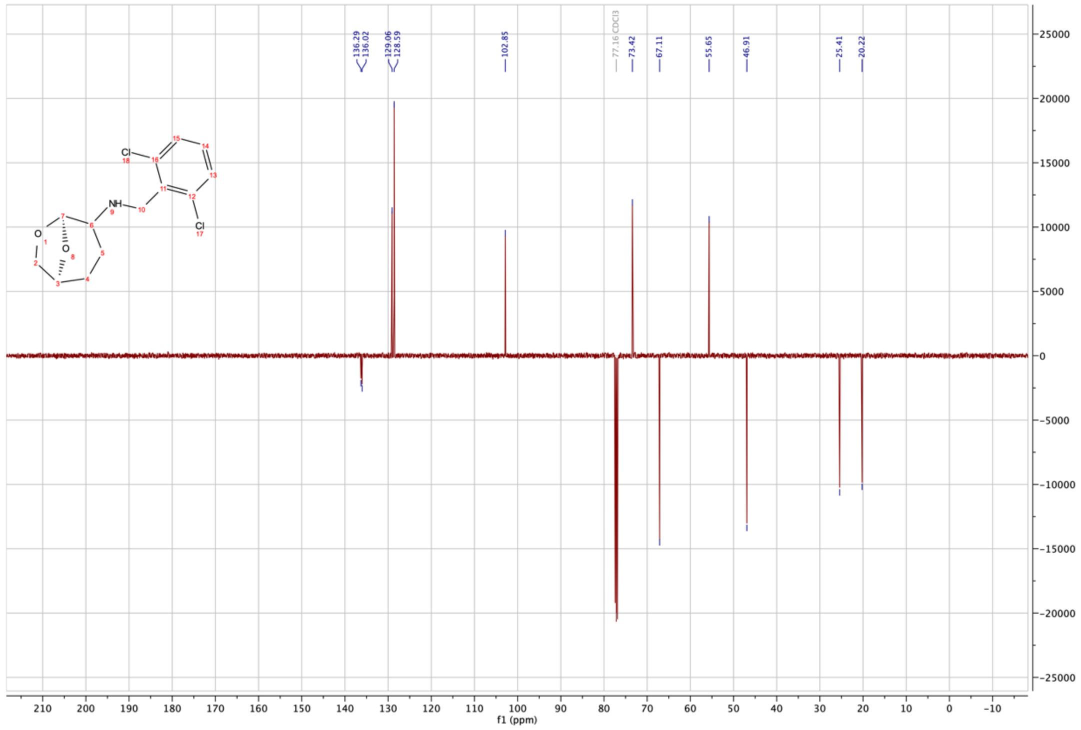

^{1}H-NMR spectrum of **8i**

^{1}H NMR (500 MHz, $CDCl_3$) δ 7.28 (d, J = 8.4 Hz, 1H), 7.28 (d, J = 7.7 Hz, 1H), 7.12 (dd, J = 8.4, 7.7 Hz, 1H), 5.42 (s$^{\Delta}$, 1H), 4.53 – 4.45 (m, 1H), 4.10 (ABq, 2H, $\Delta\delta_{AB}$ = 0.01, J_{AB} = 12.8 Hz), 3.84 (d$^{\Delta}$, J = 7.0 Hz, 1H), 3.78 (ddd, J = 6.9, 5.0, 1.4 Hz, 1H), 2.75 (ddd, J = 11.0, 5.3, 1.4 Hz, 1H), 1.92 – 1.83 (m, 2H), 1.58 – 1.51 (m, 1H), 1.49 – 1.39 (m, 1H).

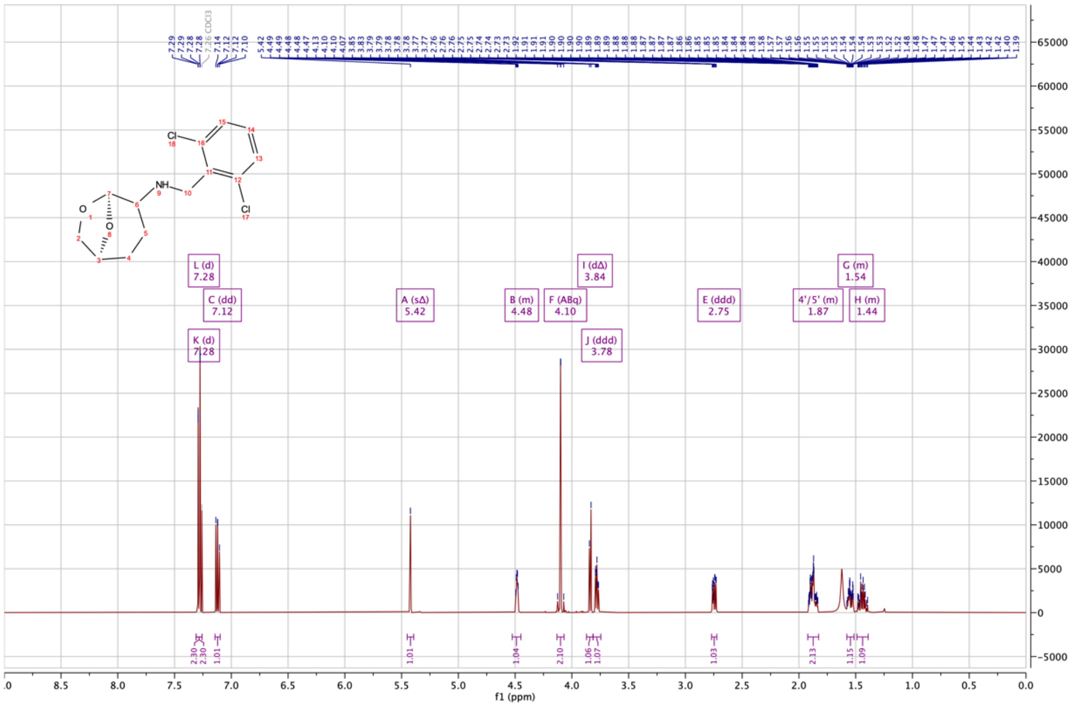

^{13}C-NMR spectrum of **8i**

^{13}C NMR (126 MHz, $CDCl_3$) δ 136.3, 136.0, 129.0, 128.6, 102.3, 73.2, 68.4, 57.4, 46.2, 28.3, 24.5.

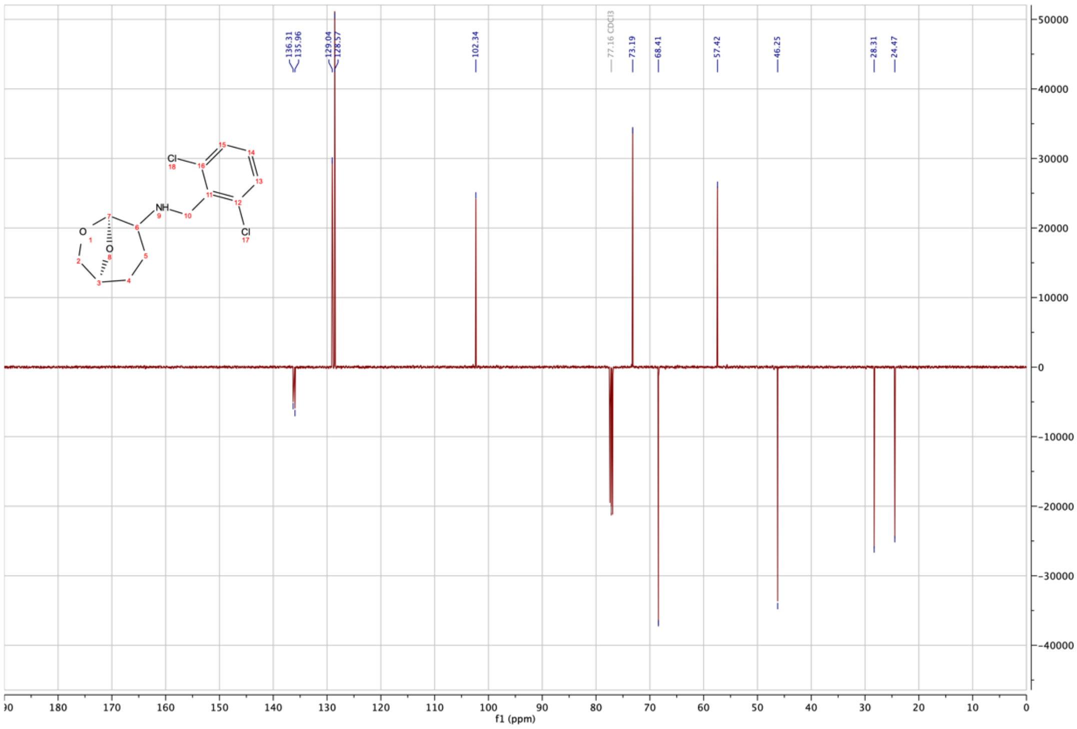

^{1}H-NMR spectrum of **9f**

^{1}H NMR (500 MHz, $CDCl_3$) δ 7.38 (dd, J = 2.4, 1.6 Hz, 1H), 7.35 (dd, J = 7.8, 7.8 Hz, 1H), 7.31 (ddd, J = 7.6, 1.3, 1.3 Hz, 1H), 7.05 (ddd, J = 8.0, 2.6, 1.2 Hz, 1H), 6.58 (d, J = 8.6 Hz, 1H), 5.41 – 5.36 (m, 1H), 4.59 – 4.52 (m, 1H), 4.25 – 4.17 (m, 1H), 4.01 (d^{Δ}, J = 7.2 Hz, 1H), 3.86 (s, 3H), 3.88 – 3.82 (m, 1H), 2.14 (dddd^{Δ}, J = 13.9, 13.9, 5.4, 5.4 Hz, 1H), 2.07 – 1.95 (m, 1H), 1.75 – 1.67 (m, 1H), 1.53 (dd^{Δ}, J = 14.1, 5.3 Hz, 1H).

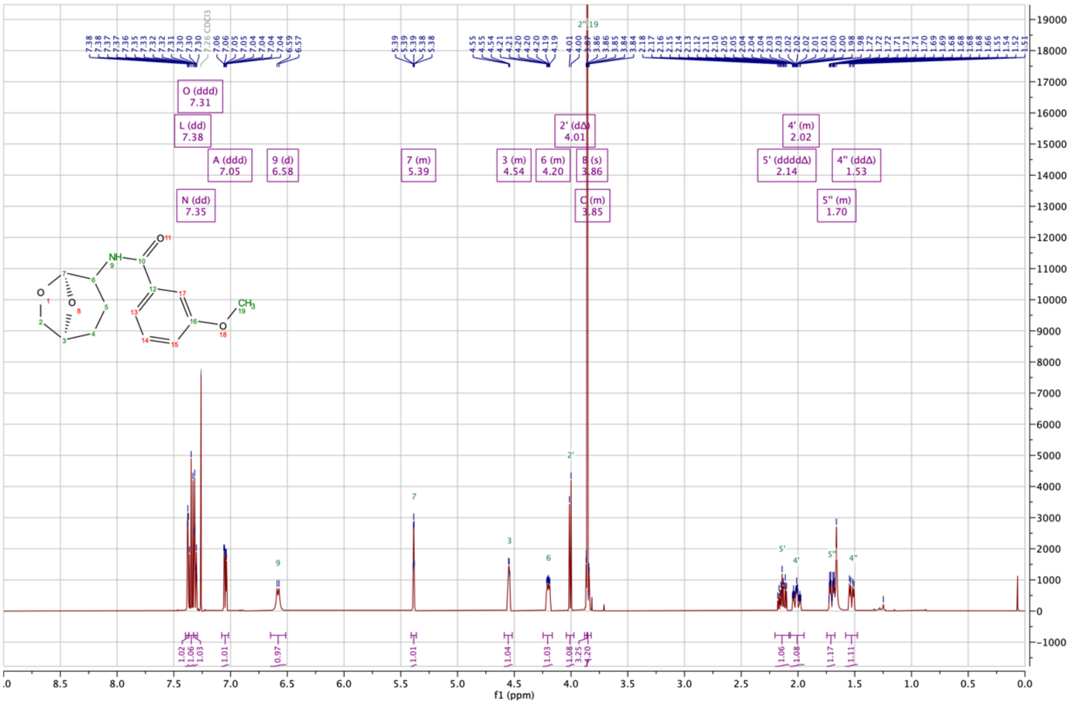

^{13}C-NMR spectrum of **9f**

^{13}C NMR (126 MHz, $CDCl_3$) δ 166.7, 160.0, 136.0, 129.7, 118.8, 117.9, 112.6, 101.6, 73.5, 67.6, 55.6, 47.7, 25.6, 21.5.

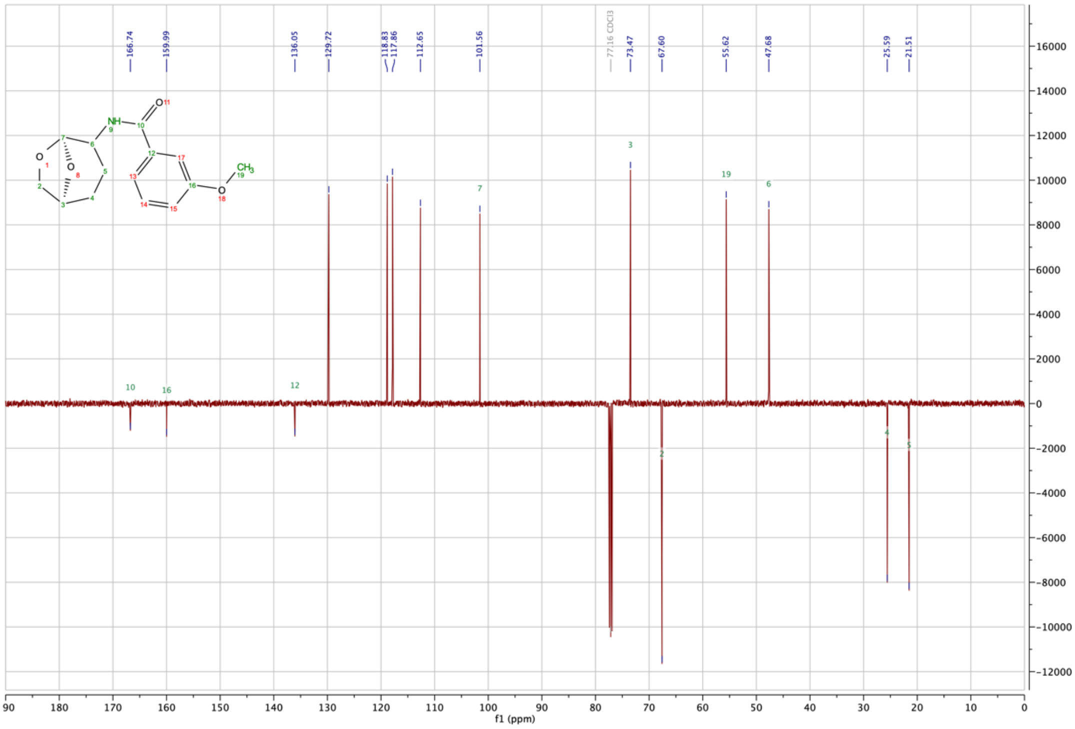

^{1}H-NMR spectrum of **9g**

^{1}H NMR (500 MHz, $CDCl_3$) δ 7.35 (dd, J = 2.4, 1.6 Hz, 1H), 7.32 (dd, J = 7.8, 7.8 Hz, 1H), 7.27 (ddd, J = 7.7, 1.4, 1.4 Hz, 1H), 7.03 (ddd, J = 8.1, 2.6, 1.1 Hz, 1H), 6.09 (d, J = 8.8 Hz, 1H), 5.40 (s^{Δ}, 1H), 4.60 – 4.52 (m, 1H), 4.31 – 4.23 (m, 1H), 3.90 (d^{Δ}, J = 7.1 Hz, 1H), 3.84 (s, 3H), 3.86 – 3.81 (m, 1H), 2.10 – 1.97 (m, 2H), 1.67 – 1.54 (m, 2H).

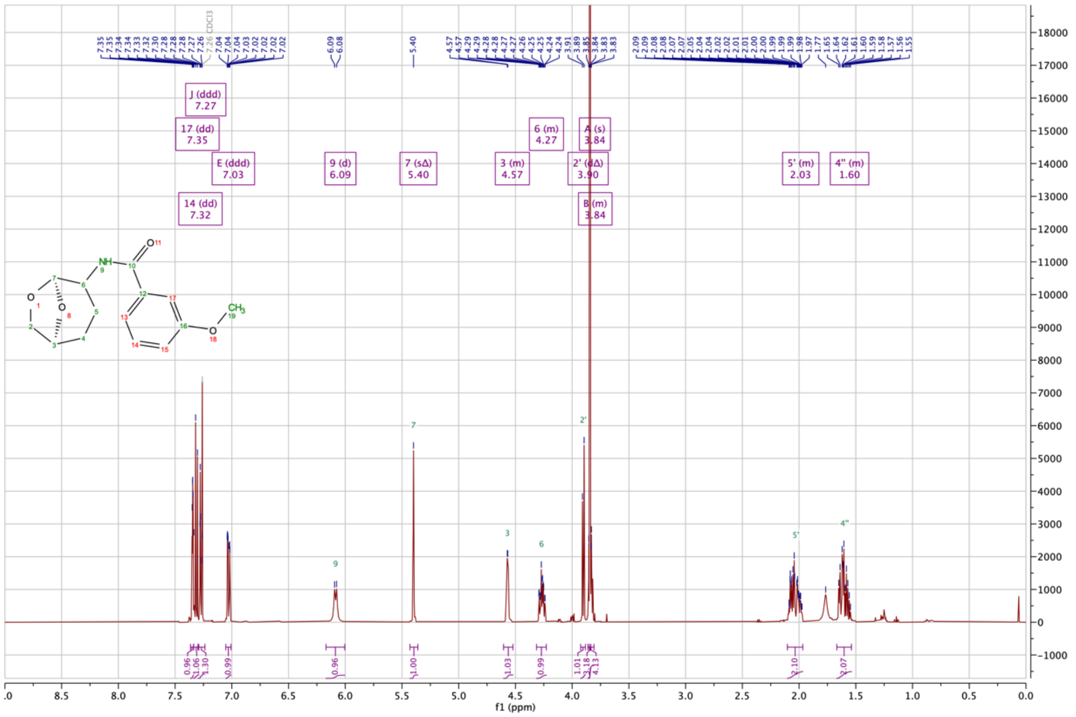

^{13}C-NMR spectrum of **9g**

^{13}C NMR (126 MHz, $CDCl_3$) δ 166.9, 160.0, 135.9, 129.7, 118.8, 118.0, 112.5, 102.1, 73.2, 68.3, 55.6, 49.5, 27.9, 23.6.

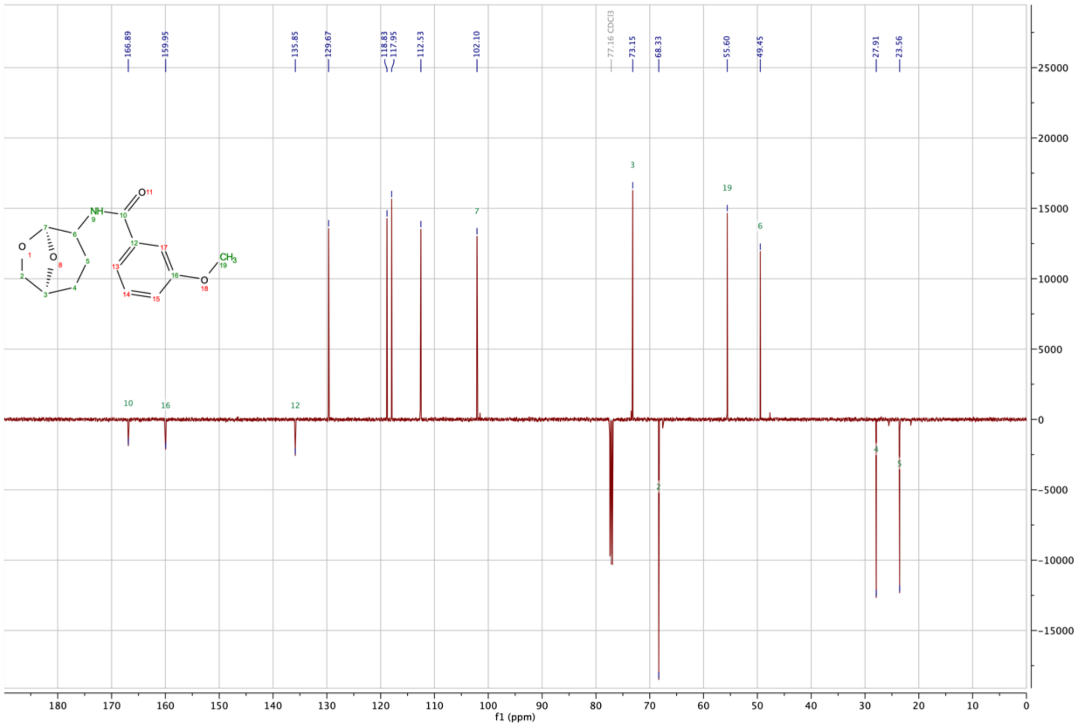

^{1}H-NMR spectrum of **10a**

^{1}H NMR (500 MHz, $CDCl_3$) δ 7.39 – 7.27 (m, 1H)[a,b], 7.15 – 6.95 (m, 3H)[a,b], 5.41 – 5.24 (m, 1.5H)[a,b], 5.17 – 5.03 (m, 2H)[a,b], 4.81 (d, *J* = 8.6 Hz, 0.5H)[a], 4.54 – 4.50 (m, 0.5H)[a], 4.50 – 4.47 (m, 0.5H)[b], 3.95 (d[Δ], *J* = 7.2 Hz, 0.5H)[b], 3.84 (d[Δ], *J* = 6.9 Hz, 0.5H)[a], 3.83 – 3.70 (m, 2H)[a,b], 2.12 – 1.85 (m, 2H)[a,b], 1.61 – 1.38 (m, 2H)[a,b].

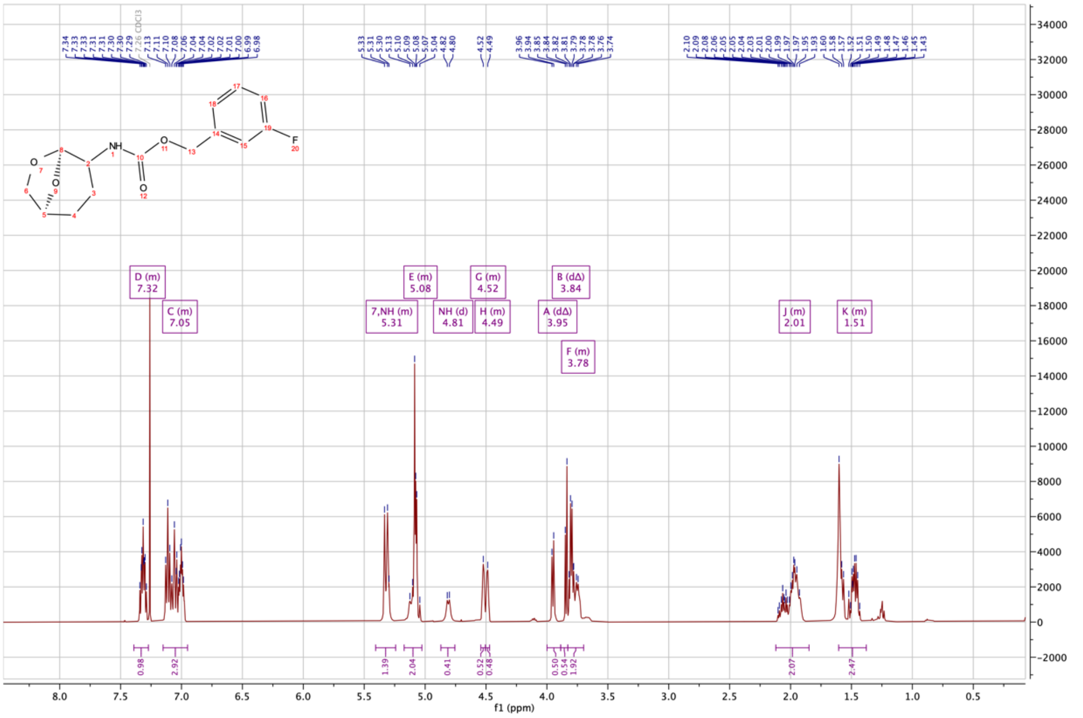

^{13}C-NMR spectrum of **10a**

^{13}C NMR (126 MHz, $CDCl_3$) δ 163.0 (d, *J* = 246.3 Hz)[a/b], 163.0 (d, *J* = 246.2 Hz)[a/b], 155.5, 155.5, 139.2 – 139.0 (m)[a,b], 130.4 – 130.1 (m)[a,b], 123.7 – 123.4 (m)[a,b], 115.6 – 114.7 (m)[a,b], 102.2[a], 101.5[b], 73.4[b], 73.1[a], 68.3[a], 67.5[b], 66.0[a,b], 50.9[a], 49.0[b], 27.9[a], 25.4[b], 23.7[a], 21.7[b].

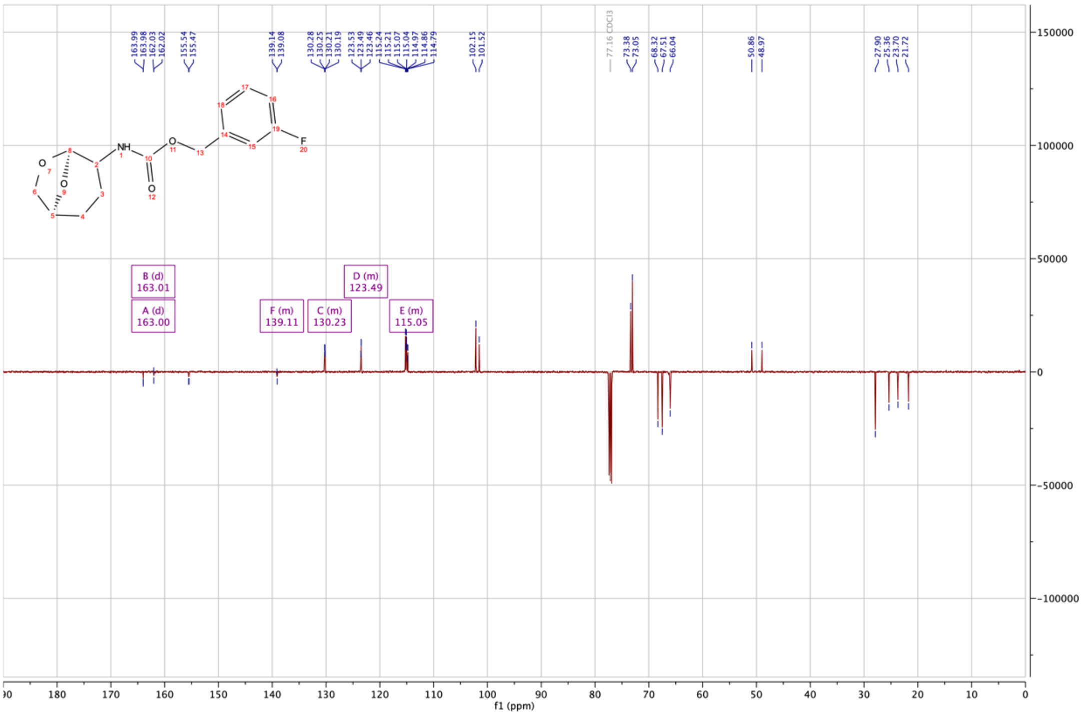

^{1}H-NMR spectrum of **11b**

^{1}H NMR (500 MHz, DMSO-d6) δ 8.55 (s, 0.5H)[b], 8.43 (s, 0.5H)[a], 7.40 – 7.31 (m, 2H)[a,b] 7.25 – 7.18 (m, 2H)[a,b], 6.95 – 6.84 (m, 1H)[a,b], 6.45 (d, J = 8.9 Hz, 0.5H)[b], 6.06 (d, J = 8.6 Hz, 0.5H)[a], 5.26 – 5.21 (m, 0.5H)[b], 5.21 (sΔ, 0.5H)[a], 4.58 – 4.47 (m, 1H)[a], 3.95 (d[Δ], J = 7.2 Hz, 0.5H)[b], 3.87 (d[Δ], J = 7.1 Hz, 0.5H)[a], 3.68 – 3.52 (m, 2H)[a,b], 2.03 – 1.90 (m, 0.5H)[b], 1.90 – 1.72 (m, 1.5H)[a,b], 1.64 – 1.55 (m, 0.5H)[b], 1.52 – 1.41 (m, 1H)[a], 1.39 (dd[Δ], J = 14.0, 5.2 Hz, 0.5H)[b].

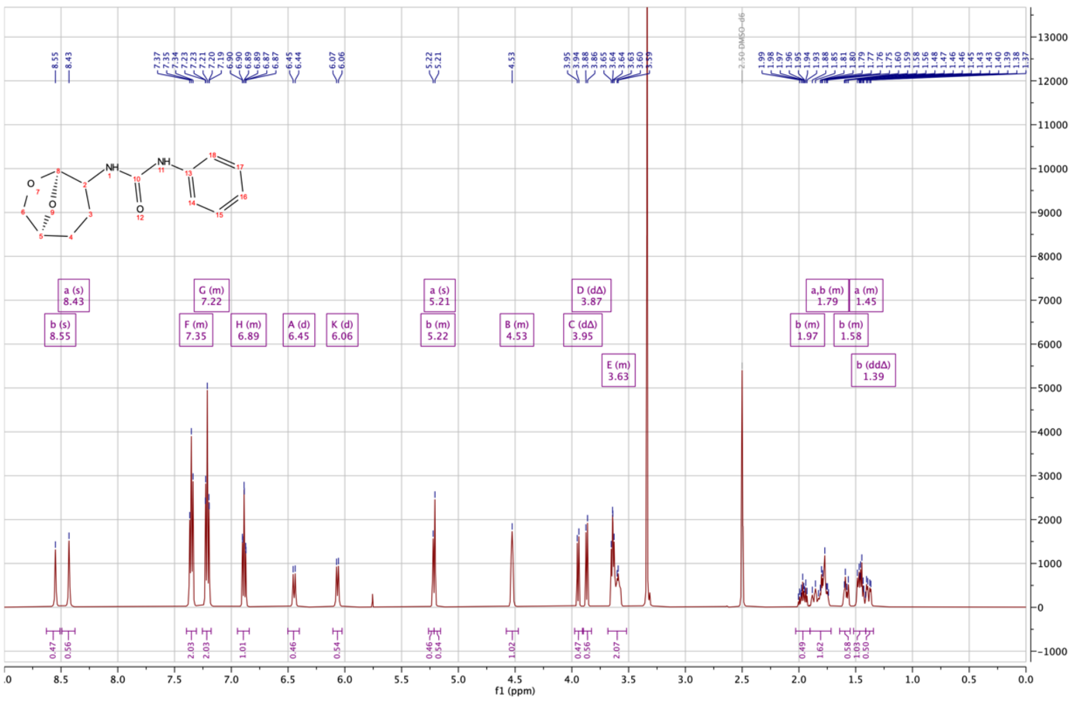

^{13}C-NMR spectrum of **11b**

^{13}C NMR (126 MHz, DMSO) δ 154.4[a/b], 154.4[a/b], 140.3[b], 140.2[a], 128.7[a,b], 121.2[a], 121.1[b], 117.5[a], 117.4[b], 101.6[a], 101.0[b], 72.5[b], 72.3[a], 67.5[a], 66.5[b], 49.1[a], 46.8[b], 27.5[a], 24.8[b], 23.1[a], 21.5[b].

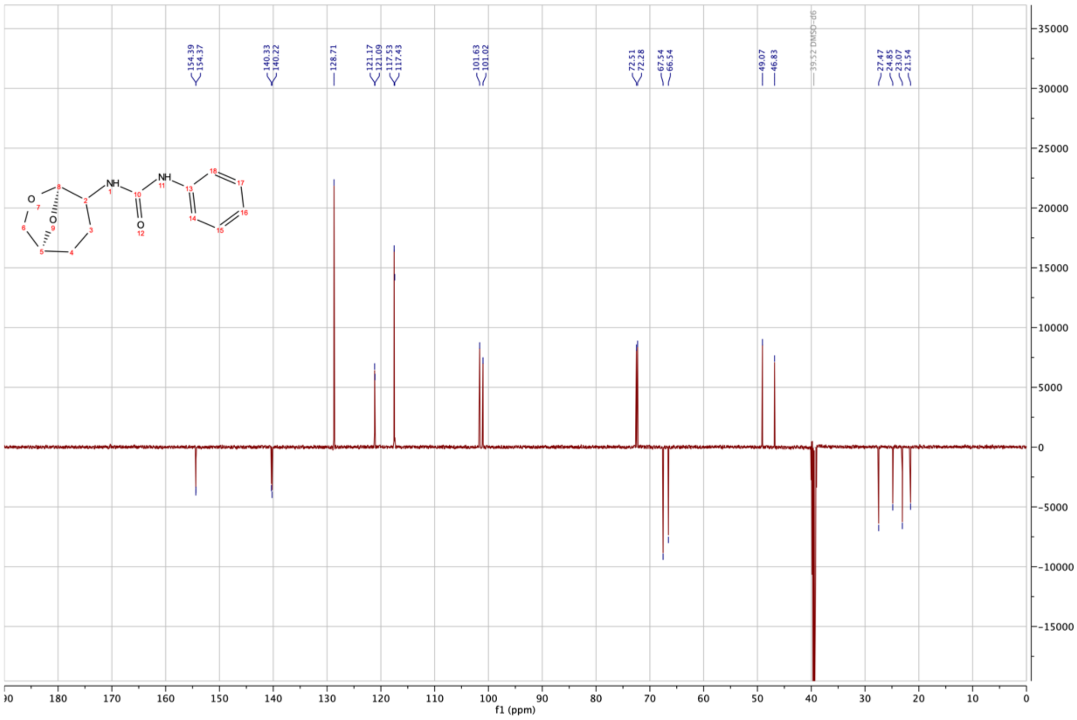

^{1}H-NMR spectrum of **12c**

^{1}H NMR (600 MHz, CDCl$_3$) δ 7.78 – 7.73 (m, 2H), 7.32 – 7.27 (m, 2H), 5.09 (d, J = 8.5 Hz, 1H), 4.97 (s$^{\Delta}$, 1H), 4.50 – 4.40 (m, 1H), 3.87 (d$^{\Delta}$, J = 7.3 Hz, 1H), 3.78 – 3.70 (m, 1H), 3.34 – 3.25 (m, 1H), 2.42 (s, 3H), 2.02 – 1.87 (m, 2H), 1.57 – 1.37 (m, 2H).

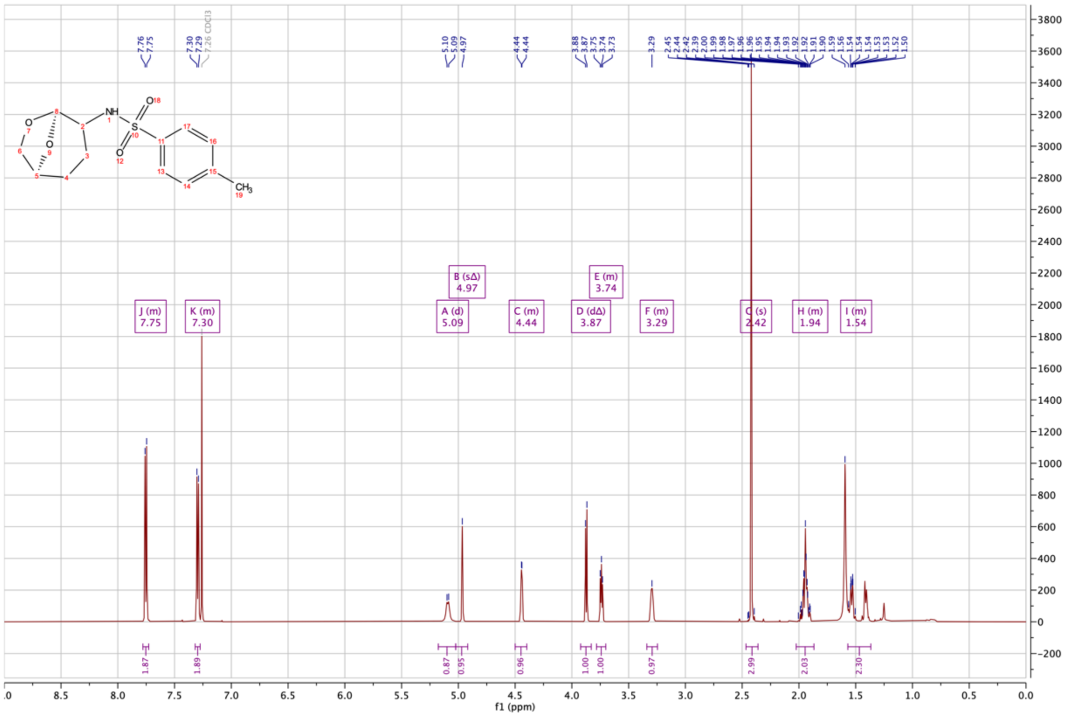

^{13}C-NMR spectrum of **12c**

^{13}C NMR (151 MHz, CDCl$_3$) δ 143.7, 138.2, 130.0, 127.0, 101.2, 73.2, 67.6, 51.0, 25.1, 22.0, 21.7.

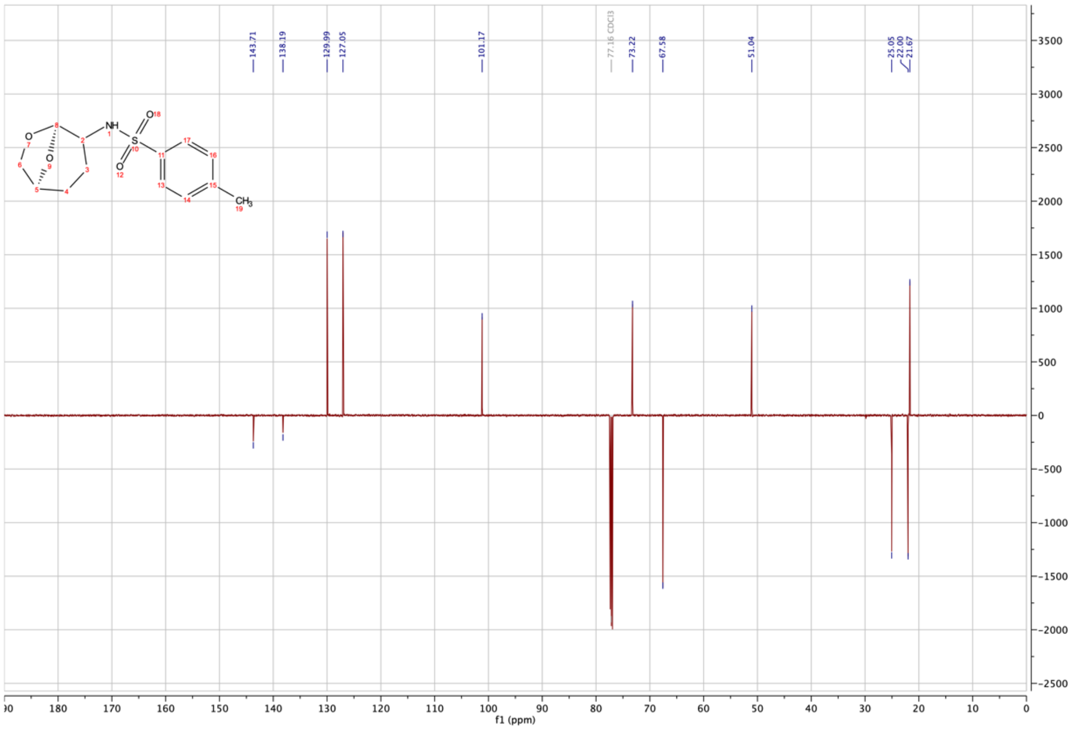

^{1}H-NMR spectrum of **12d**

^{1}H NMR (600 MHz, $CDCl_3$) δ 7.80 – 7.73 (m, 2H), 7.33 – 7.27 (m, 2H), 5.05 (s^{Δ}, 1H), 4.68 (d, J = 10.0 Hz, 1H), 4.48 – 4.42 (m, 1H), 3.80 (d^{Δ}, J = 7.2 Hz, 1H), 3.76 – 3.72 (m, 1H), 3.37 – 3.31 (m, 1H), 2.42 (s, 3H), 1.89 – 1.78 (m, 2H), 1.57 – 1.40 (m, 2H).

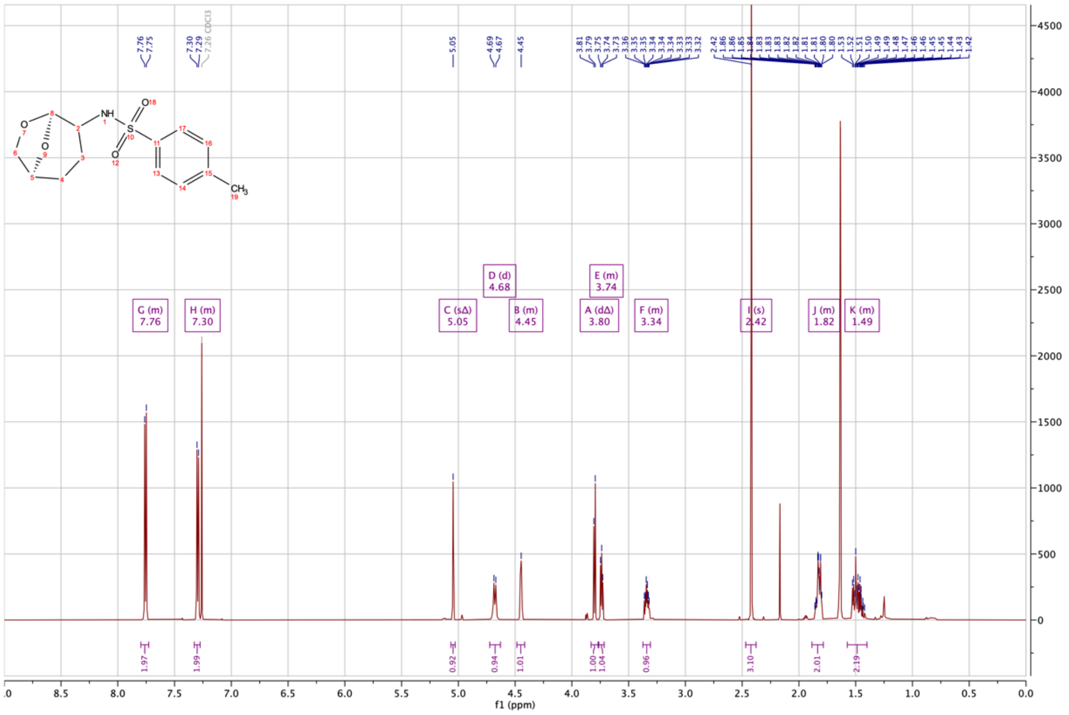

^{13}C-NMR spectrum of **12d**

^{13}C NMR (151 MHz, $CDCl_3$) δ 143.7, 138.5, 130.0, 127.0, 101.9, 73.0, 68.3, 53.0, 28.0, 24.7, 21.7.

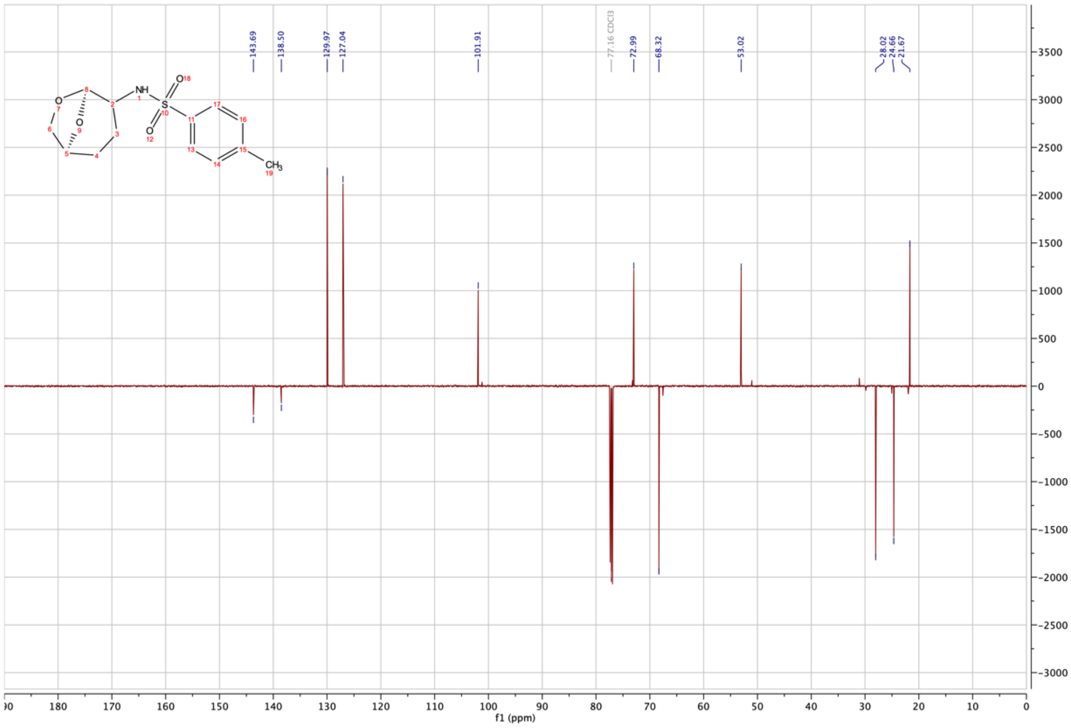

References

1. Jæger Pedersen, M.; Pedersen, C.M. Reactivity, Selectivity, and Synthesis of 4- *C* -Silylated Glycosyl Donors and 4-Deoxy Analogues. *Angew. Chemie Int. Ed.* **2021**, *60*, 2689–2693, doi:10.1002/anie.202009209.

2. Jung, M.E.; Kiankarimi, M. Synthesis of Methylene-Expanded 2',3'-Dideoxyribonucleosides. *J. Org. Chem.* **1998**, *63*, 8133–8144, doi:10.1021/jo980436l.

3. Figliolia, R.; Cavigli, P.; Comuzzi, C.; Del Zotto, A.; Lovison, D.; Strazzolini, P.; Susmel, S.; Zuccaccia, D.; Ballico, M.; Baratta, W. CNN Pincer Ruthenium Complexes for Efficient Transfer Hydrogenation of Biomass-Derived Carbonyl Compounds. *Dalt. Trans.* **2020**, *49*, 453–465, doi:10.1039/c9dt04292j.

4. Valeev, F.A.; Gorobets, E. V.; Tsypysheva, I.P.; Singizova, G.S.; Kalimullina, L.K.; Safarov, M.G.; Shitikova, O. V.; Miftakhov, M.S. Stereochemical Aspects of the Beckman Rearrangement of Oximes of Levoglucosenone and Its Dihydro Derivative. Enantioselective Synthesis of (+)-γ-Pelargonolactone. *Chem. Nat. Compd.* **2003**, *39*, 563–568, doi:10.1023/B:CONC.0000018110.36123.f2.

5. Zhao, F.; Zhang, L.D.; Hao, Y.; Chen, N.; Bai, R.; Wang, Y.J.; Zhang, C.C.; Li, G.S.; Hao, L.J.; Shi, C.; et al. Identification of 3-Substituted-6-(1-(1H-[1,2,3]Triazolo[4,5-b]Pyrazin-1-Yl)Ethyl)Quinoline Derivatives as Highly Potent and Selective Mesenchymal-Epithelial Transition Factor (c-Met) Inhibitors via Metabolite Profiling-Based Structural Optimization. *Eur. J. Med. Chem.* **2017**, *134*, 147–158, doi:10.1016/j.ejmech.2017.03.085.

6. Valeev, F.A.; Kalimullina, L.K.; Salikhov, S.M.; Shitikova, O. V.; Tsypysheva, I.P.; Safarov, M.G. Synthesis of 2-Amino Derivatives of Levoglucosenone. *Chem. Nat. Compd.* **2004**, *40*, 521–525, doi:10.1007/s10600-005-0052-8.

7. Kuhl, N.; Turnbull, B.W.H.; Ji, Y.; Larson, R.T.; Shevlin, M.; Prier, C.K.; Chung, C.K.; Desmond, R.; Guetschow, E.; He, C.Q.; et al. Utilizing Biocatalysis and a Sulfolane-Mediated Reductive Acetal Opening to Access Nemtabrutinib from Cyrene. *Green Chem* **2023**, doi:https://doi.org/10.1039/D2GC04117K.
